# An international genome-wide meta-analysis of primary biliary cholangitis: Novel risk loci and candidate drugs

**DOI:** 10.1016/j.jhep.2021.04.055

**Published:** 2021-09

**Authors:** Heather J. Cordell, James J. Fryett, Kazuko Ueno, Rebecca Darlay, Yoshihiro Aiba, Yuki Hitomi, Minae Kawashima, Nao Nishida, Seik-Soon Khor, Olivier Gervais, Yosuke Kawai, Masao Nagasaki, Katsushi Tokunaga, Ruqi Tang, Yongyong Shi, Zhiqiang Li, Brian D. Juran, Elizabeth J. Atkinson, Alessio Gerussi, Marco Carbone, Rosanna Asselta, Angela Cheung, Mariza de Andrade, Aris Baras, Julie Horowitz, Manuel A.R. Ferreira, Dylan Sun, David E. Jones, Steven Flack, Ann Spicer, Victoria L. Mulcahy, Jinyoung Byan, Younghun Han, Richard N. Sandford, Konstantinos N. Lazaridis, Christopher I. Amos, Gideon M. Hirschfield, Michael F. Seldin, Pietro Invernizzi, Katherine A. Siminovitch, Xiong Ma, Minoru Nakamura, George F. Mells, Canadian PBC Consortium, Katherine A. Siminovitch, Gideon M. Hirschfield, Andrew Mason, Catherine Vincent, Gang Xie, Jinyi Zhang, Chinese PBC Consortium, Ruqi Tang, Xiong Ma, Zhiqiang Li, Yongyong Shi, Italian PBC Study Group, Andrea Affronti, Piero L. Almasio, Domenico Alvaro, Pietro Andreone, Angelo Andriulli, Francesco Azzaroli, Pier Maria Battezzati, Antonio Benedetti, MariaConsiglia Bragazzi, Maurizia Brunetto, Savino Bruno, Vincenza Calvaruso, Vincenzo Cardinale, Giovanni Casella, Nora Cazzagon, Antonio Ciaccio, Barbara Coco, Agostino Colli, Guido Colloredo, Massimo Colombo, Silvia Colombo, Laura Cristoferi, Carmela Cursaro, Lory Saveria Crocè, Andrea Crosignani, Daphne D’Amato, Francesca Donato, Gianfranco Elia, Luca Fabris, Stefano Fagiuoli, Carlo Ferrari, Annarosa Floreani, Andrea Galli, Edoardo Giannini, Ignazio Grattagliano, Pietro Lampertico, Ana Lleo, Federica Malinverno, Clara Mancuso, Fabio Marra, Marco Marzioni, Sara Massironi, Alberto Mattalia, Luca Miele, Chiara Milani, Lorenzo Morini, Filomena Morisco, Luigi Muratori, Paolo Muratori, Grazia A. Niro, Sarah O’Donnell, Antonio Picciotto, Piero Portincasa, Cristina Rigamonti, Vincenzo Ronca, Floriano Rosina, Giancarlo Spinzi, Mario Strazzabosco, Mirko Tarocchi, Claudio Tiribelli, Pierluigi Toniutto, Luca Valenti, Maria Vinci, Massimo Zuin, Japan-PBC-GWAS Consortium, Hitomi Nakamura, Seigo Abiru, Shinya Nagaoka, Atsumasa Komori, Hiroshi Yatsuhashi, Hiromi Ishibashi, Masahiro Ito, Kiyoshi Migita, Hiromasa Ohira, Shinji Katsushima, Atsushi Naganuma, Kazuhiro Sugi, Tatsuji Komatsu, Tomohiko Mannami, Kouki Matsushita, Kaname Yoshizawa, Fujio Makita, Toshiki Nikami, Hideo Nishimura, Hiroshi Kouno, Hirotaka Kouno, Hajime Ota, Takuya Komura, Yoko Nakamura, Masaaki Shimada, Noboru Hirashima, Toshiki Komeda, Keisuke Ario, Makoto Nakamuta, Tsutomu Yamashita, Kiyoshi Furuta, Masahiro Kikuchi, Noriaki Naeshiro, Hironao Takahashi, Yutaka Mano, Seiji Tsunematsu, Iwao Yabuuchi, Yusuke Shimada, Kazuhiko Yamauchi, Rie Sugimoto, Hironori Sakai, Eiji Mita, Masaharu Koda, Satoru Tsuruta, Hiroshi Kamitsukasa, Takeaki Sato, Naohiko Masaki, Tatsuro Kobata, Nobuyoshi Fukushima, Yukio Ohara, Toyokichi Muro, Eiichi Takesaki, Hitoshi Takaki, Tetsuo Yamamoto, Michio Kato, Yuko Nagaoki, Shigeki Hayashi, Jinya Ishida, Yukio Watanabe, Masakazu Kobayashi, Michiaki Koga, Takeo Saoshiro, Michiyasu Yagura, Keisuke Hirata, Atsushu Tanaka, Hajime Takikawa, Mikio Zeniya, Masanori Abe, Morikazu Onji, Shuichi Kaneko, Masao Honda, Kuniaki Arai, Teruko Arinaga-Hino, Etsuko Hashimoto, Makiko Taniai, Takeji Umemura, Satoru Joshita, Kazuhiko Nakao, Tatsuki Ichikawa, Hidetaka Shibata, Satoshi Yamagiwa, Masataka Seike, Koichi Honda, Shotaro Sakisaka, Yasuaki Takeyama, Masaru Harada, Michio Senju, Osamu Yokosuka, Tatsuo Kanda, Yoshiyuki Ueno, Kentaro Kikuchi, Hirotoshi Ebinuma, Takashi Himoto, Michio Yasunami, Kazumoto Murata, Masashi Mizokami, Kazuhito Kawata, Shinji Shimoda, Yasuhiro Miyake, Akinobu Takaki, Kazuhide Yamamoto, Katsuji Hirano, Takafumi Ichida, Akio Ido, Hirohito Tsubouchi, Kazuaki Chayama, Kenichi Harada, Yasuni Nakanuma, Yoshihiko Maehara, Akinobu Taketomi, Ken Shirabe, Yuji Soejima, Akira Mori, Shintaro Yagi, Shinji Uemoto, Egawa H, Tomohiro Tanaka, Noriyo Yamashiki, Sumito Tamura, Yasuhiro Sugawara, Norihiro Kokudo, US PBC Consortium, Brian D. Juran, Elizabeth J. Atkinson, Angela Cheung, Mariza de Andrade, Konstantinos N. Lazaridis, Naga Chalasani, Vel Luketic, Joseph Odin, Kapil Chopra, Aris Baras, Julie Horowitz, Goncalo Abecasis, Michael Cantor, Giovanni Coppola, Aris Economides, Luca A. Lotta, John D. Overton, Jeffrey G. Reid, Alan Shuldiner, Christina Beechert, Caitlin Forsythe, Erin D. Fuller, Zhenhua Gu, Michael Lattari, Alexander Lopez, John D. Overton, Thomas D. Schleicher, Maria Sotiropoulos Padilla, Karina Toledo, Louis Widom, Sarah E. Wolf, Manasi Pradhan, Kia Manoochehri, Ricardo H. Ulloa, Xiaodong Bai, Suganthi Balasubramanian, Leland Barnard, Andrew Blumenfeld, Gisu Eom, Lukas Habegger, Alicia Hawes, Shareef Khalid, Jeffrey G. Reid, Evan K. Maxwell, William Salerno, Jeffrey C. Staples, Marcus B. Jones, Lyndon J. Mitnaul, UK-PBC Consortium, Richard Sturgess, Christopher Healey, Andrew Yeoman, Anton VJ. Gunasekera, Paul Kooner, Kapil Kapur, V. Sathyanarayana, Yiannis Kallis, Javaid Subhani, Rory Harvey, Roger McCorry, Paul Rooney, David Ramanaden, Richard Evans, Thiriloganathan Mathialahan, Jaber Gasem, Christopher Shorrock, Mahesh Bhalme, Paul Southern, Jeremy A. Tibble, David A. Gorard, Susan Jones, George Mells, Victoria Mulcahy, Brijesh Srivastava, Matthew R. Foxton, Carole E. Collins, David Elphick, Mazn Karmo, Francisco Porras-Perez, Michael Mendall, Tom Yapp, Minesh Patel, Roland Ede, Joanne Sayer, James Jupp, Neil Fisher, Martyn J. Carter, Konrad Koss, Jayshri Shah, Andrzej Piotrowicz, Glyn Scott, Charles Grimley, Ian R. Gooding, Simon Williams, Judith Tidbury, Guan Lim, Kuldeep Cheent, Sass Levi, Dina Mansour, Matilda Beckley, Coral Hollywood, Terry Wong, Richard Marley, John Ramage, Harriet M. Gordon, Jo Ridpath, Theodore Ngatchu, Vijay Paul Bob Grover, Ray G. Shidrawi, George Abouda, L. Corless, Mark Narain, Ian Rees, Ashley Brown, Simon Taylor-Robinson, Joy Wilkins, Leonie Grellier, Paul Banim, Debasish Das, Michael A. Heneghan, Howard Curtis, Helen C. Matthews, Faiyaz Mohammed, Mark Aldersley, Raj Srirajaskanthan, Giles Walker, Alistair McNair, Amar Sharif, Sambit Sen, George Bird, Martin I. Prince, Geeta Prasad, Paul Kitchen, Adrian Barnardo, Chirag Oza, Nurani N. Sivaramakrishnan, Prakash Gupta, Amir Shah, Chris DJ. Evans, Subrata Saha, Katharine Pollock, Peter Bramley, Ashis Mukhopadhya, Stephen T. Barclay, Natasha McDonald, Andrew J. Bathgate, Kelvin Palmer, John F. Dillon, Simon M. Rushbrook, Robert Przemioslo, Chris McDonald, Andrew Millar, Cheh Tai, Stephen Mitchell, Jane Metcalf, Syed Shaukat, Mary Ninkovic, Udi Shmueli, Andrew Davis, Asifabbas Naqvi, Tom JW. Lee, Stephen Ryder, Jane Collier, Howard Klass, Matthew E. Cramp, Nichols Sharer, Richard Aspinall, Deb Ghosh, Andrew C. Douds, Jonathan Booth, Earl Williams, Hyder Hussaini, John Christie, Steven Mann, Douglas Thorburn, Aileen Marshall, Imran Patanwala, Aftab Ala, Julia Maltby, Ray Matthew, Chris Corbett, Sam Vyas, Saket Singhal, Dermot Gleeson, Sharat Misra, Jeff Butterworth, Keith George, Tim Harding, Andrew Douglass, Harriet Mitchison, Simon Panter, Jeremy Shearman, Gary Bray, Michael Roberts, Graham Butcher, Daniel Forton, Zahid Mahmood, Matthew Cowan, Debashis Das, Chin Lye Ch’ng, Mesbah Rahman, Gregory C.A. Whatley, Emma Wesley, Aditya Mandal, Sanjiv Jain, Stephen P. Pereira, Mark Wright, Palak Trivedi, Fiona H. Gordon, Esther Unitt, Altaf Palejwala, Andrew Austin, Vishwaraj Vemala, Allister Grant, Andrew D. Higham, Alison Brind, Ray Mathew, Mark Cox, Subramaniam Ramakrishnan, Alistair King, Simon Whalley, Jocelyn Fraser, S.J. Thomson, Andrew Bell, Voi Shim Wong, Richard Kia, Ian Gee, Richard Keld, Rupert Ransford, James Gotto, Charles Millson

**Affiliations:** 25 Departments of Medicine, Immunology and Medical Sciences, University of Toronto, Canada; 26 Mount Sinai Hospital, Lunenfeld-Tanenbaum Research Institute, Canada; 27 Toronto General Research Institute, Toronto, Ontario, Canada; 28 Toronto Centre for Liver Disease, Division of Gastroenterology and Hepatology, University of Toronto, Toronto, Ontario, Canada; 29 Dept of Medicine, University of Alberta, Edmonton, Alberta, Canada; 30 Universite de Montreal Hospital Centre, Saint-Luc Hospital, Montreal, Quebec, Canada; 31 Lunenfeld Tanenbaum Research Institute, Toronto, Canada; 32 Lunenfeld Tanenbaum Research Institute, Toronto, Canada; 33 Division of Gastroenterology and Hepatology, Key Laboratory of Gastroenterology and Hepatology, Ministry of Health, State Key Laboratory for Oncogenes and Related Genes, Renji Hospital, School of Medicine, Shanghai Jiao Tong University, Shanghai Institute of Digestive Disease, China; 34 Bio-X Institutes, Key Laboratory for the Genetics of Developmental and Neuropsychiatric Disorders (Ministry of Education), Collaborative Innovation Center for Brain Science, Shanghai Jiao Tong University, China; 35 Affiliated Hospital of Qingdao University and Biomedical Sciences Institute of Qingdao University (Qingdao Branch of SJTU Bio-X Institutes), Qingdao University, China; 36 Azienda Ospedaliera Ospedali Riuniti Villa Sofia-Cervello, Palermo, Italy; 37 Gastroenterology & Hepatology Unit, Di.Bi.M.I.S., University of Palermo, Palermo, Italy; 38 Department of Medico-Surgical Sciences and Biotechnologies, Polo Pontino, University Sapienza of Rome Eleonora Lorillard Spencer-Cenci Foundation, Rome, Italy; 39 Department of Medical and Surgical Sciences, Bologna University, Bologna, Italy; 40 IRCCS Casa Sollievo della Sofferenza Hospital, San Giovanni Rotondo, Italy; 41 Department of Medical and Surgical Sciences (DIMEC) University of Bologna, Bologna, Italy; 42 San Paolo Hospital Medical School, Università di Milano, Milan, Italy; 43 Università Politecnica delle Marche, Ancona, Italy; 44 Department of Medico-Surgical Sciences and Biotechnologies, Polo Pontino, University Sapienza of Rome, Rome, Italy; 45 Azienda Ospedaliera Universitaria Pisana, Pisa, Italy; 46 Department of Internal Medicine, Ospedale Fatebene Fratelli e Oftalmico, Milan, Italy; 47 Sezione di Gastroenterologia e Epatologia, Dipartimento Biomedico di Medicina Interna e Specialistica (Di.Bi.M.I.S.) University of Palermo, Palermo, Italy; 48 Department of Medico-Surgical Sciences and Biotechnologies, Sapienza University of Rome, Viale dell’Università 37, 00185, Rome, Italy; 49 Medical Department, Desio Hospital, Desio, Italy; 50 Department of Surgery, Oncology and Gastroenterology, University of Padua, Padova, Italy; 51 Division of Gastroenterology and Center for Autoimmune Liver Diseases, Department of Medicine and Surgery, University of Milano-Bicocca, Monza, Italy; 52 Azienda Ospedaliera Universitaria Pisana, Pisa, Italy; 53 Department of Internal Medicine, AO Provincia di Lecco, Lecco, Italy; 54 Department of Internal Medicine, San Pietro Hospital, Bergamo, Ponte San Pietro, Italy; 55 Humanitas Clinical and Research Center, IRCCS, Rozzano, Italy; 56 Treviglio Hospital, Treviglio, Italy; 57 Division of Gastroenterology and Center for Autoimmune Liver Diseases, Department of Medicine and Surgery, University of Milano-Bicocca, Monza, Italy; 58 Hepatology Unit, Department of Medical and Surgical Sciences, University Hospital of Bologna, Italy; 59 University of Trieste, & Fondazione Italiana Fegato (FIF) Trieste, Italy; 60 San Paolo Hospital Medical School, Università di Milano, Milan, Italy; 61 Division of Gastroenterology and Center for Autoimmune Liver Diseases, Department of Medicine and Surgery, University of Milano-Bicocca, Monza, Italy; 62 Fondazione IRCCS Ca’ Granda, Ospedale Maggiore Policlinico, Milan, Italy; 63 Azienda Ospedaliero-Universitaria di Parma, Parma, Italy; 64 University of Padova, Padova, Italy; 65 Gastroenterologia Epatologia e Trapiantologia, Papa Giovanni XXIII Hospital, Bergamo, Italy; 66 Azienda Ospedaliero-Universitaria di Parma, Parma, Italy; 67 Department. of Surgical, Oncological and Gastroenterological Sciences, University of Padova, Padova, Italy; 68 University of Florence, Florence, Italy; 69 Gastroenterology Unit, Department Internal Medicine, Policlinico San Martino, University of Genoa, Genoa, Italy; 70 Italian College of General Practicioners, ASL Bari, Italy; 71 Division of Gastroenterology and Hepatology, Fondazione IRCCS Ca’ Granda Ospedale Maggiore Policlinico, Milan, Italy; 72 Department of Biomedical Sciences, Humanitas University, Division of Internal Medicine and Hepatology, Department of Gastroenterology, Humanitas Clinical and Research Center IRCCS, Via A. Manzoni 56, 20089 Rozzano (MI), Italy; 73 Division of Gastroenterology and Center for Autoimmune Liver Diseases, Department of Medicine and Surgery, University of Milano-Bicocca, Monza, Italy; 74 Division of Gastroenterology and Center for Autoimmune Liver Diseases, Department of Medicine and Surgery, University of Milano-Bicocca, Monza, Italy; 75 University of Florence, Florence, Italy; 76 Università Politecnica delle Marche, Ancona, Italy; 77 Division of Gastroenterology and Center for Autoimmune Liver Diseases, Department of Medicine and Surgery, University of Milano-Bicocca, Monza, Italy; 78 Santa Croce Carle Hospital, Cuneo, Italy; 79 Internal Medicine, Gastroenterology and Liver Unit, A. Gemelli Polyclinic, Sacro Cuore Catholic University, 20123 Rome, Italy; 80 Division of Gastroenterology and Center for Autoimmune Liver Diseases, Department of Medicine and Surgery, University of Milano-Bicocca, Monza, Italy; 81 Magenta Hospital, Magenta, Italy; 82 University of Naples, Federico II, Naples, Italy; 83 Department of Clinical Medicine, University of Bologna, Bologna, Italy; 84 Department of Clinical Medicine, University of Bologna, Bologna, Italy; 85 IRCCS Casa Sollievo della Sofferenza Hospital, San Giovanni Rotondo, Italy; 86 Division of Gastroenterology and Center for Autoimmune Liver Diseases, Department of Medicine and Surgery, University of Milano-Bicocca, Monza, Italy; 87 University of Genoa, Genoa, Italy; 88 Department of Interdisciplinary Medicine, University Medical School, Bari, Italy; 89 Department of Translational Medicine, Università del Piemonte Orientale UPO, 28100 Novara, Italy; 90 Division of Gastroenterology and Center for Autoimmune Liver Diseases, Department of Medicine and Surgery, University of Milano-Bicocca, Monza, Italy; 91 Division of Gastroenterology & Hepatology, Center for Predictive Medicine, Gradenigo Hospital, Turin, Italy; 92 Azienda Ospedaliera Valduce, Como, Italy; 93 Yale University, New Haven, Connecticut, USA; 94 University of Florence, Florence, Italy; 95 University of Trieste, & Fondazione Italiana Fegato (FIF) Trieste, Italy; 96 University of Udine, Udine, Italy; 97 Internal Medicine and Metabolic Diseases, Fondazione IRCCS Ca’ Granda Ospedale Policlinico Milano, Department of Pathophysiology and Transplantation, Università degli Studi di Milano, Milan, Italy; 98 Ospedale Niguarda, Milan, Italy; 99 San Paolo Hospital Medical School, Università di Milano, Milan, Italy; 100 Clinical Research Center, National Hospital Organization (NHO) Nagasaki Medical Center, Omura, Japan; 101 Department of Gastroenterology and Rheumatic Diseases, Fukushima Medical University of Medicine, Fukushima, Japan; 102 Headquaters of PBC Research in the NHO Study Group for Liver Disease in Japan (NHOSLJ), Clinical Research Center, NHO Nagasaki Medical Center, Omura, Nagasaki, Japan; 103 Department of Medicine, Teikyo University School of Medicine, Tokyo, Japan; 104 Department of Gastroenterology and Hepatology, Tokyo Jikei University School of Medicine, Tokyo, Japan; 105 Department of Gastroenterology and Metabology, Ehime University Graduate School of Medicine, Matsuyama, Japan; 106 Department of Gastroenterology, Kanazawa University Graduate School of Medicine, Kanazawa, Japan; 107 Division of Gastroenterology, Department of Medicine, Kurume University School of Medicine, Kurume, Japan; 108 Department of Medicine and Gastroenterology, Tokyo Women’s Medical University, Tokyo, Japan; 109 Department of Medicine, Division of Gastroenterology and Hepatology, Shinshu University School of Medicine, Matsumoto, Japan; 110 Department of Gastroenterology and Hepatology, Nagasaki University Graduate School of Biomedical Sciences, Nagasaki, Japan; 111 Division of Gastroenterology and Hepatology,Niigata University Graduate School of Medical and Dental Sciences, Niigata, Japan; 112 Faculty of Medicine, Oita University, Oita, Japan; 113 Department of Gastroenterology and Medicine, Fukuoka University School of Medicine, Fukuoka, Japan; 114 The Third Department of Internal Medicine, School of Medicine, University of Occupational and Environmental Health, Kitakyushu, Japan; 115 Department of Medicine and Clinical Oncology, Graduate School of Medicine, Chiba University, Chiba, Japan; 116 Department of Gastroenterology, Yamagata University Faculty of Medicine, Yamagata, Japan; 117 Department of Internal Medicine, Teikyo University Mizonokuchi Hospital, Kawasaki, Japan; 118 Division of Gastroenterology and Hepatology, Department of Internal Medicine, Keio Graduate School of Medicine, Tokyo, Japan; 119 Department of Medical Technology, Kagawa Prefectural University of Health Sciences, Kagawa, Japan; 120 Department of Clinical Medicine, Institute of Tropical Medicine, Nagasaki University, Nagasakin, Japan; 121 The Research Center for Hepatitis and Immunology, National Center for Global Health and Medicine, Ichikawa, Japan; 122 Hepatology Division, Department of Internal Medicine II, Hamamatsu University School of Medicine, Hamamatsu, Shizuoka Japan; 123 Department of Medicine and Biosystemic Science, Kyushu University Graduate School of Medical Sciences, Fukuoka, Japan; 124 Department of Gastroenterology and Hepatology, Okayama University Graduate School of Medicine, Dentistry and Pharmaceutical Sciences, Okayama, Japan; 125 Department of Gastroenterology and Hepatology, Juntendo University Shizuoka Hospital, Shizuoka, Japan; 126 Department of Digestive and Lifestyle–Related Disease, Kagoshima University Graduate School of Medical and Dental Science, Kagoshima, Japan; 127 Department of Gastroenterology and Metabolism, Applied Life Sciences, Institute of Biomedical & Health Sciences, Hiroshima University, Hiroshima, Japan; 128 Department of Human Pathology, Kanazawa University Graduate School of Medicine, Kanazawa, Japan; 129 Department of Surgery and Science, Kyushu University Graduate School of Medical Sciences, Fukuoka, Japan; 130 Division of Hepato-Biliary-Pancreatic and Transplant Surgery, Department of Surgery, Graduate School of Medicine, Kyoto University, Kyoto, Japan; 131 Department of Surgery, Tokyo Women’s Medical University, Tokyo, Japan; 132 Organ Transplantation Service, The University of Tokyo, Tokyo, Japan; 133 Hepatobiliary and Pancreatic Surgery Division and Artificial Organ and Transplantation Division, Department of Surgery, Graduate School of Medicine, The University of Tokyo, Japan; 134 Division of Gastroenterology and Hepatology, Mayo Clinic, Rochester, Minnesota, United States; 135 Division of Biomedical Statistics and Informatics Mayo Clinic, Rochester, Minnesota, United States; 136 Division of Gastroenterology and Hepatology, Mayo Clinic, Rochester, Minnesota, United States; 137 Division of Biomedical Statistics and Informatics Mayo Clinic, Rochester, Minnesota, United States; 138 Division of Gastroenterology and Hepatology, Mayo Clinic, Rochester, Minnesota, United States; 139 Indiana University, Indiana, United States; 140 Virginia Commonwealth University, Virginia, United States; 141 Icahn School of Medicine, Mount Sinai, New York, United States; 142 University of Pittsburgh, United States; 143 Regeneron, United States; 144 Aintree University Hospital, Aintree University Hospitals NHS Foundation Trust, United Kingdom; 145 Airedale General Hospital, Airedale NHS Foundation Trust, United Kingdom; 146 Nevill Hall Hospital, Royal Gwent Hospital, Ysbyty Ystrad Fawr, Aneurin Bevan University Health Board, United Kingdom; 147 Ashford Hospital, St Peter’s Hospital, Ashford and St Peter’s NHS Foundation Trust, United Kingdom; 148 Queen’s Hospital, King George Hospital, Barking, Havering and Redbridge University Hospitals NHS Trust, United Kingdom; 149 Barnsley Hospital, Barnsley Hospital NHS Foundation Trust, United Kingdom; 150 Newham University Hospital, St Bartholemew’s Hospital, The Royal London Hospital, Whipps Cross University Hospital, Barts Health NHS Trust, United Kingdom; 151 Basildon University Hospital, Basildon and Thurrock University Hospitals NHS Foundation Trust, United Kingdom; 152 Bedford Hospital, Bedford Hospitals NHS Trust, United Kingdom; 153 Royal Victoria Hospital, Belfast Health and Social Care Trust, United Kingdom; 154 Glan Clwyd Hospital, Betsi Cadwaladr University Health Board, United Kingdom; 155 Llandudno General Hospital, Betsi Cadwaladr University Health Board, United Kingdom; 156 Wrexham Maelor Hospital, Betsi Cadwaladr University Health Board, United Kingdom; 157 Ysbyty Gwynedd, Betsi Cadwaladr University Health Board, United Kingdom; 158 Blackpool Victoria Hospital, Blackpool Teaching Hospitals NHS Foundation Trusts, United Kingdom; 159 Royal Bolton Hospital, Bolton NHS Foundation Trust, United Kingdom; 160 Bradford Royal Infirmary, Bradford Teaching Hospitals NHS Foundation Trust, United Kingdom; 161 Royal Sussex County Hospital, Brighton and Sussex University Hospitals NHS Trust, United Kingdom; 162 Amersham Hospital, Stoke Mandeville Hospital, Wycombe Hospital, Buckinghamshire Healthcare NHS Trust, United Kingdom; 163 Calderdale Royal Hospital, Huddersfield Royal Infirmary, Calderdale and Huddersfield NHS trust, United Kingdom; 164 Addenbrooke’s Hospital, Cambridge University Hospitals NHS Foundation Trust, United Kingdom; 165 University Hospital Llandough, University Hospital of Wales, Cardiff and Vale University Health Board, United Kingdom; 166 Chelsea and Westminster Hospital, Chelsea & Westminster NHS Foundation Trust, United Kingdom; 167 West Middlesex University Hospital, Chelsea & Westminster NHS Foundation Trust, United Kingdom; 168 Chesterfield Royal Hospital, Chesterfield Royal Hospital NHS Foundation Trust, United Kingdom; 169 Countess of Chester Hospital, Countess of Chester Hospital NHS Foundation Trust, United Kingdom; 170 Darlington Memorial Hospital, University Hospital of North Durham, County Durham and Darlington NHS Foundation Trust, United Kingdom; 171 Croydon University Hospital, Purley War Memorial Hospital, Croydon Health Services NHS Trust, United Kingdom; 172 Princess of Wales Hospital, Cwm Taf Morgannwg University Health Board, United Kingdom; 173 Prince Charles Hospital, Royal Glamorgan Hospital, Ysbyty Cwm Cynon, Cwm Taf Morgannwg University Health Board, United Kingdom; 174 Darent Valley Hospital, Queen Mary’s Hospital Sidcup, Dartford And Gravesham NHS Trust, United Kingdom; 175 Bassetlaw Hospital, Doncaster Royal Infirmary, Doncaster and Bassetlaw Hospitals NHS Foundation Trust, United Kingdom; 176 Dorset County Hospital, Dorset County Hospital NHS Foundation Trust, United Kingdom; 177 Russells Hall Hospital, Dudley Group of Hospitals NHS Trust, United Kingdom; 178 Lister Hospital, Queen Elizabeth II Hospital, East and North Hertfordshire NHS Trust, United Kingdom; 179 Macclesfield District General Hospital, East Cheshire NHS Trust, United Kingdom; 180 Buckland Hospital, Kent and Canterbury Hospital, Queen Elizabeth The Queen Mother Hospital, William Harvey Hospital Ashford, East Kent Hospitals University NHS Foundation Trust, United Kingdom; 181 Burnley General Hospital, Royal Blackburn Hospital, East Lancashire Hospitals NHS Trust, United Kingdom; 182 Colchester General Hospital, East Suffolk and North Essex NHS Foundation Trust, United Kingdom; 183 Ipswich Hospital, East Suffolk and North Essex Foundation Trust, United Kingdom; 184 Conquest Hospital, Eastbourne District General Hospital, East Sussex Healthcare NHS Trust, United Kingdom; 185 Epsom General Hospital, Epsom and St Helier University Hospitals NHS Trust, United Kingdom; 186 Frimley Park Hospital, Frimley Health NHS Foundation Trust, United Kingdom; 187 Heatherwood Hospital, Wexham Park Hospital, Frimley Health NHS Foundation Trust, United Kingdom; 188 Queen Elizabeth Hospital, Gateshead Health NHS Foundation Trust, United Kingdom; 189 George Eliot Hospital, George Eliot Hospital NHS Trust, United Kingdom; 190 Cheltenham General Hospital, Gloucestershire Royal Hospital, Gloucestershire Hospitals NHS Foundation Trust, United Kingdom; 191 Guy’s Hospital, St Thomas’ Hospital, Guy’s and St Thomas’ NHS Foundation Trust, United Kingdom; 192 Basingstoke and North Hampshire Hospital, Hampshire Hospitals Foundation trust, United Kingdom; 193 Royal Hampshire County Hospital, Hampshire Hospitals NHS Foundation Trust, United Kingdom; 194 Harrogate District Hospital, Harrogate and District Foundation Trust, United Kingdom; 195 Good Hope Hospital, Heartlands Hospital, Solihull Hospital, Heart of England NHS Foundation Trust, United Kingdom; 196 Hillingdon Hospital, Hillingdon Hospitals NHS Foundation Trust, United Kingdom; 197 Homerton University Hospital, Homerton University Hospital NHS Foundation Trust, United Kingdom; 198 Castle Hill Hospital, Hull Royal Infirmary, Hull University Teaching Hospitals NHS Trust, United Kingdom; 199 Bronglais Hospital, Hywel Dda University Health Board, United Kingdom; 200 Prince Philip Hospital, Withybush General Hospital, Central Middlesex Hospital, Glangwili General Hospital, Hywel Dda University Health Board, United Kingdom; 201 Charing Cross Hospital, Hammersmith Hospital, St Mary’s Hospital, Imperial College, Imperial College Healthcare NHS Trust, United Kingdom; 202 St Mary’s Hospital, Imperial College, Imperial College Healthcare NHS Trust, United Kingdom; 203 St Mary’s Hospital, Isle of Wight, Isle of Wight NHS Trust, United Kingdom; 204 James Paget University Hospital, James Paget University Hospitals NHS Foundation Trust, United Kingdom; 205 Kettering General Hospital, Kettering General Hospital NHS Foundation Trust, United Kingdom; 206 King’s College Hospital, King’s College Hospital, United Kingdom; 207 Beckenham Beacon, Orpington Hospital, Princess Royal University Hospital, King’s College Hospital NHS Foundation Trust, United Kingdom; 208 Kingston Hospital, Kingston Hospital NHS Foundation Trust, United Kingdom; 209 Chorley and South Ribble Hospital, Royal Preston Hospital, Lancashire Teaching Hospitals NHS Foundation Trust, United Kingdom; 210 St James’s University Hospital, Leeds Teaching Hospitals NHS Trust, United Kingdom; 211 Lewisham Hospital, University Hospital Lewisham, Lewisham and Greenwich NHS Trust, United Kingdom; 212 Queen Elizabeth Hospital, Lewisham and Greenwich NHS Trust, United Kingdom; 213 Central Middlesex Hospital, Northwick Park and St Mark’s Hospitals, London North West Healthcare NHS Trust, United Kingdom; 214 Luton and Dunstable University Hospital, Luton and Dunstable University Hospital, United Kingdom; 215 Maidstone Hospital, Tunbridge Wells Hospital, Maidstone and Tunbridge Wells NHS Trust, United Kingdom; 216 Manchester Royal Infirmary, Trafford General Hospital, Manchester University NHS Foundation Trust, United Kingdom; 217 Wythenshawe Hospital, Manchester University NHS Foundation Trust, United Kingdom; 218 Medway Maritime Hospital, Medway NHS Foundation Trust, United Kingdom; 219 Broomfield Hospital, St Peters Hospital, Mid-Essex Hospitals NHS Trust, United Kingdom; 220 Dewsbury and District Hospital, Mid Yorkshire Hospitals NHS Trust, United Kingdom; 221 Milton Keynes Hospital, Milton Keynes Hospital NHS Foundation Trust, United Kingdom; 222 University Hospital Crosshouse, NHS Ayrshire & Arran, United Kingdom; 223 Borders General Hospital, NHS Borders, United Kingdom; 224 Dumfries and Galloway Royal Infirmary, NHS Dumfries & Galloway, United Kingdom; 225 Queen Margaret Hospital, Victoria Hospital, NHS Fife, United Kingdom; 226 Falkirk Community Hospital, Forth Valley Royal Hospital, Stirling Community Hospital, NHS Forth Valley, United Kingdom; 227 Aberdeen Royal Infirmary, Dr Gray’s Hospital, NHS Grampian, United Kingdom; 228 Gartnavel General Hospital, Glasgow Royal Infirmary, Inverclyde Royal Hospital, Royal Alexandra Hospital, Southern General Hospital, Victoria Infirmary, NHS Greater Glasgow and Clyde, United Kingdom; 229 Hairmyres Hospital, Monklands Hospital, Wishaw General Hospital, NHS Lanarkshire, United Kingdom; 230 Royal Infirmary of Edinburgh, St John’s Hospital, NHS Lothian, United Kingdom; 231 Western General Hospital, NHS Lothian, United Kingdom; 232 Ninewells Hospital, NHS TaWyside, United Kingdom; 233 Norfolk and Norwich University Hospital, Norfok and Norwich University Hospitals NHS Foundation Trust, United Kingdom; 234 Southmead Hospital, North Bristol NHS Trust, United Kingdom; 235 Cumberland Infirmary, West Cumberland Hospital, North Cumbria University Hospitals NHS Foundation Trust, United Kingdom; 236 North Middlesex Hospital, North Middlesex University Hospital NHS Trust; 237 University Hospital of Hartlepool, University Hospital of North Tees, North Tees and Hartlepool NHS Foundation Trust, United Kingdom; 238 Hinchingbrooke Hospital, North West Anglia NHS Trust; 239 Peterborough City Hospital, Stamford & Rutland Hospital, North West Anglia NHS Foundation Trust, United Kingdom; 240 Northampton General Hospital, Northampton General Hospital NHS Trust, United Kingdom; 241 North Devon District Hospital, Northern Devon Healthcare NHS Trust, United Kingdom; 242 Diana Princess of Wales Hospital, Scunthorpe General Hospital, Northern Lincolnshire and Goole NHS Foundation Trust, United Kingdom; 243 North Tyneside General Hospital, Northumbria Healthcare NHS Foundation Trust, United Kingdom; 244 Nottingham City Hospital, Queen’s Medical Centre, NIHR Nottingham Biomedical Research Centre at Nottingham University Hospitals NHS Trust, United Kingdom; 245 John Radcliffe Hospital, Oxford University Hospitals NHS Trust, United Kingdom; 246 Fairfield General Hospital, North Manchester General Hospital, Rochdale Infirmary, The Royal Oldham Hospital, Pennine Acute Hospitals NHS Trust, United Kingdom; 247 Derriford Hospital, University Hospitals Plymouth NHS Trust, United Kingdom; 248 Poole Hospital, Poole Hospital NHS Foundation Trust, United Kingdom; 249 Queen Alexandra Hospital, Portsmouth Hospitals NHS Trust, United Kingdom; 250 St Margaret’s Hospital, The Princess Alexandra Hospital, Princess Alexandra Hospital NHS Trust, United Kingdom; 251 The Queen Elizabeth Hospital King’s Lynn, King’s Lynn NHS Foundation Trust, United Kingdom; 252 Royal Berkshire Hospital, Royal Berkshire NHS Foundation Trust, United Kingdom; 253 Royal Bournemouth Hospital, Royal Bournemouth and Christchurch Hospitals NHS Foundation Trust, United Kingdom; 254 Royal Cornwall Hospital, Royal Cornwall Hospitals NHS Trust, United Kingdom; 255 Royal Devon and Exeter Hospital, Royal Devon And Exeter Nhs Foundation Trust, United Kingdom; 256 Barnet and Chase Farm Hospitals, Royal Free London NHS Trust, United Kingdom; 257 The Royal Free Hospital, Royal Free London NHS Foundation Trust, United Kingdom; 258 Royal Liverpool University Hospital, Royal Liverpool and Broadgreen University Hospitals NHS Trust, United Kingdom; 259 Royal Surrey County Hospital, Royal Surrey County Hospital NHS Foundation Trust, United Kingdom; 260 Royal United Bath Hospital, Royal United Hospitals Bath NHS Foundation Trust, United Kingdom; 261 Cannock Chase Hospital, Royal Wolverhampton Hospitals NHS Trust, United Kingdom; 262 New Cross Hospital, Royal Wolverhampton NHS Trust, United Kingdom; 263 Salisbury District Hospital, Salisbury NHS Foundation Trust, United Kingdom; 264 Sandwell General Hospital, Sandwell & West Birmingham Hospitals NHS Trust, United Kingdom; 265 Northern General Hospital, Royal Hallamshire Hospital, Sheffield Teaching Hospitals NHS Foundation Trust, United Kingdom; 266 King’s Mill Hospital, Newark Hospital, Sherwood Forest Hospitals NHS Foundation Trust, United Kingdom; 267 Princess Royal Hospital, Royal Shrewsbury Hospital, Shrewsbury and Telford Hospital NHS Trust, United Kingdom; 268 Torbay Hospital, Torbay and South Devon NHS Foundation Trust, United Kingdom; 269 Lagan Valley Hospital, Ulster Hospital, South Eastern Health and Social Care Trust, United Kingdom; 270 Friarage Hospital, The James Cook University Hospital, South Tees Hospitals NHS Foundation Trust, United Kingdom; 271 Sunderland Royal Hospital, Sunderland Royal Hospital, South Tyneside and Sunderland NHS Foundation Trust, United Kingdom; 272 South Tyneside District Hospital, South Tyneside and Sunderland NHS Foundation Trust, United Kingdom; 273 Warwick Hospital, South Warwickshire NHS Foundation Trust, United Kingdom; 274 Southend University Hospital, Southend University Hospital NHS Foundation Trust, United Kingdom; 275 Ormskirk District General Hospital, Southport and Formby District General Hospital, Southport & Ormskirk Hospital NHS Trust, United Kingdom; 276 St George’s Hospital, St George’s University Hospitals NHS Foundation Trust, United Kingdom; 277 Stepping Hill Hospital, Stockport NHS Foundation Trust, United Kingdom; 278 East Surrey Hospital, Surrey and Sussex Healthcare NHS Trust, United Kingdom; 279 Morriston Hospital, Singleton Hospital, Swansea Bay University Health Board, United Kingdom; 280 Neath Port Talbot Hospital, Swansea Bay University Health Board, United Kingdom; 281 Tameside General Hospital, Tameside and Glossop integrated care NHS Trust, United Kingdom; 282 Musgrove Park Hospital, Taunton and Somerset NHS Foundation Trust, United Kingdom; 283 Lincoln County Hospital, United Lincolnshire Hospitals NHS Trust, United Kingdom; 284 Pilgrim Hospital Boston, United Lincolnshire Hospitals NHS Trust, United Kingdom; 285 University College Hospital, University College London Hospitals NHS Foundation Trust, United Kingdom; 286 Southampton General Hospital, University Hospital Southampton NHS Foundation Trust, United Kingdom; 287 The Queen Elizabeth Hospital, University Hospitals Birmingham NHS Foundation Trust, United Kingdom; 288 Bristol Royal Infirmary, University Hospitals Bristol NHS Foundation Trust, United Kingdom; 289 University Hospital, Coventry, University Hospital Coventry and Warwickshire NHS Trust, United Kingdom; 290 Queen’s Hospital, University Hospitals of Derby and Burton NHS Foundation Trust, United Kingdom; 291 Royal Derby Hospital, University Hospitals of Derby and Burton NHS Foundation Trust, United Kingdom; 292 Glenfield Hospital, Leicester General Hospital, Leicester Royal Infirmary, University Hospitals of Leicester NHS Trust, United Kingdom; 293 Royal Lancaster Infirmary, University Hospitals of Morecambe Bay NHS Foundation Trust, United Kingdom; 294 Royal Stoke University Hospital, University Hospitals of North Midlands NHS Trust, United Kingdom; 295 County Hospital, University Hospitals of North Midlands NHS Trust, United Kingdom; 296 Walsall Manor Hospital, Walsall Healthcare NHS Trust, United Kingdom; 297 Warrington Hospital, Warrington and Halton Hospitals NHS Foundation Trust, United Kingdom; 298 Hemel Hempstead General Hospital, St Albans City Hospital, Watford General Hospital, West Hertfordshire Hospitals NHS Trust, United Kingdom; 299 West Suffolk Hospital, West Suffolk NHS Foundation Trust, United Kingdom; 300 St Richard’s Hospital, Western Sussex Hospitals NHS Foundation Trust, United Kingdom; 301 Worthing Hospital, Western Sussex Hospitals NHS Foundation Trust, United Kingdom; 302 Weston General Hospital, Weston Area Health Trust, United Kingdom; 303 The Whittington Hospital, Whittington Hospital NHS Trust, United Kingdom; 304 Arrowe Park Hospital, Victoria Central Hospital, Wirral University Teaching Hospital NHS Foundation Trust, United Kingdom; 305 Alexandra Hospital, Kidderminster Hospital and Treatment Centre, Worcestershire Royal Hospital, Worcestershire Acute Hospitals NHS trust, United Kingdom; 306 Royal Albert Edward Infirmary, Wrightington, Wigan And Leigh NHS Trust, United Kingdom; 307 The County Hospital, Wye Valley NHS Trust, United Kingdom; 308 Yeovil District Hospital, Yeovil District Hospital NHS Foundation Trust, United Kingdom; 309 Bridlington Hospital, Scarborough Hospital, The York Hospital, York Teaching Hospitals NHS Foundation Trust, United Kingdom; 1 Population Health Sciences Institute, Faculty of Medical Sciences, Newcastle University, Newcastle upon Tyne, United Kingdom; 2 Genome Medical Science Project, National Center for Global Health and Medicine (NCGM), Tokyo, Japan; 3 Clinical Research Center, National Hospital Organization, Nagasaki Medical Center, Omura, Japan; 4 Department of Human Genetics, Graduate School of Medicine, The University of Tokyo, Tokyo, Japan; 5 Human Biosciences Unit for the Top Global Course Center for the Promotion of Interdisciplinary Education and Research, Kyoto University, Kyoto, Japan; 6 Center for Genomic Medicine, Graduate School of Medicine, Kyoto University, Kyoto, Japan; 7 Division of Gastroenterology and Hepatology, Key Laboratory of Gastroenterology and Hepatology, Ministry of Health, State Key Laboratory for Oncogenes and Related Genes, Renji Hospital, School of Medicine, Shanghai JiaoTong University, Shanghai Institute of Digestive Disease, Shanghai, China; 8 Bio-X Institutes, Key Laboratory for the Genetics of Developmental and Neuropsychiatric Disorders (Ministry of Education), Collaborative Innovation Center for Brain Science, Shanghai Jiao Tong University, Shanghai, China; 9 Affiliated Hospital of Qingdao University and Biomedical Sciences Institute of Qingdao University (Qingdao Branch of SJTU Bio-X Institutes), Qingdao University, Qingdao, China; 10 Division of Gastroenterology and Hepatology, Mayo Clinic, Rochester, Minnesota, United States; 11 Division of Biomedical Statistics and Informatics, Mayo Clinic, Rochester, Minnesota, United States; 12 Division of Gastroenterology and Center for Autoimmune Liver Diseases, Department of Medicine and Surgery, University of Milano-Bicocca, Monza, Italy; 13 European Reference Network on Hepatological Diseases (ERN RARE-LIVER), San Gerardo Hospital, Monza, Italy; 14 Department of Biomedical Sciences, Humanitas University, Pieve Emanuele, Milan, Italy; 15 Humanitas Clinical and Research Center, IRCCS, Rozzano, Milan, Italy; 16 Regeneron Genetics Center, Tarrytown, New York, United States; 17 Translational and Clinical Research Institute, Faculty of Medical Sciences, Newcastle University, Newcastle upon Tyne, United Kingdom; 18 Academic Department of Medical Genetics, University of Cambridge, Cambridge, United Kingdom; 19 Institute for Clinical and Translational Research, Baylor College of Medicine, Houston, Texas, United States; 20 Toronto Centre for Liver Disease, Division of Gastroenterology and Hepatology, University of Toronto, Toronto, Ontario, Canada; 21 University of California, Davis, California, United States; 22 Departments of Medicine, Immunology and Medical Sciences, University of Toronto, Toronto, Ontario, Canada; 23 Mount Sinai Hospital, Lunenfeld-Tanenbaum Research Institute and Toronto General Research Institute, Toronto, Ontario, Canada; 24 Department of Hepatology, Nagasaki Graduate School of Biomedical Sciences, Japan

**Keywords:** UK-PBC, ERN RARE-LIVER, ALSPAC, Genomic co-localization, Network-based *in silico* drug efficacy screening

## Abstract

**Backgrounds & Aims:**

Primary biliary cholangitis (PBC) is a chronic liver disease in which autoimmune destruction of the small intrahepatic bile ducts eventually leads to cirrhosis. Many patients have inadequate response to licensed medications, motivating the search for novel therapies. Previous genome-wide association studies (GWAS) and meta-analyses (GWMA) of PBC have identified numerous risk loci for this condition, providing insight into its aetiology. We undertook the largest GWMA of PBC to date, aiming to identify additional risk loci and prioritise candidate genes for *in silico* drug efficacy screening.

**Methods:**

We combined new and existing genotype data for 10,516 cases and 20,772 controls from 5 European and 2 East Asian cohorts.

**Results:**

We identified 56 genome-wide significant loci (20 novel) including 46 in European, 13 in Asian, and 41 in combined cohorts; and a 57^th^ genome-wide significant locus (also novel) in conditional analysis of the European cohorts. Candidate genes at newly identified loci include *FCRL3*, *INAVA*, *PRDM1*, *IRF7*, *CCR6*, *CD226*, and *IL12RB1*, which each play key roles in immunity. Pathway analysis reiterated the likely importance of pattern recognition receptor and TNF signalling, JAK-STAT signalling, and differentiation of T helper (T_H_)1 and T_H_17 cells in the pathogenesis of this disease. Drug efficacy screening identified several medications predicted to be therapeutic in PBC, some of which are well-established in the treatment of other autoimmune disorders.

**Conclusions:**

This study has identified additional risk loci for PBC, provided a hierarchy of agents that could be trialled in this condition, and emphasised the value of genetic and genomic approaches to drug discovery in complex disorders.

**Lay summary:**

Primary biliary cholangitis (PBC) is a chronic liver disease that eventually leads to cirrhosis. In this study, we analysed genetic information from 10,516 people with PBC and 20,772 healthy individuals recruited in Canada, China, Italy, Japan, the UK, or the USA. We identified several genetic regions associated with PBC. Each of these regions contains several genes. For each region, we used diverse sources of evidence to help us choose the gene most likely to be involved in causing PBC. We used these ‘candidate genes’ to help us identify medications that are currently used for treatment of other conditions, which might also be useful for treatment of PBC.

## Highlights

• Trans-ethnic genome-wide meta-analysis (GWMA) of susceptibility to primary biliary cholangitis (PBC).
• Five cohorts of European ancestry and two East Asian cohorts (n = 10,516 cases and 20,772 controls).
• Identification of 21 additional risk loci for PBC.
• Preliminary evidence that the genetic architecture of PBC is broadly shared across European and East Asian populations.
• Identification (using *in silico* drug efficacy screening) of medications potentially suitable for re-purposing to PBC.

## Graphical abstract

**Figure:**
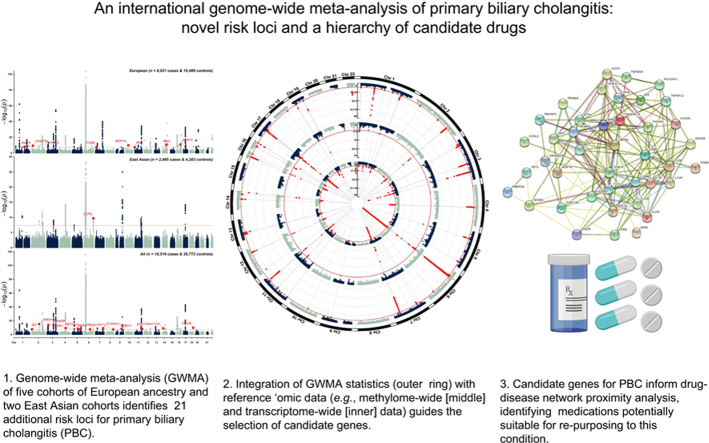

## Introduction

Primary biliary cholangitis (PBC) is a chronic liver disease in which autoimmune injury to the small intrahepatic bile ducts eventually leads to cirrhosis. Only 2 medications, ursodeoxycholic acid (UDCA) and obeticholic acid (OCA), are licensed for the treatment of PBC. Many patients have inadequate response to both agents, leaving them at risk of progressive liver disease. Notwithstanding recent advances, novel therapies are needed for this condition.

Delineating the genetic architecture of PBC can provide insight into its aetiology – and more specifically, identify potential drug targets. Therefore, over the past decade, our respective groups have undertaken genome-wide association studies (GWAS) of PBC in Canadian-US,^1^ Italian,^2^ British,^3^ Japanese,^4^ and Chinese^5^ cohorts; and in 2015, we undertook a genome-wide meta-analysis (GWMA) of the Canadian-US, Italian, and British discovery panels.^6^ These studies have identified genome-wide significant associations at the human leukocyte antigen (HLA) locus and 42 non-HLA loci.

Our GWMA in 2015 did not include the Japanese or Chinese discovery panels. Furthermore, since 2015, our respective groups have undertaken genome-wide genotyping of substantially expanded Canadian, Italian, UK, and US cohorts. Therefore, we present an updated GWMA of PBC that includes these expanded cohorts, as well as the Japanese and Chinese discovery panels. In this study, we aimed to: i) capitalise on the increased sample size to discover additional risk loci for PBC; ii) explore population-specific genetic heterogeneity at known and newly identified risk loci; iii) integrate GWMA statistics with publicly available epigenetic, gene expression, and proteomic datasets to prioritise causal variants and candidate genes; and iv) use these candidate genes for *in silico* drug efficacy screening to identify agents potentially suitable for re-purposing to PBC.

## Materials and methods

*Participants and genotyping* are summarised in Table 1 and detailed in the supplementary information. Written informed consent was obtained from each participant. The research conformed to the ethical guidelines of the 1975 Declaration of Helsinki.

**Table 1:** **Discovery panels included in the current study**.

Panel (Ref)	Cases	Controls	Variants∗	Platform
European panels				
‘Old’ Italian (2)	444	901	13,113,694	Illumina Human610-Quad (Cases), Illumina 1M-Duo (Controls)
WTCCC3 (3)	1,816	5,155	12,881,032	Illumina Human-660 W Quad (Cases), Illumina 1M-Duo (Controls)
‘New’ Canadian-UK	4,615	9,233	8,656,760	Illumina HumanCoreExome
‘New’ Italian	255	579	9,264,788	Illumina HumanCoreExome
‘New’ US	891	621	9,964,354	Illumina Infinium Global Screening Array (GSA) v1
European combined	8,021	16,489	5,186,747	**-**
Asian panels				
Japanese (4)	1,377	1,495	7,308,269	Affymetrix Axiom Genome-Wide ASI 1
Chinese (5)	1,118	2,788	6,934,908	HumanOmniZhongHua-8
Asian combined	2,495	4,283	5,347,815	**-**
All combined	10,516	20,772	2,817,608	**-**

WTCCC3, Wellcome Trust Case-Control Consortium 3.

∗ Number of variants following pre- and post-imputation quality control.

### Quality control

For the European and Japanese panels, quality control (QC) checks were performed at Newcastle University, UK, using the software package PLINK.^7^ Specific QC thresholds to determine outliers were based on visual inspection and varied by panel. For the European panels, we first removed variants with minor allele frequency (MAF) <0.01; genotype call rate <97% (<95% for the ‘old’ Italian, WTCCC3, and ‘new’ US panels); or significant deviation from Hardy Weinberg Equilibrium (HWE) (*p* <10^−6^). We then removed samples with rates of missing data >2% (>4% for the new US panel); whole-genome heterozygosity >3.25 standard deviations from the mean; apparent gender discrepancies (based on X-chromosomal heterozygosity >0.2 for men and <0.2 for women); estimated proportion of identity-by-descent sharing with another sample >0.1 (based on subsets of between 38,000 and 97,000 variants pruned for linkage disequilibrium [LD]); or that did not cluster with the CEU HapMap2 population (based on visual inspection of the first 2 principal components). For the Japanese panel, we used the dataset described in Kawashima *et al.* (2012),^4^ except for the additional removal of 4 cases and 10 controls with apparent gender discrepancies.

All samples recruited in China were processed and analysed on Chinese servers to comply with the Regulation of the People's Republic of China on the Administration of Human Genetic Resources. Thus, for the Chinese panel, QC checks were undertaken on a local server in Shanghai, China. Variants were removed with MAF <0.5%, genotype call rate <95%, or deviation from HWE in controls *p* ≤1x10^−6^. Samples were removed with rates of missing data ≥5% or pairwise identity-by-state, PI_HAT >0.25. Population outliers were identified for exclusion using principal component analysis.

### Genome-wide imputation and post-imputation quality control

For the European and Japanese panels, we used the autosomal variants and samples passing QC to carry out genome-wide imputation within each individual panel using the Michigan Imputation Server with Eagle2 phasing,^8^ informed by the 1000 Genomes Phase 3 reference panel. Following imputation, we discarded variants with imputation R^2^ <0.5; non-unique alleles at the same position; or imputation call rate <90% (based on assigning genotypes according to the most likely genotype call and setting genotypes to missing if the most likely genotype call had posterior probability <0.9). We also used the resulting common set of imputed variants to check for sample duplicates/relationships *across* the European panels (based on estimated identity-by-descent sharing using 25,873 variants pruned for LD) and removed 1 person from each of the 137 identified relative pairs.

For the Chinese panel, genome-wide imputation was undertaken on a local server in Shanghai, China, using SHAPEIT^9^ and IMPUTE2,^10^ and the 1000 Genomes Phase 3 reference panel. Following imputation, we discarded variants with call rates <95% (having set genotypes to missing if the most likely genotype call had posterior probability <0.9), MAF <0.01, or HWE *p* <1×10^-6^ in controls. The resulting imputation summary statistics (log odds ratios [lnORs], standard errors, and *p* values) were submitted without individual-level data to Newcastle, UK, for meta-analysis with the other panels.

### Statistical analysis of European and Japanese cohorts

Within each panel, we performed association analysis of the genome-wide imputed data using logistic regression of disease phenotype on single nucleotide polymorphism (SNP) genotype (coded 0,1,2) in PLINK,^7^ with the first 10 principal components (from a pruned set of SNPs with the HLA region removed) included as covariates to correct for population stratification. (The rationale for removing the HLA region was that inclusion of SNPs in this region would risk generating components that explain variation primarily caused by strong HLA-disease association, rather than population stratification.) For all but the new Canadian-UK panel, the resulting genomic control (GC) inflation factor λ was modest (<1.026); therefore, we carried out GC correction within each panel by multiplying the standard error (SE) of the estimated lnOR for each SNP by √λ. For the new Canadian-UK panel, λ was somewhat inflated at 1.091; therefore, we re-analysed the new Canadian-UK data using a logistic mixed model score test (including the first 10 principal components as covariates) as implemented in the GMMAT package,^11^ resulting in a slightly deflated λ of 0.971. The SE of the estimated lnOR for each SNP from PLINK was then (conservatively) adjusted to match that implied by the GMMAT test statistic. Specifically, we multiplied the PLINK-derived SE for each SNP by a SNP-specific factor *γ*, where *γ* was chosen so that the resulting *χ*^2^ test statistic (lnOR/γSE)^2^ for that SNP had a *p* value equal to the *p* value from GMMAT. GC correction was also performed for the Chinese summary statistics (λ = 1.050) by multiplying the SE of the estimated lnOR for each SNP by √λ.

### Meta-analysis of European, Asian, and combined cohorts

We used the software package META^12^ to perform fixed-effect meta-analysis of the resulting lnORs and adjusted SEs from i) the 5 European panels; ii) the 2 Asian panels; and (3) all 7 panels, in each case restricting the analysis to variants that (following post-imputation QC) appeared within all panels. Within each meta-analysed set (European, Asian, and combined), a further GC correction was performed (to adjust for the inflation factors of 1.041, 1.033, and 1.080, seen within the European, Asian, and combined cohorts, respectively) to produce the final set of genome-wide results. Specifically, as for the individual panels above, the SE of the final lnOR for each SNP was multiplied by √λ, and the test statistic and *p* value were re-calculated accordingly. This use of “double” GC correction might be considered overly conservative, given that part of the observed inflation could be due to polygenicity. We explored this using LD score regression (LDSR)^13^ to compare our original results with those obtained using no GC (or GMMAT-derived) correction at all. We also compared our results from all panels combined with those obtained using trans-ethnic meta-regression analysis as implemented in the software package MR-MEGA^14^ (see supplementary information for details).

### Prioritisation of candidate causal variants and candidate genes

We used the FINEMAP^15^ package and Conditional and Joint Analysis (COJO)^16^ implemented within GCTA^17^ to refine and look for independent associations within genome-wide significant risk loci. We used FINEMAP to construct ‘credible sets’ of variants most likely to be causal in PBC. We used the ENSEMBL Variant Effect Predictor,^18^ FUMA (Functional Mapping and Annotation) GWAS^19^ platform, and reference panels from the Avon Longitudinal Study of Parents and Children (ALSPAC, http://www.bristol.ac.uk/alspac/)^20^ and the INTERVAL study (http://www.donorhealth-btru.nihr.ac.uk/studies/interval-study/)^21^ for mapping and functional annotation of the first set of ‘credible causal variants’ at each risk locus.

Adapting the approach of Barbeira *et al.* (2018),^22^ we used the MetaXcan package; our European GWMA summary statistics; and reference panels from ALSPAC, the Genotype-Tissue Expression (GTEx) project (https://gtexportal.org/),^23^ and the INTERVAL study to derive genome-wide genetic prediction models of DNA methylation, gene expression, and serum protein levels in cases and controls. We used these models to correlate predicted DNA methylation, gene expression, and serum protein levels with disease status in methylome-wide, transcriptome-wide, and serum proteome-wide association studies (MWAS, TWAS, and PWAS, respectively).

We used the moloc package^24^ to look for co-localisation of association signals from our GWMA of the European panels with those derived from mapping of methylation, expression, and protein-quantitative trait loci (mQTLs, eQTLs, pQTLs) in ALSPAC, the GTEx project, and the INTERVAL study, respectively. Finally, we used the DEPICT package^25^ to prioritise the most likely causal gene at risk loci based on gene function.

### Enrichment analysis

We used the STRING Database^26^ to look for enrichment of protein-protein interactions and functional annotations amongst candidate genes; and the DAVID resource^27^ to look for enrichment of KEGG pathways by genes with minimum *p*_GWMA_ <0.01.

### Network-based *in silico* drug efficacy screening

We employed the approach of Guney *et al.* (2016)^28^ in which known drug targets and candidate genes for a disease are used to estimate a drug-disease proximity measure, *z*, that quantifies the closeness (or proximity) of the drug and disease gene networks, respectively, correcting for the known biases of the interactome. For this analysis, we used the drug targets listed in DrugBank (https://www.drugbank.com/, accessed January 2021) and candidate genes for PBC prioritised as above. See the supplementary information for details.

## Results

### GWMA identifies 21 additional genome-wide significant risk loci for PBC

Following QC, the European panels consisted of 5,186,747 variants across 8,021 cases and 16,489 controls; Asian panels, 5,347,815 variants across 2,495 cases and 4,283 controls; and all panels combined, 2,817,608 variants across 10,516 cases and 20,772 controls (Table 1). Of note, there was substantial reduction in the number of variants in all panels combined compared to the European or Asian panels. This resulted from limited overlap of variants that passed post-imputation QC in the European compared to the Asian panels, explained by our use of different genotyping platforms across cohorts, and different LD patterns in Europeans compared to Asians.

GWMA of the European panels identified 46 loci at genome-wide significance (*p* <5×10^−8^); GWMA of the Asian panels, 13 loci at genome-wide significance; and GWMA of all panels combined, 41 loci at genome-wide significance (Fig. S1). Altogether, we identified 56 genome-wide significant risk loci in one or other meta-analysis (Table S1, Fig. S2). Using COJO, we identified an additional risk locus at 19p13.11 with genome-wide significance in conditional analysis of European panels (*p* = 4.66×10^-8^), having narrowly missed this threshold in the main, unconditional analysis (*p* = 6.55×10^-8^) (Fig. S2.57). Thus, a total of 57 genome-wide significant risk loci were identified in the current study. Of these, 21 were not identified in previous studies; and 2, 1q23.1 and 11q24.3, were previously identified at suggestive rather than genome-wide significance (Tables 2A&B).^4^^,^^29^

**Table 2:** **Newly identified or newly confirmed risk loci with replicated evidence of association**.

**Table 2A**									

	**Lead variant in the European panels**			**Lead variant in the Asian panels**			**Lead variant in the combined panels**		
**Locus**	**Variant:A1/A2**	***p* value**	**Beta**	**Variant:A1/A2**	***p* value**	**Beta**	**Variant:A1/A2**	***p* value**	**Beta**
***Gene***	***Chr:BP***	***p***_***perm.***_	***SE***	***Chr:BP***	***p***_***perm.***_	***SE***	***Chr:BP***		***SE***
2p25.1	rs891058:A/G	5.39×10^-7^	-0.12	rs3111414:C/G	1.75×10^-4^	0.17	rs13416555:G/C	2.95×10^-8^	-0.12
*ID2*	*2:8,442,547*	—	*0.02*	*2:8,443,859*	*0.0017*	*0.04*	*2:8,441,735*		*0.02*
2q21.3	rs859767:G/A	1.54×10^-9^	-0.14	rs842349:T/G	1.76×10^-9^	-0.24	rs859767:G/A	8.94×10^-16^	-0.16
*TMEM163*	*2:135,341,200*	—	*0.02*	*2:135,342,452*	*<0.0001*	*0.04*	*2:135,341,200*		*0.02*
6q21	rs58926232:G/C	6.75×10^-7^	0.14	rs4134466:A/G	6.71×10^-7^	0.20	rs742108:A/G	3.16×10^-8^	0.13
*PRDM1*	*6:10,6563,612*	—	*0.03*	*6:106,577,368*	*0.0001*	*0.04*	*6:106,582,920*		*0.02*
6q27	rs3093024:A/G	2.37×10^-6^	0.10	rs4709148:T/C	2.18×10^-10^	-0.25	rs968334:T/C	3.98×10^-10^	0.12
*CCR6*	*6:167,532,793*	*0.0001*	*0.02*	*6:167,521,676*	—	*0.04*	*6:167,526,096*		*0.02*
11q24.3∗	rs10893872:T/C	9.07×10^-6^	0.10	rs11430718:G/GA	1.11×10^-6^	-0.19	rs10893872:T/C	9.77×10^-9^	0.11
*ETS1*	*11:128,325,553*	—	*0.02*	*11:128,307,445*	*<0.0001*	*0.04*	*11:128,325,553*		*0.02*
14q13.2	rs712315:A/T	5.70×10^-7^	0.15	rs199892962:AT/A	4.36×10^-6^	0.20	rs799469:G/A	1.73×10^-9^	0.15
*FAM177A1*	*14:35,409,701*	—	*0.03*	*14:35,646,404*	*0.0020*	*0.04*	*14:35,444,425*		*0.03*
**Table 2B**									
	**Lead variant in the European panels**			**Lead variant in the Asian panels**			**Lead variant in the combined panels**		
**Locus**	**Variant**	***p* value**	**Beta**	**Variant**	***p* value**	**Beta**	**Variant**	***p* value**	**Beta**
***Gene***	***Chr:BP***		***SE***	***Chr:BP***	***p***_***perm.***_	***SE***	***Chr:BP***		***SE***
1q23.1∗	rs945635:G/C	1.59×10^-8^	-0.12	rs60459521:G/C	1.25×10^-3^	-0.46	rs11264790:T/C	2.25×10^-8^	-0.11
*FCRL3*	*1:157,670,290*		*0.02*	*1:157,147,588*	—	*0.14*	*1:157,636,074*		*0.02*
1q32.1	rs55734382:T/C	2.06×10^-9^	-0.14	rs117214467:C/T	8.55×10^-3^	-0.33	rs12122721:A/G	6.95×10^-7^	-0.11
*INAVA*	*1:201,019,059*		*0.02*	*1:200,436,787*	—	*0.13*	*1:200,984,480*		*0.02*
2p23.3	rs34655300:T/C	5.23×10^-10^	0.14	rs893589:A/G	9.41×10^-4^	0.15	rs6711622:A/G	3.89×10^-8^	0.11
*DNMT3A*	*2:25,514,333*		*0.02*	*2:25,259,442*	—	*0.05*	*2:25,531,350*		*0.02*
3p24.2	rs6550965:A/C	3.65×10^-14^	0.16	rs6807549:T/G	1.37×10^-3^	0.17	rs6550965:A/C	1.50×10^-14^	0.15
*RARB*	*3:25,383,587*		*0.02*	*3:24,951,404*	—	*0.05*	*3:25,383,587*		*0.02*
4q24	rs7663401:C/T	2.76×10^-8^	-0.13	rs79109654:T/C	8.56×10^-5^	0.37	rs2007403:T/C	6.19×10^-10^	0.13
*TET2*	*4:106,128,954*		*0.02*	*4:106,170,514*	*0.0040*	*0.09*	*4:106,131,210*		*0.02*
5q21.1	rs141002831:T/TCA	1.47×10^-7^	0.12	rs157181:A/C	3.94×10^-5^	0.21	rs60643069:GA/G	2.48×10^-9^	0.13
*ST8SIA4*	*5:100,202,282*		*0.02*	*5:100,103,288*	*0.0032*	*0.05*	*5:100,238,073*		*0.02*
5q31.3	rs10062349:G/A	7.36×10^-8^	-0.12	rs3761757:A/C	7.48×10^-3^	-0.14	rs6874308:C/T	4.67×10^-8^	-0.11
*NDFIP1*	*5:141,509,597*		*0.02*	*5:141,488,219*	—	*0.05*	*5:141,506,911*		*0.02*
7p21.1	rs7805218:A/G	4.12×10^-8^	0.13	rs77984571:C/G	7.54×10^-3^	-0.14	rs7786537:C/G	1.12×10^-5^	-0.11
*ITGB8*	*7:20,378,801*		*0.02*	*7:20,512,650*	—	*0.05*	*7:20,427,776*		*0.02*
7q34	rs370193557:GAAT/G	1.89×10^-8^	0.12	rs12056141:G/A	1.05×10^-3^	0.18	rs370193557:G/GAAT	9.37×10^-10^	-0.12
*ZC3HAV1*	*7:138,729,543*		*0.02*	*7:138,797,730*	—	*0.05*	*7:138,729,543*		*0.02*
8q24.21	rs4733851:A/G	2.18×10^-7^	0.11	rs1902780:C/T	5.51×10^-4^	-0.13	rs4733851:G/A	4.98×10^-8^	-0.11
*PVT1*	*8:129,264,420*		*0.02*	*8:129,211,788*	—	*0.04*	*8:129,264,420*		*0.02*
9q22.33	rs11390003:GA/G	2.56×10^-8^	-0.15	rs10283737:G/T	1.24×10^-3^	0.15	rs112500293:T/C	7.63×10^-9^	-0.15
*TRIM14*	*9:100,741,912*		*0.03*	*9:100,780,063*	—	*0.05*	*9:100,763,455*		*0.03*
10q11.23	rs7097397:A/G	2.42×10^-10^	-0.14	rs76129863:T/C	4.83×10^-3^	0.56	rs7922169:T/G	5.47×10^-8^	0.11
*WDFY4*	*10:50,025,396*		*0.02*	*10:50,437,561*	—	*0.20*	*10:50,045,456*		*0.02*
11p15.5	rs58523027:TAA/T	4.00×10^-8^	-0.12	rs3216:C/G	8.17×10^-2^	-0.10	rs9667500:G/A	1.74×10^-4^	-0.08
*IRF7*	*11:646,986*		*0.02*	*11:214,421*	—	*0.06*	*11:683,761*		*0.02*
14q32.12	rs72699866:A/G	2.89×10^-11^	-0.20	rs76914265:G/C	1.16×10^-4^	-0.30	rs4904964:C/A	2.45×10^-8^	-0.12
*RIN3*	*14:93,114,787*		*0.03*	*14:93,219,854*	*0.0143*	*0.08*	*14:93,099,867*		*0.02*
16q22.1	rs79577483:G/A	1.23×10^-11^	0.21	rs698729:G/C	1.90×10^-2^	-0.12	rs111644390:TC/T	1.18×10^-9^	0.17
*DPEP3*	*16:68,036,939*		*0.03*	*16:68,624,205*	—	*0.05*	*16:68,046,323*		*0.03*
18q22.2	rs1808094:T/C	2.79×10^-9^	0.13	rs76486918:T/C	2.72×10^-3^	-0.91	rs1808094:T/C	1.66×10^-10^	0.12
*CD226*	*18:67,526,026*		*0.02*	*18:67,081,620*	—	*0.30*	*18:67,526,026*		*0.02*

Results for the lead variant at newly identified or newly confirmed risk loci with *p* <5×10^-8^ in fixed-effect meta-analysis of the European, Asian, or combined panels. (A) Evidence of association was taken to be conclusive because: i) an unequivocal association signal at the same locus was observed in both the European and the Asian panels; and ii) where the lead variant at the locus was different in the European *vs*. the Asian panels, permutation testing confirmed the significance of the signal in the validating dataset at *p*_perm_ <0.00217 (see supplementary information and Table S1). (B) Evidence of association was taken to be strong but not conclusive because unequivocal association was evident in the European but not the Asian panels, or permutation testing was not significant at *p*_perm_ <0.00217. Gene: candidate gene at the risk locus (which is not necessarily the mapped gene). A1, tested allele; A2, alternative allele; BP, base pair position; Chr, chromosome; *p*_perm_, permutation *p* value; OR, odds ratio.

∗ Note that 1q23.1 and 11q24.3 were previously identified at suggestive level of significance in the study by Kawashima *et al*. (2017).

At 6 newly identified or newly confirmed risk loci, we considered evidence of association to be conclusive because: i) an unequivocal association signal was evident in both the European and Asian panels; and ii) where the lead variant at the locus was different in the European compared to the Asian panels, permutation testing confirmed the significance of a signal in the validating dataset, located in proximity to the primary signal in the index dataset (*p*_permutation_ <0.00217, corresponding to *p* <0.05 Bonferroni-corrected for 23 tests; see supplementary information for details) (Table 2A, Table S1, Fig. S2).

At 17 newly identified or newly confirmed risk loci, we considered evidence of association to be strong but not conclusive because unequivocal association was evident in the European but not the Asian panels, or permutation testing was not significant at *p*_permutation_ <0.00217 (Table 2B, Table S1, Fig. S2). We note, however, that most of these loci achieved levels of significance in the Asian panels that were suggestive for validation, including 2 loci with suggestive permutation *p* values (4q24, *p*_permutation_ = 0.0040; and 5q21.1, *p*_permutation_ = 0.0032).

We confirmed genome-wide significant associations at 34 of 43 previously identified risk loci for PBC – but not at 9 previously identified risk loci. Seven of these 9 loci nevertheless showed a convincing association signal, albeit at *p* >5×10^-8^ (Table S2, Fig. S3). We found no evidence of association at the 15q25.1 locus (harbouring *IL16*) that was discovered and validated in the Chinese GWAS by Qiu *et al.* (2017) ^5^; this is explained by the absence of a signal in the Japanese and European panels. Coverage of the 19p13.2 locus was too sparse to test association.

Using FINEMAP and COJO, we found that at most risk loci, the association signal was best explained by a single variant – but at 16 loci, it was best explained by ≥2 independent variants (Table S3). Notable examples include the 2q32.2 locus harbouring *STAT4*, with 3 independent variants; 3q25.33 (*IL12A*, 3 variants); 7q32.1 (*IRF5*, 2 variants); and 16p13.13 (*CLEC16A*, 2 variants) – all consistent with previous studies showing ≥2 independent associations at each of these loci.

We compared our original results to those obtained without GC (or GMMAT-derived) correction. As expected, without correction, all loci previously identified as genome-wide significant reached slightly higher levels of significance, while a few loci that did not reach genome-wide significance in our original analysis, now (just) did so (Fig. S4 and Table S4). We also compared our original results for all panels combined with those obtained using trans-ethnic meta-regression analysis, implemented in MR-MEGA. Results from MR-MEGA were highly concordant with those from our original analysis (Fig. S5), also providing genome-wide significant confirmation of an independent association signal at 7q32.1, which exhibited significant heterogeneity in the direction of effects between the Asian and European cohorts (Table S5 and Fig. S6).

### PBC shows genetic correlation with other autoimmune conditions

Recognising that most risk loci for PBC are also risk loci for other autoimmune conditions (Table S6), we used LDSR implemented via LD Hub^30^ to evaluate the genetic correlation between PBC (using summary statistics from our European panels) and complex traits with GWAS summary statistics in the LD Hub database. We found significant genetic correlation between PBC and other immune-mediated inflammatory disorders, including systemic lupus erythematosus (SLE, rg = 0.54, *p* = 2.87×10^-14^), rheumatoid arthritis (RA, rg = 0.26, *p* = 3.77×10^-5^), and inflammatory bowel disease (IBD, rg = 0.23, *p* = 6.97×10^-5^) (Table S7). We were unable to test genetic correlation of PBC with autoimmune thyroid disease, Sjögren syndrome, or systemic sclerosis because GWAS summary statistics for these conditions were not available in LD Hub at the time of interrogation (19.09.2019).

### The genetic architecture of PBC is broadly shared across European and Asian populations

To evaluate consistency between European and Asian signals, we applied permutation testing where warranted and standard meta-analysis measures of heterogeneity to the lead variants at each of the 56 genome-wide significant risk loci identified or confirmed in the main, unconditional analyses (Table S1). We found concordance between risk loci operating in European and Asian populations, considering i) the much smaller sample size of the Asian panels; and ii) the interrogation of different variants in the European compared to the Asian panels, for reasons given above (for a detailed commentary of each risk locus, see Fig. S2). With few exceptions, we also found concordance between the lnORs seen in the combined Asian and combined European panels (Fig. S7).

To investigate overall concordance in the genetic basis of PBC between European and Asian populations, we estimated the proportion of trait variance explained (on the liability scale) in the Japanese cohort (for which individual-level genotype data were available) by sets of variants chosen according to their *p* values in the European GWMA (see supplementary information). Regardless of the *p* value threshold and the assumed trait prevalence, variants showing some level of association in the European GWMA explained more of the trait variance than an equivalent number of randomly chosen variants – in most instances, significantly more – supporting the conclusion that loci influencing the risk of PBC in Europeans, also influence its risk in Asians (Table S8).

Thus, while equivalently powered cohorts, accurately genotyped at the same set of variants, would be required to fully address the question of population-specific genetic heterogeneity, our results provide preliminary evidence that the genetic architecture of PBC is broadly shared across European and Asian populations.

### Co-localisation and DEPICT enable prioritisation of candidate genes

In functional annotation, we found that credible causal variants included missense variants in 21 genes at 14 risk loci; splice variants in 8 genes at 5 risk loci; and stop variants in 2 genes at 2 risk loci (Table S9). Few of these variants were predicted to be deleterious. Credible causal variants at all genome-wide significant risk loci mapped to chromatin interacting regions (CIRs), mQTLs, eQTLs, or pQTLs (Tables S10–12); and in the MWAS, TWAS, and PWAS, we predicted differential methylation, transcription, or translation of genes at and beyond GWMA-significant loci (Tables S13–15, Fig. S8). These observations suggest that the genetic architecture of PBC confers susceptibility to disease mainly by influencing the regulation of expression of causal genes. Therefore, we sought co-localisation of GWMA with mQTL, eQTL, or pQTL association signals, aiming to pinpoint causal variants and genes across the genome. Using moloc, we identified 251 co-localisation models with posterior probability of association ≥0.80, implicating variants and genes at 60 loci (Table S16, Fig. S8C). Of these, 28 correspond to GWMA-significant risk loci, where co-localisation models implicate candidate genes such as *IL12RB2* (1p31.3), *FCRL3* (1q23.1), and *INAVA* (1q32.1). Association at the other 32 loci did not reach genome-wide significance in the GWMA; co-localisation models nevertheless implicate highly plausible candidate genes at some of these loci, such as *CCL21* (9p13.3) and *IL2RB* (22q12.3).

We found that candidate genes implicated by co-localisation were broadly concordant with those implicated by functional annotation of credible causal variants, and by the MWAS, TWAS, and PWAS. As in previous studies, we also observed that candidate genes at disparate risk loci are evidently related in function, *e.g.*, *IL12A* (3q25.33), *IL12B* (5q33.3), *IL12RB1* (19p13.11), and *IL12RB2* (1p31.3). Therefore, we used DEPICT^25^ to prioritise candidate genes at genome-wide significant risk loci based on gene function. In this way, we identified 82 candidate genes with a false discovery rate (FDR) <5% across 48 loci (Table S17). As expected, genes prioritised by DEPICT overlapped with those prioritised by the other approaches (Table S18).

We used the information garnered above to finalise a list of top candidate genes at genome-wide significant risk loci (Table S18). Using STRING,^26^ we found these genes to be highly enriched for protein-protein interactions (*p* <1.0×10^-16^), with enrichment at FDR <5% of the following KEGG pathways: T helper (T_H_)1 and T_H_2 cell differentiation, T_H_17 cell differentiation, and toll-like receptor (TLR), RIG-I-like receptor (RLR), TNF, NF-κB, and JAK-STAT signalling pathways, amongst others (Fig. S9). For comparison, we undertook enrichment analysis using DAVID^27^ of 1,388 genes with minimum *p*_GWMA_ <0.01, which identified enrichment at FDR <5% of the following KEGG pathways: antigen processing and presentation, FcγR-mediated phagocytosis, NK cell-mediated cytotoxicity, and T cell receptor, B cell receptor, PI3K-AKT, FcεRI, JAK-STAT, NF-κB, and MAPK signalling pathways, amongst others (Table S19).

### *In silico* drug efficacy screening identifies agents potentially suitable for re-purposing to PBC

In the approach of Guney *et al.* (2016),^28^ the more negative the value of *z*, the closer the drug and disease gene networks. A cut-off of *z* ≤-0.15 is taken to show that the drug is proximal to the disease and thus, might exert pharmacological effects on it. In our analysis, we identified many agents with *z* ≤-0.15, which are therefore predicted to exert pharmacological effects on PBC (Table 3, Table S20). Top-ranking drugs that might be predicted to ameliorate PBC included several immunomodulators, such as ustekinumab, an anti-IL-12/23 monoclonal antibody used for psoriasis and Crohn’s disease (*z* = -4.757); belatacept, a CTLA-4 fusion protein used in organ transplantation (*z* = -4.709); and abatacept, a CTLA-4 fusion protein used for RA, juvenile idiopathic arthritis (JIA), and psoriatic arthritis (*z* = -4.603). Of interest, other top-ranking agents include the retinoids etretinate and its metabolite acitretin, both of which are used for the treatment of psoriasis (*z* = -3.879 and *z* = -4.548, respectively). Top-ranking drugs that might be predicted to exacerbate PBC included the pharmacological interferons, such as interferon alfa-2a and interferon beta-1b (*z* = -2.748 and *z* = -2.688, respectively). Amongst recognised treatments for PBC, fenofibrate scored *z* = -0.986; bezafibrate, *z* = -0.866; and OCA, *z* = -0.737, respectively. Thus, these drugs might be predicted to exert pharmacological effects on PBC. Conversely, UDCA scored *z* = +0.171, meaning it is not predicted to treat the genetically determined component of disease in PBC.

**Table 3:** ***In silico* drug efficacy screening**.

Drug name	*z*	*p* value	Description
Ustekinumab	-4.757	9.82×10^-^^7^	Anti-IL-12/23 p40 antibody
Belatacept	-4.709	1.24×10^-^^6^	IgG1 Fc/CTLA-4 fusion protein
Abatacept	-4.603	2.08×10^-^^6^	IgG1 Fc/CTLA-4 fusion protein
Acitretin	-4.548	2.71×10^-^^6^	Oral retinoid
Denosumab	-4.416	5.03×10^-^^6^	Anti-TNFSF11 antibody
Etretinate	-3.879	5.24×10^-^^5^	Oral retinoid
Tofacitinib	-3.340	4.19×10^-^^4^	Janus kinase inhibitor
Basiliximab	-3.320	4.50×10^-^^4^	Anti-IL2Rα antibody
Gilteritinib	-3.310	4.66×10^-^^4^	Tyrosine kinase inhibitor
Fostamatinib	-3.305	4.75×10^-^^4^	Tyrosine kinase inhibitor
Imatinib	-3.189	7.14×10^-^^4^	Tyrosine kinase inhibitor
Dexchlorpheniramine maleate	-3.182	7.31×10^-^^4^	Antihistamine
Linagliptin	-3.010	1.31×10^-^^3^	Dipeptidyl Peptidase-IV Inhibitor
Brigatinib	-2.961	1.53×10^-^^3^	ALK and EGFR inhibitor
Interferon alfa-2a	-2.748	3.00×10^-^^3^	Alpha interferon
Interferon beta-1b	-2.688	3.59×10^-^^3^	Beta interferon
Metformin	-1.894	0.029	Biguanide antidiabetic agent
Fenofibrate	-0.986	0.162	Fibrate, PPAR-α agonist
Bezafibrate	-0.866	0.193	Fibrate, PPAR-α/δ/γ agonist
Obeticholic acid	-0.737	0.231	Bile acid, FXR agonist
Rifampicin	-0.627	0.265	Antibiotic
Ursodeoxycholic acid	+0.171	0.568	Bile acid

Results for top-ranking agents and current treatments for primary biliary cholangitis, *z* being a drug-disease proximity measure, defined as *z* = (d_c_-μ)/σ where d_c_ is the average shortest path length between the drug's targets and the nearest disease gene, and μ and σ are calculated via a randomisation procedure as described in the supplementary information. Guney *et al.* define a drug to be proximal to a disease if its proximity follows *z* ≤−0.15 (*p* ≤0.44), and distant otherwise.

## Discussion

We report the largest GWMA of PBC undertaken to date, with a sample size four times greater than that of our previous study. In this better-powered study, we identified 21 additional genome-wide significant risk loci; showed that the genetic architecture of PBC is broadly shared across European and Asian populations; prioritised candidate genes at known and newly identified genome-wide significant risk loci; and used these candidate genes to identify medications predicted to treat the genetically determined component of disease in PBC, which might therefore be suitable for re-purposing to this condition.

**Fig. 1:**
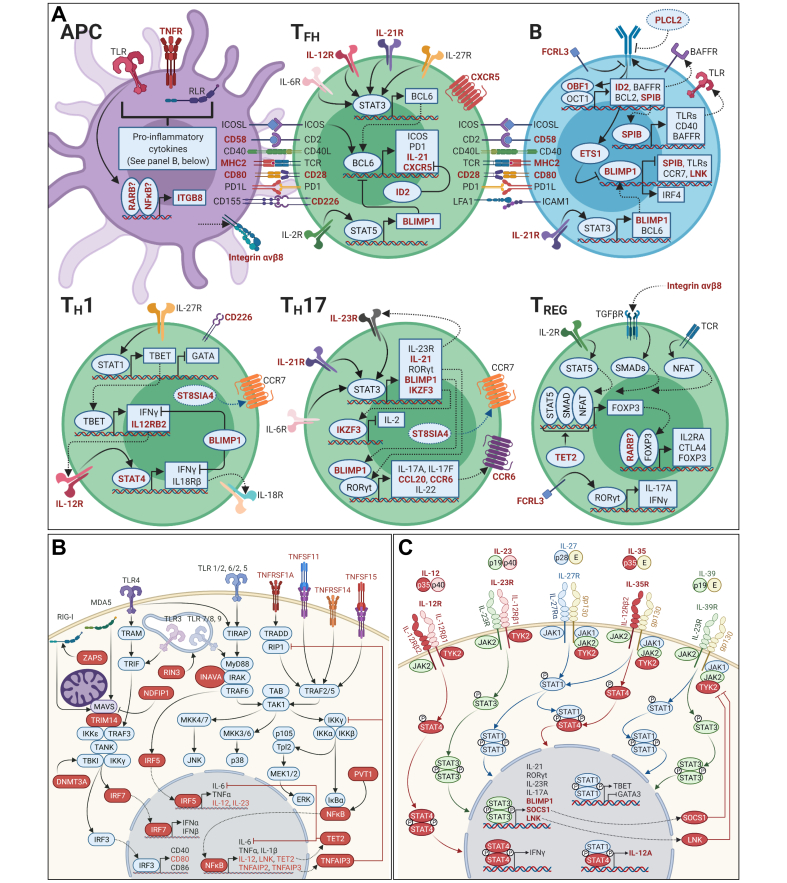
Biological processes implicated by candidate genes  prioritised in the current study. (A) T and B cell activation, and differentiation of T follicular helper, T_H_1, T_H_17, and T_REG_ cells; (B) pattern recognition receptor and TNF signalling in antigen presenting cells; and (C) signalling by the IL-12 family of cytokines. T_H_, T helper; T_REG,_ regulatory T. (Figure created with BioRender.com).

Candidate genes at newly identified or newly confirmed risk loci provide additional insights into the pathogenesis of PBC (Fig. 1). Thus, *INAVA* (1q32.1) amplifies pattern recognition receptor (PRR) signalling; *DNMT3A* (2p23.3), *ZC3HAV1* (7q34), and *TRIM14* (9q22.33) are each involved in RLR signalling; *TET2* (4q24) represses transcription of IL-6; and *PVT1* (8q24.21) regulates inflammation via NF-κB and MAPK pathways. Chemokine receptor 6 (*CCR6*, 6q27) interacts with CCL20 in the chemotaxis of dendritic cells and lymphocytes to inflamed epithelia; *ST8SIA4* (5q21.1) is required for the interaction of CCR7 with CCL21 in the trafficking of immune cells to secondary lymphatic organs; and *CD226* (18q22.2) participates in lymphocyte and NK cell adhesion and signalling. Fc receptor-like protein 3 (*FCRL3*, 1q23.1), *ID2* (2p25.1), *TET2* (4q24), *RARB* (3p24.2), *NDFIP1* (5q31.3), *ITGB8* (7p21.1), and *CD226* (18q22.2) are each involved in the differentiation of T_H_1, T_H_17, or regulatory T cells. As expected, enrichment analysis of candidate genes reiterated the importance of PRR, TNF, and NF-κB signalling, and T_H_1/T_H_17 cell differentiation in this disease. These findings are consistent with functional data emphasising the importance of innate immune cell hypersensitivity, chemokine signalling and immune cell trafficking, and T_H_1/T_H_17 cell polarisation in PBC pathogenesis, as summarised by Gulamhusein and Hirschfield (2020)^31^ in their recent review.

There is considerable current interest in the ‘Druggable Genome’, *i.e.*, the use of genome-wide approaches to find targets for drug discovery (for example, see the Open Targets initiative at https://www.opentargets.org/). In the current study, having prioritised candidate genes, we used network-based *in silico* drug efficacy screening to identify agents potentially suitable for re-purposing to PBC. Given our other findings – including genetic correlation of PBC with SLE, RA, and IBD – it is expected that the top-ranking medications should include immunomodulators already approved for the treatment of RA, JIA, IBD, MS, or psoriasis.

The evidence to support re-purposing of these immunomodulators to PBC is circumstantial yet convincing – but circumspection is required. For example, in the current study, LDSR demonstrated genetic correlation with IBD; enrichment analysis showed association with ‘T_H_1 and T_H_2 cell differentiation’; and drug efficacy screening suggested that ustekinumab, an anti-IL-12/23 monoclonal antibody used for treatment of Crohn’s disease, might exert pharmacological effects on PBC. Therefore, it is notable that ustekinumab showed minimal effect on PBC in the trial by Hirschfield *et al.* (2016).^32^ Similarly, drug efficacy screening suggested that abatacept, a CTLA-4 fusion protein used for treatment of RA, might be effective for treatment of PBC – but abatacept showed no effect on PBC in the trial by Bowlus *et al.* (2019).^33^ A potential explanation for these discrepant observations, also expounded by Bowlus *et al.*,^33^ is that the evaluation of immunomodulators in PBC might require a change in clinical trial design. Thus, immunomodulators might require immunological rather than cholestatic endpoints; might be more effective in early disease, before the cholestatic liver injury predominates; and might require combined treatment of both the autoimmune and cholestatic injuries. Re-design of clinical trials in PBC might be contentious but the use of genomic data to prioritise potential agents for PBC is not, as new treatments for PBC are needed and the druggable genome provides a framework to find them.

It is notable that in drug efficacy screening, UDCA – well-established as first-line treatment for PBC – was not predicted to be therapeutic in this condition. One possibility is that UDCA serves primarily to treat a cholestatic liver injury that is critical to disease progression but orthogonal to the genetically determined, autoimmune processes that confer risk of disease. Conversely, OCA (a potent FXR agonist) and the fibrates, bezafibrate and fenofibrate (PPAR-α/δ/γ and PPAR-α agonists, respectively), are expected to have immune-modulatory as well as anti-cholestatic effects.^34^^,^^35^

We acknowledge 2 major limitations of the study. First, the absence of an independent validation cohort meant we were unable to confirm several newly identified risk loci. Other strategies, such as cross-phenotype meta-analysis, may be required for external validation of these loci. Second, the use of different genotyping platforms across cohorts meant that at many risk loci, the lead variant in the European panels was not represented in the Asian panels, or *vice versa*. This, together with marked disparity in the sample size of the European *vs*. the Asian panels, meant that we were unable to fully address the question of population-specific genetic heterogeneity.

In conclusion, our large, trans-ethnic GWMA of PBC has identified additional risk loci; found little evidence for population-specific genetic heterogeneity; and, through functional annotation of credible causal variants and multi-omic analysis, allowed us to prioritise candidate genes, and thereby prioritise drugs potentially suitable for re-purposing to PBC. This study emphasises the value of genomic approaches to provide biological insight and guide the development of novel therapies.

### Abbreviations

ALSPAC, Avon Longitudinal Study of Parents and Children; COJO, Conditional and Joint Analysis; eQTL, expression quantitative trait locus; FDR, false discovery rate; FUMA, functional mapping and annotation; GC, genomic control; GTEx, genotype-tissue expression; GWAS, genome-wide association study; GWMA, genome-wide meta-analysis; HLA, human leukocyte antigen; HWE, Hardy-Weinburg equilibrium; IBD, inflammatory bowel disease; JIA, juvenile inflammatory arthritis; LD, linkage disequilibrium; LDSR, linkage disequilibrium score regression; MAF, minor allele frequency; mQTL, methylation quantitative trait locus; MS, multiple sclerosis; MWAS, methylome-wide association study; OCA, obeticholic acid; OR, odds ratio; PBC, primary biliary cholangitis; pQTL, protein-quantitative trait locus; PWAS, proteome-wide association study; QC, quality control; RA, rheumatoid arthritis; SLE, systemic lupus erythematosus; SNP, single nucleotide polymorphism; T_H_, T helper; TWAS, transcriptome-wide association study; UDCA, ursodeoxycholic acid.

## Financial support

HJC is funded by a Wellcome Trust Senior Research Fellowship in Basic Biomedical Science (102858/Z/13/Z). JJF is funded by a BBSRC DTP studentship (BB/M011186/1). The University of Cambridge has received salary support in respect of RNS from the NHS in the East of England through the Clinical Academic Reserve. KNL was supported by the 10.13039/100000002NIH, DK80670. CIA is a Cancer Prevention Research Institute of Texas (CPRIT) Established Scholar and is supported by RR170048 and CA186566. AG, MC, and PI are supported by unrestricted research funding from AMAF Monza ONLUS and AIRCS, and partially supported by the 10.13039/501100003407Italian Ministry of University and Research (MIUR) - Department of Excellence project PREMIA (PREcision MedIcine Approach: bringing biomarker research to clinic). KAS is supported by the Sherman Family Chair in Genomic Medicine and a Foundation grant from the 10.13039/501100000024Canadian Institutes for Health Research (353710) and an 10.13039/100012171Ontario Research Fund award (RE-09090). MN is funded by a Grant-in-Aid for Clinical Research from the National Hospital Organization and grants from 10.13039/501100001691Japan Society for the Promotion of Science (26293181, 17H04169). KT and MN are funded by grants from 10.13039/100009619Japan Agency for Medical Research and Development (AMED) (JP20km0405205 and JP20km0405501). GFM was funded by a post-doctoral fellowship from the 10.13039/501100000272National Institute for Health Research (NIHR) Rare Diseases – Translational Research Collaboration (RD-TRC) and is now funded by a Clinician Academic Research Partnership (CARP) award from the 10.13039/501100000265Medical Research Council (MRC), UK. The 10.13039/501100000265MRC, UK (Grant ref: 217065/Z/19/Z) and the 10.13039/501100000883University of Bristol provide core support for ALSPAC, and the ALSPAC GWAS data were generated by Sample Logistics and Genotyping Facilities at Wellcome Sanger Institute and LabCorp (Laboratory Corporation of America) using support from 23andMe. A comprehensive list of grants funding is available on the ALSPAC website (http://www.bristol.ac.uk/alspac/external/documents/grant-acknowledgements.pdf). The research included in the current study involved collection of genotype data from the ARIES mothers funded by the 10.13039/100004440Wellcome Trust (WT088806), and collection of the ARIES methylation data funded by the 10.13039/501100000268BBSRC (BBI025751/1 and BB/I025263/1). UK-PBC was funded by a Stratified Medicine award from the 10.13039/501100000265MRC, UK (MR/L001489/1).

## Authors’ contributions

HJC, DEJ, RNS, KNL, PI, MFS, KAS, CIA, XM, MN and GFM conceived and planned the project. KNL, PI, MFS, KAS, XM, MN and GFM directed recruitment, sample collection and genotyping. HJC, JJF, KU, RD and CIA carried out the analyses. All authors contributed to interpretation of the results. HJC and GFM wrote the manuscript. All authors provided critical feedback and shaped the analysis and manuscript.

## Data availability statement

Following publication, summary statistics from the current study will be deposited with the European Genome-phenome Archive.

## Conflicts of interest

GMH has consulted and/or been a speaker for Intercept, Genfit, Cymabay, GSK, and Falk. RNS and GFM have each received research funding from Intercept Pharmaceuticals. HJC, JJF, KU, RD, YA, YH, MK, NN, S-SK, OG, YK, MN, KT, RT, YS, ZL, BDJ, EJA, AG, MC, RA, AC, MdA, AB, JH, MARF, DS, DEJ, SF, AS, VLM, KNL, CIA, MFS, PI, KAS, XM and MN report no conflicts of interest.

Please refer to the accompanying ICMJE disclosure forms for further details.
