# Corrigendum to ‘An international genome-wide meta-analysis of primary biliary cholangitis: Novel risk loci and candidate drugs’ [J Hepatol 2021;75(3):572–581]

**DOI:** 10.1016/j.jhep.2021.11.015

**Published:** 2022-02

**Authors:** Heather J. Cordell, James J. Fryett, Kazuko Ueno, Rebecca Darlay, Yoshihiro Aiba, Yuki Hitomi, Minae Kawashima, Nao Nishida, Seik-Soon Khor, Olivier Gervais, Yosuke Kawai, Masao Nagasaki, Katsushi Tokunaga, Ruqi Tang, Yongyong Shi, Zhiqiang Li, Brian D. Juran, Elizabeth J. Atkinson, Alessio Gerussi, Marco Carbone, Rosanna Asselta, Angela Cheung, Mariza de Andrade, Aris Baras, Julie Horowitz, Manuel A.R. Ferreira, Dylan Sun, David E. Jones, Steven Flack, Ann Spicer, Victoria L. Mulcahy, Jinyoung Byun, Younghun Han, Richard N. Sandford, Konstantinos N. Lazaridis, Christopher I. Amos, Gideon M. Hirschfield, Michael F. Seldin, Pietro Invernizzi, Katherine A. Siminovitch, Xiong Ma, Minoru Nakamura, George F. Mells, Canadian PBC Consortium, Katherine A. Siminovitch, Gideon M. Hirschfield, Andrew Mason, Catherine Vincent, Gang Xie, Jinyi Zhang, Chinese PBC Consortium, Ruqi Tang, Xiong Ma, Zhiqiang Li, Yongyong Shi, Italian PBC Study Group, Andrea Affronti, Piero L. Almasio, Domenico Alvaro, Pietro Andreone, Angelo Andriulli, Francesco Azzaroli, Pier Maria Battezzati, Antonio Benedetti, Maria Consiglia Bragazzi, Maurizia Brunetto, Savino Bruno, Vincenza Calvaruso, Vincenzo Cardinale, Giovanni Casella, Nora Cazzagon, Antonio Ciaccio, Barbara Coco, Agostino Colli, Guido Colloredo, Massimo Colombo, Silvia Colombo, Laura Cristoferi, Carmela Cursaro, Lory Saveria Crocè, Andrea Crosignani, Daphne D’Amato, Francesca Donato, Gianfranco Elia, Luca Fabris, Stefano Fagiuoli, Carlo Ferrari, Annarosa Floreani, Andrea Galli, Edoardo Giannini, Ignazio Grattagliano, Pietro Lampertico, Ana Lleo, Federica Malinverno, Clara Mancuso, Fabio Marra, Marco Marzioni, Sara Massironi, Alberto Mattalia, Luca Miele, Chiara Milani, Lorenzo Morini, Filomena Morisco, Luigi Muratori, Paolo Muratori, Grazia A. Niro, Sarah O’Donnell, Antonio Picciotto, Piero Portincasa, Cristina Rigamonti, Vincenzo Ronca, Floriano Rosina, Giancarlo Spinzi, Mario Strazzabosco, Mirko Tarocchi, Claudio Tiribelli, Pierluigi Toniutto, Luca Valenti, Maria Vinci, Massimo Zuin, Japan-PBC-GWAS Consortium, Hitomi Nakamura, Seigo Abiru, Shinya Nagaoka, Atsumasa Komori, Hiroshi Yatsuhashi, Hiromi Ishibashi, Masahiro Ito, Kiyoshi Migita, Hiromasa Ohira, Shinji Katsushima, Atsushi Naganuma, Kazuhiro Sugi, Tatsuji Komatsu, Tomohiko Mannami, Kouki Matsushita, Kaname Yoshizawa, Fujio Makita, Toshiki Nikami, Hideo Nishimura, Hiroshi Kouno, Hirotaka Kouno, Hajime Ota, Takuya Komura, Yoko Nakamura, Masaaki Shimada, Noboru Hirashima, Toshiki Komeda, Keisuke Ario, Makoto Nakamuta, Tsutomu Yamashita, Kiyoshi Furuta, Masahiro Kikuchi, Noriaki Naeshiro, Hironao Takahashi, Yutaka Mano, Seiji Tsunematsu, Iwao Yabuuchi, Yusuke Shimada, Kazuhiko Yamauchi, Rie Sugimoto, Hironori Sakai, Eiji Mita, Masaharu Koda, Satoru Tsuruta, Hiroshi Kamitsukasa, Takeaki Sato, Naohiko Masaki, Tatsuro Kobata, Nobuyoshi Fukushima, Yukio Ohara, Toyokichi Muro, Eiichi Takesaki, Hitoshi Takaki, Tetsuo Yamamoto, Michio Kato, Yuko Nagaoki, Shigeki Hayashi, Jinya Ishida, Yukio Watanabe, Masakazu Kobayashi, Michiaki Koga, Takeo Saoshiro, Michiyasu Yagura, Keisuke Hirata, Atsushu Tanaka, Hajime Takikawa, Mikio Zeniya, Masanori Abe, Morikazu Onji, Shuichi Kaneko, Masao Honda, Kuniaki Arai, Teruko Arinaga-Hino, Etsuko Hashimoto, Makiko Taniai, Takeji Umemura, Satoru Joshita, Kazuhiko Nakao, Tatsuki Ichikawa, Hidetaka Shibata, Satoshi Yamagiwa, Masataka Seike, Koichi Honda, Shotaro Sakisaka, Yasuaki Takeyama, Masaru Harada, Michio Senju, Osamu Yokosuka, Tatsuo Kanda, Yoshiyuki Ueno, Kentaro Kikuchi, Hirotoshi Ebinuma, Takashi Himoto, Michio Yasunami, Kazumoto Murata, Masashi Mizokami, Kazuhito Kawata, Shinji Shimoda, Yasuhiro Miyake, Akinobu Takaki, Kazuhide Yamamoto, Katsuji Hirano, Takafumi Ichida, Akio Ido, Hirohito Tsubouchi, Kazuaki Chayama, Kenichi Harada, Yasuni Nakanuma, Yoshihiko Maehara, Akinobu Taketomi, Ken Shirabe, Yuji Soejima, Akira Mori, Shintaro Yagi, Shinji Uemoto, Egawa H, Tomohiro Tanaka, Noriyo Yamashiki, Sumito Tamura, Yasuhiro Sugawara, Norihiro Kokudo, US PBC Consortium, Brian D. Juran, Elizabeth J. Atkinson, Angela Cheung, Mariza de Andrade, Konstantinos N. Lazaridis, Naga Chalasani, Vel Luketic, Joseph Odin, Kapil Chopra, Aris Baras, Julie Horowitz, Goncalo Abecasis, Michael Cantor, Giovanni Coppola, Aris Economides, Luca A. Lotta, John D. Overton, Jeffrey G. Reid, Alan Shuldiner, Christina Beechert, Caitlin Forsythe, Erin D. Fuller, Zhenhua Gu, Michael Lattari, Alexander Lopez, John D. Overton, Thomas D. Schleicher, Maria Sotiropoulos Padilla, Karina Toledo, Louis Widom, Sarah E. Wolf, Manasi Pradhan, Kia Manoochehri, Ricardo H. Ulloa, Xiaodong Bai, Suganthi Balasubramanian, Leland Barnard, Andrew Blumenfeld, Gisu Eom, Lukas Habegger, Alicia Hawes, Shareef Khalid, Jeffrey G. Reid, Evan K. Maxwell, William Salerno, Jeffrey C. Staples, Marcus B. Jones, Lyndon J. Mitnaul, UK-PBC Consortium, Richard Sturgess, Christopher Healey, Andrew Yeoman, Anton V.J. Gunasekera, Paul Kooner, Kapil Kapur, V. Sathyanarayana, Yiannis Kallis, Javaid Subhani, Rory Harvey, Roger McCorry, Paul Rooney, David Ramanaden, Richard Evans, Thiriloganathan Mathialahan, Jaber Gasem, Christopher Shorrock, Mahesh Bhalme, Paul Southern, Jeremy A. Tibble, David A. Gorard, Susan Jones, George Mells, Victoria Mulcahy, Brijesh Srivastava, Matthew R. Foxton, Carole E. Collins, David Elphick, Mazn Karmo, Francisco Porras-Perez, Michael Mendall, Tom Yapp, Minesh Patel, Roland Ede, Joanne Sayer, James Jupp, Neil Fisher, Martyn J. Carter, Konrad Koss, Jayshri Shah, Andrzej Piotrowicz, Glyn Scott, Charles Grimley, Ian R. Gooding, Simon Williams, Judith Tidbury, Guan Lim, Kuldeep Cheent, Sass Levi, Dina Mansour, Matilda Beckley, Coral Hollywood, Terry Wong, Richard Marley, John Ramage, Harriet M. Gordon, Jo Ridpath, Theodore Ngatchu, Vijay Paul Bob Grover, Ray G. Shidrawi, George Abouda, L. Corless, Mark Narain, Ian Rees, Ashley Brown, Simon Taylor-Robinson, Joy Wilkins, Leonie Grellier, Paul Banim, Debasish Das, Michael A. Heneghan, Howard Curtis, Helen C. Matthews, Faiyaz Mohammed, Mark Aldersley, Raj Srirajaskanthan, Giles Walker, Alistair McNair, Amar Sharif, Sambit Sen, George Bird, Martin I. Prince, Geeta Prasad, Paul Kitchen, Adrian Barnardo, Chirag Oza, Nurani N. Sivaramakrishnan, Prakash Gupta, Amir Shah, Chris D.J. Evans, Subrata Saha, Katharine Pollock, Peter Bramley, Ashis Mukhopadhya, Stephen T. Barclay, Natasha McDonald, Andrew J. Bathgate, Kelvin Palmer, John F. Dillon, Simon M. Rushbrook, Robert Przemioslo, Chris McDonald, Andrew Millar, Cheh Tai, Stephen Mitchell, Jane Metcalf, Syed Shaukat, Mary Ninkovic, Udi Shmueli, Andrew Davis, Asifabbas Naqvi, Tom J.W. Lee, Stephen Ryder, Jane Collier, Howard Klass, Matthew E. Cramp, Nichols Sharer, Richard Aspinall, Deb Ghosh, Andrew C. Douds, Jonathan Booth, Earl Williams, Hyder Hussaini, John Christie, Steven Mann, Douglas Thorburn, Aileen Marshall, Imran Patanwala, Aftab Ala, Julia Maltby, Ray Matthew, Chris Corbett, Sam Vyas, Saket Singhal, Dermot Gleeson, Sharat Misra, Jeff Butterworth, Keith George, Tim Harding, Andrew Douglass, Harriet Mitchison, Simon Panter, Jeremy Shearman, Gary Bray, Michael Roberts, Graham Butcher, Daniel Forton, Zahid Mahmood, Matthew Cowan, Debashis Das, Chin Lye Ch'ng, Mesbah Rahman, Gregory C.A. Whatley, Emma Wesley, Aditya Mandal, Sanjiv Jain, Stephen P. Pereira, Mark Wright, Palak Trivedi, Fiona H. Gordon, Esther Unitt, Altaf Palejwala, Andrew Austin, Vishwaraj Vemala, Allister Grant, Andrew D. Higham, Alison Brind, Ray Mathew, Mark Cox, Subramaniam Ramakrishnan, Alistair King, Simon Whalley, Jocelyn Fraser, S.J. Thomson, Andrew Bell, Voi Shim Wong, Richard Kia, Ian Gee, Richard Keld, Rupert Ransford, James Gotto, Charles Millson

**Affiliations:** 25 Departments of Medicine, Immunology and Medical Sciences, University of Toronto, Canada; 26 Mount Sinai Hospital, Lunenfeld-Tanenbaum Research Institute, Canada; 27 Toronto General Research Institute, Toronto, Ontario, Canada; 28 Toronto Centre for Liver Disease, Division of Gastroenterology and Hepatology, University of Toronto, Toronto, Ontario, Canada; 29 Dept of Medicine, University of Alberta, Edmonton, Alberta, Canada; 30 Universite de Montreal Hospital Centre, Saint-Luc Hospital, Montreal, Quebec, Canada; 31 Lunenfeld Tanenbaum Research Institute, Toronto, Canada; 32 Lunenfeld Tanenbaum Research Institute, Toronto, Canada; 33 Division of Gastroenterology and Hepatology, Key Laboratory of Gastroenterology and Hepatology, Ministry of Health, State Key Laboratory for Oncogenes and Related Genes, Renji Hospital, School of Medicine, Shanghai Jiao Tong University, Shanghai Institute of Digestive Disease, China; 34 Bio-X Institutes, Key Laboratory for the Genetics of Developmental and Neuropsychiatric Disorders (Ministry of Education), Collaborative Innovation Center for Brain Science, Shanghai Jiao Tong University, China; 35 Affiliated Hospital of Qingdao University and Biomedical Sciences Institute of Qingdao University (Qingdao Branch of SJTU Bio-X Institutes), Qingdao University, China; 36 Azienda Ospedaliera Ospedali Riuniti Villa Sofia-Cervello, Palermo, Italy; 37 Gastroenterology & Hepatology Unit, Di.Bi.M.I.S., University of Palermo, Palermo, Italy; 38 Department of Medico-Surgical Sciences and Biotechnologies, Polo Pontino, University Sapienza of Rome; Eleonora Lorillard Spencer-Cenci Foundation, Rome, Italy; 39 Department of Medical and Surgical Sciences, Bologna University, Bologna, Italy; 40 IRCCS Casa Sollievo della Sofferenza Hospital, San Giovanni Rotondo, Italy; 41 Department of Medical and Surgical Sciences (DIMEC) University of Bologna, Bologna, Italy; 42 San Paolo Hospital Medical School, UniversitÃ di Milano, Milan, Italy; 43 UniversitÃÂ Politecnica delle Marche, Ancona, Italy; 44 Department of Medico-Surgical Sciences and Biotechnologies, Polo Pontino, University Sapienza of Rome, Rome, Italy; 45 Azienda Ospedaliera Universitaria Pisana, Pisa, Italy; 46 Department of Internal Medicine, Ospedale Fatebene Fratelli e Oftalmico, Milan, Italy; 47 Sezione di Gastroenterologia e Epatologia, Dipartimento Biomedico di Medicina Interna e Specialistica (Di.Bi.M.I.S.) University of Palermo, Palermo, Italy; 48 Department of Medico-Surgical Sciences and Biotechnologies, Sapienza University of Rome, Viale dell'UniversitÃÂ 37, 00185, Rome, Italy; 49 Medical Department, Desio Hospital, Desio, Italy; 50 Department of Surgery, Oncology and Gastroenterology, University of Padua, Padova, Italy; 51 Division of Gastroenterology and Center for Autoimmune Liver Diseases, Department of Medicine and Surgery, University of Milano-Bicocca, Monza, Italy; 52 Azienda Ospedaliera Universitaria Pisana, Pisa, Italy; 53 Department of Internal Medicine, AO Provincia di Lecco, Lecco, Italy; 54 Department of Internal Medicine, San Pietro Hospital, Bergamo, Ponte San Pietro, Italy; 55 Humanitas Clinical and Research Center, IRCCS, Rozzano, Italy; 56 Treviglio Hospital, Treviglio, Italy; 57 Division of Gastroenterology and Center for Autoimmune Liver Diseases, Department of Medicine and Surgery, University of Milano-Bicocca, Monza, Italy; 58 Hepatology Unit, Department of Medical and Surgical Sciences, University Hospital of Bologna, Italy; 59 University of Trieste, & Fondazione Italiana Fegato (FIF) Trieste, Italy; 60 San Paolo Hospital Medical School, UniversitÃÂ di Milano, Milan, Italy; 61 Division of Gastroenterology and Center for Autoimmune Liver Diseases, Department of Medicine and Surgery, University of Milano-Bicocca, Monza, Italy; 62 Fondazione IRCCS Ca’ Granda, Ospedale Maggiore Policlinico, Milan, Italy; 63 Azienda Ospedaliero-Universitaria di Parma, Parma, Italy; 64 University of Padova, Padova, Italy; 65 Gastroenterologia Epatologia e Trapiantologia, Papa Giovanni XXIII Hospital, Bergamo, Italy; 66 Azienda Ospedaliero-Universitaria di Parma, Parma, Italy; 67 Department. of Surgical, Oncological and Gastroenterological Sciences, University of Padova, Padova, Italy; 68 University of Florence, Florence, Italy; 69 Gastroenterology Unit, Department Internal Medicine, Policlinico San Martino, University of Genoa, Genoa, Italy; 70 Italian College of General Practicioners, ASL Bari, Italy; 71 Division of Gastroenterology and Hepatology, Fondazione IRCCS Ca' Granda Ospedale Maggiore Policlinico, Milan, Italy; 72 Department of Biomedical Sciences, Humanitas University, Division of Internal Medicine and Hepatology, Department of Gastroenterology, Humanitas Clinical and Research Center IRCCS, Via A. Manzoni 56, 20089 Rozzano (MI), Italy; 73 Division of Gastroenterology and Center for Autoimmune Liver Diseases, Department of Medicine and Surgery, University of Milano-Bicocca, Monza, Italy; 74 Division of Gastroenterology and Center for Autoimmune Liver Diseases, Department of Medicine and Surgery, University of Milano-Bicocca, Monza, Italy; 75 University of Florence, Florence, Italy; 76 UniversitÃ Politecnica delle Marche, Ancona, Italy; 77 Division of Gastroenterology and Center for Autoimmune Liver Diseases, Department of Medicine and Surgery, University of Milano-Bicocca, Monza, Italy; 78 Santa Croce Carle Hospital, Cuneo, Italy; 79 Internal Medicine, Gastroenterology and Liver Unit, A. Gemelli Polyclinic, Sacro Cuore Catholic University, 20123 Rome, Italy; 80 Division of Gastroenterology and Center for Autoimmune Liver Diseases, Department of Medicine and Surgery, University of Milano-Bicocca, Monza, Italy; 81 Magenta Hospital, Magenta, Italy; 82 University of Naples, Federico II, Naples, Italy; 83 Department of Clinical Medicine, University of Bologna, Bologna, Italy; 84 Department of Clinical Medicine, University of Bologna, Bologna, Italy; 85 IRCCS Casa Sollievo della Sofferenza Hospital, San Giovanni Rotondo, Italy; 86 Division of Gastroenterology and Center for Autoimmune Liver Diseases, Department of Medicine and Surgery, University of Milano-Bicocca, Monza, Italy; 87 University of Genoa, Genoa, Italy; 88 Department of Interdisciplinary Medicine, University Medical School, Bari, Italy; 89 Department of Translational Medicine, UniversitÃÂ del Piemonte Orientale UPO, 28100 Novara, Italy; 90 Division of Gastroenterology and Center for Autoimmune Liver Diseases, Department of Medicine and Surgery, University of Milano-Bicocca, Monza, Italy; 91 Division of Gastroenterology & Hepatology, Center for Predictive Medicine, Gradenigo Hospital, Turin, Italy; 92 Azienda Ospedaliera Valduce, Como, Italy; 93 Yale University, New Haven, Connecticut, USA; 94 University of Florence, Florence, Italy; 95 University of Trieste, & Fondazione Italiana Fegato (FIF) Trieste, Italy; 96 University of Udine, Udine, Italy; 97 Internal Medicine and Metabolic Diseases, Fondazione IRCCS Ca' Granda Ospedale Policlinico Milano, Department of Pathophysiology and Transplantation, UniversitÃÂ degli Studi di Milano, Milan, Italy; 98 Ospedale Niguarda, Milan, Italy; 99 San Paolo Hospital Medical School, UniversitÃ di Milano, Milan, Italy; 100 Clinical Research Center, National Hospital Organization (NHO) Nagasaki Medical Center, Omura, Japan; 101 Department of Gastroenterology and Rheumatic Diseases, Fukushima Medical University of Medicine, Fukushima, Japan; 102 Headquaters of PBC Research in the NHO Study Group for Liver Disease in Japan (NHOSLJ), Clinical Research Center, NHO Nagasaki Medical Center, Omura, Nagasaki, Japan; 103 Department of Medicine, Teikyo University School of Medicine, Tokyo, Japan; 104 Department of Gastroenterology and Hepatology, Tokyo Jikei University School of Medicine, Tokyo, Japan; 105 Department of Gastroenterology and Metabology, Ehime University Graduate School of Medicine, Matsuyama, Japan; 106 Department of Gastroenterology, Kanazawa University Graduate School of Medicine, Kanazawa, Japan; 107 Division of Gastroenterology, Department of Medicine, Kurume University School of Medicine, Kurume, Japan; 108 Department of Medicine and Gastroenterology, Tokyo Women’s Medical University, Tokyo, Japan; 109 Department of Medicine, Division of Gastroenterology and Hepatology, Shinshu University School of Medicine, Matsumoto, Japan; 110 Department of Gastroenterology and Hepatology, Nagasaki University Graduate School of Biomedical Sciences, Nagasaki, Japan; 111 Division of Gastroenterology and Hepatology,Niigata University Graduate School of Medical and Dental Sciences, Niigata, Japan; 112 Faculty of Medicine, Oita University, Oita, Japan; 113 Department of Gastroenterology and Medicine, Fukuoka University School of Medicine, Fukuoka, Japan; 114 The Third Department of Internal Medicine, School of Medicine, University of Occupational and Environmental Health, Kitakyushu, Japan; 115 Department of Medicine and Clinical Oncology, Graduate School of Medicine, Chiba University, Chiba, Japan; 116 Department of Gastroenterology, Yamagata University Faculty of Medicine, Yamagata, Japan; 117 Department of Internal Medicine, Teikyo University Mizonokuchi Hospital, Kawasaki, Japan; 118 Division of Gastroenterology and Hepatology, Department of Internal Medicine, Keio Graduate School of Medicine, Tokyo, Japan; 119 Department of Medical Technology, Kagawa Prefectural University of Health Sciences, Kagawa, Japan; 120 Department of Clinical Medicine, Institute of Tropical Medicine, Nagasaki University, Nagasakin, Japan; 121 The Research Center for Hepatitis and Immunology, National Center for Global Health and Medicine, Ichikawa, Japan; 122 Hepatology Division, Department of Internal Medicine II, Hamamatsu University School of Medicine, Hamamatsu, Shizuoka Japan; 123 Department of Medicine and Biosystemic Science, Kyushu University Graduate School of Medical Sciences, Fukuoka, Japan; 124 Department of Gastroenterology and Hepatology, Okayama University Graduate School of Medicine, Dentistry and Pharmaceutical Sciences, Okayama, Japan; 125 Department of Gastroenterology and Hepatology, Juntendo University Shizuoka Hospital, Shizuoka, Japan; 126 Department of Digestive and Lifestyle–Related Disease, Kagoshima University Graduate School of Medical and Dental Science, Kagoshima, Japan; 127 Department of Gastroenterology and Metabolism, Applied Life Sciences, Institute of Biomedical & Health Sciences, Hiroshima University, Hiroshima, Japan; 128 Department of Human Pathology, Kanazawa University Graduate School of Medicine, Kanazawa, Japan; 129 Department of Surgery and Science, Kyushu University Graduate School of Medical Sciences, Fukuoka, Japan; 130 Division of Hepato-Biliary-Pancreatic and Transplant Surgery, Department of Surgery, Graduate School of Medicine, Kyoto University, Kyoto, Japan; 131 Department of Surgery, Tokyo Women’s Medical University, Tokyo, Japan; 132 Organ Transplantation Service, The University of Tokyo, Tokyo, Japan; 133 Hepatobiliary and Pancreatic Surgery Division and Artificial Organ and Transplantation Division, Department of Surgery, Graduate School of Medicine, The University of Tokyo, Japan; 134 Division of Gastroenterology and Hepatology, Mayo Clinic, Rochester, Minnesota, United States; 135 Division of Biomedical Statistics and Informatics Mayo Clinic, Rochester, Minnesota, United States; 136 Division of Gastroenterology and Hepatology, Mayo Clinic, Rochester, Minnesota, United States; 137 Division of Biomedical Statistics and Informatics Mayo Clinic, Rochester, Minnesota, United States; 138 Division of Gastroenterology and Hepatology, Mayo Clinic, Rochester, Minnesota, United States; 139 Indiana University, Indiana, United States; 140 Virginia Commonwealth University, Virginia, United States; 141 Icahn School of Medicine, Mount Sinai, New York, United States; 142 University of Pittsburgh, United States; 143 Regeneron, United States; 144 Aintree University Hospital, Aintree University Hospitals NHS Foundation Trust, United Kingdom; 145 Airedale General Hospital, Airedale NHS Foundation Trust, United Kingdom; 146 Nevill Hall Hospital, Royal Gwent Hospital, Ysbyty Ystrad Fawr, Aneurin Bevan University Health Board, United Kingdom; 147 Ashford Hospital, St Peter's Hospital, Ashford and St Peter's NHS Foundation Trust, United Kingdom; 148 Queen's Hospital, King George Hospital, Barking, Havering and Redbridge University Hospitals NHS Trust, United Kingdom; 149 Barnsley Hospital, Barnsley Hospital NHS Foundation Trust, United Kingdom; 150 Newham University Hospital, St Bartholemew's Hospital, The Royal London Hospital, Whipps Cross University Hospital, Barts Health NHS Trust, United Kingdom; 151 Basildon University Hospital, Basildon and Thurrock University Hospitals NHS Foundation Trust, United Kingdom; 152 Bedford Hospital, Bedford Hospitals NHS Trust, United Kingdom; 153 Royal Victoria Hospital, Belfast Health and Social Care Trust, United Kingdom; 154 Glan Clwyd Hospital, Betsi Cadwaladr University Health Board, United Kingdom; 155 Llandudno General Hospital, Betsi Cadwaladr University Health Board, United Kingdom; 156 Wrexham Maelor Hospital, Betsi Cadwaladr University Health Board, United Kingdom; 157 Ysbyty Gwynedd, Betsi Cadwaladr University Health Board, United Kingdom; 158 Blackpool Victoria Hospital, Blackpool Teaching Hospitals NHS Foundation Trusts, United Kingdom; 159 Royal Bolton Hospital, Bolton NHS Foundation Trust, United Kingdom; 160 Bradford Royal Infirmary, Bradford Teaching Hospitals NHS Foundation Trust, United Kingdom; 161 Royal Sussex County Hospital, Brighton and Sussex University Hospitals NHS Trust, United Kingdom; 162 Amersham Hospital, Stoke Mandeville Hospital, Wycombe Hospital, Buckinghamshire Healthcare NHS Trust, United Kingdom; 163 Calderdale Royal Hospital, Huddersfield Royal Infirmary, Calderdale and Huddersfield NHS trust, United Kingdom; 164 Addenbrooke's Hospital, Cambridge University Hospitals NHS Foundation Trust, United Kingdom; 165 University Hospital Llandough, University Hospital of Wales, Cardiff and Vale University Health Board, United Kingdom; 166 Chelsea and Westminster Hospital, Chelsea & Westminster NHS Foundation Trust, United Kingdom; 167 West Middlesex University Hospital, Chelsea & Westminster NHS Foundation Trust, United Kingdom; 168 Chesterfield Royal Hospital, Chesterfield Royal Hospital NHS Foundation Trust, United Kingdom; 169 Countess of Chester Hospital, Countess of Chester Hospital NHS Foundation Trust, United Kingdom; 170 Darlington Memorial Hospital, University Hospital of North Durham, County Durham and Darlington NHS Foundation Trust, United Kingdom; 171 Croydon University Hospital, Purley War Memorial Hospital, Croydon Health Services NHS Trust, United Kingdom; 172 Princess of Wales Hospital, Cwm Taf Morgannwg University Health Board, United Kingdom; 173 Prince Charles Hospital, Royal Glamorgan Hospital, Ysbyty Cwm Cynon, Cwm Taf Morgannwg University Health Board, United Kingdom; 174 Darent Valley Hospital, Queen Mary's Hospital Sidcup, Dartford And Gravesham NHS Trust, United Kingdom; 175 Bassetlaw Hospital, Doncaster Royal Infirmary, Doncaster and Bassetlaw Hospitals NHS Foundation Trust, United Kingdom; 176 Dorset County Hospital, Dorset County Hospital NHS Foundation Trust, United Kingdom; 177 Russells Hall Hospital, Dudley Group of Hospitals NHS Trust, United Kingdom; 178 Lister Hospital, Queen Elizabeth II Hospital, East and North Hertfordshire NHS Trust, United Kingdom; 179 Macclesfield District General Hospital, East Cheshire NHS Trust, United Kingdom; 180 Buckland Hospital, Kent and Canterbury Hospital, Queen Elizabeth The Queen Mother Hospital, William Harvey Hospital Ashford, East Kent Hospitals University NHS Foundation Trust, United Kingdom; 181 Burnley General Hospital, Royal Blackburn Hospital, East Lancashire Hospitals NHS Trust, United Kingdom; 182 Colchester General Hospital, East Suffolk and North Essex NHS Foundation Trust, United Kingdom; 183 Ipswich Hospital, East Suffolk and North Essex Foundation Trust, United Kingdom; 184 Conquest Hospital, Eastbourne District General Hospital, East Sussex Healthcare NHS Trust, United Kingdom; 185 Epsom General Hospital, Epsom and St Helier University Hospitals NHS Trust, United Kingdom; 186 Frimley Park Hospital, Frimley Health NHS Foundation Trust, United Kingdom; 187 Heatherwood Hospital, Wexham Park Hospital, Frimley Health NHS Foundation Trust, United Kingdom; 188 Queen Elizabeth Hospital, Gateshead Health NHS Foundation Trust, United Kingdom; 189 George Eliot Hospital, George Eliot Hospital NHS Trust, United Kingdom; 190 Cheltenham General Hospital, Gloucestershire Royal Hospital, Gloucestershire Hospitals NHS Foundation Trust, United Kingdom; 191 Guy's Hospital, St Thomas' Hospital, Guy's and St Thomas' NHS Foundation Trust, United Kingdom; 192 Basingstoke and North Hampshire Hospital, Hampshire Hospitals Foundation trust, United Kingdom; 193 Royal Hampshire County Hospital, Hampshire Hospitals NHS Foundation Trust, United Kingdom; 194 Harrogate District Hospital, Harrogate and District Foundation Trust, United Kingdom; 195 Good Hope Hospital, Heartlands Hospital, Solihull Hospital, Heart of England NHS Foundation Trust, United Kingdom; 196 Hillingdon Hospital, Hillingdon Hospitals NHS Foundation Trust, United Kingdom; 197 Homerton University Hospital, Homerton University Hospital NHS Foundation Trust, United Kingdom; 198 Castle Hill Hospital, Hull Royal Infirmary, Hull University Teaching Hospitals NHS Trust, United Kingdom; 199 Bronglais Hospital, Hywel Dda University Health Board, United Kingdom; 200 Prince Philip Hospital, Withybush General Hospital, Central Middlesex Hospital, Glangwili General Hospital, Hywel Dda University Health Board, United Kingdom; 201 Charing Cross Hospital, Hammersmith Hospital, St Mary's Hospital, Imperial College, Imperial College Healthcare NHS Trust, United Kingdom; 202 St Mary's Hospital, Imperial College, Imperial College Healthcare NHS Trust, United Kingdom; 203 St Mary's Hospital, Isle of Wight, Isle of Wight NHS Trust, United Kingdom; 204 James Paget University Hospital, James Paget University Hospitals NHS Foundation Trust, United Kingdom; 205 Kettering General Hospital, Kettering General Hospital NHS Foundation Trust, United Kingdom; 206 King's College Hospital, King's College Hospital, United Kingdom; 207 Beckenham Beacon, Orpington Hospital, Princess Royal University Hospital, King's College Hospital NHS Foundation Trust, United Kingdom; 208 Kingston Hospital, Kingston Hospital NHS Foundation Trust, United Kingdom; 209 Chorley and South Ribble Hospital, Royal Preston Hospital, Lancashire Teaching Hospitals NHS Foundation Trust, United Kingdom; 210 St James's University Hospital, Leeds Teaching Hospitals NHS Trust, United Kingdom; 211 Lewisham Hospital, University Hospital Lewisham, Lewisham and Greenwich NHS Trust, United Kingdom; 212 Queen Elizabeth Hospital, Lewisham and Greenwich NHS Trust, United Kingdom; 213 Central Middlesex Hospital, Northwick Park and St Mark's Hospitals, London North West Healthcare NHS Trust, United Kingdom; 214 Luton and Dunstable University Hospital, Luton and Dunstable University Hospital, United Kingdom; 215 Maidstone Hospital, Tunbridge Wells Hospital, Maidstone and Tunbridge Wells NHS Trust, United Kingdom; 216 Manchester Royal Infirmary, Trafford General Hospital, Manchester University NHS Foundation Trust, United Kingdom; 217 Wythenshawe Hospital, Manchester University NHS Foundation Trust, United Kingdom; 218 Medway Maritime Hospital, Medway NHS Foundation Trust, United Kingdom; 219 Broomfield Hospital, St Peters Hospital, Mid-Essex Hospitals NHS Trust, United Kingdom; 220 Dewsbury and District Hospital, Mid Yorkshire Hospitals NHS Trust, United Kingdom; 221 Milton Keynes Hospital, Milton Keynes Hospital NHS Foundation Trust, United Kingdom; 222 University Hospital Crosshouse, NHS Ayrshire & Arran, United Kingdom; 223 Borders General Hospital, NHS Borders, United Kingdom; 224 Dumfries and Galloway Royal Infirmary, NHS Dumfries & Galloway, United Kingdom; 225 Queen Margaret Hospital, Victoria Hospital, NHS Fife, United Kingdom; 226 Falkirk Community Hospital, Forth Valley Royal Hospital, Stirling Community Hospital, NHS Forth Valley, United Kingdom; 227 Aberdeen Royal Infirmary, Dr Gray's Hospital, NHS Grampian, United Kingdom; 228 Gartnavel General Hospital, Glasgow Royal Infirmary, Inverclyde Royal Hospital, Royal Alexandra Hospital, Southern General Hospital, Victoria Infirmary, NHS Greater Glasgow and Clyde, United Kingdom; 229 Hairmyres Hospital, Monklands Hospital, Wishaw General Hospital, NHS Lanarkshire, United Kingdom; 230 Royal Infirmary of Edinburgh, St John's Hospital, NHS Lothian, United Kingdom; 231 Western General Hospital, NHS Lothian, United Kingdom; 232 Ninewells Hospital, NHS TaWyside, United Kingdom; 233 Norfolk and Norwich University Hospital, Norfok and Norwich University Hospitals NHS Foundation Trust, United Kingdom; 234 Southmead Hospital, North Bristol NHS Trust, United Kingdom; 235 Cumberland Infirmary, West Cumberland Hospital, North Cumbria University Hospitals NHS Foundation Trust, United Kingdom; 236 North Middlesex Hospital, North Middlesex University Hospital NHS Trust, United Kingdom; 237 University Hospital of Hartlepool, University Hospital of North Tees, North Tees and Hartlepool NHS Foundation Trust, United Kingdom; 238 Hinchingbrooke Hospital, North West Anglia NHS Trust, United Kingdom; 239 Peterborough City Hospital, Stamford & Rutland Hospital, North West Anglia NHS Foundation Trust, United Kingdom; 240 Northampton General Hospital, Northampton General Hospital NHS Trust, United Kingdom; 241 North Devon District Hospital, Northern Devon Healthcare NHS Trust, United Kingdom; 242 Diana Princess of Wales Hospital, Scunthorpe General Hospital, Northern Lincolnshire and Goole NHS Foundation Trust, United Kingdom; 243 North Tyneside General Hospital, Northumbria Healthcare NHS Foundation Trust, United Kingdom; 244 Nottingham City Hospital, Queen's Medical Centre, NIHR Nottingham Biomedical Research Centre at Nottingham University Hospitals NHS Trust, United Kingdom; 245 John Radcliffe Hospital, Oxford University Hospitals NHS Trust, United Kingdom; 246 Fairfield General Hospital, North Manchester General Hospital, Rochdale Infirmary, The Royal Oldham Hospital, Pennine Acute Hospitals NHS Trust, United Kingdom; 247 Derriford Hospital, University Hospitals Plymouth NHS Trust, United Kingdom; 248 Poole Hospital, Poole Hospital NHS Foundation Trust, United Kingdom; 249 Queen Alexandra Hospital, Portsmouth Hospitals NHS Trust, United Kingdom; 250 St Margaret's Hospital, The Princess Alexandra Hospital, Princess Alexandra Hospital NHS Trust, United Kingdom; 251 The Queen Elizabeth Hospital King's Lynn, King's Lynn NHS Foundation Trust, United Kingdom; 252 Royal Berkshire Hospital, Royal Berkshire NHS Foundation Trust, United Kingdom; 253 Royal Bournemouth Hospital, Royal Bournemouth and Christchurch Hospitals NHS Foundation Trust, United Kingdom; 254 Royal Cornwall Hospital, Royal Cornwall Hospitals NHS Trust, United Kingdom; 255 Royal Devon and Exeter Hospital, Royal Devon And Exeter Nhs Foundation Trust, United Kingdom; 256 Barnet and Chase Farm Hospitals, Royal Free London NHS Trust, United Kingdom; 257 The Royal Free Hospital, Royal Free London NHS Foundation Trust, United Kingdom; 258 Royal Liverpool University Hospital, Royal Liverpool and Broadgreen University Hospitals NHS Trust, United Kingdom; 259 Royal Surrey County Hospital, Royal Surrey County Hospital NHS Foundation Trust, United Kingdom; 260 Royal United Bath Hospital, Royal United Hospitals Bath NHS Foundation Trust, United Kingdom; 261 Cannock Chase Hospital, Royal Wolverhampton Hospitals NHS Trust, United Kingdom; 262 New Cross Hospital, Royal Wolverhampton NHS Trust, United Kingdom; 263 Salisbury District Hospital, Salisbury NHS Foundation Trust, United Kingdom; 264 Sandwell General Hospital, Sandwell & West Birmingham Hospitals NHS Trust, United Kingdom; 265 Northern General Hospital, Royal Hallamshire Hospital, Sheffield Teaching Hospitals NHS Foundation Trust, United Kingdom; 266 King's Mill Hospital, Newark Hospital, Sherwood Forest Hospitals NHS Foundation Trust, United Kingdom; 267 Princess Royal Hospital, Royal Shrewsbury Hospital, Shrewsbury and Telford Hospital NHS Trust, United Kingdom; 268 Torbay Hospital, Torbay and South Devon NHS Foundation Trust, United Kingdom; 269 Lagan Valley Hospital, Ulster Hospital, South Eastern Health and Social Care Trust, United Kingdom; 270 Friarage Hospital, The James Cook University Hospital, South Tees Hospitals NHS Foundation Trust, United Kingdom; 271 Sunderland Royal Hospital, Sunderland Royal Hospital, South Tyneside and Sunderland NHS Foundation Trust, United Kingdom; 272 South Tyneside District Hospital, South Tyneside and Sunderland NHS Foundation Trust, United Kingdom; 273 Warwick Hospital, South Warwickshire NHS Foundation Trust, United Kingdom; 274 Southend University Hospital, Southend University Hospital NHS Foundation Trust, United Kingdom; 275 Ormskirk District General Hospital, Southport and Formby District General Hospital, Southport & Ormskirk Hospital NHS Trust, United Kingdom; 276 St George's Hospital, St George’s University Hospitals NHS Foundation Trust, United Kingdom; 277 Stepping Hill Hospital, Stockport NHS Foundation Trust, United Kingdom; 278 East Surrey Hospital, Surrey and Sussex Healthcare NHS Trust, United Kingdom; 279 Morriston Hospital, Singleton Hospital, Swansea Bay University Health Board, United Kingdom; 280 Neath Port Talbot Hospital, Swansea Bay University Health Board, United Kingdom; 281 Tameside General Hospital, Tameside and Glossop integrated care NHS Trust, United Kingdom; 282 Musgrove Park Hospital, Taunton and Somerset NHS Foundation Trust, United Kingdom; 283 Lincoln County Hospital, United Lincolnshire Hospitals NHS Trust, United Kingdom; 284 Pilgrim Hospital Boston, United Lincolnshire Hospitals NHS Trust, United Kingdom; 285 University College Hospital, University College London Hospitals NHS Foundation Trust, United Kingdom; 286 Southampton General Hospital, University Hospital Southampton NHS Foundation Trust, United Kingdom; 287 The Queen Elizabeth Hospital, University Hospitals Birmingham NHS Foundation Trust, United Kingdom; 288 Bristol Royal Infirmary, University Hospitals Bristol NHS Foundation Trust, United Kingdom; 289 University Hospital, Coventry, University Hospital Coventry and Warwickshire NHS Trust, United Kingdom; 290 Queen's Hospital, University Hospitals of Derby and Burton NHS Foundation Trust, United Kingdom; 291 Royal Derby Hospital, University Hospitals of Derby and Burton NHS Foundation Trust, United Kingdom; 292 Glenfield Hospital, Leicester General Hospital, Leicester Royal Infirmary, University Hospitals of Leicester NHS Trust, United Kingdom; 293 Royal Lancaster Infirmary, University Hospitals of Morecambe Bay NHS Foundation Trust, United Kingdom; 294 Royal Stoke University Hospital, University Hospitals of North Midlands NHS Trust, United Kingdom; 295 County Hospital, University Hospitals of North Midlands NHS Trust, United Kingdom; 296 Walsall Manor Hospital, Walsall Healthcare NHS Trust, United Kingdom; 297 Warrington Hospital, Warrington and Halton Hospitals NHS Foundation Trust, United Kingdom; 298 Hemel Hempstead General Hospital, St Albans City Hospital, Watford General Hospital, West Hertfordshire Hospitals NHS Trust, United Kingdom; 299 West Suffolk Hospital, West Suffolk NHS Foundation Trust, United Kingdom; 300 St Richard's Hospital, Western Sussex Hospitals NHS Foundation Trust, United Kingdom; 301 Worthing Hospital, Western Sussex Hospitals NHS Foundation Trust, United Kingdom; 302 Weston General Hospital, Weston Area Health Trust, United Kingdom; 303 The Whittington Hospital, Whittington Hospital NHS Trust, United Kingdom; 304 Arrowe Park Hospital, Victoria Central Hospital, Wirral University Teaching Hospital NHS Foundation Trust, United Kingdom; 305 Alexandra Hospital, Kidderminster Hospital and Treatment Centre, Worcestershire Royal Hospital, Worcestershire Acute Hospitals NHS trust, United Kingdom; 306 Royal Albert Edward Infirmary, Wrightington, Wigan And Leigh NHS Trust, United Kingdom; 307 The County Hospital, Wye Valley NHS Trust, United Kingdom; 308 Yeovil District Hospital, Yeovil District Hospital NHS Foundation Trust, United Kingdom; 309 Bridlington Hospital, Scarborough Hospital, The York Hospital, York Teaching Hospitals NHS Foundation Trust, United Kingdom; 1 Population Health Sciences Institute, Faculty of Medical Sciences, Newcastle University, Newcastle upon Tyne, United Kingdom; 2 Genome Medical Science Project, National Center for Global Health and Medicine (NCGM), Tokyo, Japan; 3 Clinical Research Center, National Hospital Organization, Nagasaki Medical Center, Omura, Japan; 4 Department of Human Genetics, Graduate School of Medicine, The University of Tokyo, Tokyo, Japan; 5 Human Biosciences Unit for the Top Global Course Center for the Promotion of Interdisciplinary Education and Research, Kyoto University, Kyoto, Japan; 6 Center for Genomic Medicine, Graduate School of Medicine, Kyoto University, Kyoto, Japan; 7 Division of Gastroenterology and Hepatology, Key Laboratory of Gastroenterology and Hepatology, Ministry of Health, State Key Laboratory for Oncogenes and Related Genes, Renji Hospital, School of Medicine, Shanghai JiaoTong University, Shanghai Institute of Digestive Disease, Shanghai, China; 8 Bio-X Institutes, Key Laboratory for the Genetics of Developmental and Neuropsychiatric Disorders (Ministry of Education), Collaborative Innovation Center for Brain Science, Shanghai Jiao Tong University, Shanghai, China; 9 Affiliated Hospital of Qingdao University and Biomedical Sciences Institute of Qingdao University (Qingdao Branch of SJTU Bio-X Institutes), Qingdao University, Qingdao, China; 10 Division of Gastroenterology and Hepatology, Mayo Clinic, Rochester, Minnesota, United States; 11 Division of Biomedical Statistics and Informatics, Mayo Clinic, Rochester, Minnesota, United States; 12 Division of Gastroenterology and Center for Autoimmune Liver Diseases, Department of Medicine and Surgery, University of Milano-Bicocca, Monza, Italy; 13 European Reference Network on Hepatological Diseases (ERN RARE-LIVER), San Gerardo Hospital, Monza, Italy; 14 Department of Biomedical Sciences, Humanitas University, Pieve Emanuele, Milan, Italy; 15 Humanitas Clinical and Research Center, IRCCS, Rozzano, Milan, Italy; 16 Regeneron Genetics Center, Tarrytown, New York, United States; 17 Translational and Clinical Research Institute, Faculty of Medical Sciences, Newcastle University, Newcastle upon Tyne, United Kingdom; 18 Academic Department of Medical Genetics, University of Cambridge, Cambridge, United Kingdom; 19 Institute for Clinical and Translational Research, Baylor College of Medicine, Houston, Texas, United States; 20 Toronto Centre for Liver Disease, Division of Gastroenterology and Hepatology, University of Toronto, Toronto, Ontario, Canada; 21 University of California, Davis, California, United States; 22 Departments of Medicine, Immunology and Medical Sciences, University of Toronto, Toronto, Ontario, Canada; 23 Mount Sinai Hospital, Lunenfeld-Tanenbaum Research Institute and Toronto General Research Institute, Toronto, Ontario, Canada; 24 Department of Hepatology, Nagasaki University Graduate School of Biomedical Sciences, Omura, Japan

It has come to our attention that the name of one of the authors in our manuscript was incorrectly spelled ‘Jinyoung Byan’; the correct spelling is ‘Jinyoung Byun’ as in the author list above. In addition, the excel files of the supplementary tables were not included during the online publication of our article. These have now been made available online. We apologize for any inconvenience caused.

