# Supplementary material for: An international genome-wide meta-analysis of primary biliary cholangitis: Novel risk loci and candidate drugs
Source: J Hepatol. 2021 Sep;75(3):572–81. doi: 10.1016/j.jhep.2021.04.055 (PMC8811537; doi:10.1016/j.jhep.2021.04.055)
Supplement: Multimedia component 1 [file mmc1.pdf]

# An international genome-wide meta-analysis of primary biliary cholangitis: Novel risk loci and candidate drugs

Heather J. Cordell, James J. Fryett, Kazuko Ueno, Rebecca Darlay, Yoshihiro Aiba, Yuki Hitomi, Minae Kawashima, Nao Nishida, Seik-Soon Khor, Olivier Gervais, Yosuke Kawai, Masao Nagasaki, Katsushi Tokunaga, Ruqi Tang, Yongyong Shi, Zhiqiang Li, Brian D. Juran, Elizabeth J. Atkinson, Alessio Gerussi, Marco Carbone, Rosanna Asselta, Angela Cheung, Mariza de Andrade, Aris Baras, Julie Horowitz, Manuel A.R. Ferreira, Dylan Sun, David E. Jones, Steven Flack, Ann Spicer, Victoria L. Mulcahy, Jinyoung Byan, Younghun Han, Richard N. Sandford, Konstantinos N. Lazaridis, Christopher I. Amos, Gideon M. Hirschfield, Michael F. Seldin, Pietro Invernizzi, Katherine A. Siminovitch, Xiong Ma, Minoru Nakamura, and George F. Mells for the Canadian PBC Consortium, Chinese PBC Consortium, Italian PBC Study Group, Japan-PBC-GWAS Consortium, US PBC Consortium, and UK-PBC Consortium

Table of contents

PBC Consortia ........ 3
Canadian PBC Consortium ........ 3
Chinese PBC Consortium ........ 3
Italian PBC Study Group ........ 4
Japanese PBC GWAS Consortium ........ 7
UK PBC Consortium ........ 10
US PBC Consortium ........ 18
Supplementary materials and methods ........ 20
Fig. S1 ........ 34
Fig. S2 ........ 35

Fig. S3. .......................................................................................................... 98
Fig. S4.......................................................................................................... 103
Fig. S5.......................................................................................................... 109
Fig. S6.......................................................................................................... 111
Fig. S7.......................................................................................................... 112
Fig. S8.. ........................................................................................................ 113
Fig. S9.......................................................................................................... 114
References.................................................................................................... 115
Appendix....................................................................................................... 117

**PBC Consortia**

*Canadian PBC Consortium*

**Katherine A. Siminovitch**, Departments of Medicine, Immunology and Medical Sciences, University of Toronto, and Mount Sinai Hospital, Lunenfeld-Tanenbaum Research Institute and Toronto General Research Institute, Toronto, Ontario, Canada; **Gideon M. Hirschfield**, Toronto Centre for Liver Disease, Division of Gastroenterology and Hepatology, University of Toronto, Toronto, Ontario, Canada; **Andrew Mason**, Dept of Medicine, University of Alberta, Edmonton, Alberta; **Catherine Vincent**, Universite de Montreal Hospital Centre, Saint-Luc Hospital, Montreal, Quebec; **Gang Xie**, Lunenfeld Tanenbaum Research Institute, Toronto; **Jinyi Zhang**, Lunenfeld Tanenbaum Research Institute, Toronto.

*Chinese PBC Consortium*

**Ruqi Tang**, **Xiong Ma**, Division of Gastroenterology and Hepatology, Key Laboratory of Gastroenterology and Hepatology, Ministry of Health, State Key Laboratory for Oncogenes and Related Genes, Renji Hospital, School of Medicine, Shanghai Jiao Tong University, Shanghai Institute of Digestive Disease; **Zhiqiang Li**, **Yongyong Shi**, Bio-X Institutes, Key Laboratory for the Genetics of Developmental and Neuropsychiatric Disorders (Ministry of Education), Collaborative Innovation Center for Brain Science, Shanghai Jiao Tong University & Affiliated Hospital of Qingdao University and Biomedical Sciences Institute of Qingdao University (Qingdao Branch of SJTU Bio-X Institutes), Qingdao University.

*Italian PBC Study Group*

**Andrea Affronti**, Azienda Ospedaliera Ospedali Riuniti Villa Sofia-Cervello, Palermo; **Piero L. Almasio**, Gastroenterology & Hepatology Unit, Di.Bi.M.I.S., University of Palermo, Palermo; **Domenico Alvaro**, Department of Medico-Surgical Sciences and Biotechnologies, Polo Pontino, University Sapienza of Rome; Eleonora Lorillard Spencer-Cenci Foundation, Rome; **Pietro Andreone**, Department of Medical and Surgical Sciences, Bologna University, Bologna; **Angelo Andriulli**, IRCCS Casa Sollievo della Sofferenza Hospital, San Giovanni Rotondo; **Francesco Azzaroli**, Department of Medical and Surgical Sciences (DIMEC) University of Bologna, Bologna; **Pier Maria Battezzati**, San Paolo Hospital Medical School, Università di Milano, Milan; **Antonio Benedetti**, Università Politecnica delle Marche, Ancona; **MariaConsiglia Bragazzi**, Department of Medico-Surgical Sciences and Biotechnologies, Polo Pontino, University Sapienza of Rome, Rome; **Maurizia Brunetto**, Azienda Ospedaliera Universitaria Pisana, Pisa; **Savino Bruno**, Department of Internal Medicine, Ospedale Fatebene Fratelli e Oftalmico, Milan; **Vincenza Calvaruso**, Sezione di Gastroenterologia e Epatologia, Dipartimento Biomedico di Medicina Interna e Specialistica (Di.Bi.M.I.S.) University of Palermo, Palermo; **Vincenzo Cardinale**, Department of Medico-Surgical Sciences and Biotechnologies, Sapienza University of Rome, Viale dell'Università 37, 00185, Rome; **Giovanni Casella**, Medical Department, Desio Hospital, Desio; **Nora Cazzagon**, Department of Surgery, Oncology and Gastroenterology, University of Padua, Padova; **Antonio Ciaccio**, Division of Gastroenterology and Center for Autoimmune Liver Diseases, Department of Medicine and Surgery, University of Milano-Bicocca, Monza; **Barbara Coco**, Azienda Ospedaliera Universitaria Pisana, Pisa; **Agostino Colli**, Department of Internal Medicine, AO Provincia di Lecco, Lecco; **Guido**

**Colloredo**, Department of Internal Medicine, San Pietro Hospital, Bergamo, Ponte San Pietro; **Massimo Colombo**, Humanitas Clinical and Research Center, IRCCS, Rozzano; **Silvia Colombo**, Treviglio Hospital, Treviglio; **Laura Cristoferi**, Division of Gastroenterology and Center for Autoimmune Liver Diseases, Department of Medicine and Surgery, University of Milano-Bicocca, Monza; **Carmela Cursaro**, Hepatology Unit, Department of Medical and Surgical Sciences, University Hospital of Bologna; **Lory Saveria Crocè**, University of Trieste, & Fondazione Italiana Fegato (FIF) Trieste; **Andrea Crosignani**, San Paolo Hospital Medical School, Università di Milano, Milan; **Daphne D'Amato**, Division of Gastroenterology and Center for Autoimmune Liver Diseases, Department of Medicine and Surgery, University of Milano-Bicocca, Monza; **Francesca Donato**, Fondazione IRCCS Ca' Granda, Ospedale Maggiore Policlinico, Milan; **Gianfranco Elia**, Azienda Ospedaliero-Universitaria di Parma, Parma; Luca Fabris, University of Padova, Padova; **Stefano Fagiuoli**, Gastroenterologia Epatologia e Trapiantologia, Papa Giovanni XXIII Hospital, Bergamo; **Carlo Ferrari**, Azienda Ospedaliero-Universitaria di Parma, Parma; **Annarosa Floreani**, Department. of Surgical, Oncological and Gastroenterological Sciences, University of Padova, Padova; **Andrea Galli**, University of Florence, Florence; **Edoardo Giannini**, Gastroenterology Unit, Department Internal Medicine, Policlinico San Martino, University of Genoa, Genoa; **Ignazio Grattagliano**, Italian College of General Practicioners, ASL Bari; **Pietro Lampertico**, Division of Gastroenterology and Hepatology, Fondazione IRCCS Ca' Granda Ospedale Maggiore Policlinico, Milan; **Ana Lleo**, Department of Biomedical Sciences, Humanitas University, Division of Internal Medicine and Hepatology, Department of Gastroenterology, Humanitas Clinical and Research Center IRCCS, Via A. Manzoni 56, 20089 Rozzano (MI); **Federica Malinverno**, Division of Gastroenterology and

Center for Autoimmune Liver Diseases, Department of Medicine and Surgery, University of Milano-Bicocca, Monza; **Clara Mancuso**, Division of Gastroenterology and Center for Autoimmune Liver Diseases, Department of Medicine and Surgery, University of Milano-Bicocca, Monza; **Fabio Marra**, University of Florence, Florence; **Marco Marzioni**, Università Politecnica delle Marche, Ancona; **Sara Massironi**, Division of Gastroenterology and Center for Autoimmune Liver Diseases, Department of Medicine and Surgery, University of Milano-Bicocca, Monza; **Alberto Mattalia**, Santa Croce Carle Hospital, Cuneo; **Luca Miele**, Internal Medicine, Gastroenterology and Liver Unit, A. Gemelli Polyclinic, Sacro Cuore Catholic University, 20123 Rome; **Chiara Milani**, Division of Gastroenterology and Center for Autoimmune Liver Diseases, Department of Medicine and Surgery, University of Milano-Bicocca, Monza; **Lorenzo Morini**, Magenta Hospital, Magenta; **Filomena Morisco**, University of Naples, Federico II, Naples; **Luigi Muratori**, Department of Clinical Medicine, University of Bologna, Bologna; **Paolo Muratori**, Department of Clinical Medicine, University of Bologna, Bologna; **Grazia A. Niro**, IRCCS Casa Sollievo della Sofferenza Hospital, San Giovanni Rotondo; **Sarah O'Donnell**, Division of Gastroenterology and Center for Autoimmune Liver Diseases, Department of Medicine and Surgery, University of Milano-Bicocca, Monza; **Antonio Picciotto**, University of Genoa, Genoa; **Piero Portincasa**, Department of Interdisciplinary Medicine, University Medical School, Bari; **Cristina Rigamonti**, Department of Translational Medicine, Università del Piemonte Orientale UPO, 28100 Novara; **Vincenzo Ronca**, Division of Gastroenterology and Center for Autoimmune Liver Diseases, Department of Medicine and Surgery, University of Milano-Bicocca, Monza; **Floriano Rosina**, Division of Gastroenterology & Hepatology, Center for Predictive Medicine, Gradenigo Hospital, Turin; **Giancarlo Spinzi**, Azienda Ospedaliera

Valduce, Como; **Mario Strazzabosco**, Yale University, New Haven, Connecticut, USA; **Mirko Tarocchi**, University of Florence, Florence; **Claudio Tiribelli**, University of Trieste, & Fondazione Italiana Fegato (FIF) Trieste; **Pierluigi Toniutto**, University of Udine, Udine; **Luca Valenti**, Internal Medicine and Metabolic Diseases, Fondazione IRCCS Ca' Granda Ospedale Policlinico Milano, Department of Pathophysiology and Transplantation, Università degli Studi di Milano, Milan; **Maria Vinci**, Ospedale Niguarda, Milan; **Massimo Zuin**, San Paolo Hospital Medical School, Università di Milano, Milan

*Japanese PBC GWAS Consortium*

**Hitomi Nakamura**, **Seigo Abiru**, **Shinya Nagaoka**, **Atsumasa Komori**, **Hiroshi Yatsuhashi**, **Hiromi Ishibashi**, **Masahiro Ito**, Clinical Research Center, National Hospital Organization (NHO) Nagasaki Medical Center, Omura, Japan; **Kiyoshi Migita**, **Hiromasa Ohira**, Department of Gastroenterology and Rheumatic Diseases, Fukushima Medical University of Medicine, Fukushima, Japan; **Shinji Katsushima**, **Atsushi Naganuma**, **Kazuhiro Sugi**, **Tatsuji Komatsu**, **Tomohiko Mannami**, **Kouki Matsushita**, **Kaname Yoshizawa**, **Fujio Makita**, **Toshiki Nikami**, **Hideo Nishimura**, **Hiroshi Kouno**, **Hirotaka Kouno**, **Hajime Ota**, **Takuya Komura**, **Yoko Nakamura**, **Masaaki Shimada**, **Noboru Hirashima**, **Toshiki Komeda**, **Keisuke Ario**, **Makoto Nakamuta**, **Tsutomu Yamashita**, **Kiyoshi Furuta**, **Masahiro Kikuchi**, **Noriaki Naeshiro**, **Hironao Takahashi**, **Yutaka Mano**, **Seiji Tsunematsu**, **Iwao Yabuuchi**, **Yusuke Shimada**, **Kazuhiko Yamauchi**, **Rie Sugimoto**, **Hironori Sakai**, **Eiji Mita**, **Masaharu Koda**, **Satoru Tsuruta**, **Hiroshi Kamitsukasa**, **Takeaki Sato**, **Naohiko Masaki**, **Tatsuro Kobata**, **Nobuyoshi Fukushima**, **Yukio Ohara**, **Toyokichi Muro**, **Eiichi Takesaki**, **Hitoshi Takaki**, **Tetsuo Yamamoto**, **Michio Kato**, **Yuko Nagaoki**,

**Shigeki Hayashi**, **Jinya Ishida**, **Yukio Watanabe**, **Masakazu Kobayashi**, **Michiaki Koga**, **Takeo Saoshiro**, **Michiyasu Yagura**, **Keisuke Hirata**, Headquaters of PBC Research in the NHO Study Group for Liver Disease in Japan (NHOSLJ), Clinical Research Center, NHO Nagasaki Medical Center, Omura, Nagasaki, Japan; **Atsushu Tanaka**, **Hajime Takikawa**, Department of Medicine, Teikyo University School of Medicine, Tokyo, Japan; **Mikio Zeniya**, Department of Gastroenterology and Hepatology, Tokyo Jikei University School of Medicine, Tokyo, Japan; **Masanori Abe**, **Morikazu Onji**, Department of Gastroenterology and Metabology, Ehime University Graduate School of Medicine, Matsuyama, Japan; **Shuichi Kaneko**, **Masao Honda**, **Kuniaki Arai**, Department of Gastroenterology, Kanazawa University Graduate School of Medicine, Kanazawa, Japan; **Teruko Arinaga-Hino**, Division of Gastroenterology, Department of Medicine, Kurume University School of Medicine, Kurume, Japan; **Etsuko Hashimoto**, **Makiko Taniai**, Department of Medicine and Gastroenterology, Tokyo Women's Medical University, Tokyo, Japan; **Takeji Umemura**, **Satoru Joshita**, Department of Medicine, Division of Gastroenterology and Hepatology, Shinshu University School of Medicine, Matsumoto, Japan; **Kazuhiko Nakao**, **Tatsuki Ichikawa**, **Hidetaka Shibata**, Department of Gastroenterology and Hepatology, Nagasaki University Graduate School of Biomedical Sciences, Nagasaki, Japan; **Satoshi Yamagiwa**, Division of Gastroenterology and Hepatology,Niigata University Graduate School of Medical and Dental Sciences, Niigata, Japan; **Masataka Seike**, **Koichi Honda**, Faculty of Medicine, Oita University, Oita, Japan; **Shotaro Sakisaka**, **Yasuaki Takeyama**, Department of Gastroenterology and Medicine, Fukuoka University School of Medicine, Fukuoka, Japan; **Masaru Harada**, **Michio Senju**, The Third Department of Internal Medicine, School of Medicine, University of Occupational and Environmental

Health, Kitakyushu, Japan; **Osamu Yokosuka**, **Tatsuo Kanda**, Department of Medicine and Clinical Oncology, Graduate School of Medicine, Chiba University, Chiba, Japan; **Yoshiyuki Ueno**, Department of Gastroenterology, Yamagata University Faculty of Medicine, Yamagata, Japan; **Kentaro Kikuchi**, Department of Internal Medicine, Teikyo University Mizonokuchi Hospital, Kawasaki, Japan; **Hirotoshi Ebinuma**, Division of Gastroenterology and Hepatology, Department of Internal Medicine, Keio Graduate School of Medicine, Tokyo, Japan; **Takashi Himoto**, Department of Medical Technology, Kagawa Prefectural University of Health Sciences, Kagawa, Japan; **Michio Yasunami**, Department of Clinical Medicine, Institute of Tropical Medicine, Nagasaki University, Nagasakin, Japan; **Kazumoto Murata**, **Masashi Mizokami**, The Research Center for Hepatitis and Immunology, National Center for Global Health and Medicine, Ichikawa, Japan; **Kazuhito Kawata**, Hepatology Division, Department of Internal Medicine II, Hamamatsu University School of Medicine, Hamamatsu, Shizuoka Japan; **Shinji Shimoda**, Department of Medicine and Biosystemic Science, Kyushu University Graduate School of Medical Sciences, Fukuoka, Japan; **Yasuhiro Miyake**, **Akinobu Takaki**, **Kazuhide Yamamoto**, Department of Gastroenterology and Hepatology, Okayama University Graduate School of Medicine, Dentistry and Pharmaceutical Sciences, Okayama, Japan; **Katsuji Hirano**, **Takafumi Ichida**, Department of Gastroenterology and Hepatology, Juntendo University Shizuoka Hospital, Shizuoka, Japan; **Akio Ido**, **Hirohito Tsubouchi**, Department of Digestive and Lifestyle–Related Disease, Kagoshima University Graduate School of Medical and Dental Science, Kagoshima, Japan; **Kazuaki Chayama**, Department of Gastroenterology and Metabolism, Applied Life Sciences, Institute of Biomedical & Health Sciences, Hiroshima University, Hiroshima, Japan; **Kenichi Harada**, **Yasuni Nakanuma**, Department of Human

Pathology, Kanazawa University Graduate School of Medicine, Kanazawa, Japan; **Yoshihiko Maehara**, **Akinobu Taketomi**, **Ken Shirabe**, **Yuji Soejima**, Department of Surgery and Science, Kyushu University Graduate School of Medical Sciences, Fukuoka, Japan. **Akira Mori**, **Shintaro Yagi**, **Shinji Uemoto**, Division of Hepato-Biliary-Pancreatic and Transplant Surgery, Department of Surgery, Graduate School of Medicine, Kyoto University, Kyoto, Japan. **Egawa H**, Department of Surgery, Tokyo Women's Medical University, Tokyo, Japan; **Tomohiro Tanaka**, **Noriyo Yamashiki**, Organ Transplantation Service, The University of Tokyo, Tokyo, Japan; **Sumito Tamura**, **Yasuhiro Sugawara**, **Norihiro Kokudo,** Hepatobiliary and Pancreatic Surgery Division and Artificial Organ and Transplantation Division, Department of Surgery, Graduate School of Medicine, The University of Tokyo, Japan.

*UK PBC Consortium*

**Richard Sturgess**, Aintree University Hospital, Aintree University Hospitals NHS Foundation Trust; **Christopher Healey**, Airedale General Hospital, Airedale NHS Foundation Trust; **Andrew Yeoman**, Nevill Hall Hospital, Royal Gwent Hospital, Ysbyty Ystrad Fawr, Aneurin Bevan University Health Board; **Anton VJ Gunasekera**, Ashford Hospital, St Peter's Hospital, Ashford and St Peter's NHS Foundation Trust; **Paul Kooner**, Queen's Hospital, King George Hospital, Barking, Havering and Redbridge University Hospitals NHS Trust; **Kapil Kapur**, **V Sathyanarayana**, Barnsley Hospital, Barnsley Hospital NHS Foundation Trust; **Yiannis Kallis**, Newham University Hospital, St Bartholemew's Hospital, The Royal London Hospital, Whipps Cross University Hospital, Barts Health NHS Trust; **Javaid Subhani**, Basildon University Hospital, Basildon and Thurrock University Hospitals NHS Foundation Trust; **Rory Harvey**, Bedford Hospital, Bedford Hospitals NHS Trust; **Roger McCorry**,

**Paul Rooney**, Royal Victoria Hospital, Belfast Health and Social Care Trust; **David Ramanaden**, Glan Clwyd Hospital, Betsi Cadwaladr University Health Board; **Richard Evans**, Llandudno General Hospital, Betsi Cadwaladr University Health Board; **Thiriloganathan Mathialahan**, Wrexham Maelor Hospital, Betsi Cadwaladr University Health Board; **Jaber Gasem**, Ysbyty Gwynedd, Betsi Cadwaladr University Health Board; **Christopher Shorrock**, Blackpool Victoria Hospital, Blackpool Teaching Hospitals NHS Foundation Trusts; **Mahesh Bhalme**, Royal Bolton Hospital, Bolton NHS Foundation Trust; **Paul Southern**, Bradford Royal Infirmary, Bradford Teaching Hospitals NHS Foundation Trust; **Jeremy A Tibble**, Royal Sussex County Hospital, Brighton and Sussex University Hospitals NHS Trust; **David A Gorard**, Amersham Hospital, Stoke Mandeville Hospital, Wycombe Hospital, Buckinghamshire Healthcare NHS Trust; **Susan Jones**, Calderdale Royal Hospital, Huddersfield Royal Infirmary, Calderdale and Huddersfield NHS trust; **George Mells**, **Victoria Mulcahy**, Addenbrooke's Hospital, Cambridge University Hospitals NHS Foundation Trust; **Brijesh Srivastava**, University Hospital Llandough, University Hospital of Wales, Cardiff and Vale University Health Board; **Matthew R Foxton**, Chelsea and Westminster Hospital, Chelsea & Westminster NHS Foundation Trust; **Carole E Collins**, West Middlesex University Hospital, Chelsea & Westminster NHS Foundation Trust; **David Elphick**, Chesterfield Royal Hospital, Chesterfield Royal Hospital NHS Foundation Trust; **Mazn Karmo**, Countess of Chester Hospital, Countess of Chester Hospital NHS Foundation Trust; **Francisco Porras-Perez**, Darlington Memorial Hospital, University Hospital of North Durham, County Durham and Darlington NHS Foundation Trust; **Michael Mendall**, Croydon University Hospital, Purley War Memorial Hospital, Croydon Health Services NHS Trust; **Tom Yapp**, Princess of Wales Hospital, Cwm Taf Morgannwg University Health Board; **Minesh Patel**, Prince

Charles Hospital, Royal Glamorgan Hospital, Ysbyty Cwm Cynon, Cwm Taf Morgannwg University Health Board; **Roland Ede**, Darent Valley Hospital, Queen Mary's Hospital Sidcup, Dartford And Gravesham NHS Trust; **Joanne Sayer**, Bassetlaw Hospital, Doncaster Royal Infirmary, Doncaster and Bassetlaw Hospitals NHS Foundation Trust; **James Jupp**, Dorset County Hospital, Dorset County Hospital NHS Foundation Trust; **Neil Fisher**, Russells Hall Hospital, Dudley Group of Hospitals NHS Trust; **Martyn J Carter**, Lister Hospital, Queen Elizabeth II Hospital, East and North Hertfordshire NHS Trust; **Konrad Koss**, Macclesfield District General Hospital, East Cheshire NHS Trust; **Jayshri Shah**, **Andrzej Piotrowicz**, **Glyn Scott**, Buckland Hospital, Kent and Canterbury Hospital, Queen Elizabeth The Queen Mother Hospital, William Harvey Hospital Ashford, East Kent Hospitals University NHS Foundation Trust; **Charles Grimley**, Burnley General Hospital, Royal Blackburn Hospital, East Lancashire Hospitals NHS Trust; **Ian R Gooding**, Colchester General Hospital, East Suffolk and North Essex NHS Foundation Trust; **Simon Williams**, Ipswich Hospital, East Suffolk and North Essex Foundation Trust; **Judith Tidbury**, Conquest Hospital, Eastbourne District General Hospital, East Sussex Healthcare NHS Trust; **Guan Lim**, Epsom General Hospital, Epsom and St Helier University Hospitals NHS Trust; **Kuldeep Cheent**, Frimley Park Hospital, Frimley Health NHS Foundation Trust; **Sass Levi**, Heatherwood Hospital, Wexham Park Hospital, Frimley Health NHS Foundation Trust; **Dina Mansour**, Queen Elizabeth Hospital, Gateshead Health NHS Foundation Trust; **Matilda Beckley**, George Eliot Hospital, George Eliot Hospital NHS Trust; **Coral Hollywood**, Cheltenham General Hospital, Gloucestershire Royal Hospital, Gloucestershire Hospitals NHS Foundation Trust; **Terry Wong**, **Richard Marley**, Guy's Hospital, St Thomas' Hospital, Guy's and St Thomas' NHS Foundation Trust; **John Ramage**, Basingstoke and North Hampshire Hospital, Hampshire Hospitals

Foundation trust; **Harriet M Gordon**, Royal Hampshire County Hospital, Hampshire Hospitals NHS Foundation Trust; **Jo Ridpath**, Harrogate District Hospital, Harrogate and District Foundation Trust; **Theodore Ngatchu**, Good Hope Hospital, Heartlands Hospital, Solihull Hospital, Heart of England NHS Foundation Trust; **Vijay Paul, Bob Grover**, Hillingdon Hospital, Hillingdon Hospitals NHS Foundation Trust; **Ray G Shidrawi**, Homerton University Hospital, Homerton University Hospital NHS Foundation Trust; **George Abouda**, **L Corless**, Castle Hill Hospital, Hull Royal Infirmary, Hull University Teaching Hospitals NHS Trust; **Mark Narain**, Bronglais Hospital, Hywel Dda University Health Board; **Ian Rees**, Prince Philip Hospital, Withybush General Hospital, Central Middlesex Hospital, Glangwili General Hospital, Hywel Dda University Health Board; **Ashley Brown**, Charing Cross Hospital, Hammersmith Hospital, St Mary's Hospital, Imperial College, Imperial College Healthcare NHS Trust; **Simon Taylor-Robinson**, St Mary's Hospital, Imperial College, Imperial College Healthcare NHS Trust; **Joy Wilkins**, **Leonie Grellier**, St Mary's Hospital, Isle of Wight, Isle of Wight NHS Trust; **Paul Banim**, James Paget University Hospital, James Paget University Hospitals NHS Foundation Trust; **Debasish Das**, Kettering General Hospital, Kettering General Hospital NHS Foundation Trust; **Michael A Heneghan**, King's College Hospital, King's College Hospital; **Howard Curtis**, Beckenham Beacon, Orpington Hospital, Princess Royal University Hospital, King's College Hospital NHS Foundation Trust; **Helen C Matthews**, Kingston Hospital, Kingston Hospital NHS Foundation Trust; **Faiyaz Mohammed**, Chorley and South Ribble Hospital, Royal Preston Hospital, Lancashire Teaching Hospitals NHS Foundation Trust; **Mark Aldersley**, St James's University Hospital, Leeds Teaching Hospitals NHS Trust; **Raj Srirajaskanthan**, **Giles Walker**, Lewisham Hospital, University Hospital Lewisham, Lewisham and Greenwich NHS

Trust; **Alistair McNair**, Queen Elizabeth Hospital, Lewisham and Greenwich NHS Trust; **Amar Sharif**, Central Middlesex Hospital, Northwick Park and St Mark's Hospitals, London North West Healthcare NHS Trust; **Sambit Sen**, Luton and Dunstable University Hospital, Luton and Dunstable University Hospital; **George Bird**, Maidstone Hospital, Tunbridge Wells Hospital, Maidstone and Tunbridge Wells NHS Trust; **Martin I Prince**, Manchester Royal Infirmary, Trafford General Hospital, Manchester University NHS Foundation Trust; **Geeta Prasad**, Wythenshawe Hospital, Manchester University NHS Foundation Trust; **Paul Kitchen**, **Adrian Barnardo**, Medway Maritime Hospital, Medway NHS Foundation Trust; **Chirag Oza**, Broomfield Hospital, St Peters Hospital, Mid-Essex Hospitals NHS Trust; **Nurani N Sivaramakrishnan**, Dewsbury and District Hospital, Mid Yorkshire Hospitals NHS Trust; **Prakash Gupta**, Milton Keynes Hospital, Milton Keynes Hospital NHS Foundation Trust; **Amir Shah**, University Hospital Crosshouse, NHS Ayrshire & Arran; **Chris DJ Evans**, Borders General Hospital, NHS Borders; **Subrata Saha**, Dumfries and Galloway Royal Infirmary, NHS Dumfries & Galloway; **Katharine Pollock**, Queen Margaret Hospital, Victoria Hospital, NHS Fife; **Peter Bramley**, Falkirk Community Hospital, Forth Valley Royal Hospital, Stirling Community Hospital, NHS Forth Valley; **Ashis Mukhopadhya**, Aberdeen Royal Infirmary, Dr Gray's Hospital, NHS Grampian; **Stephen T Barclay**, Gartnavel General Hospital, Glasgow Royal Infirmary, Inverclyde Royal Hospital, Royal Alexandra Hospital, Southern General Hospital, Victoria Infirmary, NHS Greater Glasgow and Clyde; **Natasha McDonald**, Hairmyres Hospital, Monklands Hospital, Wishaw General Hospital, NHS Lanarkshire; **Andrew J Bathgate**, Royal Infirmary of Edinburgh, St John's Hospital, NHS Lothian; **Kelvin Palmer**, Western General Hospital, NHS Lothian; **John F Dillon**, Ninewells Hospital, NHS Tayside; **Simon M Rushbrook**, Norfolk and Norwich University Hospital, Norfok

and Norwich University Hospitals NHS Foundation Trust; **Robert Przemioslo**, Southmead Hospital, North Bristol NHS Trust; **Chris McDonald**, Cumberland Infirmary, West Cumberland Hospital, North Cumbria University Hospitals NHS Foundation Trust; **Andrew Millar**, **Cheh Tai**, North Middlesex Hospital, North Middlesex University Hospital NHS Trust; **Stephen Mitchell**, **Jane Metcalf**, University Hospital of Hartlepool, University Hospital of North Tees, North Tees and Hartlepool NHS Foundation Trust; **Syed Shaukat**, Hinchingbrooke Hospital, North West Anglia NHS Trust; **Mary Ninkovic**, Peterborough City Hospital, Stamford & Rutland Hospital, North West Anglia NHS Foundation Trust; **Udi Shmueli**, Northampton General Hospital, Northampton General Hospital NHS Trust; **Andrew Davis**, North Devon District Hospital, Northern Devon Healthcare NHS Trust; **Asifabbas Naqvi**, Diana Princess of Wales Hospital, Scunthorpe General Hospital, Northern Lincolnshire and Goole NHS Foundation Trust; **Tom JW Lee**, North Tyneside General Hospital, Northumbria Healthcare NHS Foundation Trust; **Stephen Ryder**, Nottingham City Hospital, Queen's Medical Centre, NIHR Nottingham Biomedical Research Centre at Nottingham University Hospitals NHS Trust; **Jane Collier**, John Radcliffe Hospital, Oxford University Hospitals NHS Trust; **Howard Klass**, Fairfield General Hospital, North Manchester General Hospital, Rochdale Infirmary, The Royal Oldham Hospital, Pennine Acute Hospitals NHS Trust; **Matthew E Cramp**, Derriford Hospital, University Hospitals Plymouth NHS Trust; **Nichols Sharer**, Poole Hospital, Poole Hospital NHS Foundation Trust; **Richard Aspinall**, Queen Alexandra Hospital, Portsmouth Hospitals NHS Trust; **Deb Ghosh**, St Margaret's Hospital, The Princess Alexandra Hospital, Princess Alexandra Hospital NHS Trust; **Andrew C Douds**, The Queen Elizabeth Hospital King's Lynn, King's Lynn NHS Foundation Trust; **Jonathan Booth**, Royal Berkshire Hospital, Royal Berkshire NHS Foundation Trust; **Earl Williams**,

Royal Bournemouth Hospital, Royal Bournemouth and Christchurch Hospitals NHS Foundation Trust; **Hyder Hussaini**, Royal Cornwall Hospital, Royal Cornwall Hospitals NHS Trust; **John Christie**, Royal Devon and Exeter Hospital, Royal Devon And Exeter Nhs Foundation Trust; **Steven Mann**, Barnet and Chase Farm Hospitals, Royal Free London NHS Trust; **Douglas Thorburn**, **Aileen Marshall**, The Royal Free Hospital, Royal Free London NHS Foundation Trust; **Imran Patanwala**, Royal Liverpool University Hospital, Royal Liverpool and Broadgreen University Hospitals NHS Trust; **Aftab Ala**, Royal Surrey County Hospital, Royal Surrey County Hospital NHS Foundation Trust; **Julia Maltby**, Royal United Bath Hospital, Royal United Hospitals Bath NHS Foundation Trust; **Ray Matthew**, Cannock Chase Hospital, Royal Wolverhampton Hospitals NHS Trust; **Chris Corbett**, New Cross Hospital, Royal Wolverhampton NHS Trust; **Sam Vyas**, Salisbury District Hospital, Salisbury NHS Foundation Trust; **Saket Singhal**, Sandwell General Hospital, Sandwell & West Birmingham Hospitals NHS Trust; **Dermot Gleeson**, Northern General Hospital, Royal Hallamshire Hospital, Sheffield Teaching Hospitals NHS Foundation Trust; **Sharat Misra**, King's Mill Hospital, Newark Hospital, Sherwood Forest Hospitals NHS Foundation Trust; **Jeff Butterworth**, Princess Royal Hospital, Royal Shrewsbury Hospital, Shrewsbury and Telford Hospital NHS Trust; **Keith George**, Torbay Hospital, Torbay and South Devon NHS Foundation Trust; **Tim Harding**, Lagan Valley Hospital, Ulster Hospital, South Eastern Health and Social Care Trust; **Andrew Douglass**, Friarage Hospital, The James Cook University Hospital, South Tees Hospitals NHS Foundation Trust; **Harriet Mitchison**, Sunderland Royal Hospital, Sunderland Royal Hospital, South Tyneside and Sunderland NHS Foundation Trust; **Simon Panter**, South Tyneside District Hospital, South Tyneside and Sunderland NHS Foundation Trust; **Jeremy Shearman**, Warwick Hospital, South Warwickshire NHS Foundation

Trust; **Gary Bray**, Southend University Hospital, Southend University Hospital NHS Foundation Trust; **Michael Roberts**, **Graham Butcher**, Ormskirk District General Hospital, Southport and Formby District General Hospital, Southport & Ormskirk Hospital NHS Trust; **Daniel Forton**, St George's Hospital, St George’s University Hospitals NHS Foundation Trust; **Zahid Mahmood**, Stepping Hill Hospital, Stockport NHS Foundation Trust; **Matthew Cowan**, **Debashis Das**, East Surrey Hospital, Surrey and Sussex Healthcare NHS Trust; **Chin Lye Ch'ng**, Morriston Hospital, Singleton Hospital, Swansea Bay University Health Board; **Mesbah Rahman**, Neath Port Talbot Hospital, Swansea Bay University Health Board; **Gregory C A Whatley**, Tameside General Hospital, Tameside and Glossop integrated care NHS Trust; **Emma Wesley**, Musgrove Park Hospital, Taunton and Somerset NHS Foundation Trust; **Aditya Mandal**, Lincoln County Hospital, United Lincolnshire Hospitals NHS Trust; **Sanjiv Jain**, Pilgrim Hospital Boston, United Lincolnshire Hospitals NHS Trust; **Stephen P Pereira**, University College Hospital, University College London Hospitals NHS Foundation Trust; **Mark Wright**, Southampton General Hospital, University Hospital Southampton NHS Foundation Trust; **Palak Trivedi**, The Queen Elizabeth Hospital, University Hospitals Birmingham NHS Foundation Trust; **Fiona H Gordon**, Bristol Royal Infirmary, University Hospitals Bristol NHS Foundation Trust; **Esther Unitt**, University Hospital, Coventry, University Hospital Coventry and Warwickshire NHS Trust; **Altaf Palejwala**, Queen's Hospital, University Hospitals of Derby and Burton NHS Foundation Trust; **Andrew Austin**, Royal Derby Hospital, University Hospitals of Derby and Burton NHS Foundation Trust; **Vishwaraj Vemala**, **Allister Grant**, Glenfield Hospital, Leicester General Hospital, Leicester Royal Infirmary, University Hospitals of Leicester NHS Trust; **Andrew D Higham**, Royal Lancaster Infirmary, University Hospitals of Morecambe Bay NHS Foundation Trust; **Alison**

**Brind**, Royal Stoke University Hospital, University Hospitals of North Midlands NHS Trust; **Ray Mathew**, County Hospital, University Hospitals of North Midlands NHS Trust; **Mark Cox**, Walsall Manor Hospital, Walsall Healthcare NHS Trust; **Subramaniam Ramakrishnan**, Warrington Hospital, Warrington and Halton Hospitals NHS Foundation Trust; **Alistair King**, Hemel Hempstead General Hospital, St Albans City Hospital, Watford General Hospital, West Hertfordshire Hospitals NHS Trust; **Simon Whalley**, West Suffolk Hospital, West Suffolk NHS Foundation Trust; **Jocelyn Fraser**, St Richard's Hospital, Western Sussex Hospitals NHS Foundation Trust; **SJ Thomson**, Worthing Hospital, Western Sussex Hospitals NHS Foundation Trust; **Andrew Bell**, Weston General Hospital, Weston Area Health Trust; **Voi Shim Wong**, The Whittington Hospital, Whittington Hospital NHS Trust; **Richard Kia**, Arrowe Park Hospital, Victoria Central Hospital, Wirral University Teaching Hospital NHS Foundation Trust; **Ian Gee**, Alexandra Hospital, Kidderminster Hospital and Treatment Centre, Worcestershire Royal Hospital, Worcestershire Acute Hospitals NHS trust; **Richard Keld**, Royal Albert Edward Infirmary, Wrightington, Wigan And Leigh NHS Trust; **Rupert Ransford**, The County Hospital, Wye Valley NHS Trust; **James Gotto**, Yeovil District Hospital, Yeovil District Hospital NHS Foundation Trust; **Charles Millson**, Bridlington Hospital, Scarborough Hospital, The York Hospital, York Teaching Hospitals NHS Foundation Trust.

*US PBC Consortium*

**Brian D. Juran**, Division of Gastroenterology and Hepatology, Mayo Clinic, Rochester, Minnesota; **Elizabeth J. Atkinson**, Division of Biomedical Statistics and Informatics Mayo Clinic, Rochester, Minnesota; **Angela Cheung**, Division of Gastroenterology and Hepatology, Mayo Clinic, Rochester, Minnesota; **Mariza de**

**Andrade**, Division of Biomedical Statistics and Informatics Mayo Clinic, Rochester, Minnesota; **Konstantinos N. Lazaridis**, Division of Gastroenterology and Hepatology, Mayo Clinic, Rochester, Minnesota; **Naga Chalasani**, Indiana University, Indiana; **Vel Luketic**, Virginia Commonwealth University, Virginia; **Joseph Odin**, Icahn School of Medicine, Mount Sinai, New York; **Kapil Chopra**, University of Pittsburgh; **Aris Baras**, **Julie Horowitz**, **Goncalo Abecasis**, **Michael Cantor**, **Giovanni Coppola**, **Aris Economides**, **Luca A. Lotta**, **John D. Overton**, **Jeffrey G. Reid**, **Alan Shuldiner**, **Christina Beechert**, **Caitlin Forsythe**, **Erin D. Fuller**, **Zhenhua Gu**, **Michael Lattari**, **Alexander Lopez**, **John D. Overton**, **Thomas D. Schleicher**, **Maria Sotiropoulos Padilla**, **Karina Toledo**, **Louis Widom**, **Sarah E. Wolf**, **Manasi Pradhan**, **Kia Manoochehri**, **Ricardo H. Ulloa**, **Xiaodong Bai**, **Suganthi Balasubramanian**, **Leland Barnard**, **Andrew Blumenfeld**, **Gisu Eom**, **Lukas Habegger**, **Alicia Hawes**, **Shareef Khalid**, **Jeffrey G. Reid**, **Evan K. Maxwell**, **William Salerno**, **Jeffrey C. Staples**, **Marcus B. Jones**, **Lyndon J. Mitnaul**, Regeneron.

## Supplementary Materials and Methods

*Participants and genotyping*

In all cohorts, primary biliary cholangitis (PBC) was defined by the criteria of the European Association for the Study of the Liver (EASL) [1]. The **'old' Italian panel** was that of Liu et al. (2010) [2], in which cases were self-reported whites of Italian descent, genotyped using the Illumina Human610-Quad BeadChip, and controls were healthy Italians genotyped using the Illumina 1M-Duo BeadChip. In the current study, following imputation and quality control (QC) procedures, the old Italian panel comprised 13,113,694 variants across 444 cases and 901 controls. The **Wellcome Trust Case Control Consortium 3 (WTCCC3) panel** was that of Mells et al. (2011) [3], in which cases were self-reported whites of British descent, genotyped using the Illumina Human-660 W Quad BeadChip, and controls were UK population controls genotyped on the Illumina 1M-Duo BeadChip as part of the WTCCC2 project. In the current study, following imputation and QC, the WTCCC3 panel comprised 12,881,032 variants across 1,816 cases and 5,155 controls. The **Japanese panel** was that of Kawashima et al. (2017) [4], consisting of Japanese cases and controls genotyped using the Affymetrix Axiom Genome-Wide ASI BeadChip. In the current study, following imputation and QC, the Japanese panel comprised 7,308,269 variants across 1,377 cases and 1,495 controls. The **Chinese panel** consisted of cases from the study by Qiu et al. (2017) [5] and newly genotyped, geographically matched population controls, all self-reported Han Chinese, genotyped using the HumanOmniZhongHua-8 BeadChip. In the current study, following imputation and QC, the Chinese panel comprised 6,934,908 variants across 1,118 cases and 2,788 controls.

In the **'new' Canadian-UK panel**, cases included self-reported whites of British descent, recruited throughout the UK via the UK-PBC Consortium, and self-reported white Canadians of European descent. Genotyping of cases was undertaken using the Illumina HumanCoreExome BeadChip at the Wellcome Sanger Institute, as part of the UK-PBC project (www.uk-pbc.com, Research Ethics Committee reference 07/H0606/96). Control genotype data were obtained with permission from the UK Household Longitudinal Study (https://www.understandingsociety.ac.uk/) [6], in which UK population controls were genotyped using the Illumina HumanCoreExome BeadChip. Following imputation and QC, the new Canadian-UK panel comprised 8,656,760 variants across 4,615 cases and 9,233 controls. In the **'new' Italian panel**, cases and controls were self-reported whites of Italian descent, genotyped using the Illumina HumanCoreExome BeadChip at the Wellcome Sanger Institute, as part of UK-PBC. Following imputation and QC, the new Italian panel comprised 9,264,788 variants across 255 cases and 579 controls. In the **'new' US panel**, cases and controls were self-reported whites of European descent, genotyped using the **Illumina Infinium Global Screening Array (GSA) v1**. Following imputation and QC, the new US panel comprised 9,964,354 variants across 891 cases and 621 controls.

*Identification of genome-wide significant loci and lead SNPs*

Following meta-analysis of the European panels, Asian panels, and all panels combined, we used the SNP2GENE function of FUMA GWAS [7] to select the lead SNPs in each genome-wide significant locus (which basically corresponds to choosing the top [smallest P-value] independent SNPs in a locus, with independence within and between each locus being defined by FUMA based on default LD thresholds). This procedure was carried out for the European, Asian, and combined results separately.

We then sorted these lead SNPs from the three separate analyses by chromosome and BP position, and manually defined lead SNPs from the three different analyses that lay within 400 kb of one another as corresponding to the same locus. This resulted in 56 separate loci as listed in **Suppl. Table 1**. For loci where one or other cohort did not have a FUMA lead SNP represented (on account of not having reached genome-wide significance), we chose as the lead SNP the most significant SNP lying within 600 kb of the top (smallest P-value) SNP at that locus identified from the cohort(s) that were represented. Although, formally, this allows a separation between Asian and European lead SNPs of up to 600 kb, perusal of **Suppl. Table 1** (specifically the "Position" column) indicates that, for most loci, the lead Asian variant lies considerably closer than 600 kb to the lead European variant.

*Comparison of results with and without genomic control or GMMAT-derived correction*

We used a conservative "double" genomic control (GC) approach (see **Main Text**) to avoid incurring any inflation in our test statistics due to population stratification. This use of "double" GC might be considered overly conservative, given that part of the inflation could be due to polygenicity. We explored this for the two cohorts showing the largest inflation (Canadian-UK panel, $\lambda$=1.091; Chinese panel, $\lambda$ = 1.050) by using LD score regression [8] on the original (uncorrected) summary statistics, making use of the 1000 Genomes European and East Asian LD scores respectively provided with the software. Examination of the LD score regression intercepts (1.0272 for the Canadian-UK panel and 1.0159 for the Chinese panel) indicated that only a small proportion of the inflation is attributed to sources other than polygenic heritability. One could argue that this suggests there is, in fact, minimal unexplained inflation in our results. We therefore compared our original (GC- or GMMAT-corrected) results to

those obtained when using no GC (or GMMAT-derived) correction at all. As expected, with no correction, all loci previously identified as genome-wide significant reach slightly higher levels of significance, while a few additional loci not previously identified now (just) reach genome-wide significance (**Suppl. Figure 5**, **Suppl. Table 5**).

*Comparison of results with those obtained from MR-MEGA*

We also compared our results from all panels combined with those obtained using trans-ethnic meta-regression analysis as implemented in MR-MEGA [9]. MR-MEGA (Meta-Regression of Multi-Ethnic Genetic Association) is a tool to detect and fine-map complex trait association signals via trans-ethnic meta-regression. This approach uses genome-wide metrics of diversity between populations to derive axes of genetic variation via multi-dimensional scaling. Allelic effects of a variant across the contributing cohorts, weighted by their corresponding standard errors, are then modelled in a linear regression framework, including the axes of genetic variation as covariates. The flexibility of this model enables partitioning of the heterogeneity into components due to ancestry and residual variation, which is expected to improve fine-mapping resolution.

*Permutation procedure to evaluate consistency of association signals across populations*

For non-HLA loci, we carried out a permutation procedure to investigate the extent to which a strong association signal in one set of panels (usually the European) was supported by a nearby signal in the other set of panels (usually the Asian). Analysis was carried out at 26 loci (23 representing Asian confirmation of a European signal; three representing European confirmation of an Asian signal) where a suggestive

association signal ($P < 2\times10^{-4}$) was seen in the confirmatory panels within 600 kb of a strong signal in the primary panels.  The remaining loci were not interrogated in this way owing to futility (the level of association seen in the confirmatory panels was considered too weak to have any chance of generating a significant permutation P-value).  Focusing on the 23 newly identified (at genome-wide levels of significance) loci, we conservatively considered a newly identified finding to be corroborated if we obtained permutation $P < 0.00217$ (corresponding to $P < 0.05$ Bonferroni-corrected for 23 tests).  A full Bonferroni correction for all 56 genome-wide significant loci (including previously known loci) would lower the required threshold to $P < 0.000893$.

For European confirmation of an Asian signal, we merged the individual-level genotype data of the five European panels, randomly permuted case/control status 10,000 times, and carried out association testing within the vicinity of the primary Asian signal to determine in how many of the 10,000 replicates was an association signal as strong or stronger than that seen in the real data obtained, within the same degree of proximity to the primary Asian signal as seen in the real data.  We defined the permutation P-value as the proportion of replicates in which such a (false positive) association occurred.

For the Asian cohorts (for which individual-level Chinese genotype data was unavailable), we used the individual-level Japanese data to mimic a combined Chinese/Japanese panel by randomly selecting 1118 of the 1377 Japanese cases to be included twice (once as Japanese cases and once as Chinese cases) and doubling the 1495 Japanese controls and then randomly selecting 2788 of these to be used as Chinese controls (in addition to the 1495 used as genuine Japanese controls).  This generates a combined Chinese/Japanese panel with higher relatedness (and of purely Japanese ancestry) than is exhibited by the real data, but we did not consider that this

would have a substantial influence on the null distribution of test statistics generated by the permutation procedure. We then repeated the same permutation procedure as used for Europeans, to determine the chance of finding an association signal at least as strong as that seen in the real data, within the same degree of proximity to the primary European signal as was seen in the real data. At one locus, 14q13.2, rather than assessing the significance of the top Asian SNP (rs199892962, Asian P-value $4.36 \times 10^{-6}$) in this way, we instead assessed the significance of the Asian signal at the top combined SNP (rs799469, Asian P-value $5.14 \times 10^{-5}$) on account of the closer proximity of the top combined SNP to the top European SNP.

*Fine-mapping and construction of credible sets*

We used FINEMAP [10] version 1.4 and conditional and joint analysis (COJO) [11] implemented within GCTA [12] (specifically the "--cojo-slct" option, which performs a stepwise model selection procedure to select independently associated SNPs) to investigate the likely number of separate signals (due to separate causal variants) at each of the 56 genome-wide significant risk loci identified. We also used FINEMAP to construct credible sets of variants that together account for 95% of the posterior probability for each causal variant. Analyses were carried out for all 56 loci using the European summary statistics, and for 13 selected loci (those achieving genome-wide significance $P < 5 \times 10^{-8}$ within the Asian panels) using the Asian summary statistics, with minor allele frequencies and pairwise LD estimated from the input data (the combined European panels or the Japanese panel, respectively). We used the "--sss" option within FINEMAP to specify the "Fine-mapping with shotgun stochastic search" algorithm, the "--group-snps" option (with default settings) to group SNPs based on their correlations, and the "--n-causal-snps 3" option to set the maximum number of

causal variants allowed within a locus to 3. For prioritization of candidate genes (see below and **Main Text**), we focussed on the "first credible variants" at non-HLA loci generated by the European FINEMAP analyses. Most of these "first credible variants" came from any of the tested configurations that met the threshold to be output by FINEMAP (i.e. from configurations allowing only one variant, from configurations allowing 2 variants, or from configurations allowing 3 variants), but, in a few cases, not all configurations were included, specifically when the configuration(s) with the lowest posterior probability did not show (on the basis of visual inspection) good localisation of potential causal variants.

*Estimation of trait variance explained in the Japanese cohort by subsets of European implicated SNPs*

We used the Japanese cohort (for which we had individual-level data) to calculate the proportion of trait variance (on the liability scale) explained in this cohort by various subsets of SNPs that were chosen according to their significance (P-value thresholds 0.1, 0.01, 0.001, $1\times10^{-4}$, $1\times10^{-5}$) in the European GWMA. For each chosen threshold, the resulting SNPs were pruned based on their LD in the Japanese cohort (using the "--indep 50 5 2" command in PLINK [13]), to retain only SNPs that were approximately independent of all other SNPs. The trait variance explained by these SNPs was estimated by constructing a genetic relationship matrix and carrying out restricted maximum likelihood analysis using the "--make-grm-bin" and "--reml" options in GCTA, with prevalence assumed (as in Lui et al. 2012 [14]) to be 0.0004; we also investigated the sensitivity of the results to different choices of the prevalence. For comparison, we also estimated the proportion of trait variance (on the liability scale) explained by an

equal number of randomly chosen SNPs, again using the “--make-grm-bin” and “--reml” options in GCTA (**Suppl. Table 6**).

*Functional annotation and mapping of credible causal variants using FUMA GWAS*

We used the SNP2GENE function of FUMA GWAS for functional annotation and mapping of the first credible set of credible causal variants at each risk locus. We selected the 1000G Phase3 EUR reference panel population for annotation of credible causal variants at risk loci identified in the European panels, and the 1000G Phase3 EAS reference panel population for annotation of credible causal variants at risk loci identified in the Asian panels. We mapped variants to: (1) any gene type located within 10kb of the variant; (2) expression quantitative trait loci (eQTLs) in any available tissue type, defined by an association between genotype and gene expression significant at P $<1\times10^{-3}$; (3) chromatin interacting regions in any available dataset, defined by significant (FDR $<1\times10^{-6}$) interaction between a region containing the variant and a region from 250 bp upstream to 500 bp downstream of the transcription start site; and (4) and enhancer/promoter regions defined by Roadmap 111 epigenomes.

*TWAS/MWAS/PWAS based on predicted gene expression/methylation/protein levels*

Functional annotation and mapping of credible causal variants suggested that the genetic architecture of PBC might confer risk of disease by influencing the regulation of expression of nearby or distant genes. We therefore sought to evaluate how, at the genome-wide (or ‘omic) level, the genetic architecture of PBC might influence DNA methylation or gene expression. Thus, we used MetaXcan [15], summary statistics from our GWMA of the European panels, and reference panels from the Genotype-Tissue Expression (GTEx) project [16], Avon Longitudinal Study of Parents and

Children (ALSPAC) [17, 18], and the INTERVAL study [19] to derive genome-wide genetic prediction models of gene expression, DNA methylation, and serum protein levels, respectively, in cases and controls. We used these models to correlate predicted gene expression, DNA methylation, and serum protein levels with disease status, thus performing transcriptome-wide, methylome-wide and serum proteome-wide association studies (TWAS, MWAS and PWAS, respectively).

To predict gene expression, we used pre-existing models in diverse tissues trained by the developers of the MetaXcan package, available to download from the predictdb repository (http://predictdb.org/). Only prediction models showing squared correlation between predicted and observed expression $R^2$ ≥0.01 were available to download, resulting in gene expression prediction models for 25,314 genes.

To predict CpG methylation, we used data from ALSPAC to train our own DNA methylation prediction models. ALSPAC is a longitudinal study that recruited pregnant women resident in Avon, UK with expected dates of delivery 1st April 1991 to 31st December 1992. The initial number of pregnancies enrolled was 14,541. Of these, 674 pregnancies were excluded (due to non-live birth or unknown outcomes), resulting in 13,867 pregnancies from 13,761 individual women (see figure 1 of Fraser et al., 2013 [18]). The 855 mothers with ARIES data come from this initial enrolled sample of 13,761 mothers. Full details of the study are given in (Boyd et al., 2013 [17]; Fraser et al., 2013 [18]). Ethical approval for ALSPAC was obtained from the ALSPAC Ethics and Law Committee and the Local Research Ethics Committees; consent for biological samples has been collected in accordance with the Human Tissue Act (2004); and informed consent for the use of data collected via questionnaires and clinics was obtained from participants following the recommendations of the ALSPAC Ethics and Law Committee at the time. In ALSPAC, study data were collected and managed

using REDCap electronic data capture tools [20, 21], hosted at the University of Bristol. The ALSPAC website contains details of all the data that are available through a fully searchable data dictionary and variable search tool at http://www.bristol.ac.uk/alspac/researchers/our-data/.

In ALSPAC, DNA methylation in whole blood was measured using the 450K chip. In total, we had normalised methylation levels for ~400,000 CpG sites, adjusted for age and white blood cell proportions. The model training procedure involved (1) splitting the data into a training and test set; (2) identifying elastic net model parameters by performing 10-fold cross-validation on the training set and regressing CpG methylation on genotypes at local SNPs using elastic net with a range of parameter values considered; (3) training the model on the full training set by regressing CpG methylation on genotypes at local SNPs using elastic net with the parameters that had provided the best prediction performance in cross-validation; and (4) applying the model to the test set and calculating the correlation between predicted and observed methylation (r). The CpG methylation prediction models showing r≥0.1 were then applied to the European PBC GWMA summary statistics using the MetaXcan approach.

To predict protein levels, we used data from the INTERVAL study to train our own prediction models. The serum proteome was profiled using the SOMAscan platform. We had normalised protein levels for 3283 proteins, adjusted for age, sex, batch effects and top 3 genotype PCs. For each protein, 10-fold nested CV was performed to tune elastic net model parameters and assess prediction accuracy. 520 prediction models showing r≥0.1 were taken forward. For these 520 proteins, we trained a model using all INTERVAL study samples and took these models forward for

PWAS (applied to the European PBC GWMA summary statistics using the MetaXcan approach).

*Co-localisation of signals using moloc*

We used the moloc [22] approach to identify whether the genetic associations for the multiple phenotypes considered (PBC, gene expression, CpG methylation, and serum protein levels) co-localised to the same causal SNP, based on summary statistics (betas and SEs) from our GWMA of the European panels and the eQTL/mQTL/pQTL studies (GTEx, ARIES, INTERVAL) mentioned above. We defined co-localisation as a posterior probability ≥0.8 for a given hypothesis of pairwise, three-way, or four-way co-localisation for the SNP genotype with disease trait (G), gene expression (E), CpG methylation (M) and protein levels (P).

*Pathway analysis*

We trimmed out the SNPs in LD with top variants at P $<1\times10^{-2}$ (using "--clump-r2 0.1" and "--clump-kb 1000" commands in PLINK) comprising 3,495 independent SNPs (1,388 genes). We performed the pathway enrichment analysis such as KEGG pathway and Reactome pathway with FDR <0.2 through DAVID database [23].

*Network-based in silico drug efficacy screening*

We employed the approach of Guney et al. (2016) [24] in which candidate genes for a disease and known drug targets are used to estimate a drug-disease proximity measure, z, that quantifies the closeness of the connection between the candidate genes and the drug targets, correcting for the known biases of the interactome. This

approach was used to calculate the proximity between each drug in DrugBank (accessed January 2021) and our set of prioritized candidate genes for PBC.

The starting point for the analysis is the protein-protein interaction (PPI) network compiled by Menche et al. (2015) [25]. This network is used to construct a 'distance' between any set of drug target genes/proteins (T) and your own set of genes (S) of interest. Various distance measures could be considered; we used the (non-symmetric) measure $d_c$ defined as the average shortest path length between the drug's targets and the nearest disease gene, recognising that Guney et al. (2016) had found $d_c$ to give the best discrimination (AUC = 66%) between known and unknown drug-disease pairs. The relative proximity between the two sets of genes is then defined as $z = (d_c-\mu)/\sigma$ where μ and σ are calculated via a randomisation procedure. Specifically, two groups of genes are randomly selected to match T and S in size and degree (binned within degree intervals, so there are at least 100 genes in each bin, to avoid repeatedly choosing the same small number of high degree nodes during the degree-preserving random selection). The procedure is repeated 1000 times to give an empirical mean (μ) and standard deviation (σ) for $d_c$. Guney et al. (2016) define a drug to be proximal to a disease if its proximity satisfies $z \leq -0.15$, and distant otherwise. For more details, see Guney et al. (2016).

**This page is intentionally blank.**

**Supplementary Figures**

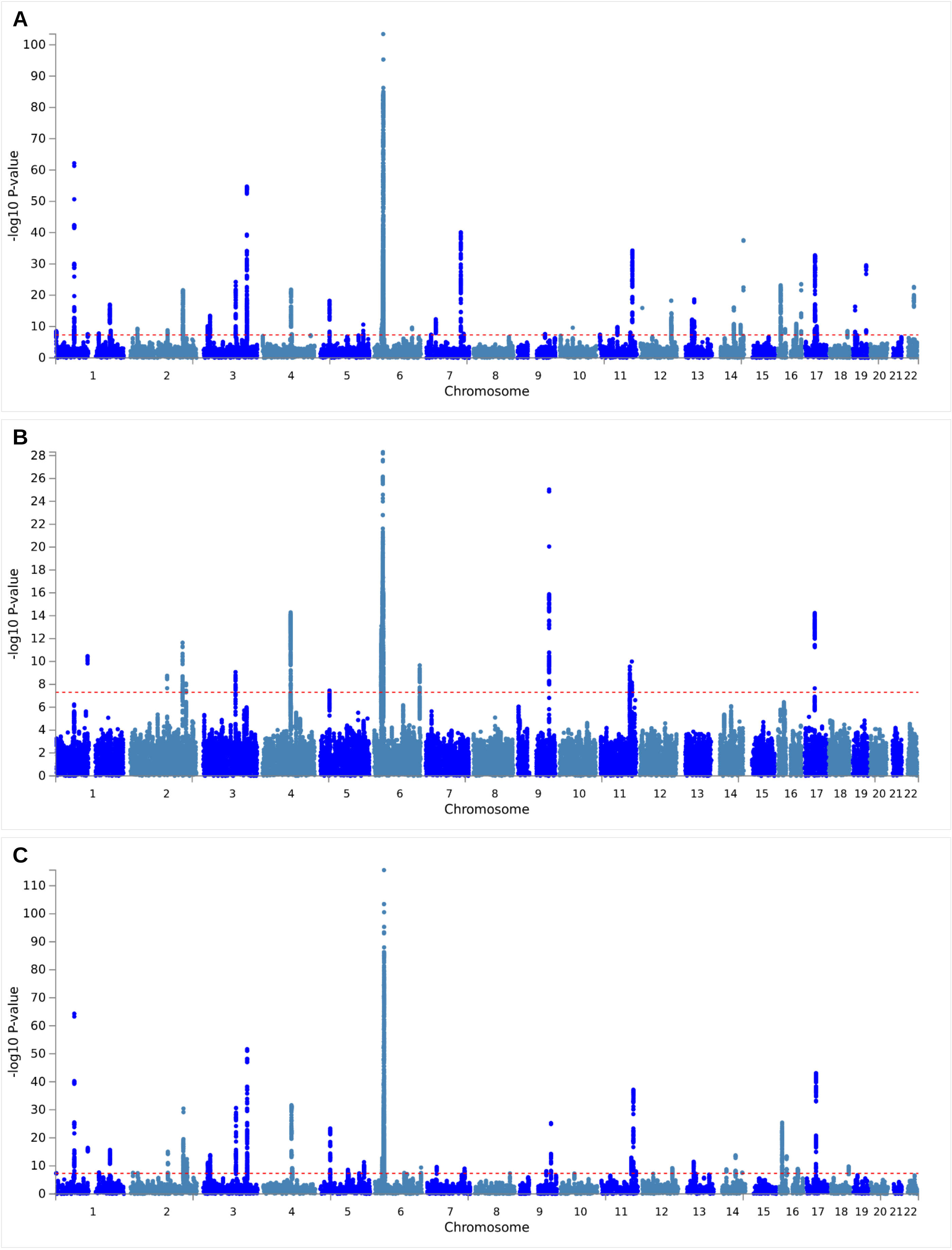


**Fig. S1: Manhattan plots from genome-wide meta-analysis of primary biliary cholangitis**. (A) European panels, with 46 genome-wide significant loci. (B) Asian panels, with 13 genome-wide significant risk loci; and (C) all panels combined, with 41 genome-wide significant risk loci.

**Fig. S2: LocusZoom plots of known and newly identified PBC risk loci reaching genome-wide significance in the current study.**

Each LocusZoom plot below shows: (1) for each variant across the risk locus, the variant's negative $\log_{10}$ P-value versus its base pair position (coloured shapes); and (2) the recombination rate at each point across the risk locus (blue solid line). The designated index variant is shown as a purple diamond while other variants are shown as circles coloured according to their known linkage disequilibrium (LD) with the index variant in the relevant population (1000G EUR or ASN). Above the figure, the **Plotted SNPs** track shows the position of each variant and demonstrates the density of coverage of the risk locus in the study. Genes and their position across the risk locus are shown below the figure. Note that the coordinates of variants and genes are based on Genome Reference Consortium Human Build 37 (GRCh37).

The LocusZoom plots demonstrate that the association signal was stronger in the European compared to the Asian panels at 49 risk loci. At 20 of these loci, association nevertheless reached genome-wide (2q21.3, 2q32.2, 3q13.33, 4q24 [1], 5p13.2, 6p21.32, 11q23.3, 17q12) or suggestive (1p31.3, 2p25.1, 3q25.33, 4q24 [2], 5q21.1, 5q33.3, 14q13.2, 14q32.12, 14q32.32, 16p13.13, 16p12.1, 16q24.1) significance in the Asian panels; and permutation testing suggested that the Asian signal corroborated the European one, albeit in some instances not at a Bonferroni-corrected level of significance. Fixed effect meta-analysis identified significant heterogeneity ($P_{het\ meta}$ <0.05) in the lnORs of one or other lead variant at 2q21.3, 2q32.2, 3q25.33, 4q24 [1], 5q21.1, and 11q23.3. At 2q32.2 (harbouring STAT4), 3q25.33 (IL12A), and 11q23.3 (CXCR5), this might reflect the presence of more than one independent association (**Suppl. Figures 2.10**, **2.15** and **2.37**, respectively) – but at the other risk

loci listed above, the most plausible explanation is different patterns of LD in European versus Asian populations.

At the remaining 29 risk loci, association reached genome-wide or suggestive significance in the European panels – but only nominal (1q23.1, 1q31.3, 1q32.1, 2p23.3, 3p24.3, 3p24.2, 5q31.3, 6q23.3, 7p21.1, 7p14.1, 7q32.1, 7q34, 8q24.21, 9q22.33, 10q11.23, 11q13.1, 12p13.31, 12q24.12, 13q14.11, 13q14.2, 14q24.1, 16q22.1, 18q22.2, 19p13.2, 19q13.33, 22q13.1) or not even nominal (1p36.32, 11p15.5, 17q21.31) significance in the Asian panels. This may be explained by the small effect size of variants at these loci, with median log odds ratio (lnOR) = 0.15 in the European panels. Permutation testing was not applied owing to futility. Fixed effect meta-analysis did not identify significant heterogeneity in the lnORs of lead variants at most of these loci – but given the lack of validation in the Asian panels, we attach little meaning to this observation.

The association signal was stronger in the Asian compared to the European panels at seven risk loci. At all seven, association was nevertheless evident in the European panels at genome-wide (1p13.1, 11q23.1) or suggestive (2q33.2, 6q21, 6q27, 9q32, 11q24.3) significance; and permutation testing confirmed that the European signal corroborated the Asian one, or vice versa ($P_{permutation} <0.0001$). Fixed effect meta-analysis of the lnORs from the European and Asian panels identified significant heterogeneity in the lnORs of rs4134466 at 6q21, rs968334 at 6q27, rs4938534 at 11q23.1, and rs11430718 at 11q24.3. At 11q23.1 (harbouring POU2AF1), this might reflect genuine variation in effect size (**Suppl. Figure 2.36**), whilst at 6q21, 6q27, and 11q24.3 (**Suppl. Figures 2.23, 2.25**, and **2.38, respectively**), the most plausible explanation is different patterns of LD in European versus Asian populations.

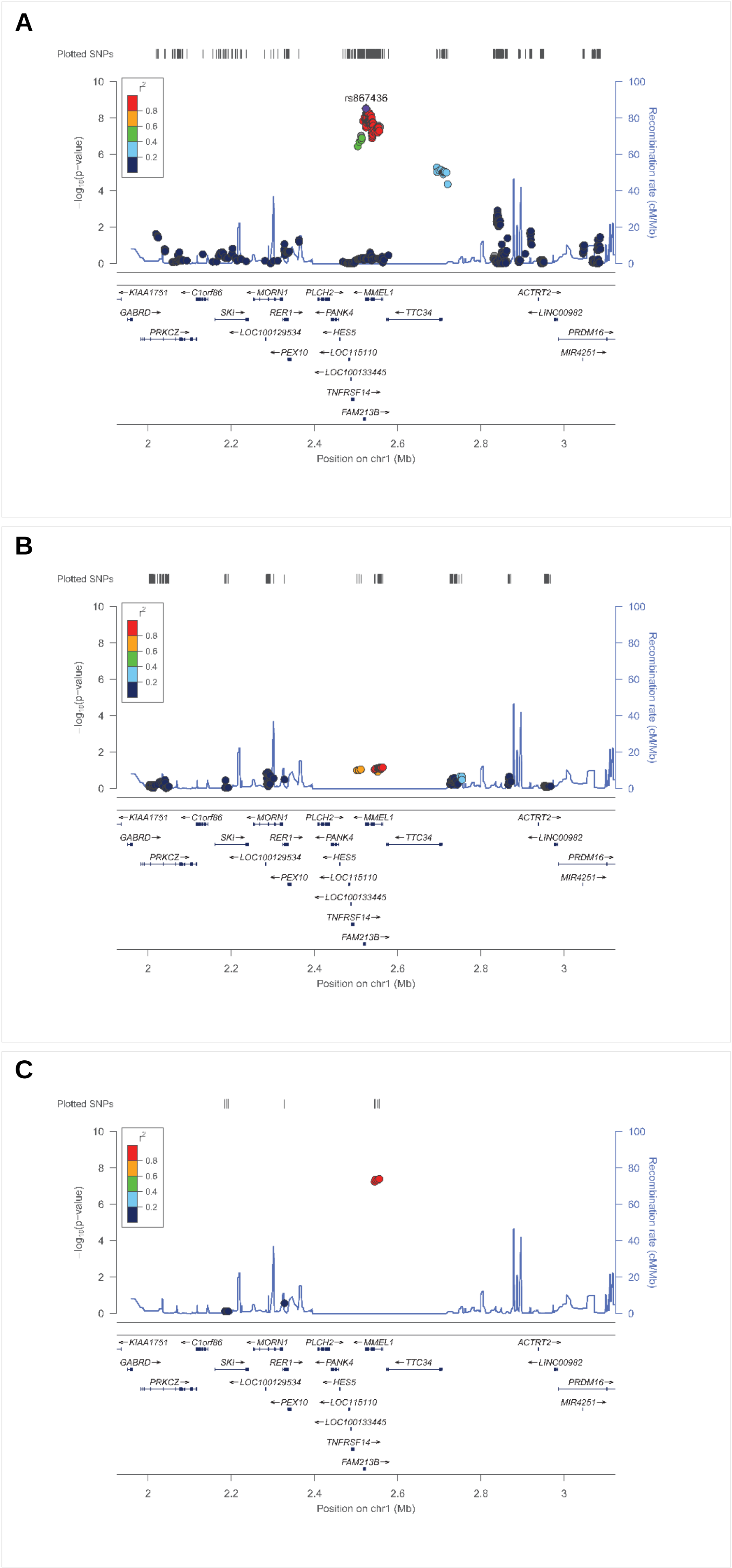


**Fig. S2.1: LocusZoom plots of the known PBC risk locus 1p36.32.** (A) European panels, (B) Asian panels, and (C) all panels combined. Association at this locus reached genome-wide significance in the European panels (rs867436 at 1:2,523,723; $P = 2.99\times10^{-9}$); not even nominal significance in the Asian panels (rs6422657 at 1:2,557,531; $P = 0.069$); and genome-wide significance in all panels combined (rs28568531 at 1:2,555,640; $P = 4.06\times10^{-8}$). Permutation testing was not applied owing to futility (see **Supplementary Methods**). Nevertheless, fixed effect meta-analysis of the European and Asian lnORs did not identify significant heterogeneity in the lnORs for rs28568531 ($P_{\text{het meta}} = 0.215$). (Note that rs867436 was not tested in the Asian panels, nor rs6422657 in the European panels.)

Note that in the example of risk locus 1p36.32 (**Suppl. Figure 2.1**, above), even though the Asian panels contained far fewer variants available for testing than the European panels (75 compared to 314), and the combined panels fewer still (see the "Plotted SNPs" track shown at the top of each plot), the lead variant in the combined panels (rs28568531) was in strong LD both with the lead variant in the European panels (rs867436) and the lead variant in the Asian panels (rs6422657), and thus represents a viable surrogate for either variant. Power calculations carried out using GPC (http://zzz.bwh.harvard.edu/gpc/) indicated that a case/control study of the same size as the Asian (combined Chinese and Japanese) panel would have good power (93% or 81% respectively) to detect at P = 0.05 or P = 0.01 respectively an effect of the same magnitude (OR = 1.131) seen in the European panel at this locus, but limited power (56% or 40% respectively) to detect such an effect at more compelling significance levels of P = 0.001 or P = 0.0002 respectively. The power drops further if we assume that the true magnitude of lnOR is one standard deviation less in Asians than in Europeans (power = 81%, 60%, 32%, 18% for P = 0.05, 0.01, 0.001, 0.0002, respectively), and further still if we assume that the true magnitude of lnOR is 1.96 standard deviations less in Asians than in Europeans (power = 60%, 36% 14%, 6.6% for P = 0.05, 0.01, 0.001, 0.0002, respectively). We note that these assumed lnORs (of 0.101 and 0.079 respectively) are not unreasonable given the fact that the observed lnOR at rs28568531 in Asians (lnOR = 0.068) was not significantly different from that seen (lnOR = 0.123) at rs28568531 in Europeans. The power would be lowered still further if (as is the case at some other loci) there were, in fact, no good surrogate variant measured in Asians for the top variant seen in Europeans.

On balance, we consider the signal at 1p36.32 to be well supported in European populations. Conversely, we conclude that the study was most likely under-powered

to reliably confirm or refute association in Asian populations, or trans-ethnic heterogeneity, at this locus.

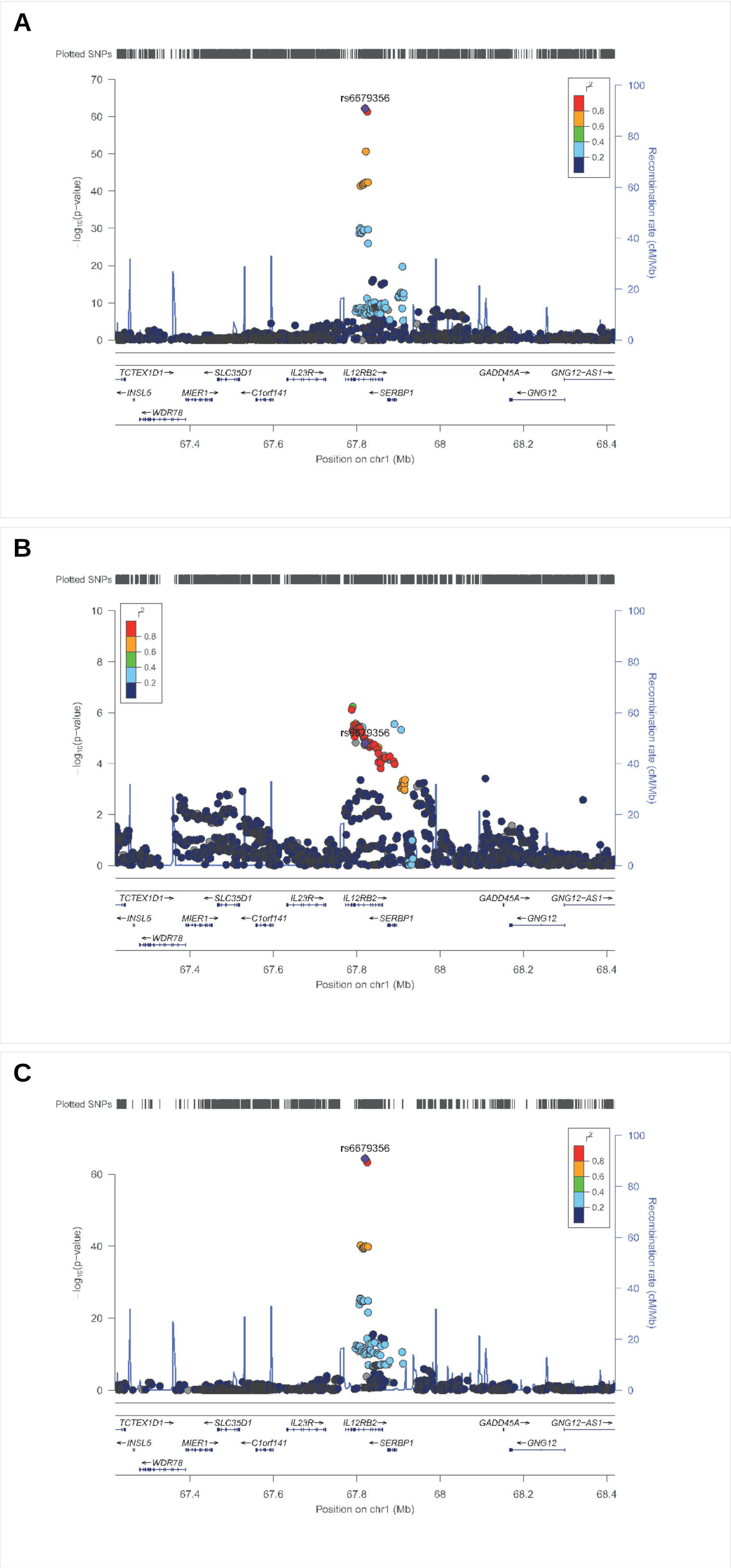


**Fig. S2.2: LocusZoom plots of known PBC risk locus 1p31.3.** (A) European panels, (B) Asian panels, and (C) all panels combined. Association at this locus reached genome-wide significance in the European panels (rs6679356 at 1:67,820,194; $P = 6.61\times10^{-63}$); suggestive significance in the Asian panels (rs150855560 at 1:67,790,300; $P = 5.75\times10^{-7}$); and genome-wide significance in all panels combined (rs6679356; $P = 4.85\times10^{-65}$). Permutation testing confirmed that the European signal at rs6679356 was corroborated by an Asian signal at rs150855560 ($P_{permutation} = 0.0004$). Furthermore, fixed effect meta-analysis of the European and Asian lnORs did not identify significant heterogeneity in the lnORs for rs6679356 ($P_{het\ meta} = 0.968$). (Note that rs150855560 was not tested in the European panels.) Thus, we consider the signal at 1p31.3 to be well supported across both European and Asian populations.

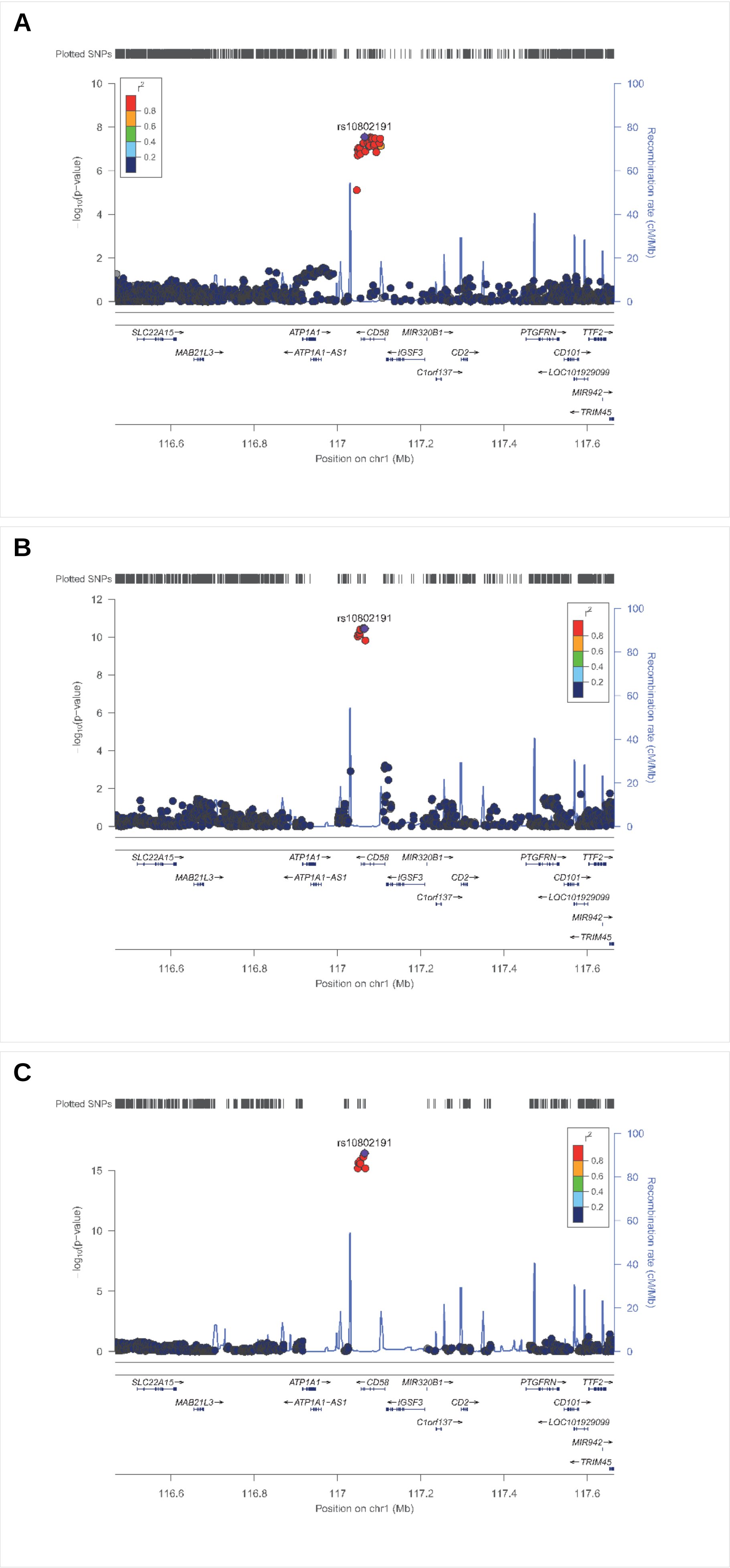


**Fig. S2.3: LocusZoom plots of the known PBC risk locus 1p13.1.** (A) European panels, (B) Asian panels, and (C) all panels combined. Association at this locus reached genome-wide significance in the European panels (rs10802191 at 1:117,065,083; $P = 2.81\times10^{-8}$), Asian panels (rs10802190 at 1:117,061,384; $P = 3.42\times10^{-11}$) and all panels combined (rs10802191; $P = 3.68\times10^{-17}$). Permutation testing confirmed that the European signal at rs10802191 was corroborated by an Asian signal at rs10802190 ($P_{permutation}$ <0.0001). Furthermore, fixed effect meta-analysis of the European and Asian lnORs did not identify significant heterogeneity in the lnORs for rs10802190 ($P_{het\ meta}$ = 0.345) or rs10802191 ($P_{het\ meta}$ = 0.390). Thus, we consider the signal at 1p13.1 to be well supported across both Asian and European populations.

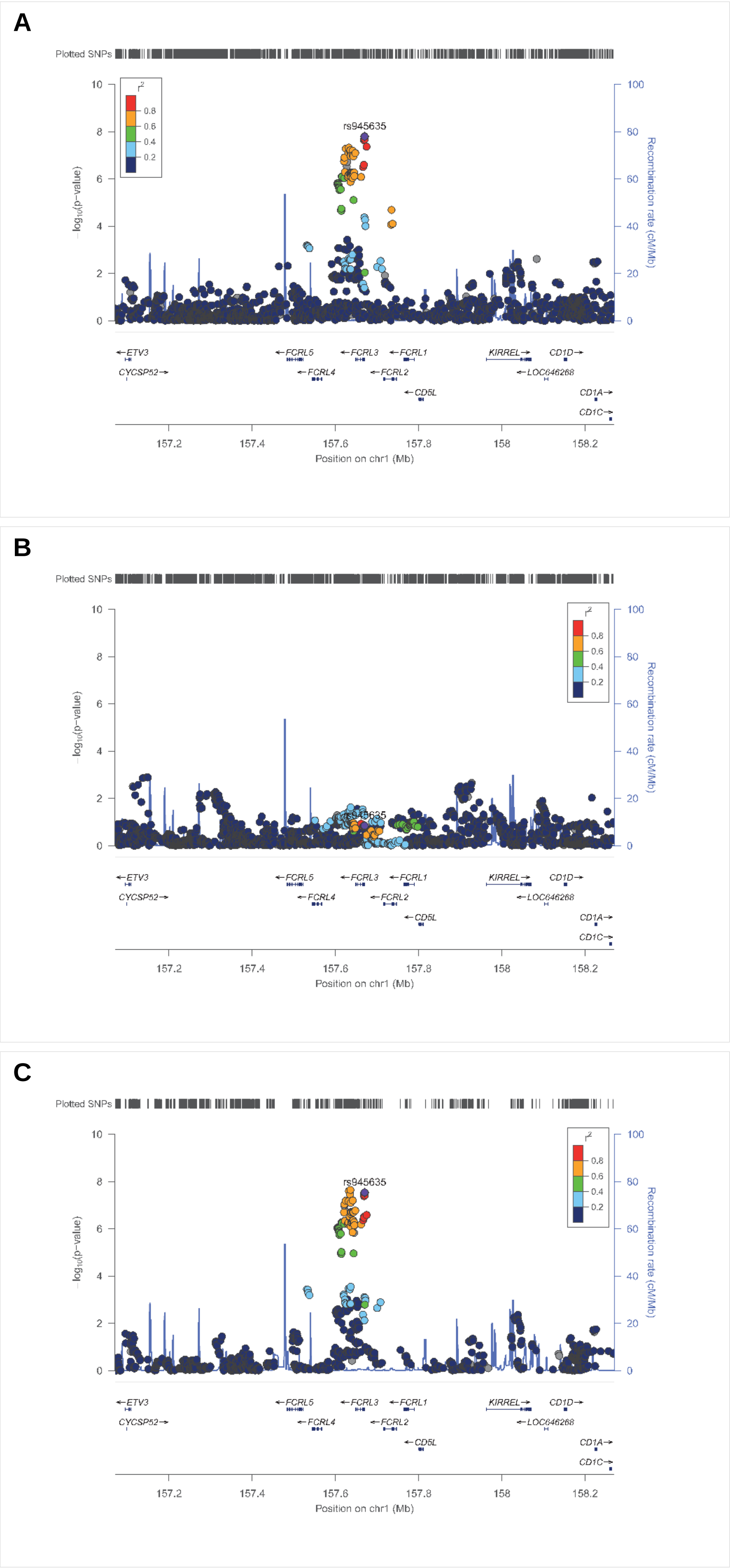


**Fig. S2.4: LocusZoom plots of the newly identified PBC risk locus 1q23.1.** (A) European panels, (B) Asian panels, and (C) all panels combined. Association at this locus reached genome-wide significance in the European panels (rs945635 at 1:157,670,290; $P = 1.59 \times 10^{-8}$); nominal significance ~0.523 Mb away (and in a different LD block) in the Asian panels (rs60459521 at 1:157,147,588; $P = 0.0012$); and genome-wide significance in all panels combined (rs11264790 at 1:157,636,074; $P = 2.25 \times 10^{-8}$). Permutation testing was not applied owing to futility. Nevertheless, fixed effect meta-analysis of the European and Asian lnORs did not identify significant heterogeneity in the lnORs for rs945635 ($P_{het\ meta} = 0.140$) or rs11264790 ($P_{het\ meta} = 0.437$). (Note that rs60459521 was not tested in the European panels.) On balance, we consider the signal at 1q23.1 to be supported in European populations. Conversely, the study was most likely under-powered to reliably confirm or refute association in Asian populations, or trans-ethnic heterogeneity, at this locus.

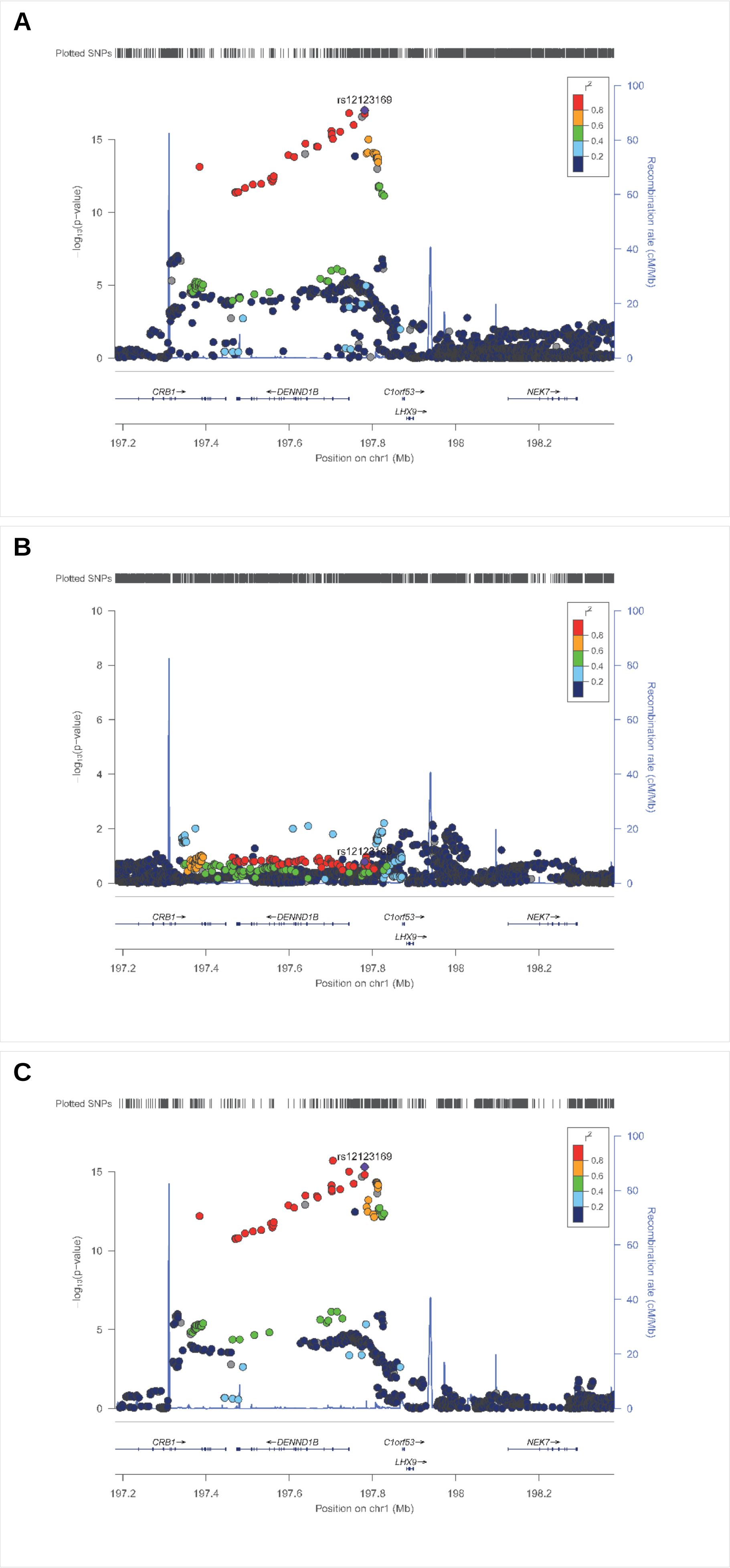


**Fig. S2.5: LocusZoom plots of the known PBC risk locus 1q31.3.** (A) European panels, (B) Asian panels, and (C) all panels combined. Association at this locus reached genome-wide significance in the European panels (rs12123169 at 1:197,780,966; $P = 9.75\times10^{-18}$); nominal significance in the Asian panels (rs35207123 at 1:197,827,444; $P = 0.0062$); and genome-wide significance in all panels combined (rs17641524 at 1:197704717; $P = 2.00\times10^{-16}$). Permutation testing was not applied owing to futility. Fixed effect meta-analysis of the European and Asian lnORs identified significant heterogeneity in the lnORs for rs12123169 ($P_{het\ meta} = 0.007$), but not for rs35207123 ($P_{het\ meta} = 0.932$) or rs17641524 ($P_{het\ meta} = 0.562$). On balance, we consider the signal at 1q31.3 to be well supported in European populations. Conversely, the study was most likely under-powered to reliably confirm or refute association in Asian populations, or trans-ethnic heterogeneity, at this locus.

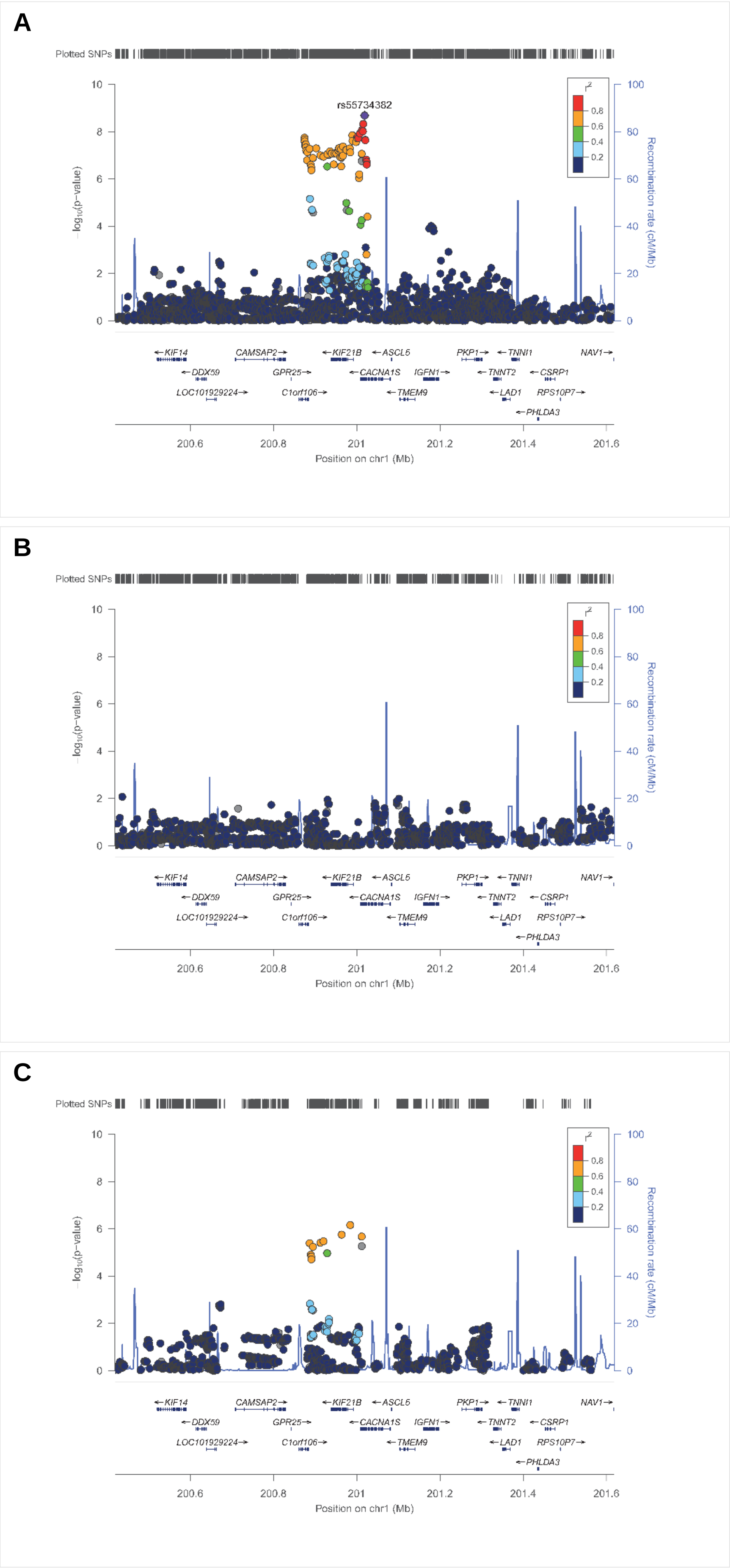


**Fig. S2.6: LocusZoom plots of the newly identified PBC risk locus 1q32.1.** (A) European panels, (B) Asian panels, and (C) all panels combined. Association at this locus reached genome-wide significance in the European panels (rs55734382 at 1:201,019,059; $P = 2.06\times10^{-9}$); nominal significance in the Asian panels (rs117214467 at 1:200,436,787; $P = 0.0085$); and suggestive significance in all panels combined (rs12122721 at 1:200,984,480; $P = 6.95\times10^{-7}$). Permutation testing was not applied owing to futility. Nevertheless, fixed effect meta-analysis of the European and Asian lnORs did not identify significant heterogeneity in the lnORs for rs12122721 ($P_{het\ meta} = 0.064$). (Note that rs55734382 was not tested in the Asian panels, nor rs117214467 in the European panels.) On balance, we consider the signal at 1q32.1 to be supported in European populations. Conversely, the study was most likely under-powered to reliably confirm or refute association in Asian populations, or trans-ethnic heterogeneity, at this locus.

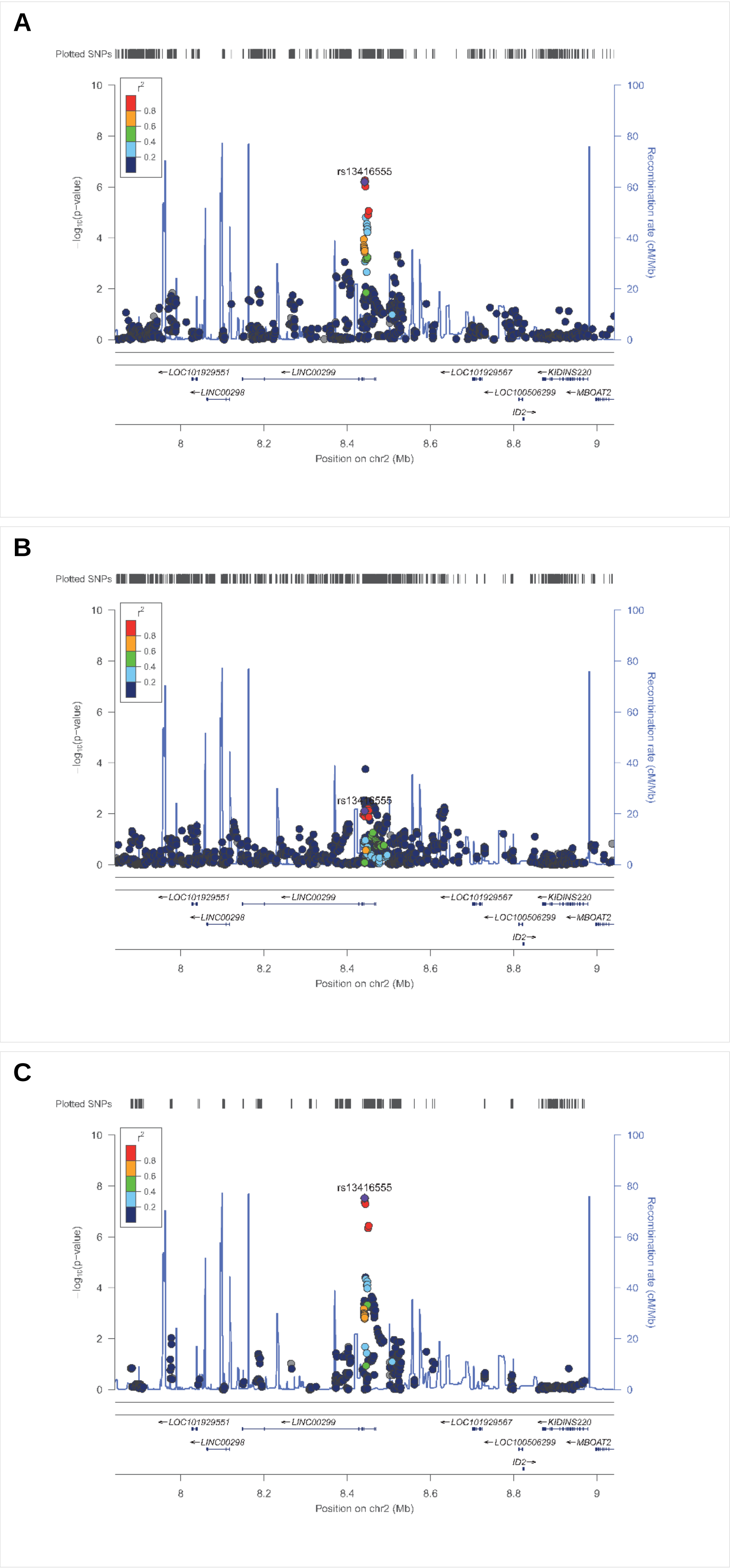


**Fig. S2.7: LocusZoom plots of the newly identified PBC risk locus 2p25.1.** (A) European panels, (B) Asian panels, and (C) all panels combined. Association at this locus reached suggestive significance in the European panels (rs891058 at 2:8,442,547; $P = 5.39\times10^{-7}$); suggestive significance in the Asian panels (rs3111414 at 2:8,443,859; $P = 1.75\times10^{-4}$); and genome-wide significance in all panels combined (rs13416555 at 2:8,441,735; $P = 2.95\times10^{-8}$). Permutation testing confirmed that the European signal at rs891058 was corroborated by an Asian signal at rs3111414 ($P_{permutation} = 0.0017$). Furthermore, fixed effect meta-analysis of the European and Asian lnORs did not identify significant heterogeneity in the lnORs for rs891058 ($P_{het\ meta} = 0.858$), rs3111414 ($P_{het\ meta} = 0.065$), or rs13416555 ($P_{het\ meta} = 0.984$). Thus, we consider the signal at 2p25.1 to be supported across European and Asian populations.

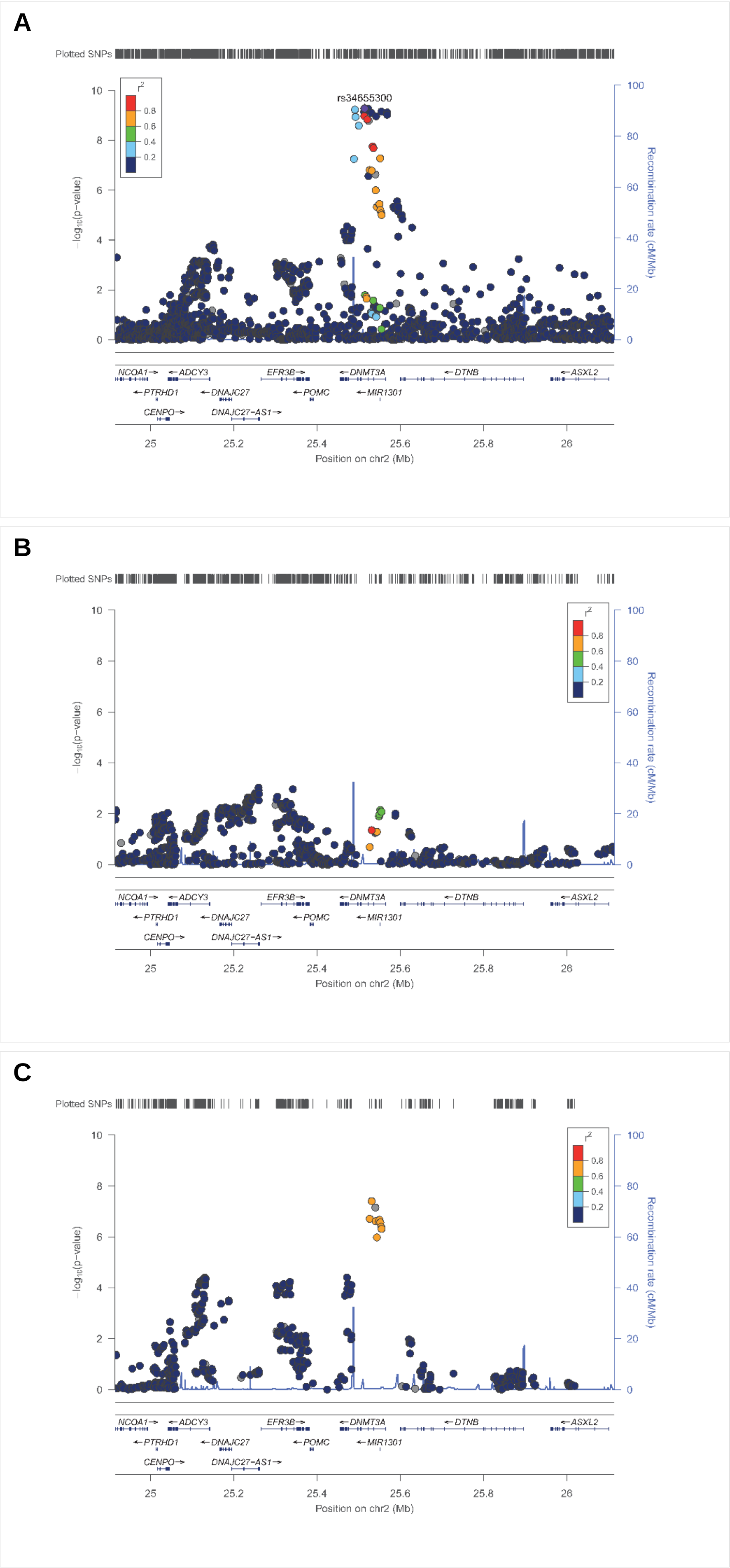


**Fig. S2.8: LocusZoom plots of the newly identified PBC risk locus 2p23.3.** (A) European panels, (B) Asian panels, and (C) all panels combined. The association signal reached genome-wide significance in the European panels (rs34655300 at 2:25,514,333; $P = 5.23 \times 10^{-10}$); nominal significance ~0.255 Mb away (and in a different LD block) in the Asian panels (rs893589 at 2:25,259,442; $P = 9.40 \times 10^{-4}$); and genome-wide significance in all panels combined (rs6711622 at 2:25,531,350; $P = 3.89 \times 10^{-8}$). Permutation testing was not applied owing to futility. Fixed effect meta-analysis of the European and Asian lnORs identified significant heterogeneity in the lnORs for rs893589 ($P_{het\ meta} = 0.002$), but not for rs6711622 ($P_{het\ meta} = 0.725$). (Note that rs34655300 was not tested in the Asian panels.) On balance, we consider the signal at 2p23.3 to be supported in European populations. Conversely, the study was most likely under-powered to reliably confirm or refute association in Asian populations, or trans-ethnic heterogeneity, at this locus.

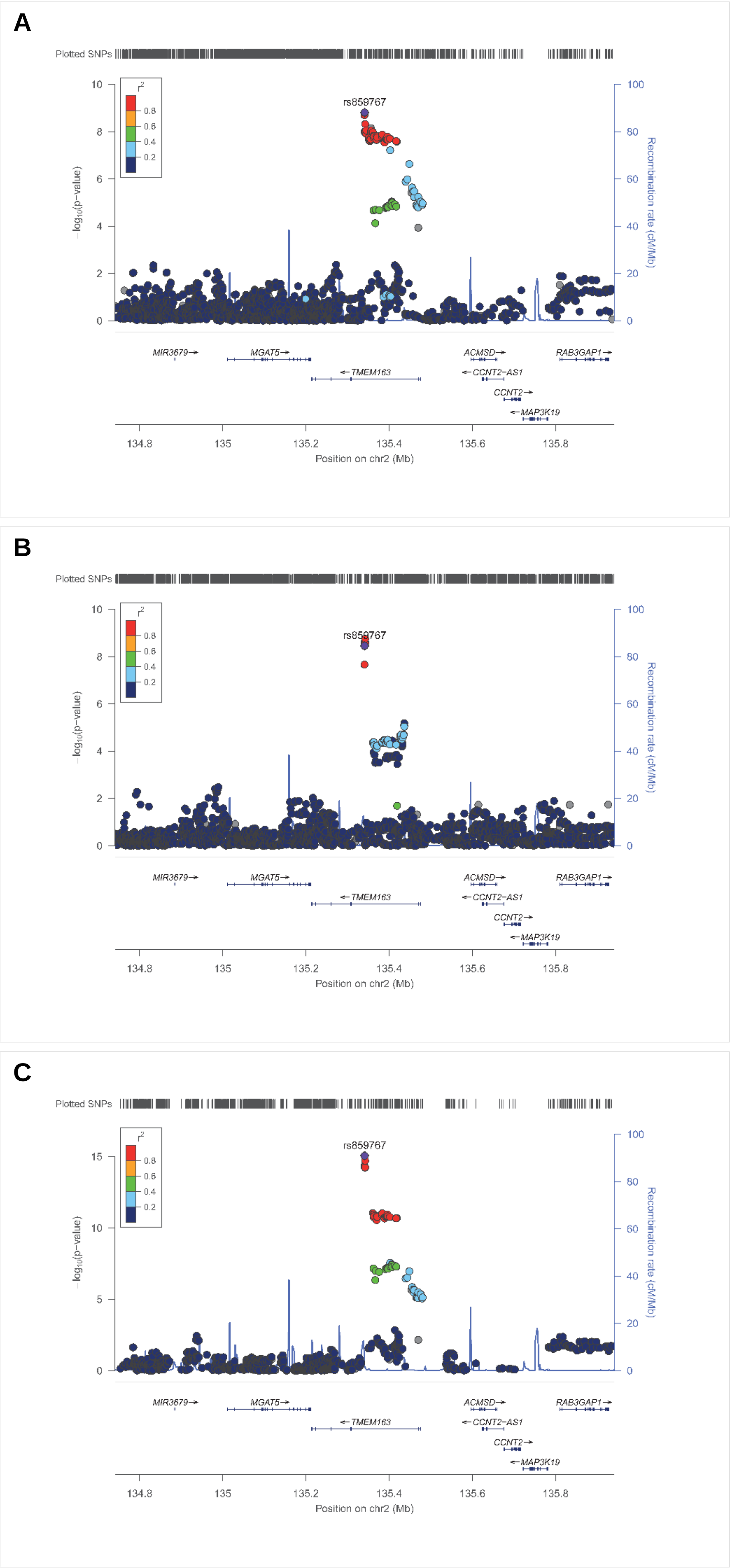


**Fig. S2.9: LocusZoom plots of the newly identified PBC risk locus 2q21.3.** (A) European panels, (B) Asian panels, and (C) all panels combined. Association at this locus reached genome-wide significance in the European panels (rs859767 at 2:135,341,200; $P = 1.54\times10^{-9}$), Asian panels (rs842349 at 2:135,342,452; $P = 1.76\times10^{-9}$), and all panels combined (rs859767; $P = 8.94\times10^{-16}$). Permutation testing confirmed that the European signal at rs859767 was corroborated by an Asian signal at rs842349 ($P_{permutation}$ <0.0001). Fixed effect meta-analysis of the European and Asian lnORs did, however, identify significant heterogeneity in the lnORs for rs842349 ($P_{het\ meta} = 0.027$) and rs859767 ($P_{het\ meta} = 0.043$). Thus, we consider the signal at 2q21.3 to be well supported across both European and Asian populations, potentially with a larger effect size in Asians.

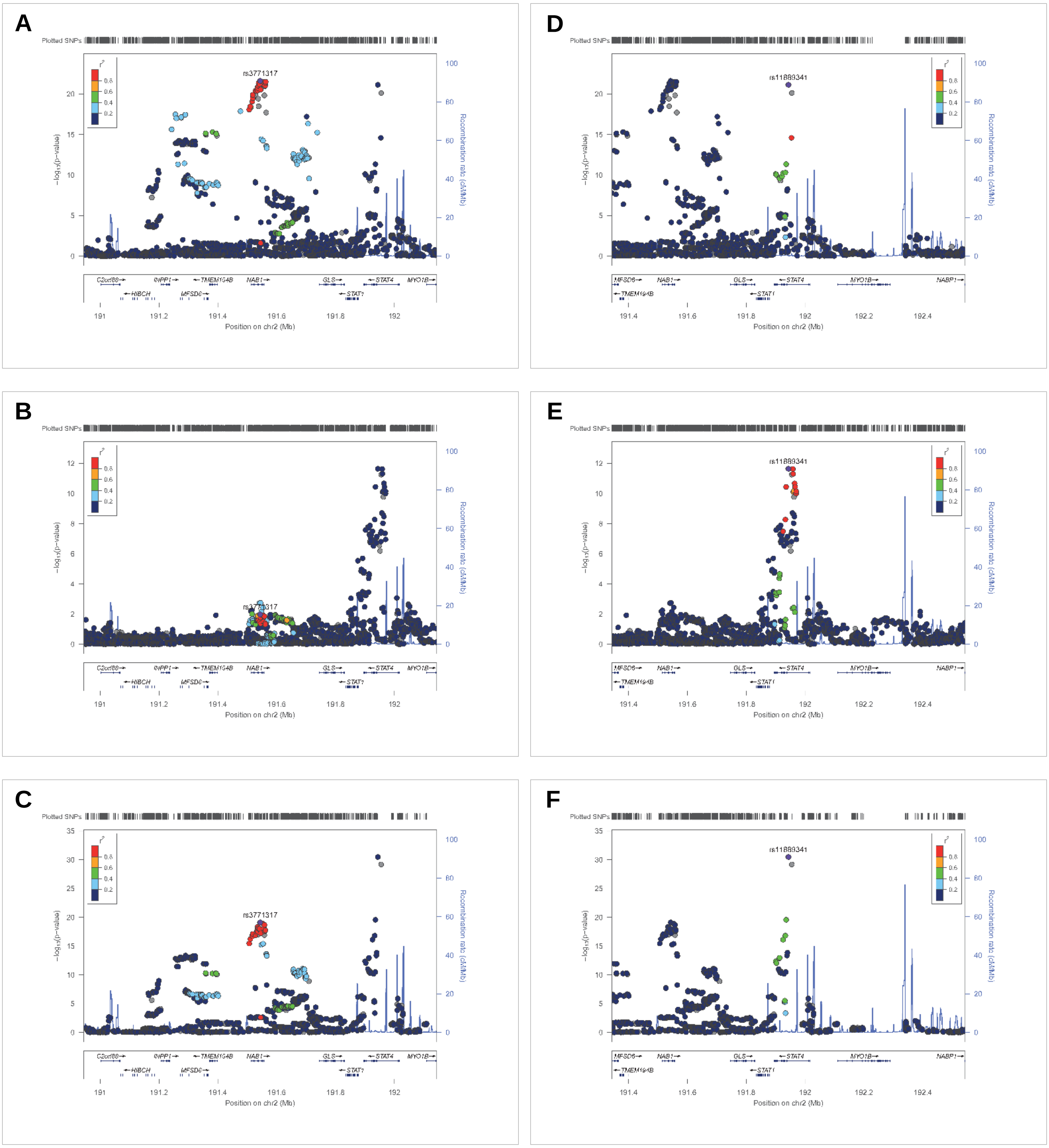


**Fig. S2.10: LocusZoom plots showing two, independent association signals at the known PBC risk locus 2q32.2.** (A, D) European panels, (B, E) Asian panels, (C, F) all panels combined. The first independent association signal (rs3771317 at 2:191,543,962) is evident in the European (A) and combined (C) panels, but not the Asian panels (B). Therefore, permutation testing was not applied to this signal. Conversely, the second independent association reached genome-wide significance in the European (rs141442738 at 2:191,955,189; P = $7.61\times10^{-21}$; plot D), Asian (rs11889341 at 2:191,943,742; P = $2.27\times10^{-12}$; plot E) and combined (rs11889341; P = $3.41\times10^{-31}$; plot F) panels. Permutation testing confirmed that the European signal at rs141442738 was corroborated by the Asian signal at rs11889341 ($P_{permutation}$ <0.0001). Fixed effect meta-analysis of the European and Asian lnORs identified significant heterogeneity in the lnORs for rs3771317 ($P_{het\ meta}$ = $1.12\times10^{-4}$), but not for rs11889341 ($P_{het\ meta}$ = 0.284). Thus, we consider this second signal at 2q32.2 to be well supported across both European and Asian populations.

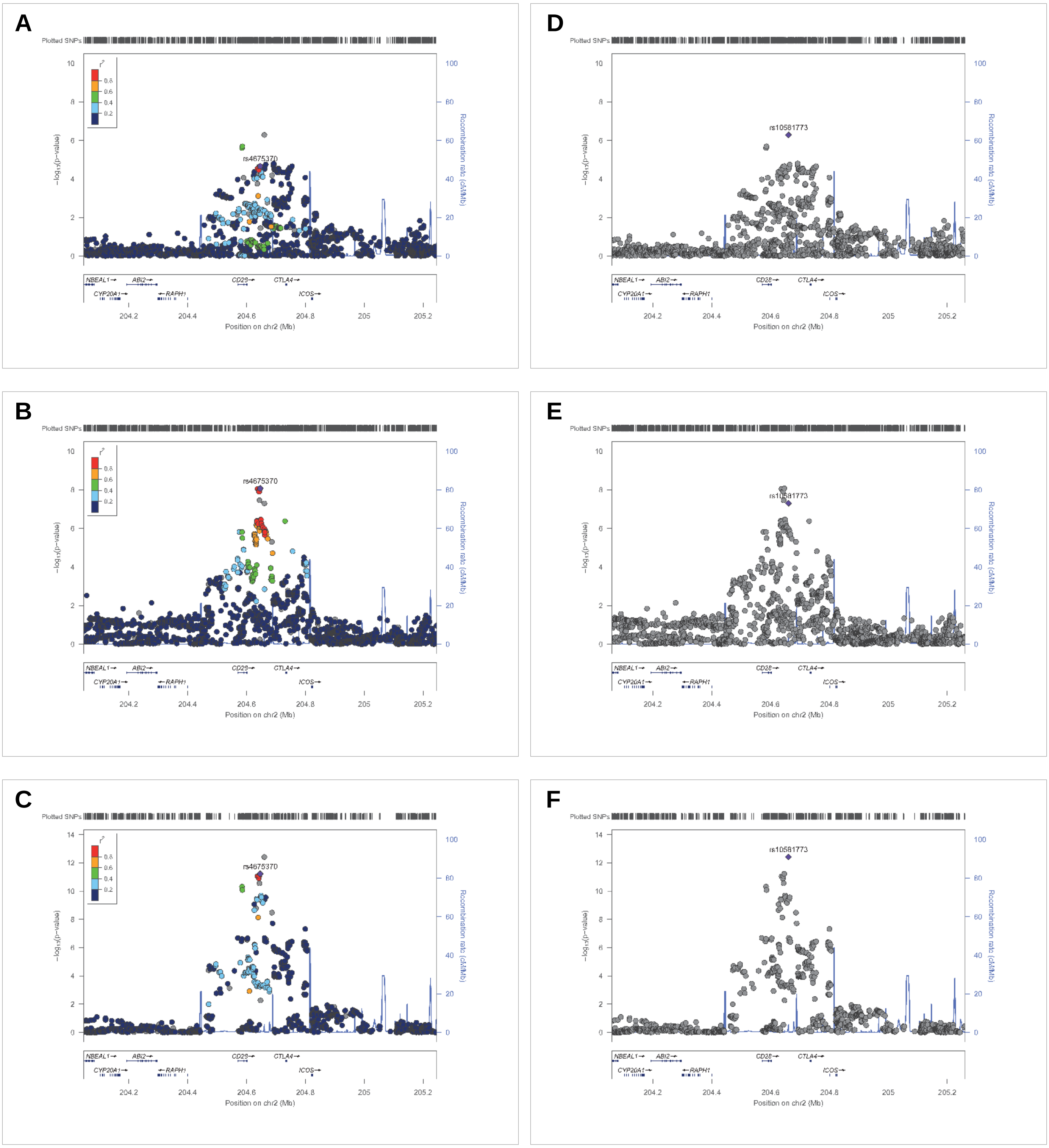


**Fig. S2.11: LocusZoom plots of the known risk locus PBC risk locus 2q33.2.** (A, D) European panels, (B, E) Asian panels, and (C, F) all panels combined. An Asian signal at rs4675370 ($P = 8.49 \times 10^{-9}$; plot B) reached suggestive level of significance in Europeans ($P = 2.17 \times 10^{-5}$; plot A), while rs10581773 (14,249 BP away) reached $P = 5.00 \times 10^{-8}$ in Asians and $P = 5.19 \times 10^{-7}$ in Europeans, resulting in overall significance levels of $P = 5.94 \times 10^{-12}$ at rs4675370, and $P = 3.82 \times 10^{-13}$ at rs10581773, in the combined panels. Permutation testing and standard meta-analysis measures of heterogeneity indicated that the European signal at rs10581773 represented a significant corroboration of the Asian signal at rs4675370 ($P_{permutation} < 0.0001$), with no significant heterogeneity seen between the Asian and European lnORs at rs10581773 ($P_{het\ meta} = 0.949$) or rs4675370 ($P_{het\ meta} = 0.173$). Thus, we consider this signal to be well supported across both European and Asian populations.

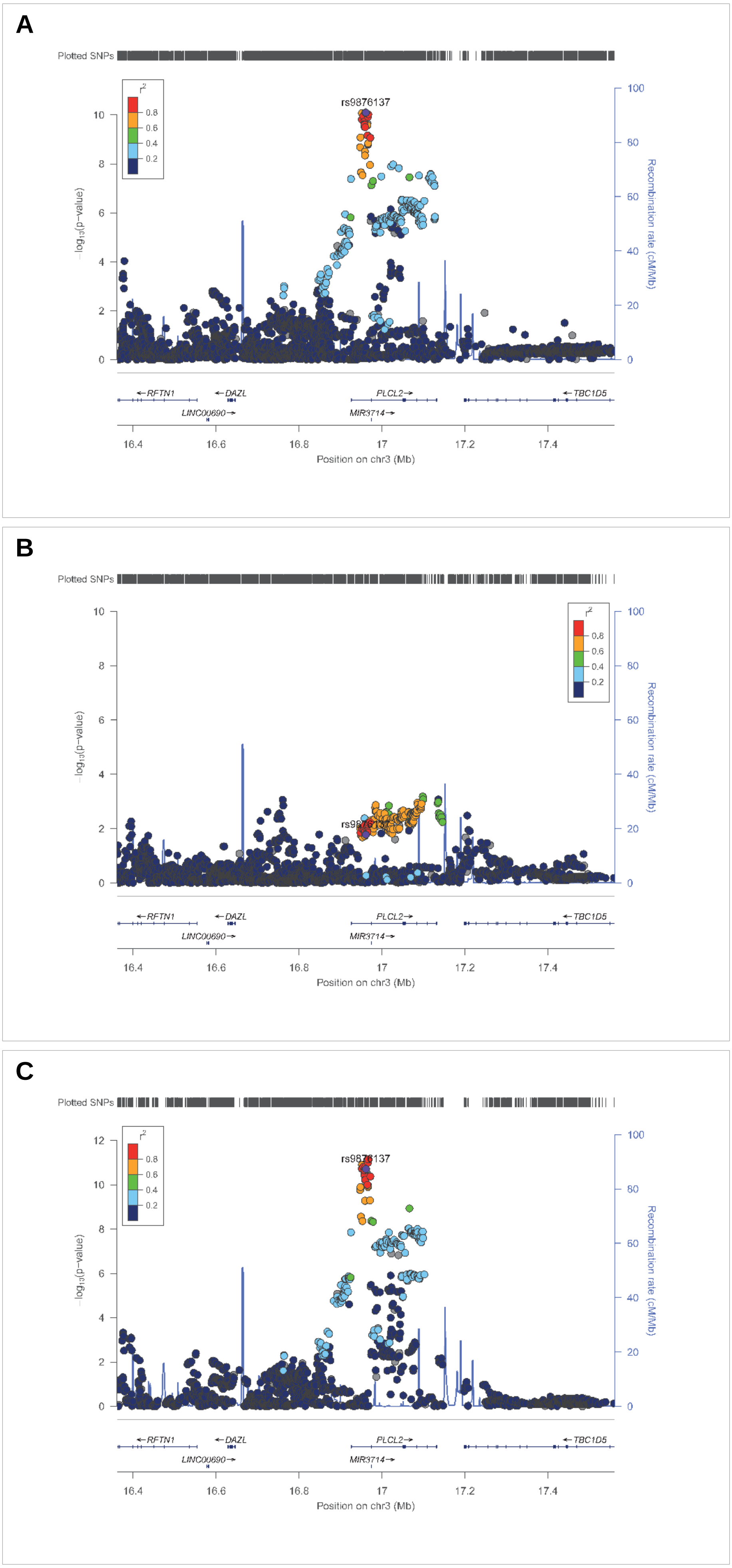


**Fig. S2.12: LocusZoom plots of the known PBC risk locus 3p24.3.** (A) European panels, (B) Asian panels, and (C) all panels combined. Association at this locus reached genome-wide significance in the European panels (rs9876137 at 3:16,961,265; $P = 8.06\times10^{-11}$); nominal significance in the Asian panels (rs4561816 at 3:17,098,500; $P = 6.58\times10^{-4}$); and genome-wide significance in all panels combined (rs1156336 at 3:16,967,221; $P = 7.13\times10^{-12}$). Permutation testing was not applied owing to futility. Nevertheless, fixed effect meta-analysis of the European and Asian lnORs did not identify significant heterogeneity in the lnORs for rs9876137 ($P_{het\ meta}$ = 0.261), rs4561816 ($P_{het\ meta}$ = 0.404), or rs1156336 ($P_{het\ meta}$ = 0.399). On balance, we consider the signal at 3p24.3 to be well supported in European populations. Conversely, notwithstanding the plausible level of association seen in the Asian panels, the study was most likely under-powered to definitively confirm or refute association in Asian populations, or trans-ethnic heterogeneity, at this locus.

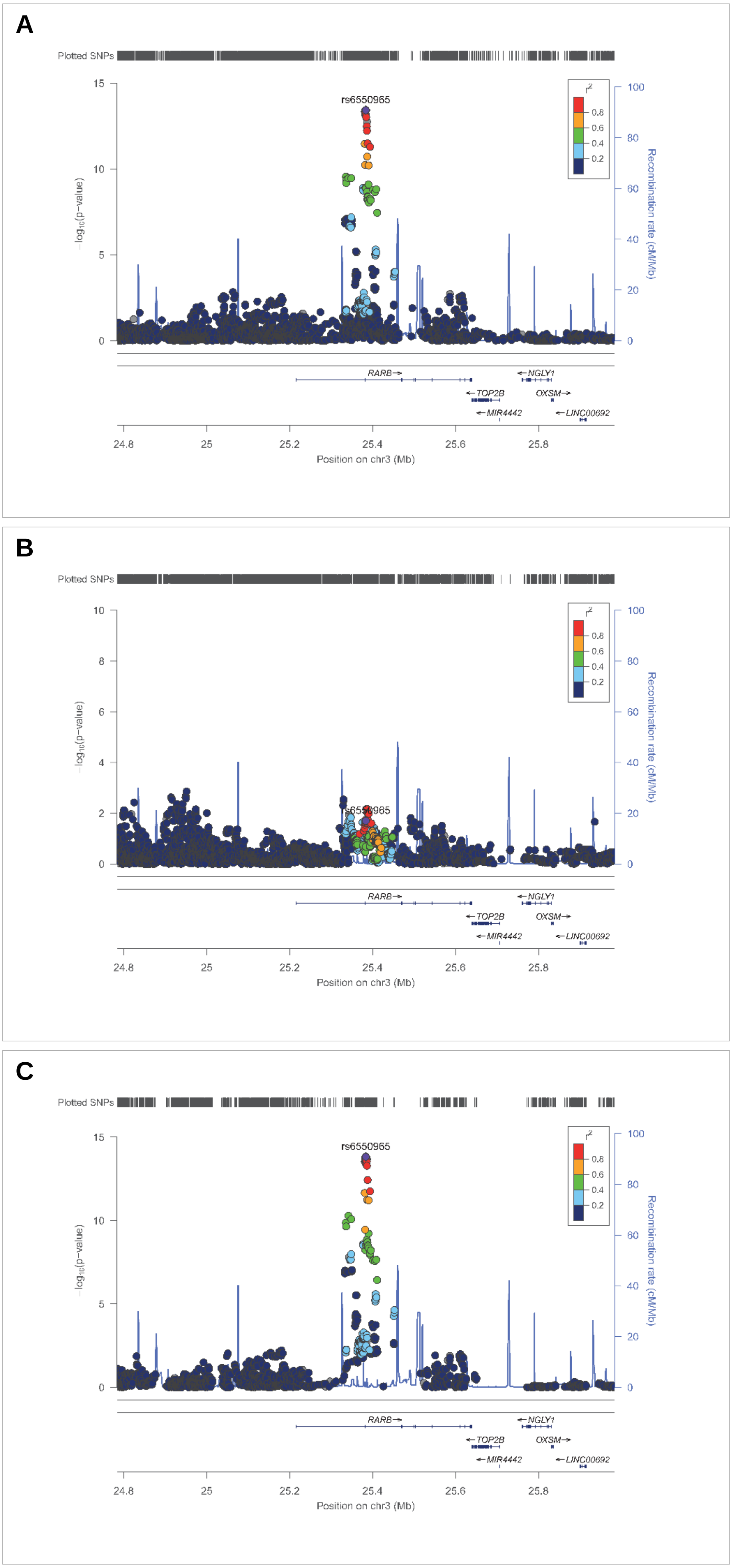


**Fig. S2.12: LocusZoom plots of the newly identified PBC risk locus 3p24.2.** (A) European panels, (B) Asian panels, and (C) all panels combined. Association at this locus reached genome-wide significance in the European panels (rs6550965 at 3:25,383,587; $P = 3.65\times10^{-14}$); nominal significance in the Asian panels (rs6807549 at 3:24,951,404; $P = 0.0014$); and genome-wide significance in the combined panels (rs6550965; $P = 1.50\times10^{-14}$). Permutation testing was not applied owing to futility. Fixed effect meta-analysis of the European and Asian lnORs identified significant heterogeneity in the lnORs for rs6807549 ($P_{het\ meta} = 0.001$), but not for rs6550965 ($P_{het\ meta} = 0.225$). On balance, we consider the signal at 3p24.2 to be supported in European populations. Conversely, the study was most likely under-powered to reliably confirm or refute association in Asian populations, or trans-ethnic heterogeneity, at this locus.

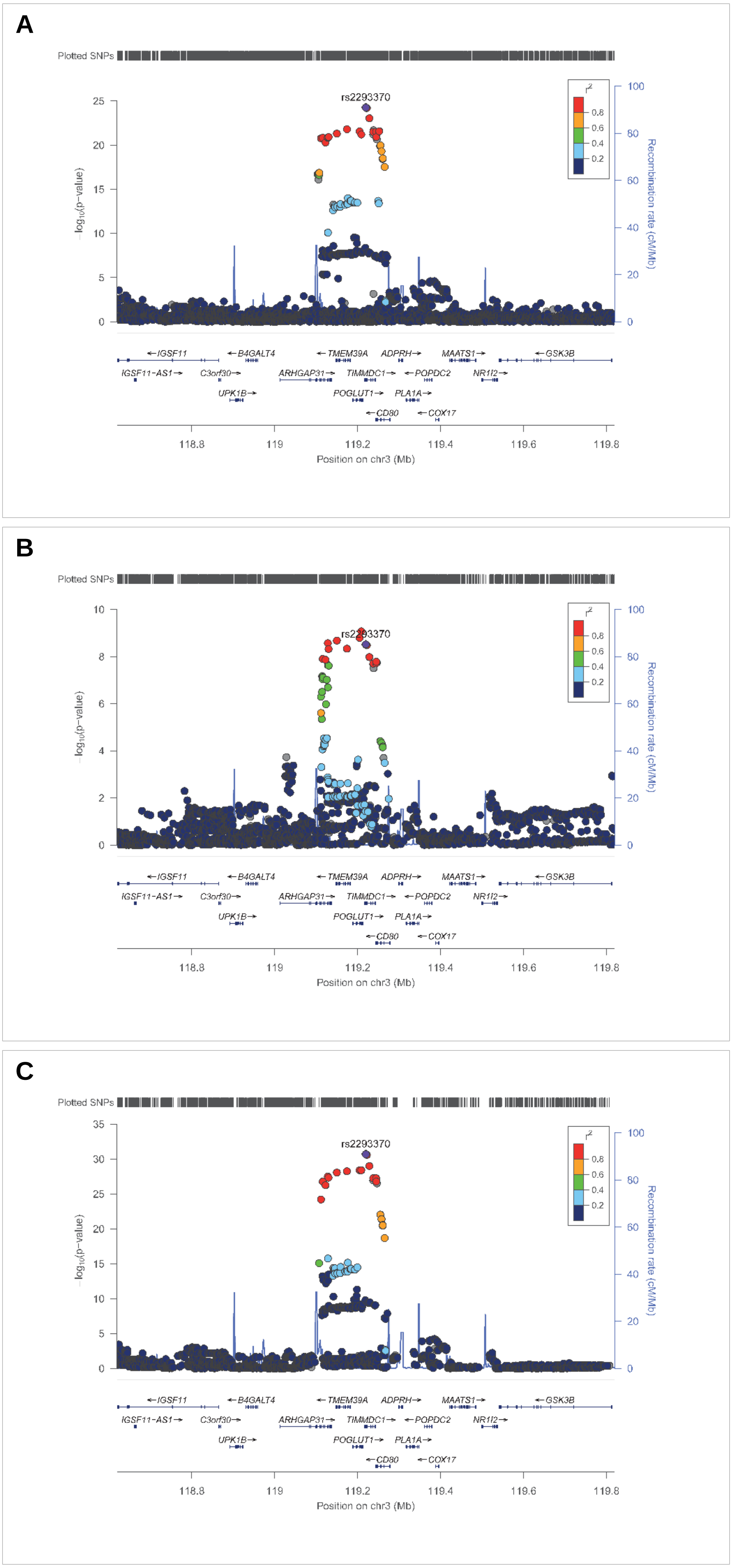


**Fig. S2.14: LocusZoom plots of the known PBC risk locus 3q13.33.** (A) European panels, (B) Asian panels, and (C) all panels combined. Association at this locus reached genome-wide significance in the European panels (rs2293370 at 3:119,219,934; $P = 5.54\times10^{-25}$), Asian panels (rs12695386 at 3:119,209,027; $P = 8.43\times10^{-10}$), and all panels combined (rs2293370; $P = 2.05\times10^{-31}$). Permutation testing confirmed that the European signal at rs2293370 was corroborated by an Asian signal at rs12695386 ($P_{permutation} <0.0001$). Furthermore, fixed effect meta-analysis of the European and Asian lnORs did not identify significant heterogeneity in the lnORs for rs12695386 ($P_{het\ meta} = 0.713$) or rs2293370 ($P_{het\ meta} = 0.412$). Thus, we consider the signal at 3q13.33 to be well supported across both European and Asian populations.

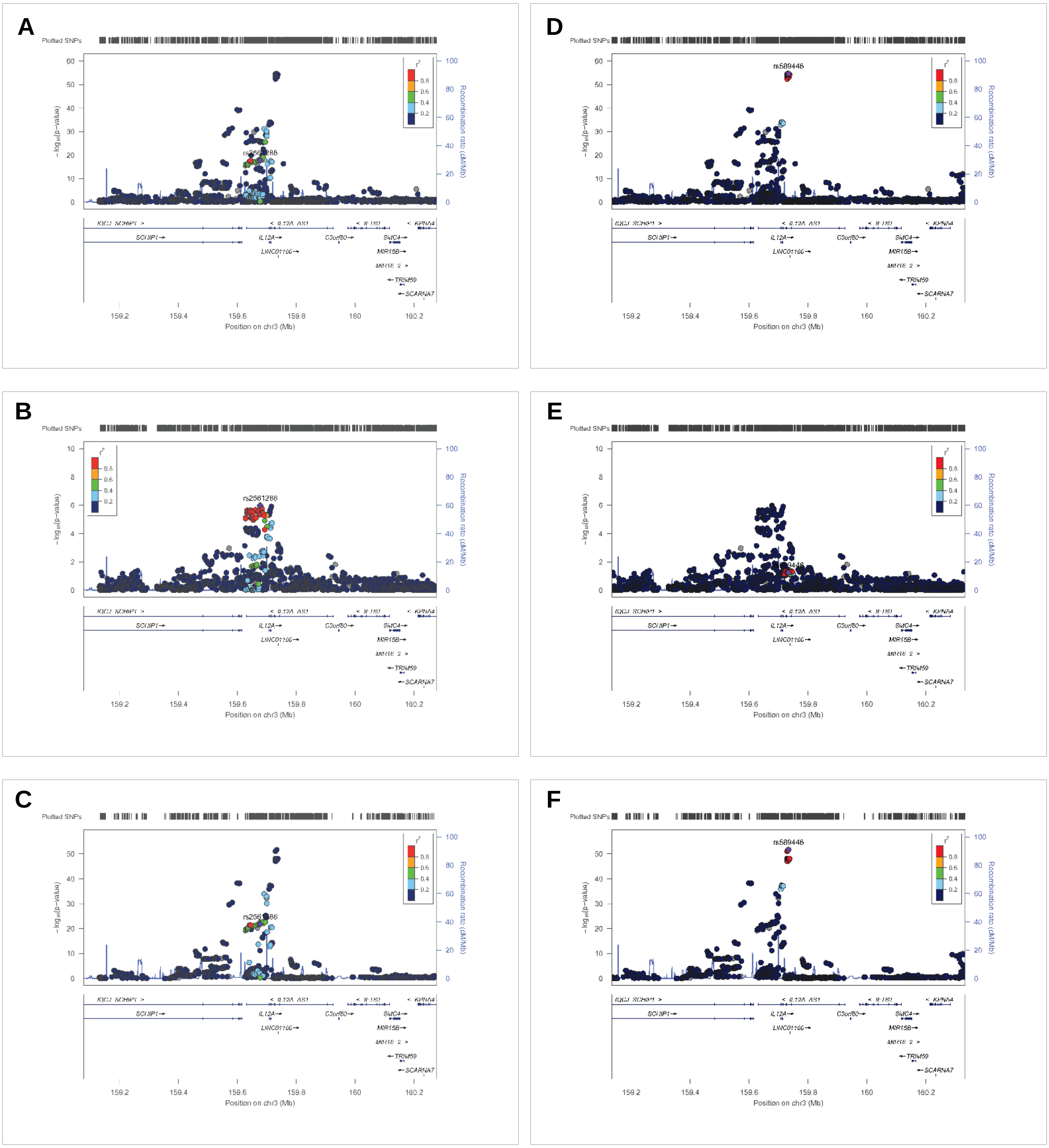


**Fig. S2.15: LocusZoom plots of the known PBC risk locus 3q25.33.** (A, D) European panels, (B, E) Asian panels, and (C, F) all panels combined. Association at this locus reached genome-wide significance in the European panels (rs589446 at 3:159,733,527; P = $1.96\times10^{-55}$; plot D); suggestive significance in the Asian panels (rs2561286 at 3:159,677,299; P = $1.02\times10^{-6}$; plot B); and genome-wide significance in the combined panels (rs589446; P=$2.23\times10^{-52}$; plot F). Permutation testing confirmed that the European signal at rs589446 was corroborated by an Asian signal at rs2561286 ($P_{permutation}$ <0.0001). Fixed effect meta-analysis of the European and Asian panels identified significant heterogeneity in the lnORs for rs589446 ($P_{het\ meta}$ = 0.001) (plots D – F), but not for rs2561286 ($P_{het\ meta}$ = 0.212). On balance, we consider the signal at 3q25.33 to be well supported across both European and Asian populations.

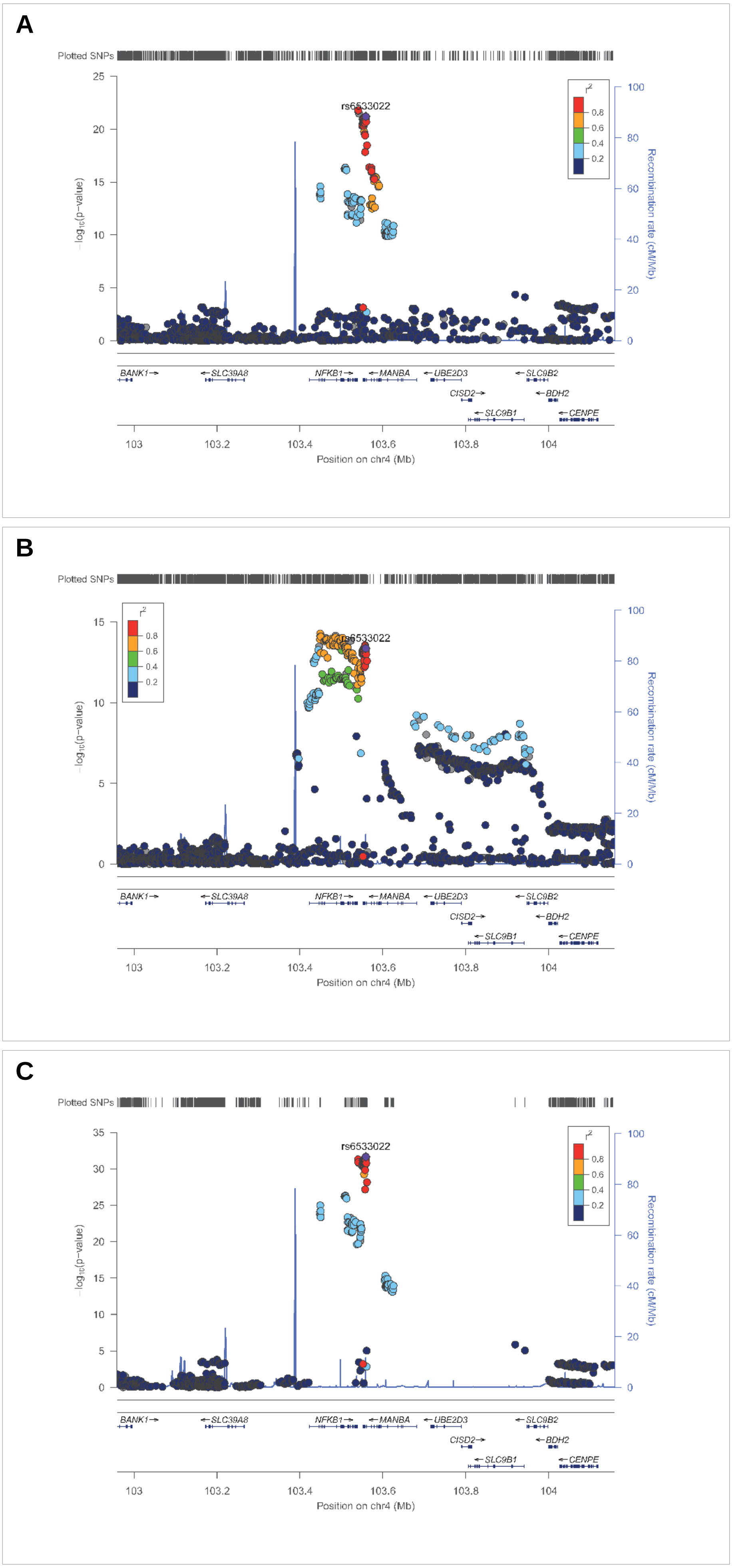


**Fig. S2.16: LocusZoom plots of the known PBC risk locus 4q24 [1].** (A) European panels, (B) Asian panels, and (C) all panels combined. Association at this locus reached genome-wide significance in the European panels (rs7674640 at 4:103,540,780; $P = 1.56\times10^{-22}$), Asian panels (rs230534 at 4:103,449,041; $P = 5.11\times10^{-15}$), and all panels combined (rs6533022 at 4:103558951; $P = 2.21\times10^{-32}$). Permutation testing confirmed that European signal at rs7674640 was corroborated by an Asian signal at rs230534 ($P_{permutation} < 0.0001$). Fixed effect meta-analysis of the European and Asian lnORs identified significant heterogeneity in the lnORs for rs230534 ($P_{het\ meta} = 0.002$), but not for rs7674640 ($P_{het\ meta} = 0.181$) or rs6533022 ($P_{het\ meta} = 0.05$). Thus, we consider this signal at 4q24 to be well supported across both European and Asian populations.

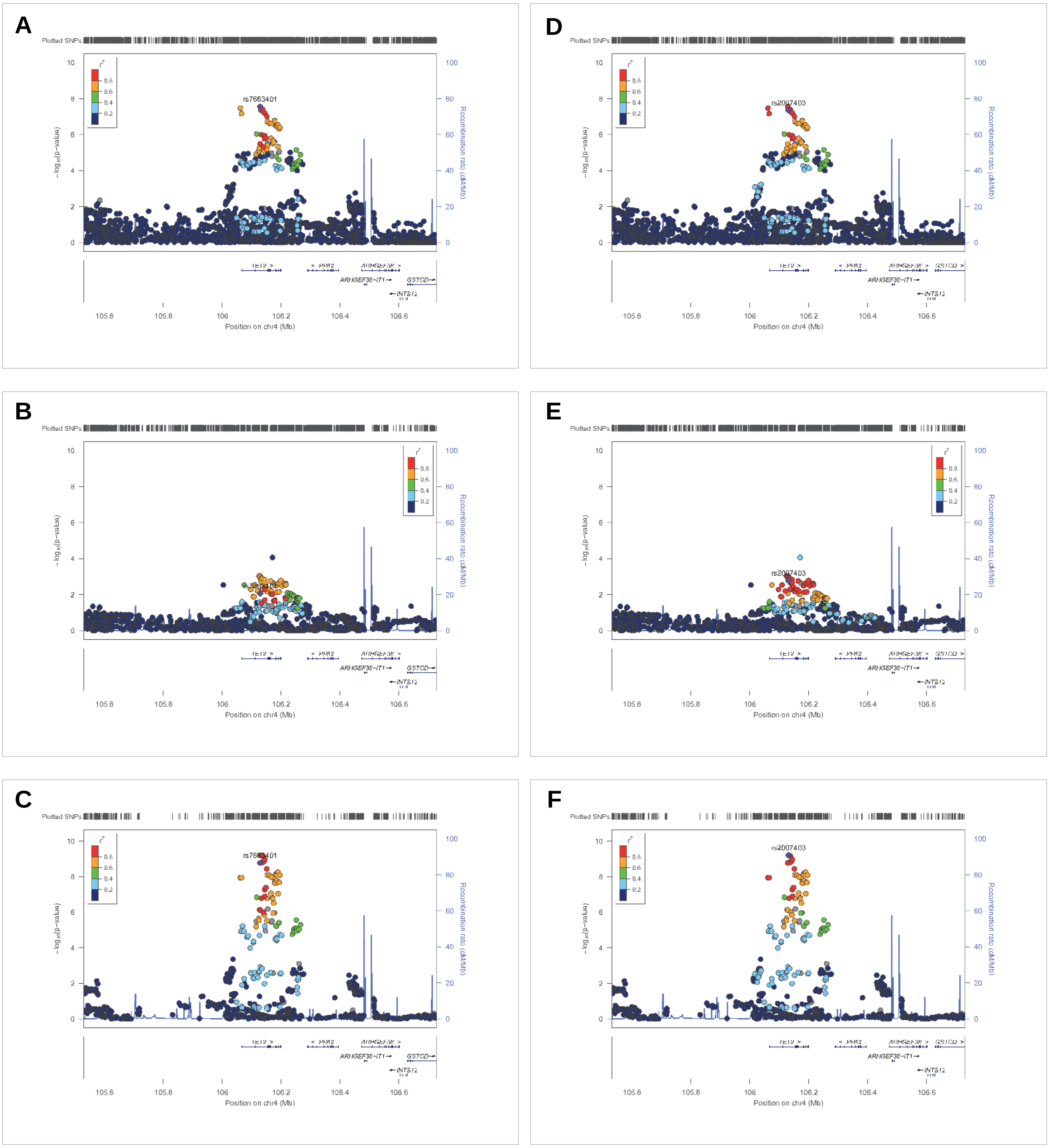


**Fig. S2.17: LocusZoom plots of the newly identified PBC risk locus 4q24 [2].** (A, D) European panels, (B, E) Asian panels, and (C, F) all panels combined. Association at this locus reached genome-wide significance in the European panels (rs7663401 at 4:106,128,954; P = $2.76\times10^{-8}$; plot D); suggestive significance in the Asian panels (rs79109654 at 4:106,170,514; P = $8.56\times10^{-5}$; plot B); and genome-wide significance in the combined panels (rs2007403 at 4:106,131,210; P = $6.19\times10^{-10}$). Permutation testing suggested that the European signal at rs7663401 was corroborated by an Asian signal at rs79109654 ($P_{permutation}$ = 0.004), albeit not at the Bonferroni-corrected threshold of P <0.00217 (correcting for 23 newly identified loci) or P < 0.000893 (correcting for 56 genome-wide significant loci). Fixed effect meta-analysis of the European and Asian panels did not identify significant heterogeneity in the lnORs for rs7663401 ($P_{het\ meta}$ = 0.773) or rs2007403 ($P_{het\ meta}$ = 0.708) (plots D – F). (Note that rs79109654 was not tested in the European panel.) Thus, we consider this signal at 4q24 to be well supported across European populations, with some evidence of support across Asian populations.

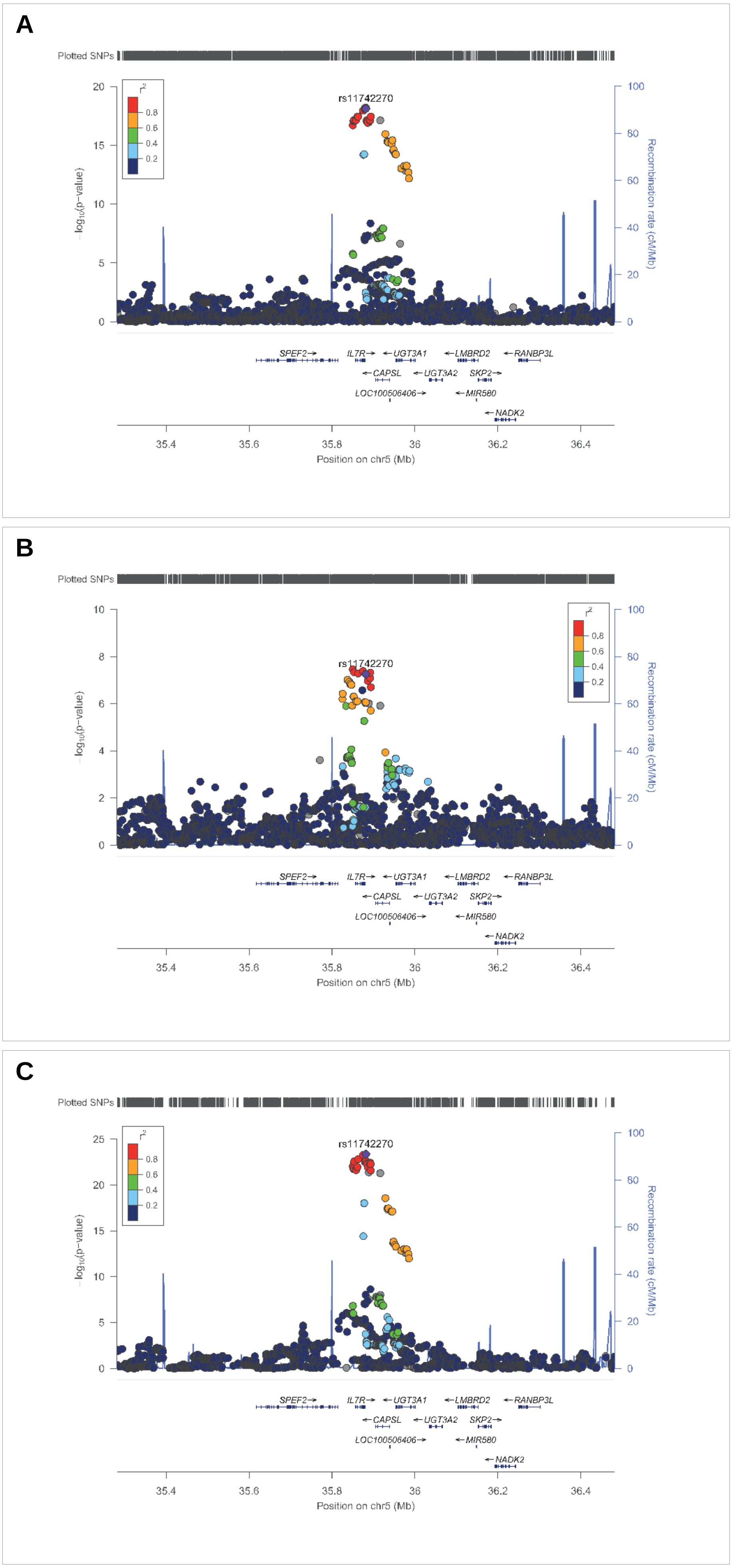


**Fig. S2.18: LocusZoom plots of the known PBC risk locus 5p13.2.** (A) European panels, (B) Asian panels, and (C) all panels combined. Association at this locus reached genome-wide significance in the European panels (rs35467801 at 5:35,881,130; $P = 6.15\times10^{-19}$), Asian panels (rs34463936 at 5:35,850,149; $P = 3.47\times10^{-8}$), and all panels combined (rs11742270 at 5:35,881,443; $P = 4.44\times10^{-24}$). Permutation testing confirmed that the European signal at rs35467801 was corroborated by an Asian signal at rs34463936 ($P_{permutation} < 0.0001$). Furthermore, fixed effect meta-analysis of the European and Asian lnORs did not identify significant heterogeneity in the lnORs for rs35467801 ($P_{het\ meta} = 0.701$), rs34463936 ($P_{het\ meta} = 0.145$), or rs11742270 ($P_{het\ meta} = 0.227$). Thus, we consider this signal to be well-supported across both European and Asian populations.

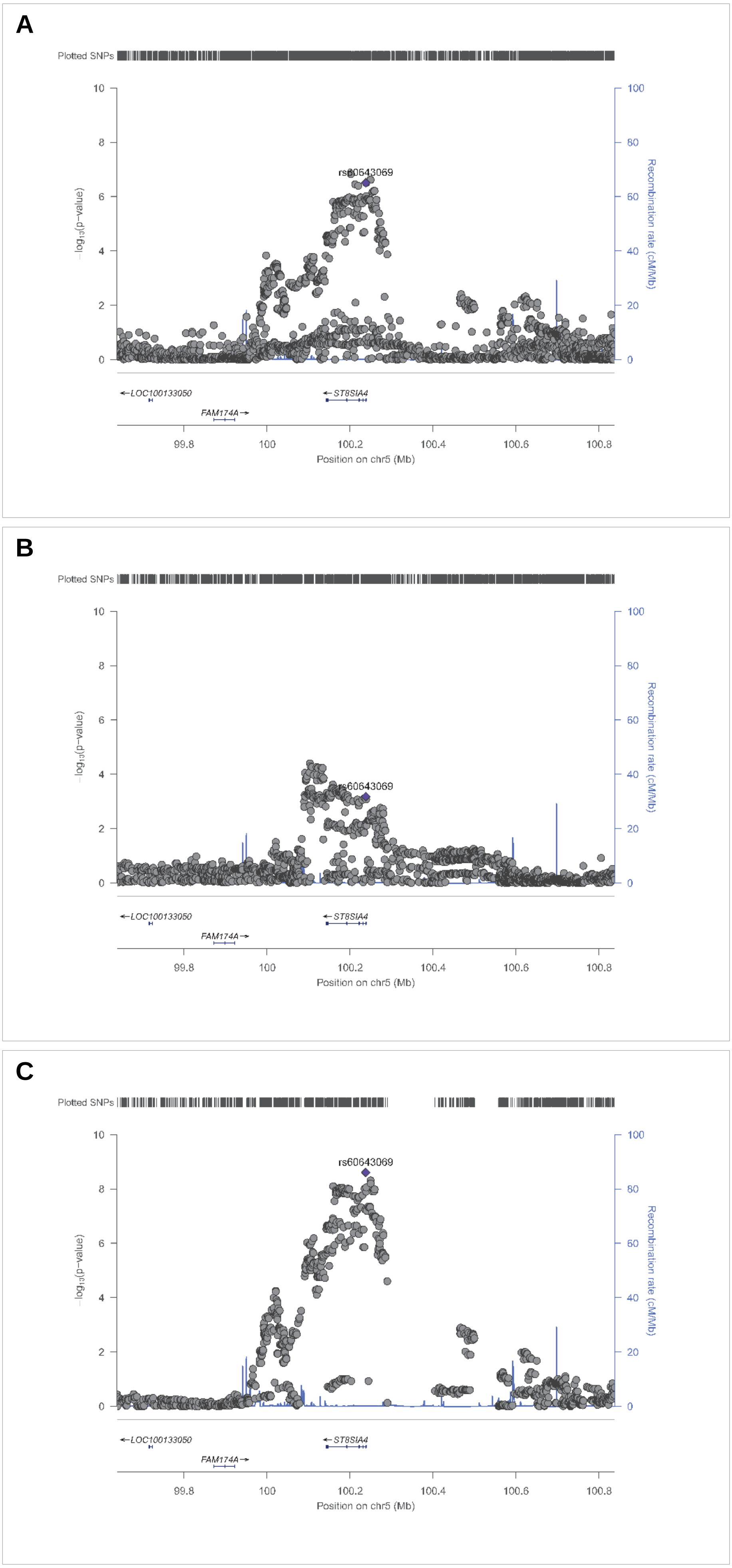


**Fig. S2.19: LocusZoom plots of the newly identified PBC risk locus 5q21.1.** (A) European panels, (B) Asian panels, and (C) all panels combined. Association at this locus reached suggestive significance in the European panels (rs141002831 at 5:100,202,282; $P = 1.47\times10^{-7}$); suggestive significance in the Asian panels (rs157181 at 5:100,103,288; $P = 3.94\times10^{-5}$); and genome-wide significance in all panels combined (rs60643069 at 5:100,238,073; $P = 2.48\times10^{-9}$). Permutation testing suggested that the European signal at rs141002831 was corroborated by an Asian signal at rs157181 ($P_{permutation} = 0.0032$), albeit not at the Bonferroni-corrected threshold of $P < 0.00217$ (correcting for 23 newly identified loci) or $P < 0.000893$ (correcting for 56 genome-wide significant loci). Fixed effect meta-analysis of the European and Asian lnORs identified significant heterogeneity in the lnORs for rs157181 ($P_{het\ meta} = 0.031$), but not for rs60643069 ($P_{het\ meta} = 0.363$). (Note that rs141002831 was not tested in the Asian panels.) On balance, we consider this signal to be well supported across European populations, with some evidence of support across Asian populations.

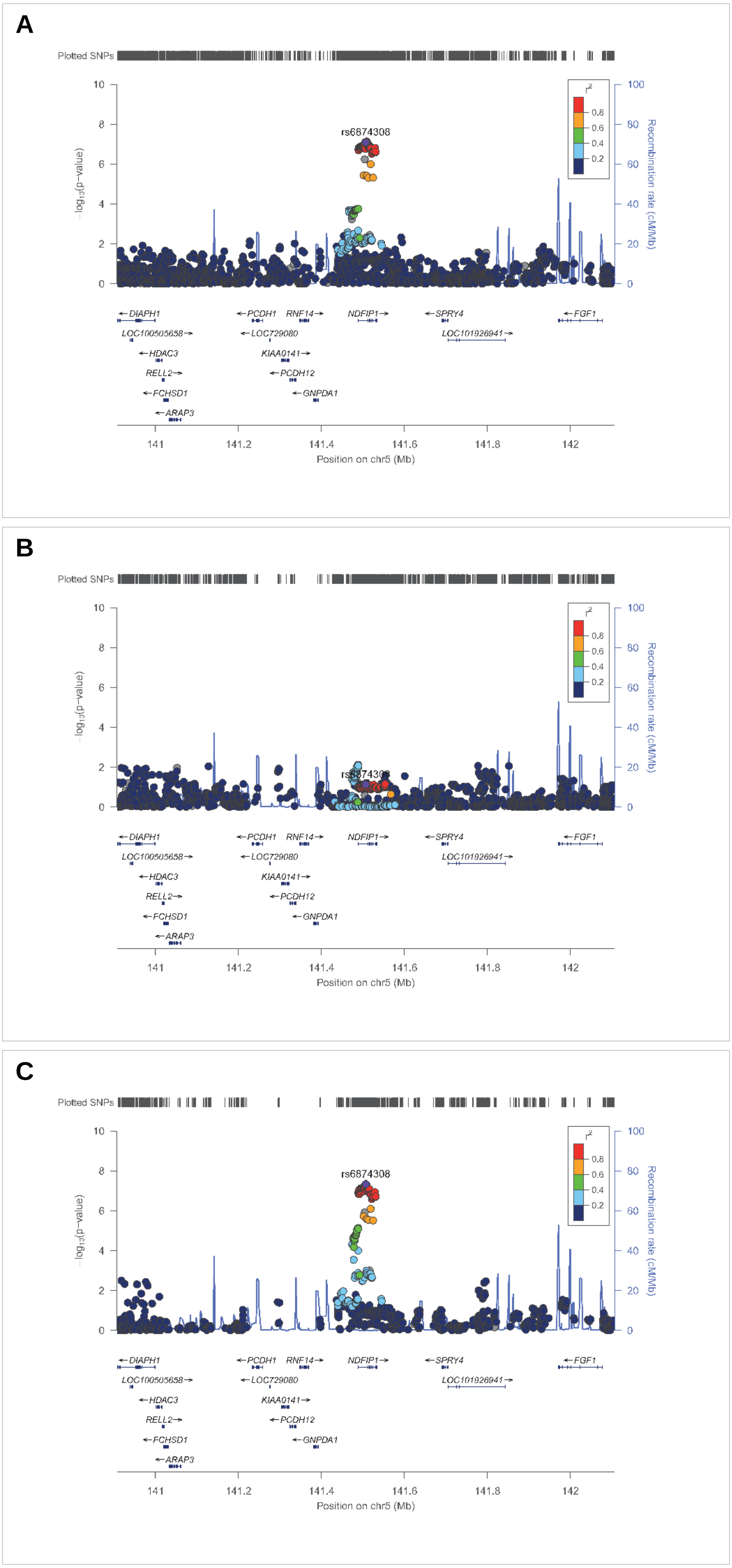


**Fig. S2.20: LocusZoom plots of newly identified PBC risk locus 5q31.3.** (A) European panels, (B) Asian panels, and (C) all panels combined. Association at this locus reached suggestive significance in the European panels (rs10062349 at 5:141,509,597; $P = 7.36\times10^{-8}$); nominal significance in the Asian panels (rs3761757 at 5:141,488,219; $P = 0.0075$); and genome-wide significance in all panels combined (rs6874308 at 5:141,506,911; $P = 4.67\times10^{-8}$). Permutation testing was not applied owing to futility. Nevertheless, fixed effect meta-analysis of the European and Asian lnORs did not identify significant heterogeneity in the lnORs for rs10062349 ($P_{het\ meta} = 0.253$); rs3761757 ($P_{het\ meta} = 0.545$); or rs6874308 ($P_{het\ meta} = 0.324$). On balance, we consider the signal at 5q31.3 to be supported in European populations. Conversely, the study was most likely under-powered to reliably confirm or refute association in Asian populations, or trans-ethnic heterogeneity, at this locus.

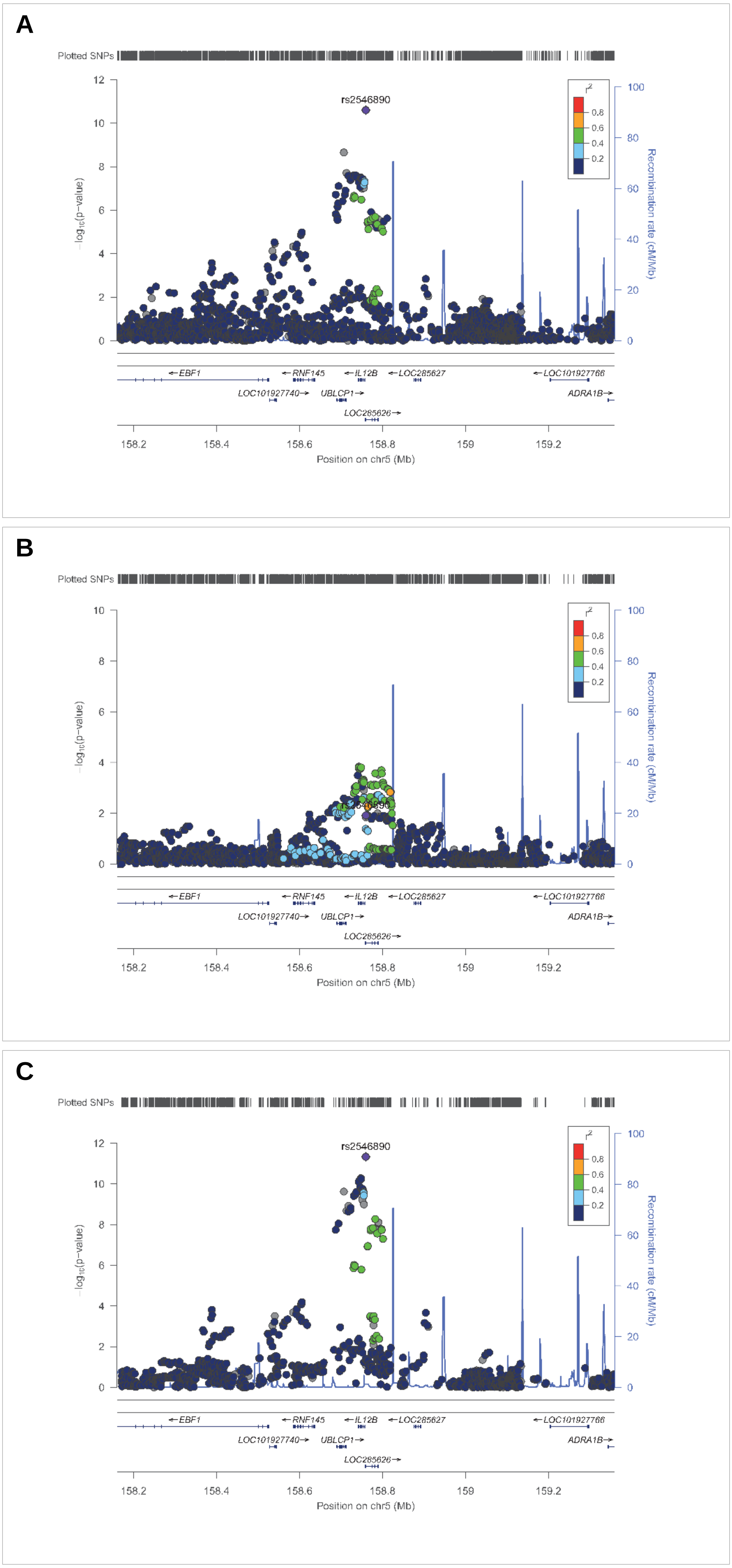


**Fig. S2.21: LocusZoom plots of the known PBC risk locus 5q33.3.** (A) European panels, (B) Asian panels, and (C) all panels combined. Association at this locus reached genome-wide significance in the European panels (rs2546890 at 5:158,759,900; $P = 2.51\times10^{-11}$); suggestive significance in the Asian panels (rs3213111 at 5:158,743,230; $P = 1.45\times10^{-4}$); and genome-wide significance in all panels combined (rs2546890; $P = 4.61\times10^{-12}$). Permutation testing suggested that the European signal at rs2546890 was corroborated by an Asian signal at rs3213111 ($P_{permutation} = 0.0061$), albeit not at the Bonferroni-corrected threshold of $P < 0.000893$ (correcting for 56 genome-wide significant loci). Furthermore, fixed effect meta-analysis of the European and Asian panels did not identify significant heterogeneity in the lnORs for rs3213111 ($P_{het\ meta} = 0.982$) or rs2546890 ($P_{het\ meta} = 0.279$). Thus, we consider the signal at 5q33.3 to be well supported across European populations, with some evidence of support across Asian populations.

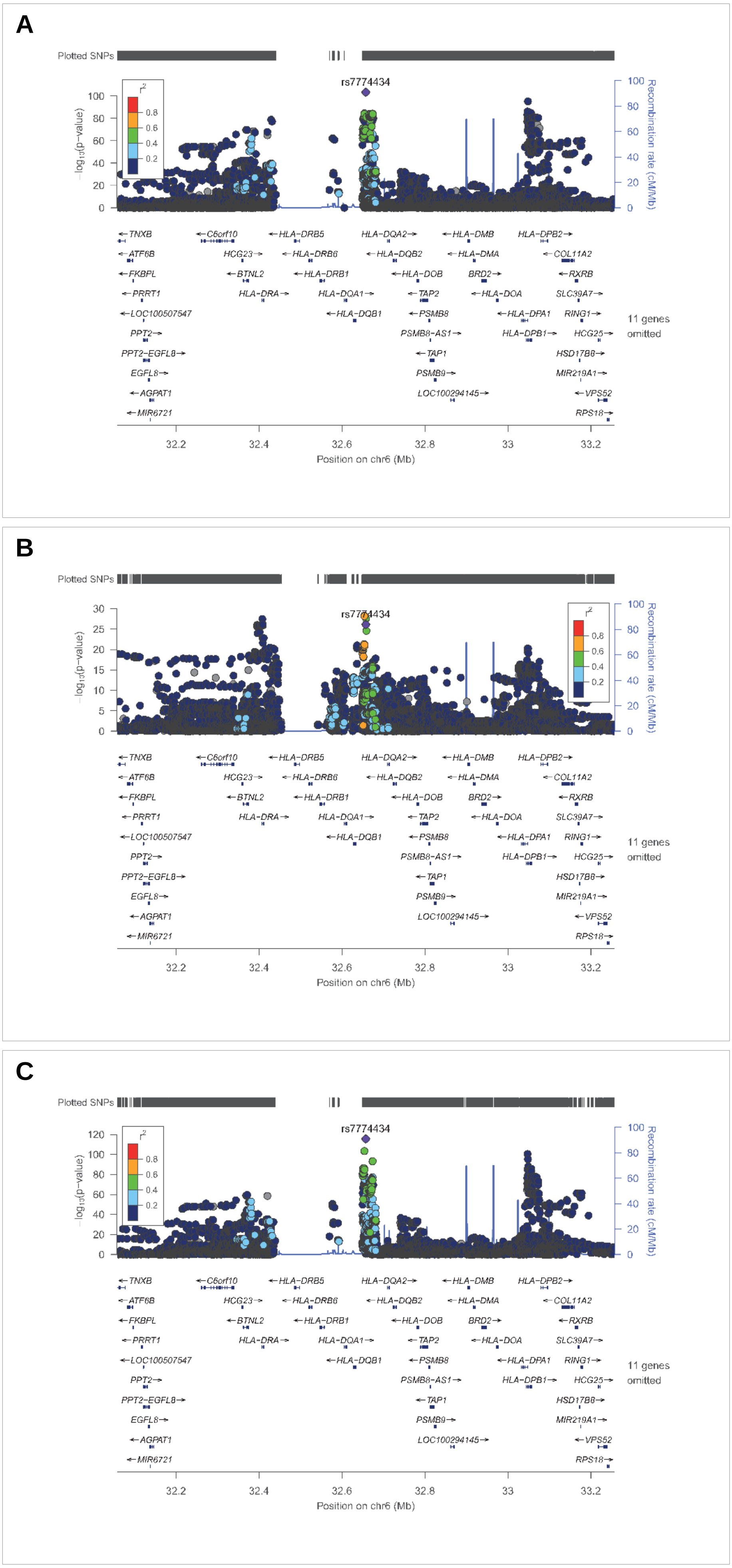


**Fig. S2.22: LocusZoom plots of the well-established PBC risk locus 6p21.32, the HLA locus.** (A) European panels, (B) Asian panels, and (C) all panels combined. An unequivocal association signal is evident in both the European and Asian panels.

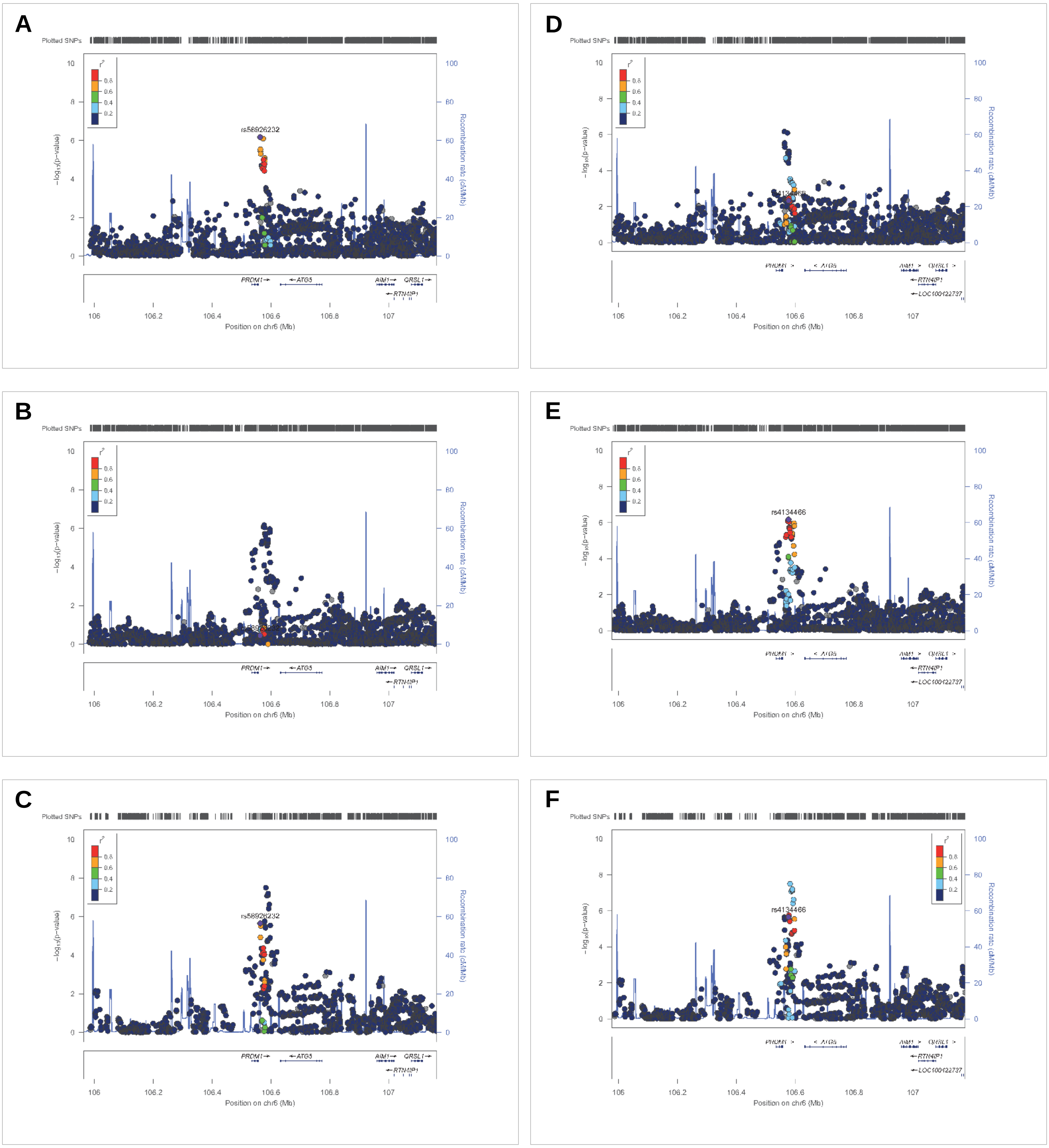
A
B
C
D
E
F
Plotted SNPs
rs58926232
rs4134466
r²
0.8
0.6
0.4
0.2
−log10(p−value)
Recombination rate (cM/Mb)
PRDM1
ATG5
AIM1
QRSL1
RTN4IP1
LOC100422737
Position on chr6 (Mb)

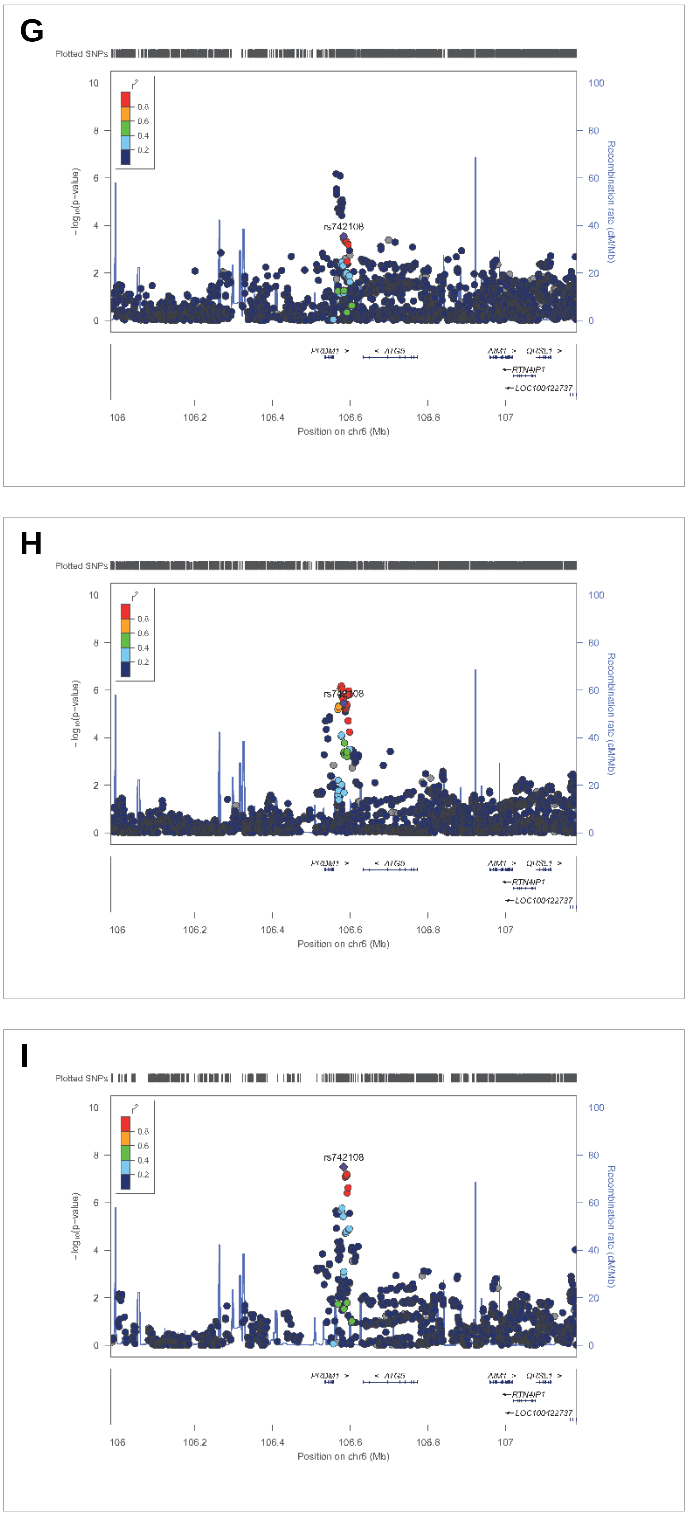


**Fig. S2.23: LocusZoom plots of the newly identified PBC risk locus 6q21.** (A, D, G) European panels, (B, E, H) Asian panels, and (C, F, I) all panels combined. Association at this locus reached suggestive significance in the European panels (rs58926232 at 6:106,563,612; $P = 6.75 \times 10^{-7}$; plot A); suggestive significance in the Asian panels (rs4134466 at 6:106,577,368; $P = 6.71 \times 10^{-7}$; plot E); and genome-wide significance in all panels combined (rs742108 at 6:106,582,920; $P = 3.16 \times 10^{-8}$). Permutation testing confirmed that the European signal at rs58926232 was corroborated by an Asian signal at rs4134466 ($P_{permutation}$ = 0.0001). Fixed effect meta-analysis of the European and Asian lnORs identified significant heterogeneity in the lnORs for rs4134466 ($P_{het\ meta}$ = 0.002), but not rs58926232 ($P_{het\ meta}$ = 0.126), or rs742108 ($P_{het\ meta}$ = 0.098) (plots G – I). On balance, we consider this signal to be well supported across both European and Asian populations.

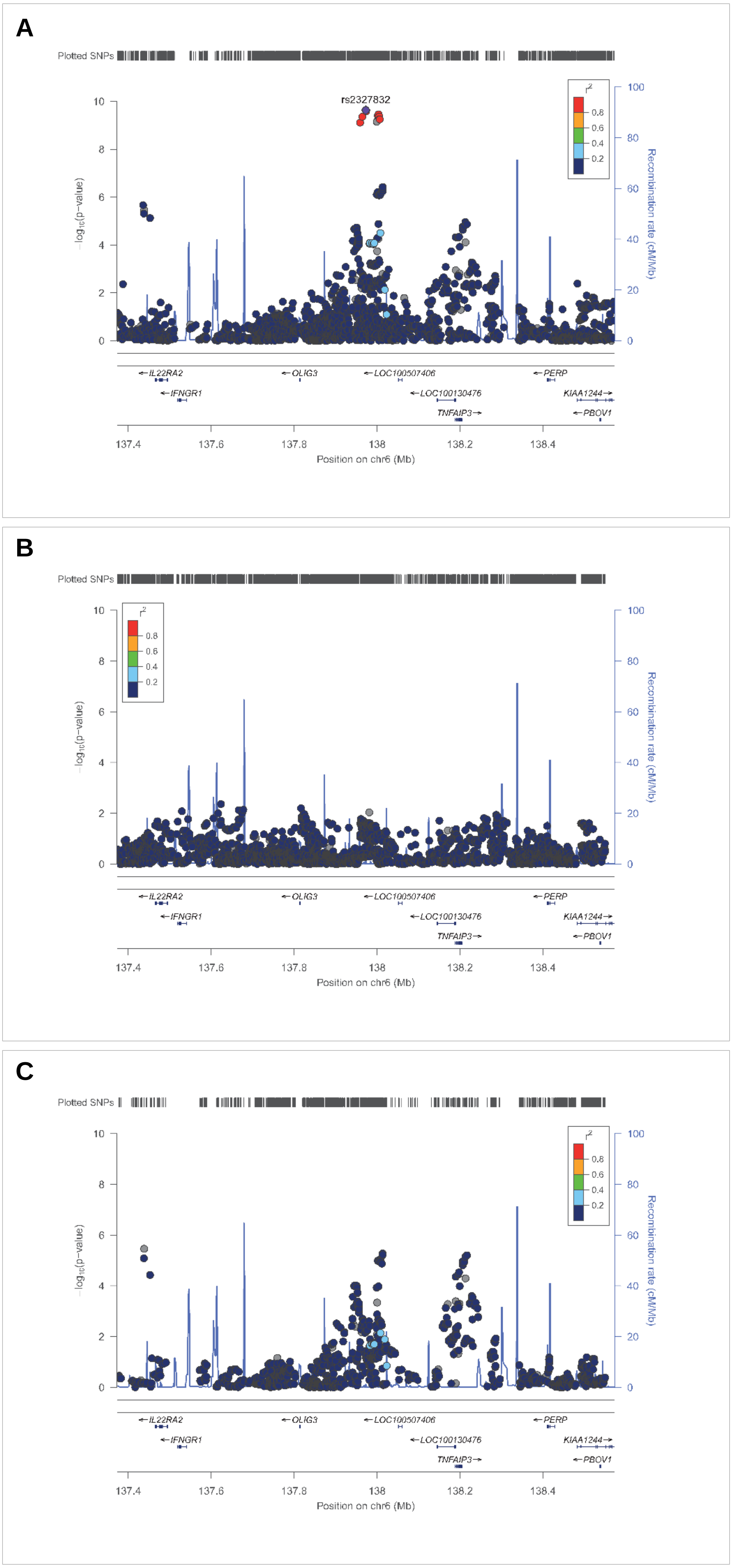


**Fig. S2.24: LocusZoom plots of the known PBC risk locus 6q23.3.** (A) European panels, (B) Asian panels, and (C) all panels combined. Association at this locus reached genome-wide significance in the European panels (rs2327832 at 6:137,973,068; $P = 2.31\times10^{-10}$); nominal significance in the Asian panels (rs35816149 at 6:137,623,819; $P = 0.0043$); and suggestive significance in all panels combined (rs59947391 at 6:137,438,765; $P = 3.43\times10^{-6}$). Permutation testing was not applied owing to futility. Nevertheless, fixed effect meta-analysis of the European and Asian lnORs did not identify significant heterogeneity in the lnORs for rs59947391 ($P_{het\ meta} = 0.538$). (Note that rs2327832 was not tested in the Asian panels, nor rs35816149 in the Asian panels.) On balance, we consider the signal at 6q23.3 to be well supported in European populations. Conversely, the study was most likely under-powered to reliably confirm or refute association in Asian populations, or trans-ethnic heterogeneity, at this locus.

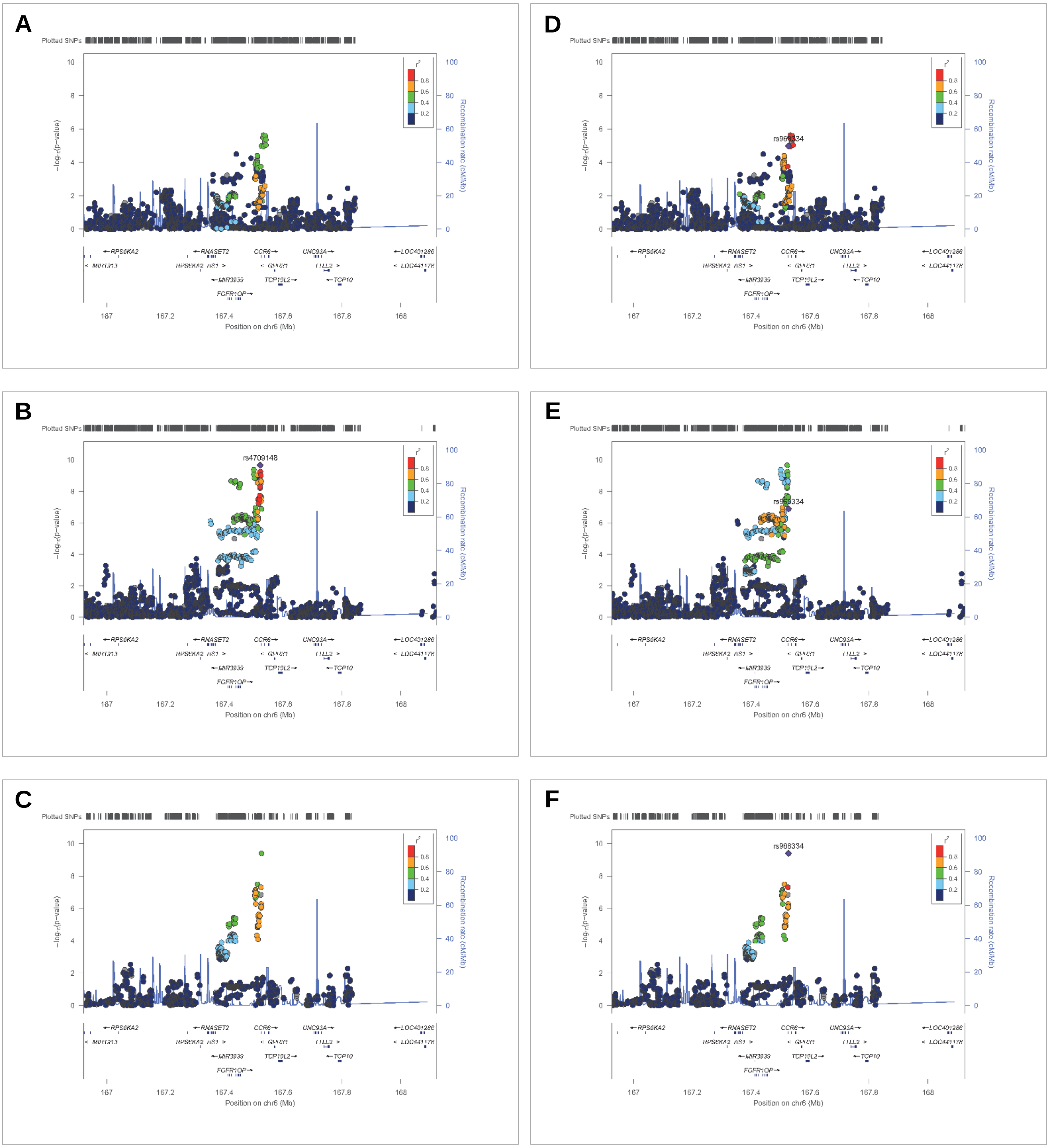


**Fig. S2.25: LocusZoom plots of the newly identified PBC risk locus 6q27.** (A, D) European panels, (B, E) Asian panels, and (C, F) all panels combined. An unequivocal association signal is evident in both the Asian and European panels. The lead variant in the Asian panels (rs4709148; P = $2.18\times10^{-10}$) was not interrogated in the European panels, but rs3093024 (11,117 BP away) reached P = $2.37\times10^{-6}$ ($P_{permutation}$ = 0.0001) in Europeans, with the top result (P = $3.98\times10^{-10}$) in combined panels seen at rs968334 (which lies 4420 BP from rs4709148), with only marginal evidence ($P_{het\ meta}$ =0.011) for heterogeneity seen between the Asian and European lnORs. Thus, we consider this signal to be well supported across both European and Asian populations.

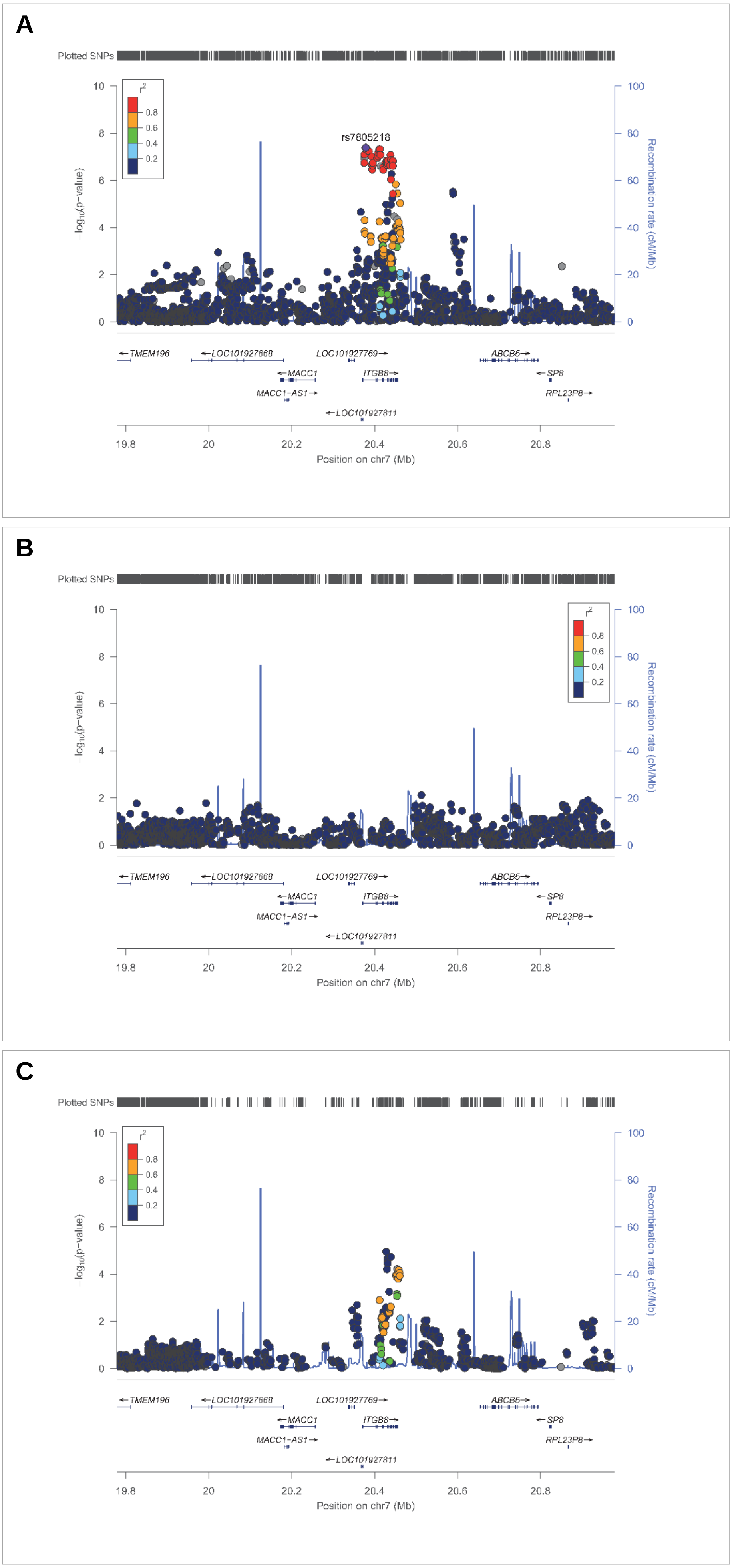


**Fig. S2.26: LocusZoom plots of the newly identified PBC risk locus 7p21.1.** (A) European panels, (B) Asian panels, and (C) all panels combined. Association at this locus reached genome-wide significance in the European panels (rs7805218 at 7:20,378,801; $P$ = 4.12×10$^{-8}$); nominal significance in the Asian panels (rs77984571 at 7:20,512,650; $P$ = 0.0075); and suggestive significance in all panels combined (rs7786537 at 7:20,427,776; $P$ = 1.12×10$^{-5}$). Permutation testing was not applied owing to futility. Nevertheless, fixed effect meta-analysis of the European and Asian lnORs did not identify significant heterogeneity in the lnORs for rs7786537 ($P_{\text{het meta}}$ = 0.183). (Note that rs7805218 was not tested in the Asian panels, nor rs77984571 in the European panels.) Thus, we consider the signal at 7p21.1 to be supported in European populations. Conversely, the study was most likely under-powered to reliably confirm or refute association in Asian populations, or trans-ethnic heterogeneity, at this locus.

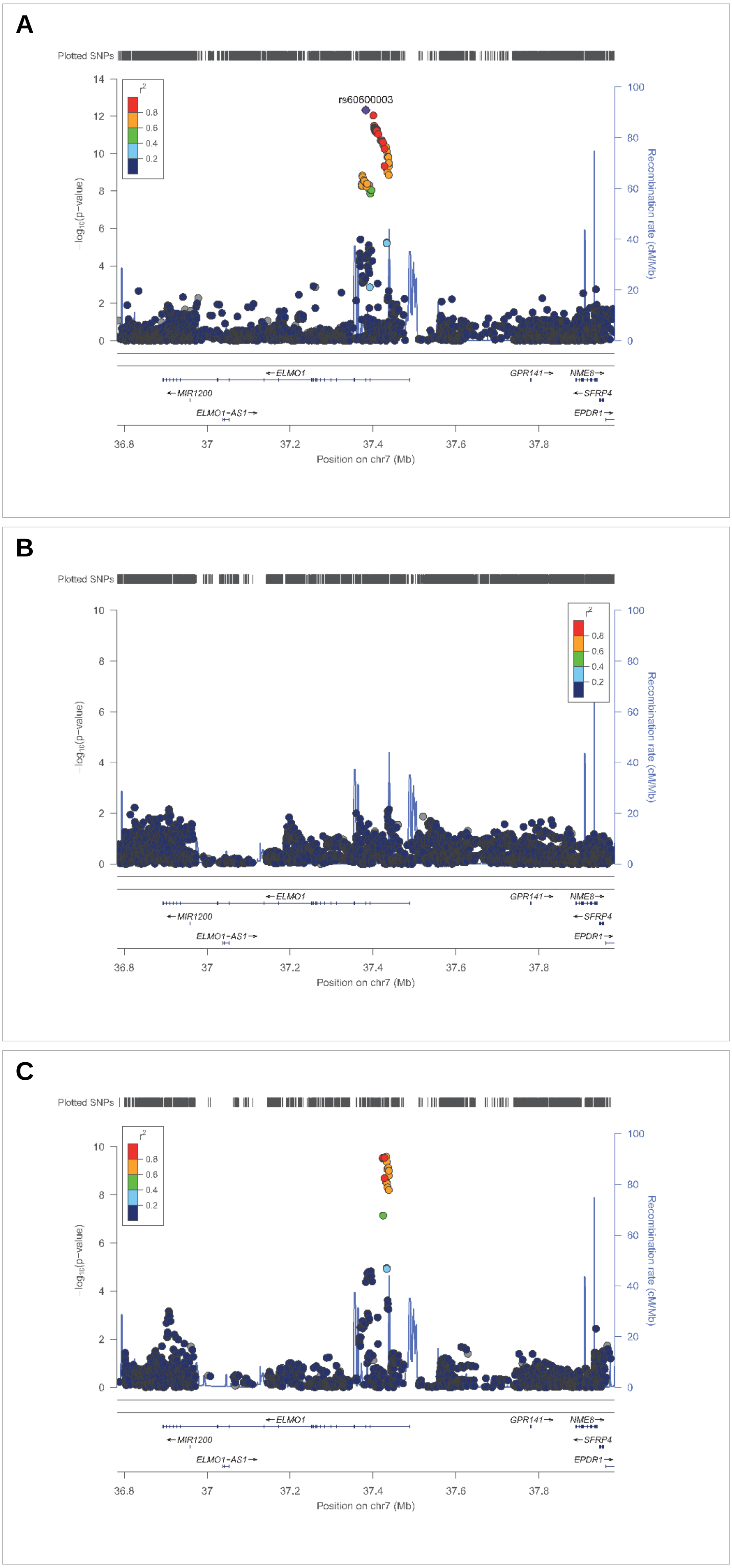


**Fig. S2.27: LocusZoom plots of the known PBC risk locus 7p14.1.** (A) European panels, (B) Asian panels, and (C) all panels combined. Association at this locus reached genome-wide significance in the European panels (rs60600003 at 7:37,382,465; $P$ = 4.70×10$^{-13}$); nominal significance in the Asian panels (rs7790608 at 7:36,825,122; $P$ = 0.0059); and genome-wide significance in all panels combined (rs10230459 at 7:37,431,559; $P$ = 2.59×10$^{-10}$). Permutation testing was not applied owing to futility. Nevertheless, fixed effect meta-analysis of the European and Asian lnORs did not identify significant heterogeneity in the lnORs for rs10230459 ($P_{\text{het meta}}$ = 0.165). (Note that rs60600003 was not tested in the Asian panels, nor rs7790608 in the European panels.) On balance, we consider the signal at 7p14.1 to be well supported in European populations. Conversely, the study was most likely under-powered to reliably confirm or refute association in Asian populations, or trans-ethnic heterogeneity, at this locus.

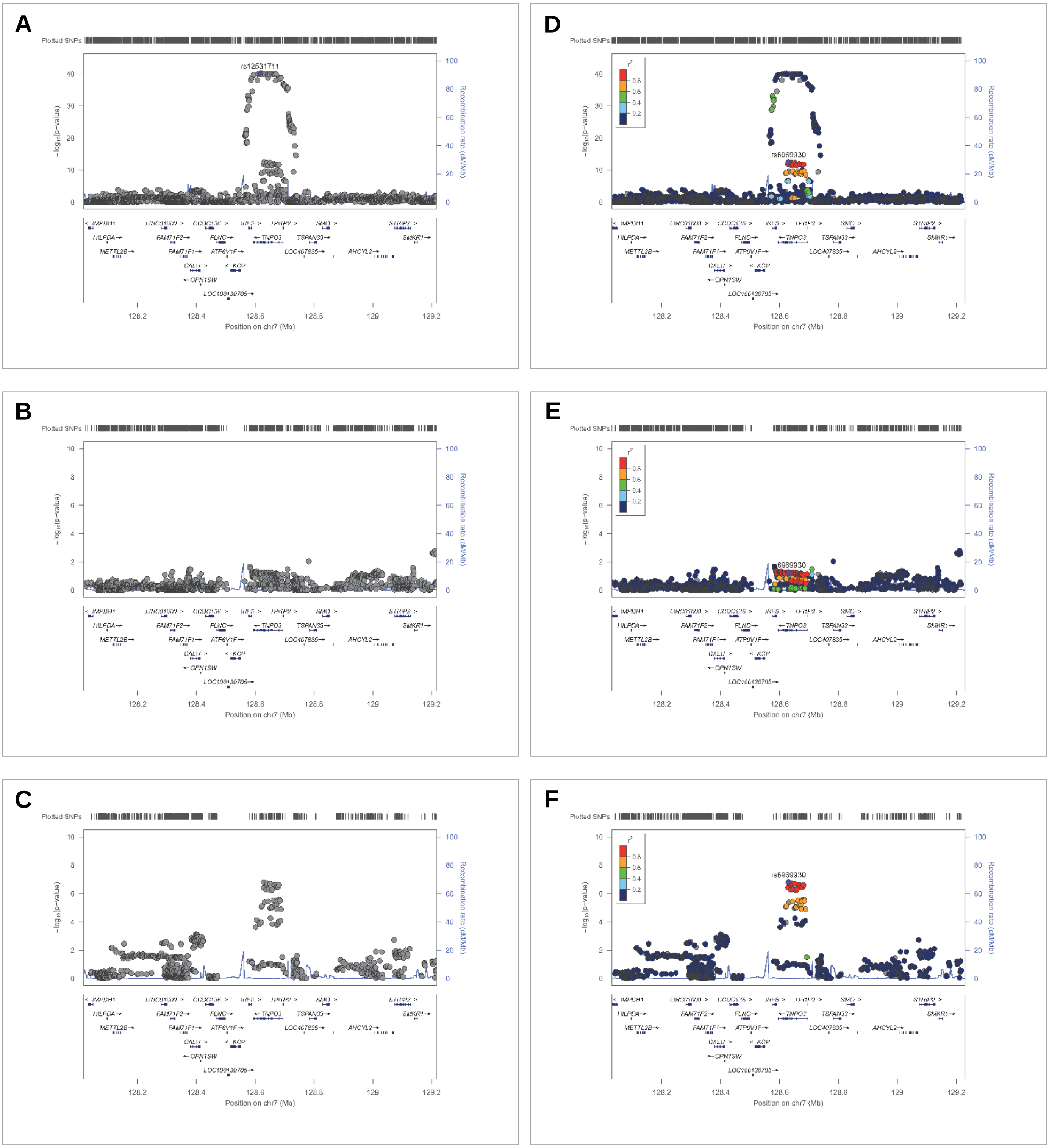


**Fig. S2.28: LocusZoom plots of the known PBC risk locus 7q32.1.** (A, D) European panels, (B, E) Asian panels, and (C, F) all panels combined. Association at this locus reached genome-wide significance in the European panels (rs12531711 at 7:128,617,466; P = $8.57\times10^{-41}$; plot A); nominal significance in the Asian panels (rs690686 at 7:129,211,489; P = 0.0016); and suggestive significance in all panels combined (rs6969930 at 7:128,630,313; P = $1.61\times10^{-7}$; plot F). Permutation testing was not applied owing to futility. Fixed effect meta-analysis of the European and Asian lnORs identified significant heterogeneity in the lnORs for rs690686 ($P_{het\ meta}$ = 0.005) and rs6969930 ($P_{het\ meta}$ = $1.06\times10^{-7}$). (Note that rs12531711 was not tested in the Asian panels.) On balance, we consider the signal at 7q32.1 to be well supported in European populations. The signal is not supported in Asian populations, however, and there might be genetic heterogeneity at this locus (c.f. **Suppl. Figure 7**).

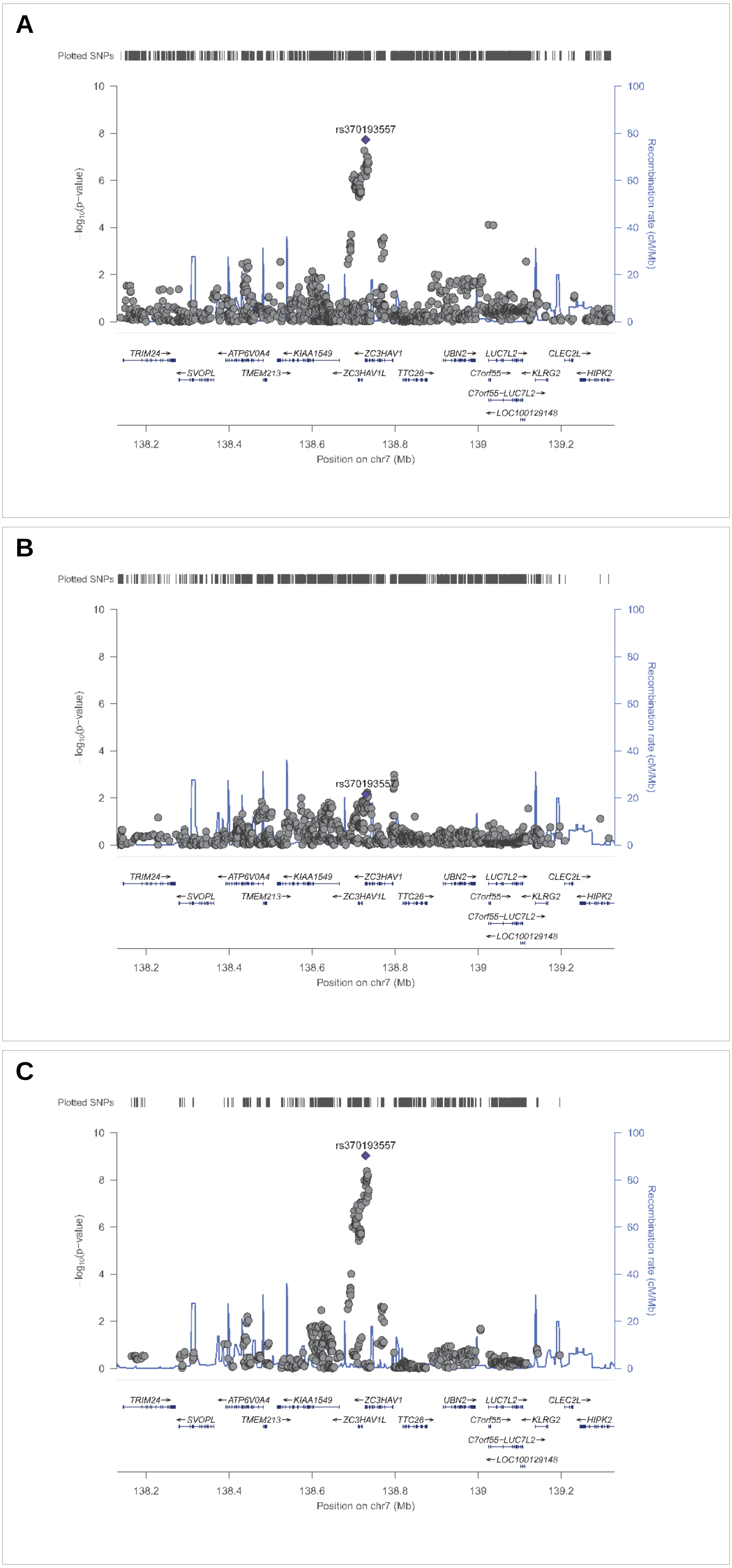


**Fig. S2.29: LocusZoom plots of the newly identified PBC risk locus 7q34.** (A) European panels, (B) Asian panels, and (C) all panels combined. Association at this locus reached genome-wide significance in the European panels (rs370193557 at 7:138,729,543; $P$ = 1.89×10$^{-8}$); nominal significance in the Asian panels (rs12056141 at 7:138,797,730; $P$ = 0.001); and genome-wide significance in all panels combined (rs370193557; $P$ = 9.37×10$^{-10}$). Permutation testing was not applied owing to futility. Nevertheless, fixed effect meta-analysis of the European and Asian lnORs did not identify significant heterogeneity in the lnORs for rs370193557 ($P_{het\ meta}$ = 0.917). (Note that rs12056141 was not tested in the European panels.) On balance, we considered the signal at 7q34 to be supported in European populations. Conversely, the study was most likely under-powered to reliably confirm or refute association in Asian populations, or trans-ethnic heterogeneity, at this locus.

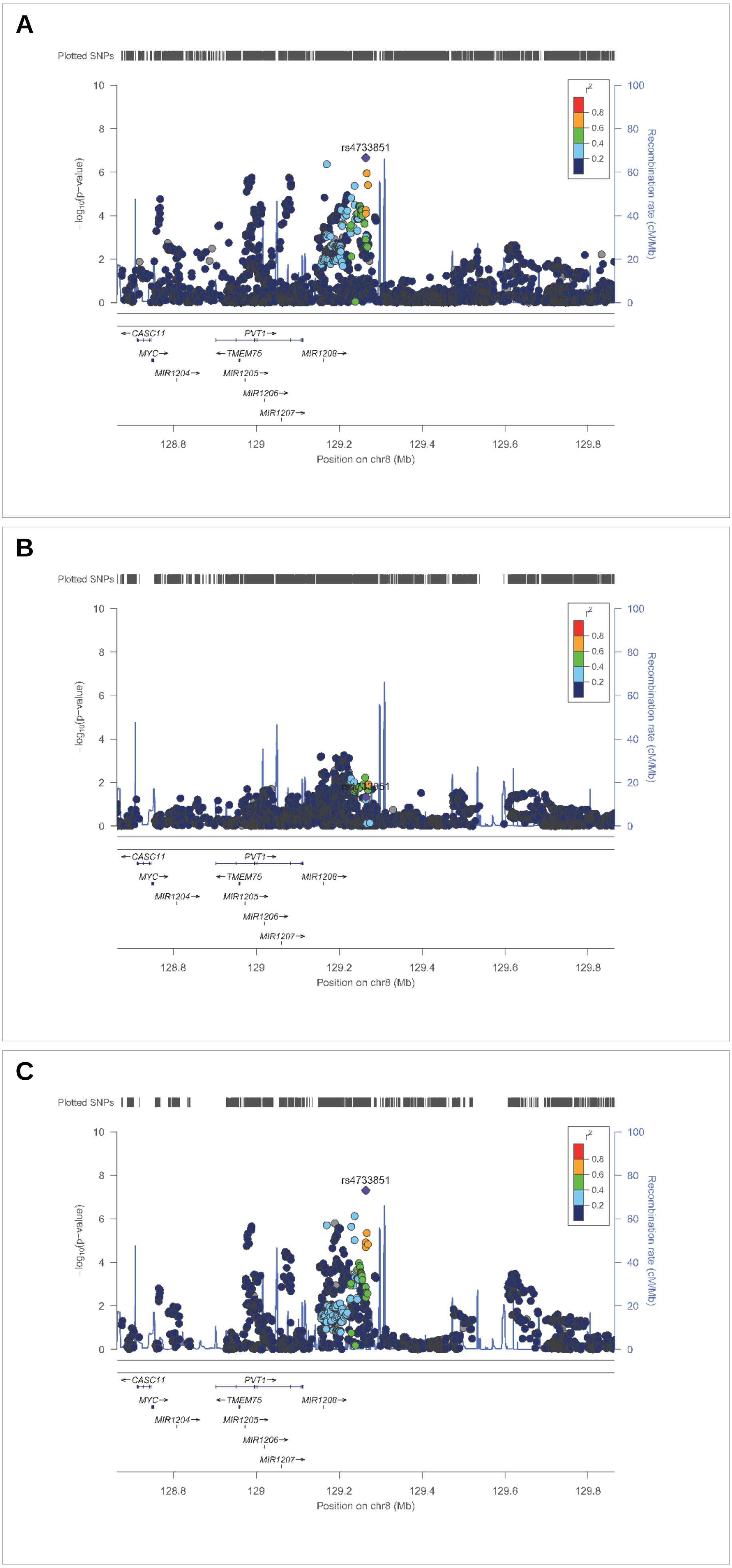


**Fig. S2.30: LocusZoom plots of the newly identified PBC risk locus 8q24.21.** (A) European panels, (B) Asian panels, and (C) all panels combined. Association at this locus reached suggestive significance in the European panels (rs4733851 at 8:129,264,420; $P = 2.18\times10^{-7}$); nominal significance in the Asian panels (rs1902780 at 8:129,211,788; $P = 5.51\times10^{-4}$); and genome-wide significance in all panels combined (rs4733851; $P = 4.98\times10^{-8}$). Permutation testing was not applied owing to futility. Nevertheless, fixed effect meta-analysis of the European and Asian lnORs did not identify significant heterogeneity in the lnORs for rs4733851 ($P_{het\ meta} = 0.895$) or rs1902780 ($P_{het\ meta} = 0.496$). On balance, we consider the signal at 8q24.21 to show some level of support across European and Asian populations. The study is most likely under-powered, however, to reliably test trans-ethnic heterogeneity at this locus.

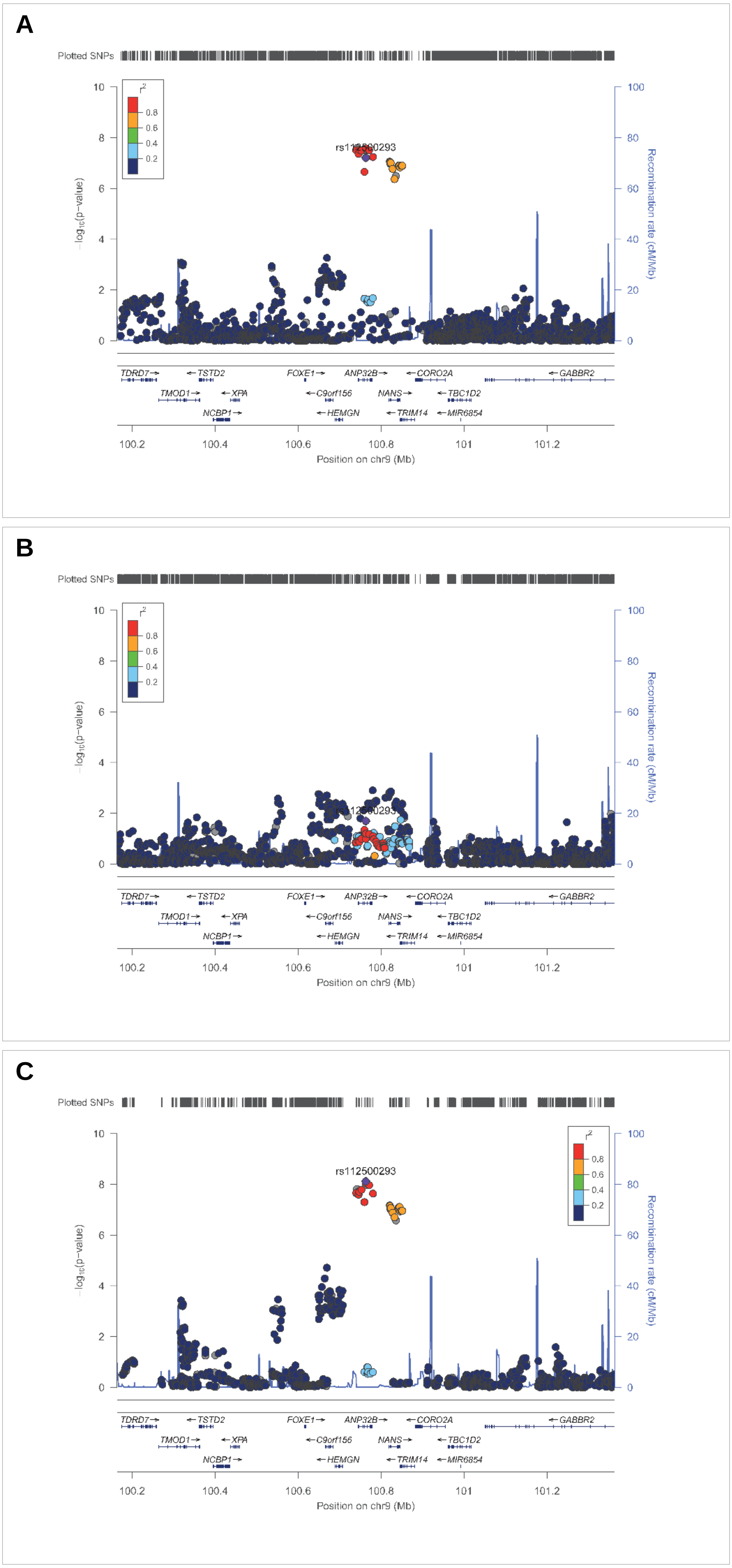


**Fig. S2.31: LocusZoom plots of the newly identified PBC risk locus 9q22.33.** (A) European panels, (B) Asian panels, and (C) all panels combined. Association at this locus reached genome-wide significance in the European panels (rs11390003 at 9:100,741,912; $2.56\times10^{-8}$); nominal significance in the Asian panels (rs10283737 at 9:100,780,063; $P = 0.0012$); and genome-wide significance in all panels combined (rs112500293 at 9:100,763,455; $P = 7.63\times10^{-9}$). Permutation testing was not applied owing to futility. Nevertheless, fixed effect meta-analysis of the European and Asian lnORs did not identify significant heterogeneity in the lnORs for rs11390003 ($P_{\text{het meta}} = 0.646$) or rs112500293 ($P_{\text{het meta}} = 0.766$). (Note that rs10283737 was not tested in the European panels.) On balance, we consider the signal at 9q22.33 to be supported in the European panel. Conversely, the study was most likely under-powered to reliably confirm or refute association in Asian populations, or trans-ethnic heterogeneity, at this locus.

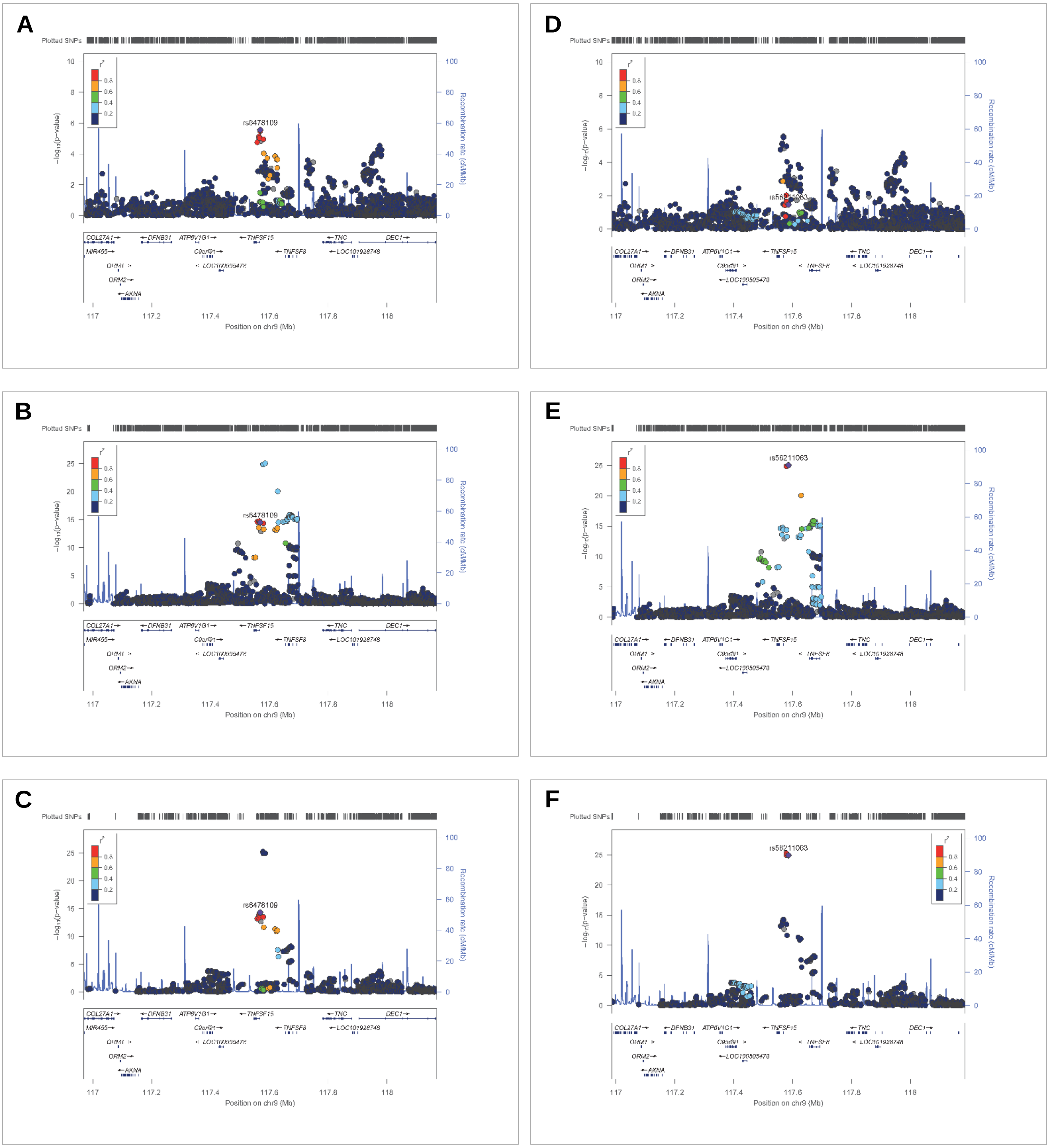


**Fig. S2.32**: LocusZoom plots of the known PBC risk locus 9q32 in the European (A, D), Asian (B, E), and combined (C, F) panels. At 9q32, in the vicinity of TNFSF15, a strikingly strong Asian signal (lnOR=0.449; OR=1.567; $P = 9.12\times10^{-26}$) at rs56211063 showed a much weaker signal ($P = 0.034$) in Europeans; however, this difference in significance can largely be attributed to the different allele frequencies of the disease-associated (C) allele in the different populations. This variant is at frequency 0.265 in the 1000 Genomes Project East Asian (EAS) samples but is at frequency only 0.0119 in the 1000 Genomes Project European (EUR) samples. Focusing on the two largest of our own European panels and the one Asian (Japanese) panel for which we had individual-level data (**Table 1**), we estimated the allele frequencies in our own data sets to be 0.00236 in the Canadian-UK panel, 0.00151 in the WTCCC3 panel and 0.507 in the Japanese panel, consistent with the expectation that the Asian panels should be much better powered than the European panels to detect effects at rs56211063, even if the effect sizes (lnORs) are similar across populations. Indeed,

fixed effect meta-analysis of our Asian and European panels did not identify heterogeneity in the lnORs at rs56211063 (P = 0.949), despite the striking difference in significance observed.

Interestingly, in Europeans, the strongest signal in the TNFSF15 region ($P = 2.79\times10^{-6}$; $P_{permutation}$ <0.0001) is seen at rs6478109 (**Supplementary Table 1**), 17,131 BP away from rs56211063. The variant, rs6478109, achieves $P = 3.33\times10^{-15}$ in the Asian panels, but its significance here can be largely attributed to its strong LD with rs56211063 ($r^2$=0.556; D'=1 in the Japanese panel). Once the effect of rs56211063 was accounted for (in either conditional or stratified logistic regression analysis), we did not find any evidence of an additional effect at rs6478109 in the Japanese panel (P > 0.4). However, this could primarily be due to a lack of power in the smaller Japanese panel, as the OR for rs6478109 conditioned on rs56211063 in the Japanese panel (OR=0.93) is not, in fact, significantly different from that seen (OR=0.88, $P_{heterogeneity}$ = 0.57 and OR=0.9, $P_{heterogeneity}$ = 0.76 respectively) in the Canadian-UK or the WTCCC3 panels.

In Europeans, the LD between rs6478109 and rs56211063 is much weaker ($r^2$=0.00112; D'=1 in the Canadian-UK panel; $r^2$=0.000739; D'=1 in the WTCCC3 panel), meaning that the separate effects of these two SNPs can be disentangled more easily. Once any effect of rs56211063 is accounted for (in either conditional or stratified logistic regression analysis), we still see significant effects at rs6478109 ($P = 7.04\times10^{-6}$ in the Canadian-UK and P = 0.013 in the WTCCC3 panels, respectively), suggesting the existence of a possible weaker second signal (over and above any effect at rs56211063) in Europeans.

Thus, in summary for this locus, it seems that the strong effect (OR=1.567; $P = 9.12\times10^{-26}$) operating in Asians at rs56211063 may, in fact, also be operating in Europeans, but the risk allele occurs at a frequency too low to be detectable at convincing significance levels, while the weaker second effect operating at rs6478109 in Europeans may, in fact, also be operating in Asians, but our current Asian sample size is not large enough to provide sufficient power for its detection.

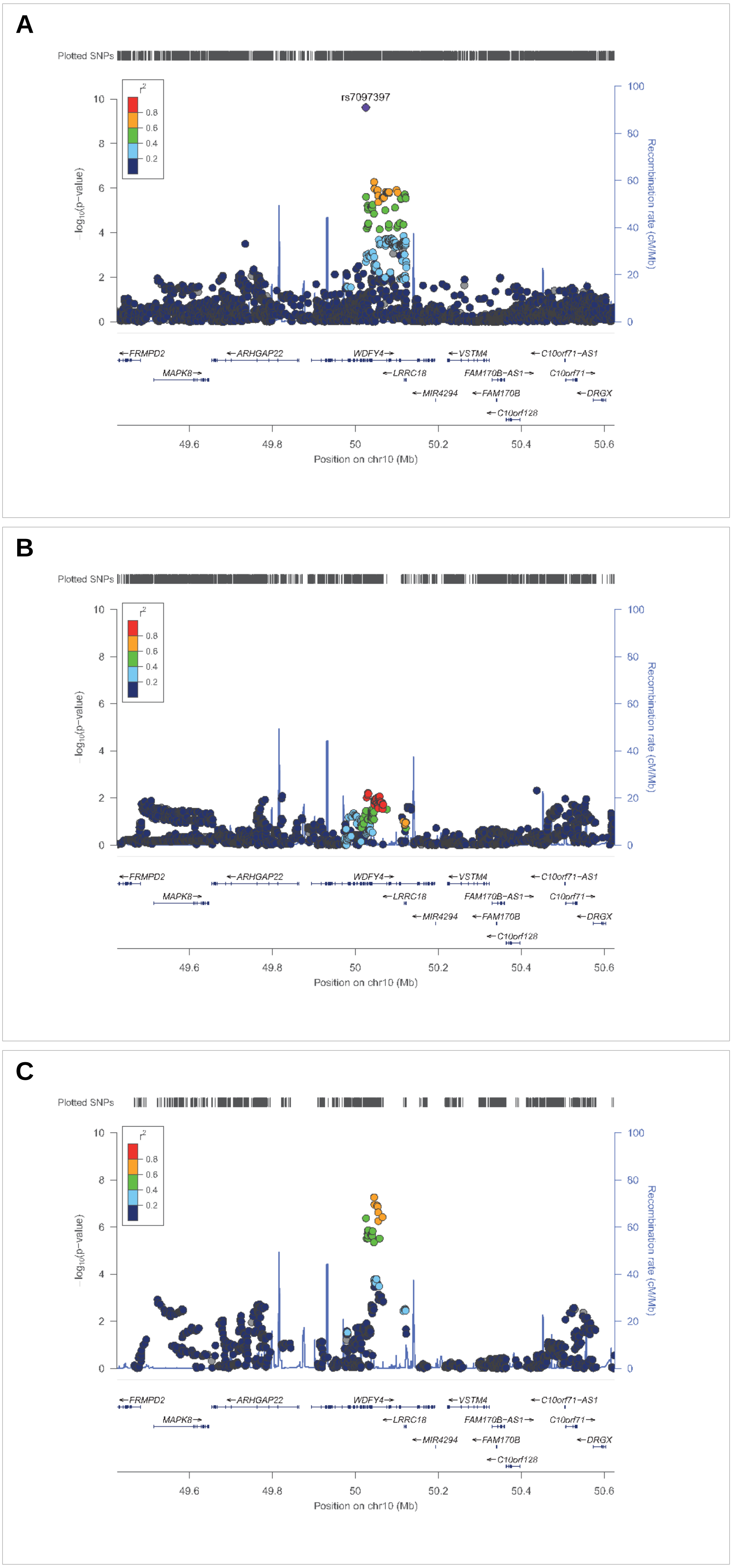


**Fig. S2.33: LocusZoom plots of the newly identified PBC risk locus 10q11.23.** (A) European panels, (B) Asian panels, and (C) all panels combined. Association at this locus reached genome-wide significance in the European panels (rs7097397 at 10:50,025,396; $P = 2.42\times10^{-10}$); nominal significance in the Asian panels (rs76129863 at 10:50,437,561; $P = 0.0048$); and genome-wide significance in all panels combined (rs7922169 at 10:50,045,456; $P = 5.47\times10^{-8}$). Permutation testing was not applied owing to futility. Nevertheless, fixed effect meta-analysis of the European and Asian lnORs did not identify significant heterogeneity in the lnORs for rs7922169 ($P_{het\ meta} = 0.841$). (Note that rs7097397 was not tested in the Asian panels, nor rs76129863 in the European panels.) On balance, we consider the signal at 10q11.23 to be supported in European populations. Conversely, the study was most likely under-powered to reliably confirm or refute association in Asian populations, or trans-ethnic heterogeneity, at this locus.

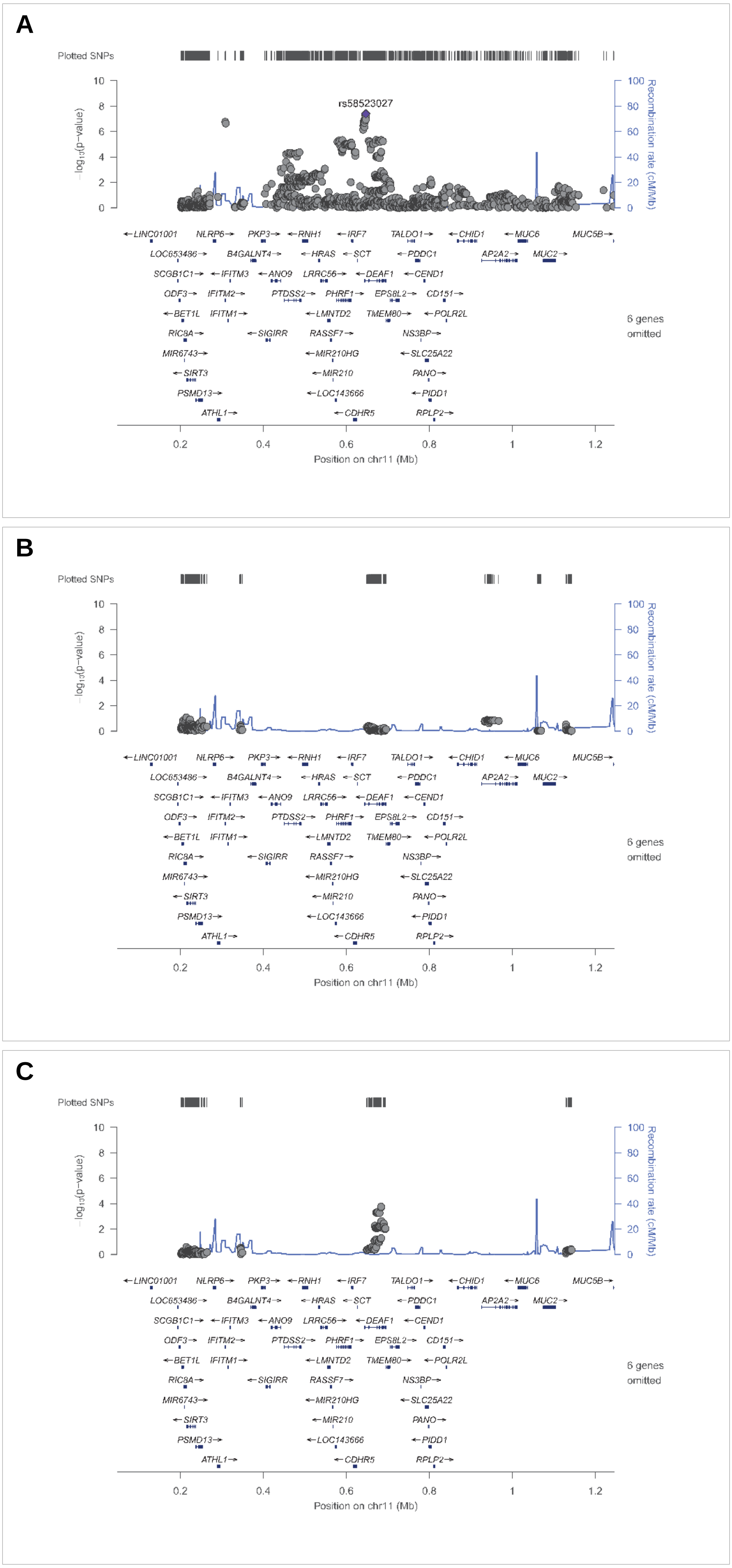


**Fig. S2.34: LocusZoom plots of the newly identified PBC risk locus 11p15.5.** (A) European panels, (B) Asian panels, and (C) all panels combined. Association at this locus reached genome-wide significance in the European panels (rs58523027 at 11:646,986; $P = 4.00\times10^{-8}$), and nominal significance in all panels combined (rs9667500 at 11:683,761; $P = 1.74\times10^{-4}$). No significant association was identified in the Asian panels (rs3216 at 11:214,421; $P = 0.082$). Permutation testing was not applied owing to futility. Nevertheless, fixed effect meta-analysis of the European and Asian lnORs did not identify significant heterogeneity in the lnORs for rs3216 ($P_{het\ meta} = 0.160$) or rs9667500 ($P_{het\ meta} = 0.119$). (Note that rs58523027 was not tested in the Asian panels.) On balance, we consider the signal at 11p15.5 to be supported in European populations. Conversely, the study was most likely under-powered to reliably confirm or refute association in Asian populations, or trans-ethnic heterogeneity, at this locus.

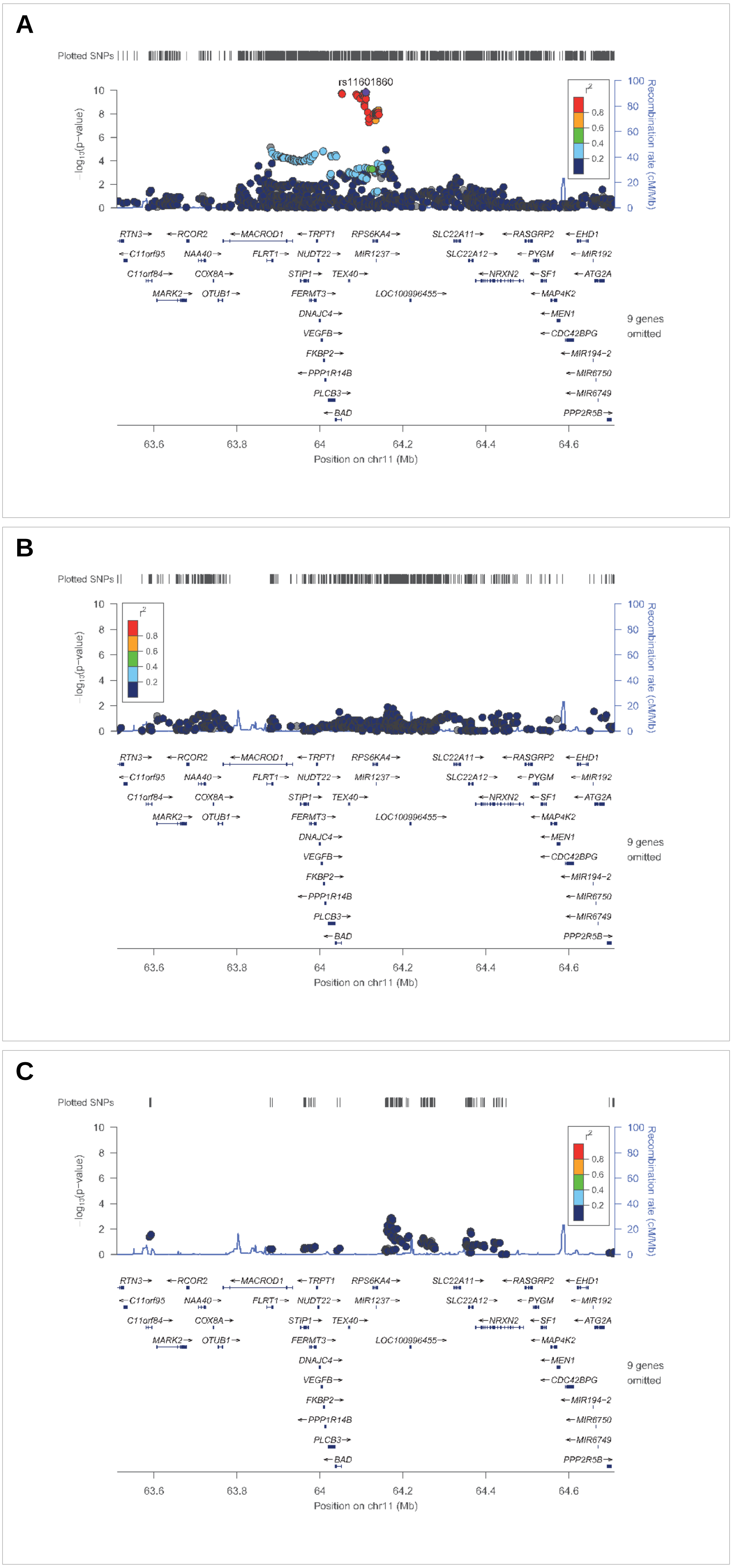


**Fig. S2.35: LocusZoom plots of the known PBC risk locus 11q13.1.** (A) European panels, (B) Asian panels, and (C) all panels combined. Association at this locus reached genome-wide significance in the European panels (rs11601860 at 11:64,110,422; $P = 1.45\times10^{-10}$); nominal significance in the Asian panels (rs750832 at 11:64,163,302; $P = 0.013$); and nominal significance in all panels combined (rs521319 at 11:64,172,031; $P = 1.34\times10^{-3}$). Permutation testing was not applied owing to futility. Nevertheless, fixed effect meta-analysis of the European and Asian lnORs did not identify significant heterogeneity in the lnORs for rs750832 ($P_{\text{het meta}} = 0.130$) or rs521319 ($P_{\text{het meta}} = 0.721$). (Note that rs11601860 was not tested in the Asian panel.) On balance, we consider the signal at 11q13.1 to be well supported in European populations. Conversely, the study was most likely under-powered to reliably confirm or refute association in Asian populations, or trans-ethnic heterogeneity, at this locus.

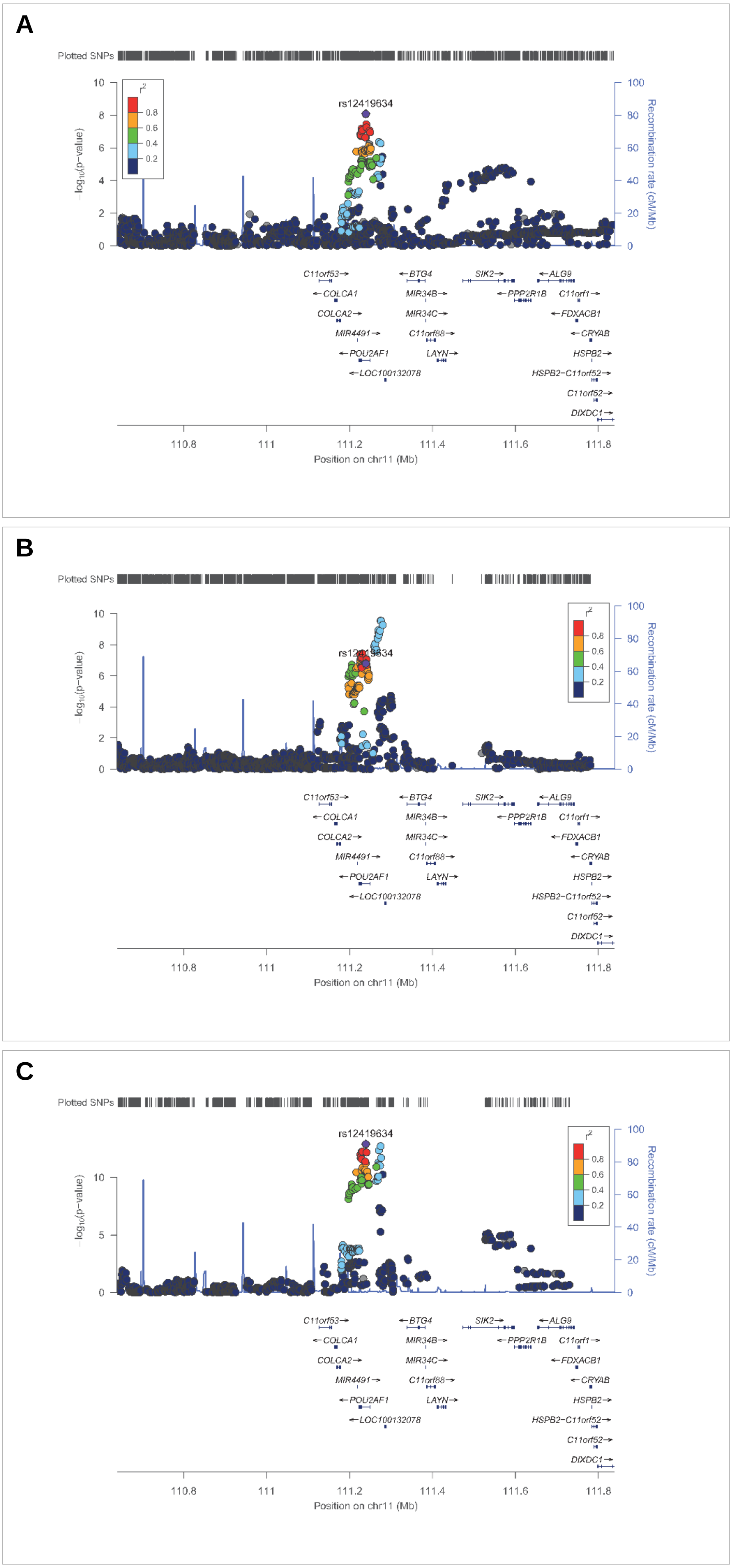


**Fig. S2.36: LocusZoom plots of known PBC risk locus 11q23.1.** (A) European panels, (B) Asian panels, and (C) all panels combined. Association at this locus reached genome-wide significance in the European panels (rs12419634 at 11:111,239,365; $P = 8.28 \times 10^{-9}$), Asian panels (rs4938534 at 11:111,275,133; $P = 2.82 \times 10^{-10}$), and all panels combined (rs12419634; $P = 1.22 \times 10^{-13}$). Permutation testing confirmed that the European signal at rs12419634 was corroborated by an Asian signal at rs4938534 ($P_{permutation} < 0.0001$). Fixed effect meta-analysis of the European and Asian lnORs identified significant heterogeneity in the lnORs for rs4938534 ($P_{het\ meta} = 0.003$), but not for rs12419634 ($P_{het\ meta} = 0.062$). On balance, we consider this signal to be well supported across both European and Asian populations.

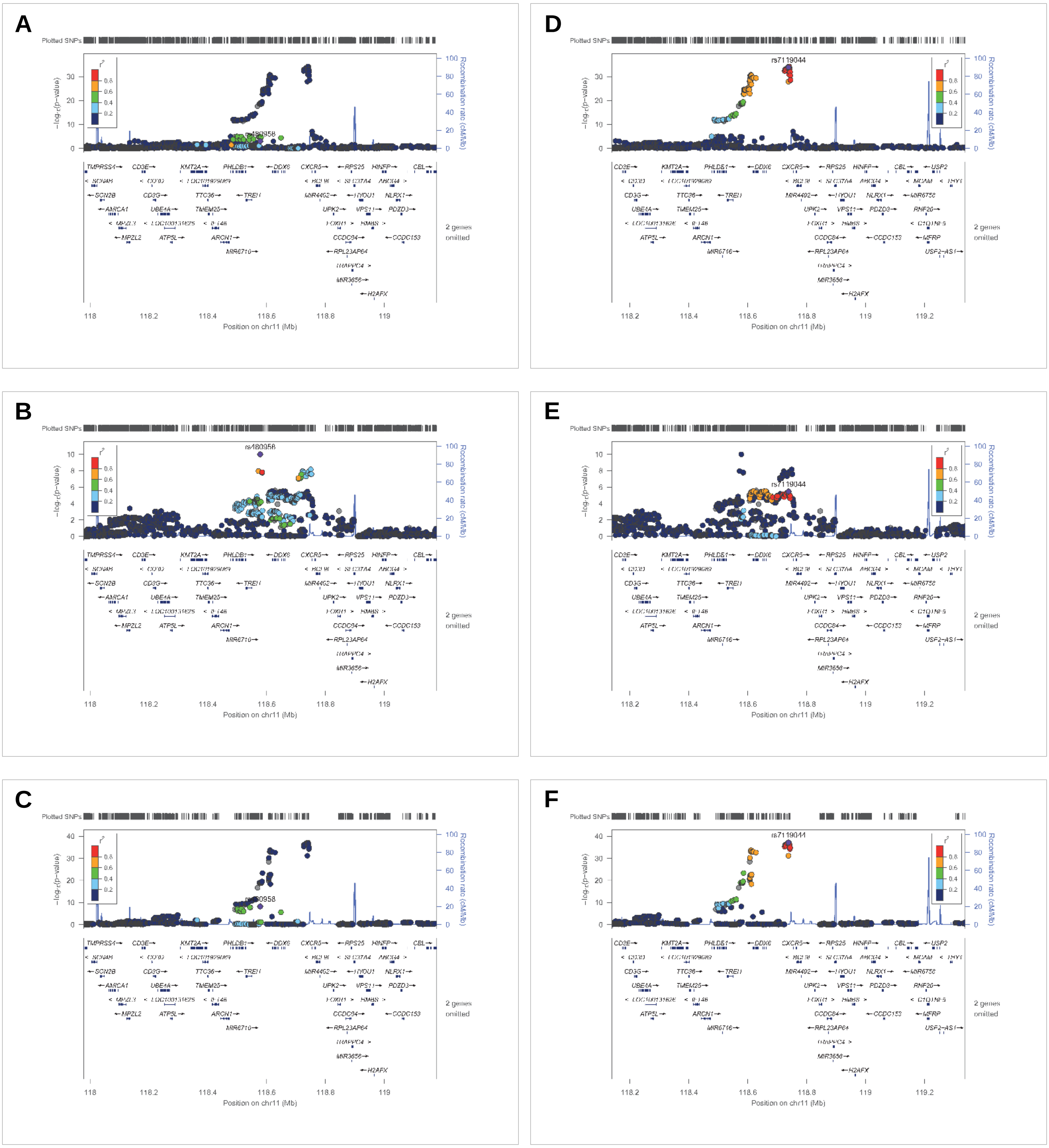


**Fig. S2.37: LocusZoom plots of the known PBC risk locus 11q23.3.** (A, D) European panels, (B, E) Asian panels, and (C, F) all panels combined. Association at this locus reached genome-wide significance in the European panels (rs201150316 at 11:118,740,104; $P = 5.16 \times 10^{-35}$); Asian panels (rs480958 at 11:118,577,990; $P = 1.01 \times 10^{-10}$); and combined panels (rs7119044 at 11:118,738,298; $P = 6.85 \times 10^{-38}$). Permutation testing at this locus confirmed that the European signal at rs201150316 was corroborated by an Asian signal at rs480958 ($P_{permutation} < 0.0001$). Fixed effect meta-analysis identified significant heterogeneity in the lnORs for rs480958 ($P_{het\ meta} = 2.97 \times 10^{-5}$), but not for rs201150316 ($P_{het\ meta} = 0.373$) or rs7119044 ($P_{het\ meta} = 0.533$). On balance, we consider this locus to be well supported across both European and Asian populations.

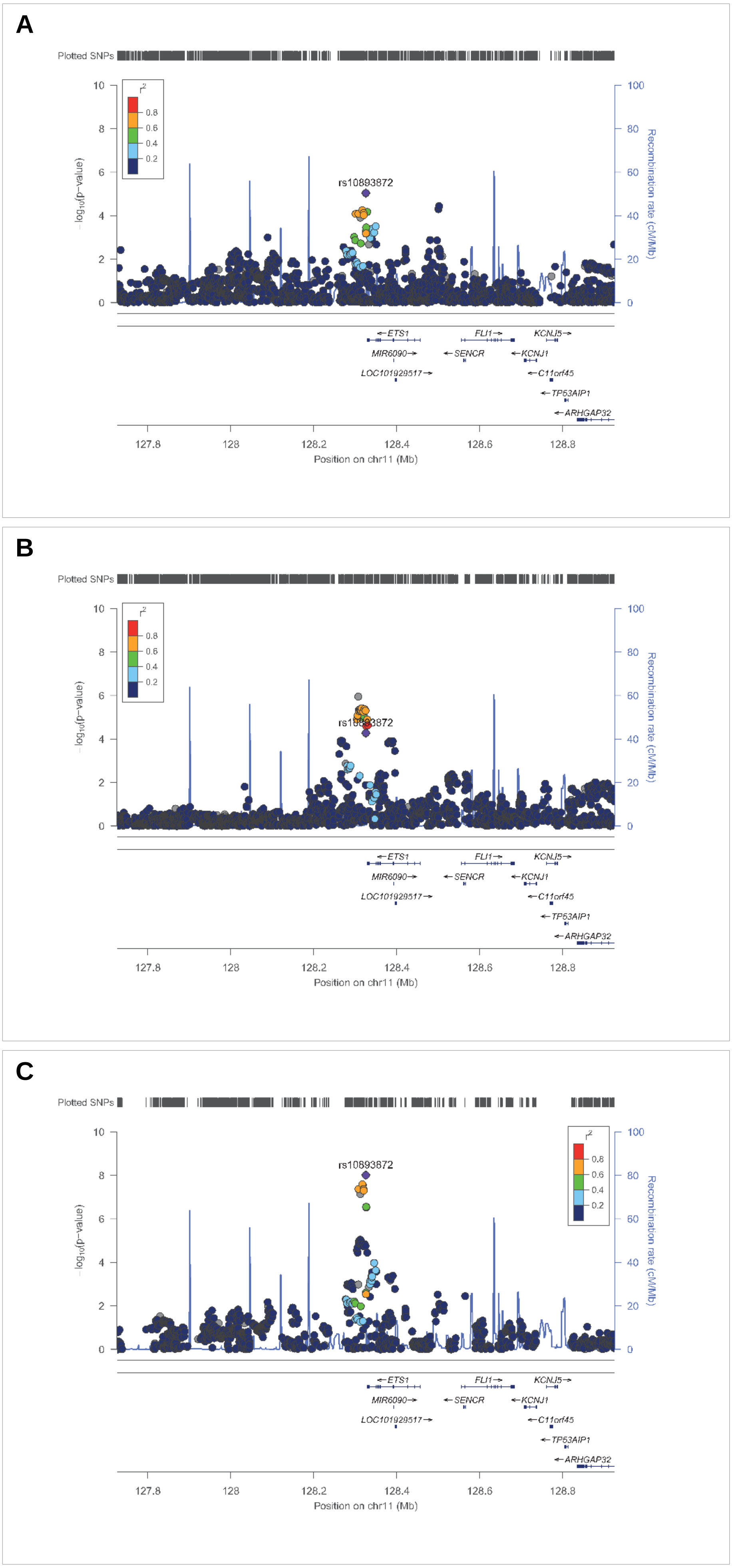


**Fig. S2.38**: LocusZoom plots of the newly identified PBC risk locus 11q24.3. (A) European panels, (B) Asian panels, and (C) all panels combined. Association at this locus reached suggestive significance in the European panels (rs10893872 at 11:128325553; $P$ = 9.07×10$^{-6}$); suggestive significance in the Asian panels (rs11430718 at 11:128,307,445; $P$ = 1.11×10$^{-6}$); and genome-wide significance in all panels combined (rs10893872; $P$ = 9.77×10$^{-9}$). Permutation testing at this locus confirmed that the European signal at rs10893872 was corroborated by an Asian signal at rs11430718 ($P_{permutation}$ <0.0001). Fixed effect meta-analysis of the European and Asian lnORs identified significant heterogeneity in the lnORs for rs11430718 ($P_{het\ meta}$ = 2.59×10$^{-4}$), but not for rs10893872 ($P_{het\ meta}$ = 0.176).

Further investigation of this locus indicated that while rs10893872 was somewhat significant in both the European panels ($P$ = 9.07×10$^{-6}$) and the Asian panels ($P$ = 5.28×10$^{-5}$), rs11430718 was somewhat significant in the Asian panels ($P$ = 1.11×10$^{-6}$) but not at all significant in the European panels ($P$ = 0.34). A possible explanation is that rs11430718 and rs10893872 are in reasonably strong LD in Asians ($r^2$ = 0.38 in our Japanese cohort, for which we had individual level data) and thus likely to mark the same signal, whereas these variants are not in LD ($r^2$ = 0.007) in our largest European cohorts (New Canadian-UK and WTCCC3). Therefore, our results are consistent with a scenario in which rs10893872 (or a variant in LD with it) is the true causal variant, with a similar signal evident at rs11430718 in Asian populations resulting from LD between rs11430718 and rs10893872.

On balance, we consider this signal to be well supported across both European and Asian panels.

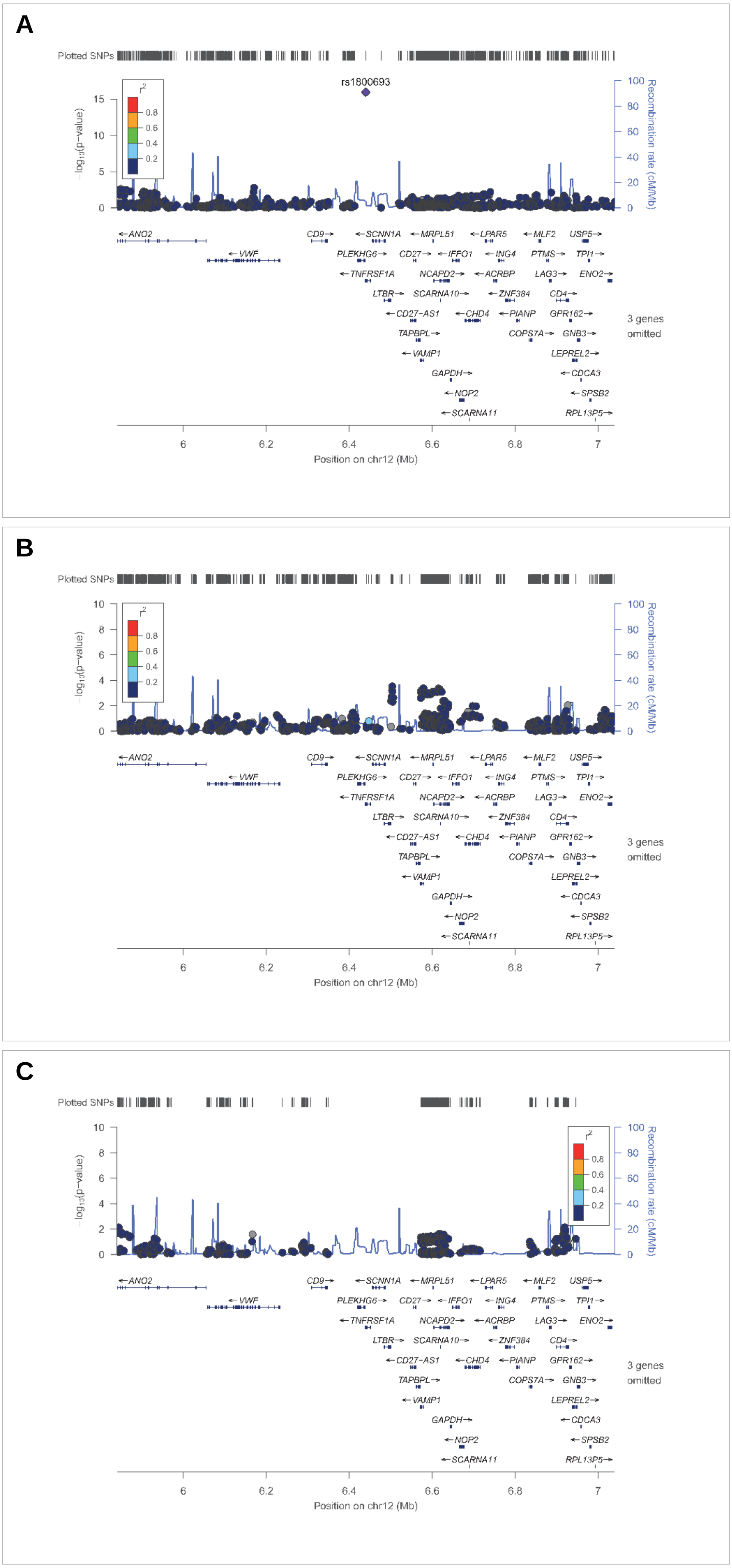


**Fig. S2.39: LocusZoom plots of the known PBC risk locus 12p13.31.** (A) European panels, (B) Asian panels, and (C) all panels combined. Association at this locus reached genome-wide significance in the European panels (rs1800693 at 12:6,440,009; *P* = $1.19\times10^{-16}$); nominal significance in the Asian panels (rs9669611 at 12:6,503,786; *P* = $2.97\times10^{-4}$); and nominal significance in all panels combined (rs11064410 at 12:6,919,569; *P* = $7.18\times10^{-3}$). Permutation testing was not applied owing to futility. Nevertheless, fixed effect meta-analysis of the European and Asian lnORs did not identify significant heterogeneity for the lnORs for rs11064410 ($P_{\text{het meta}}$ = 0.608). (Note that rs1800693 was not tested in the Asian panel, nor rs9669611 in the European panel.) On balance, we consider the signal at 12p13.31 to be well supported in European populations. Conversely, the study was most likely under-powered to reliably confirm or refute association in Asian populations, or trans-ethnic heterogeneity, at this locus.

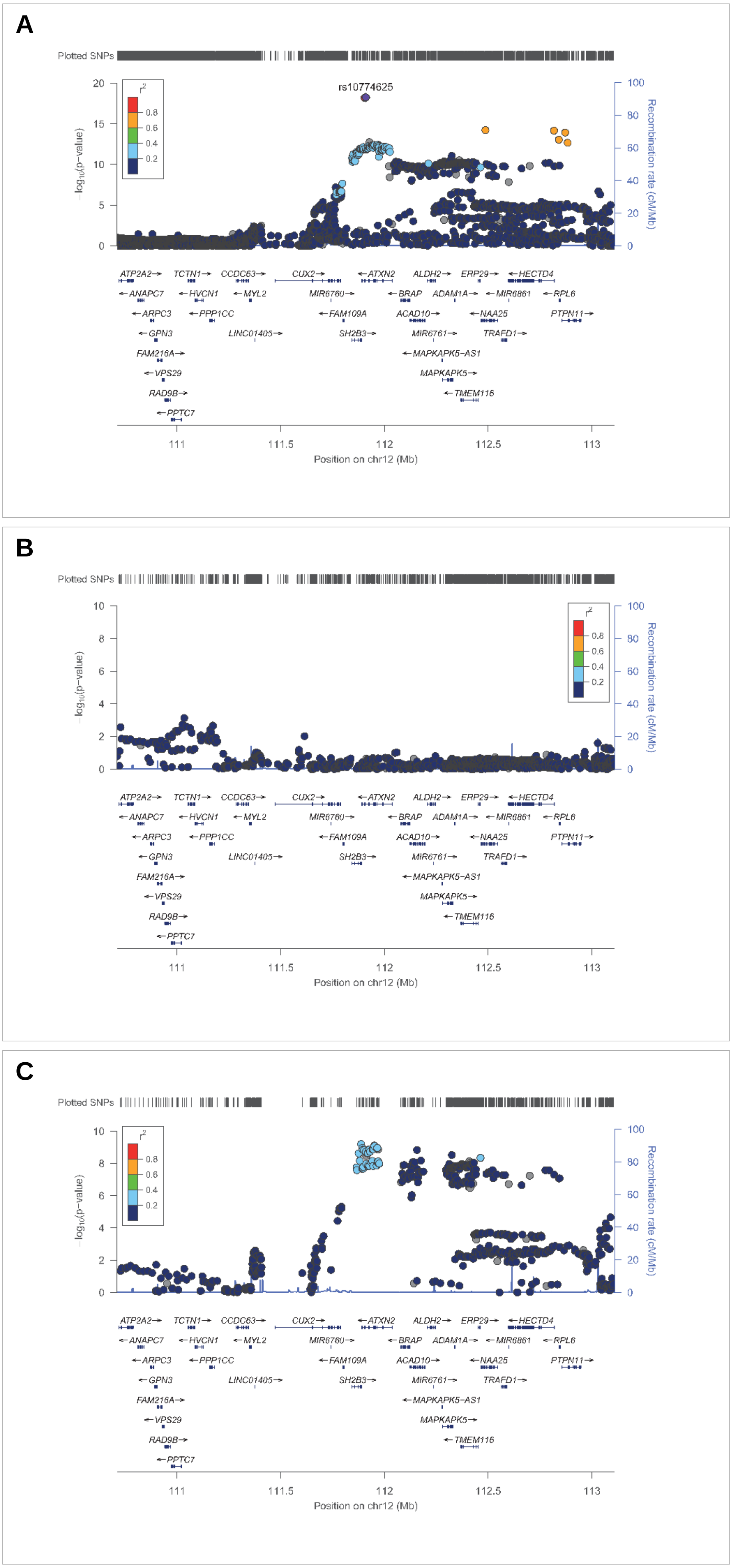


**Fig. S2.40: LocusZoom plots of the known PBC risk locus 12q24.12.** (A) European panels, (B) Asian panels, and (C) all panels combined. Association at this locus reached genome-wide significance in the European panels (rs35350651 at 12:111,907,431; $P$ = 5.50×10$^{-19}$); nominal significance in the Asian panels (rs6489979 at 12:111,614,736; $P$ = 0.0096); and genome-wide significance in all panels combined (rs739496 at 12:111,887,659; $P$ = 6.29×10$^{-10}$). Permutation testing was not applied owing to futility. Nevertheless, fixed effect meta-analysis of the European and Asian lnORs did identify significant heterogeneity in the lnORs for rs739496 ($P_{het\ meta}$ = 9.23×10$^{-4}$). (Note that rs35350651 was not tested in the Asian panel, nor rs6489979 in the European panel.) On balance, we consider the signal at 12q24.12 to be well supported in European populations. Conversely, the study was most likely under-powered to reliably confirm or refute association in Asian populations, or trans-ethnic heterogeneity, at this locus.

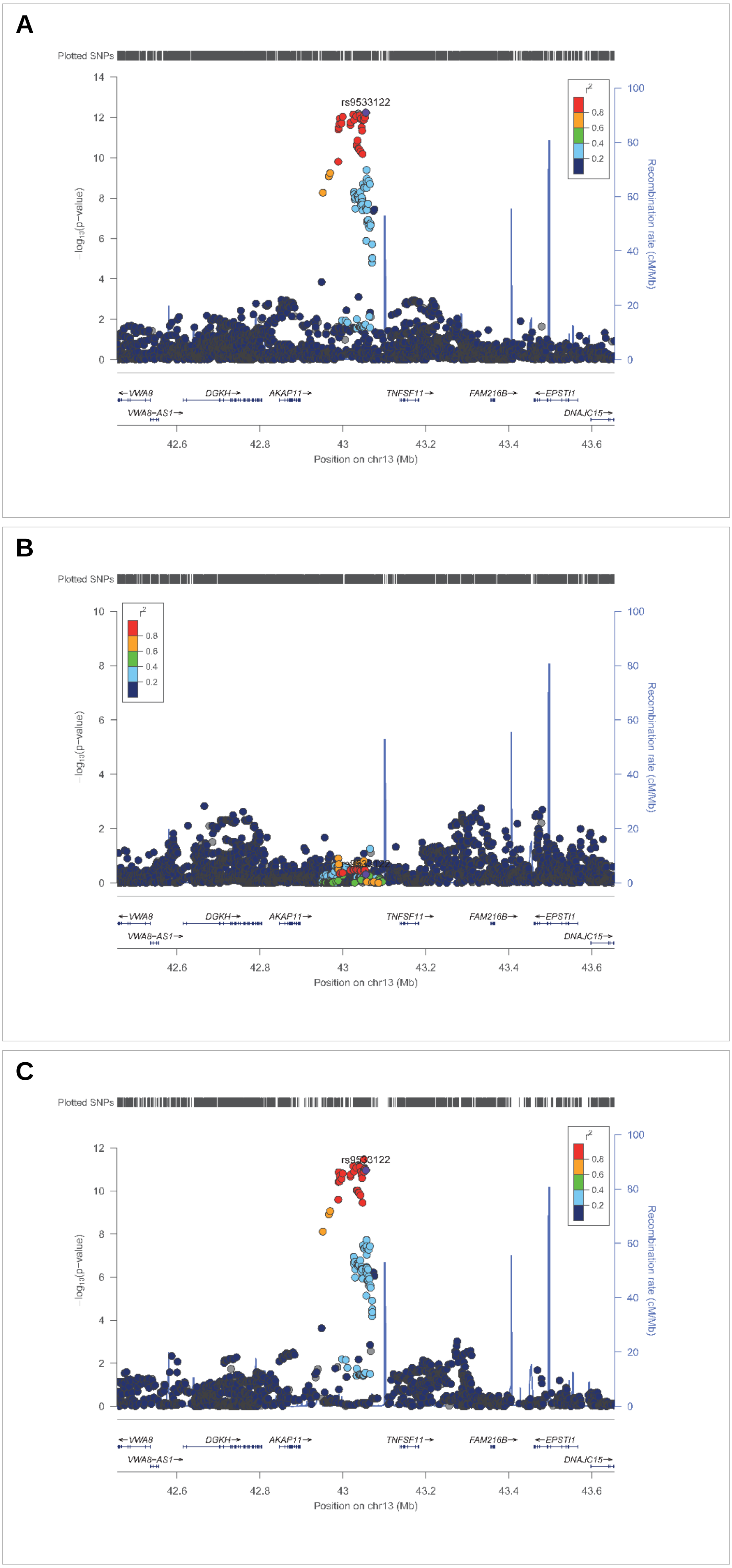


**Fig. S2.41: LocusZoom plots of the known PBC risk locus 13q14.11.** (A) European panels, (B) Asian panels, and (C) all panels combined. Association at this locus reached genome-wide significance in the European panels (rs9533122 at 13:43,055,002; $P$ = 5.83×10$^{-13}$); nominal significance in the Asian panels (13:42644298:GCTT:ACTT at 13:42,644,298; $P$ = 4.35×10$^{-4}$); and genome-wide significance in all panels combined (rs79344245 at 13:43,050,454; $P$ = 3.39×10$^{-12}$). Permutation testing was not applied owing to futility. Fixed effect meta-analysis of the European and Asian lnORs identified significant heterogeneity in the lnORs for rs9533122 ($P_{het\ meta}$ = 0.033) and 13:42644298:GCTT:ACTT ($P_{het\ meta}$ = 7.41×10$^{-4}$), but not for rs79344245 ($P_{het\ meta}$ = 0.084). On balance, we consider the signal at this locus to be well supported in European populations. Conversely, the study was most likely under-powered to reliably confirm or refute association in Asian populations, or trans-ethnic heterogeneity, at this locus.

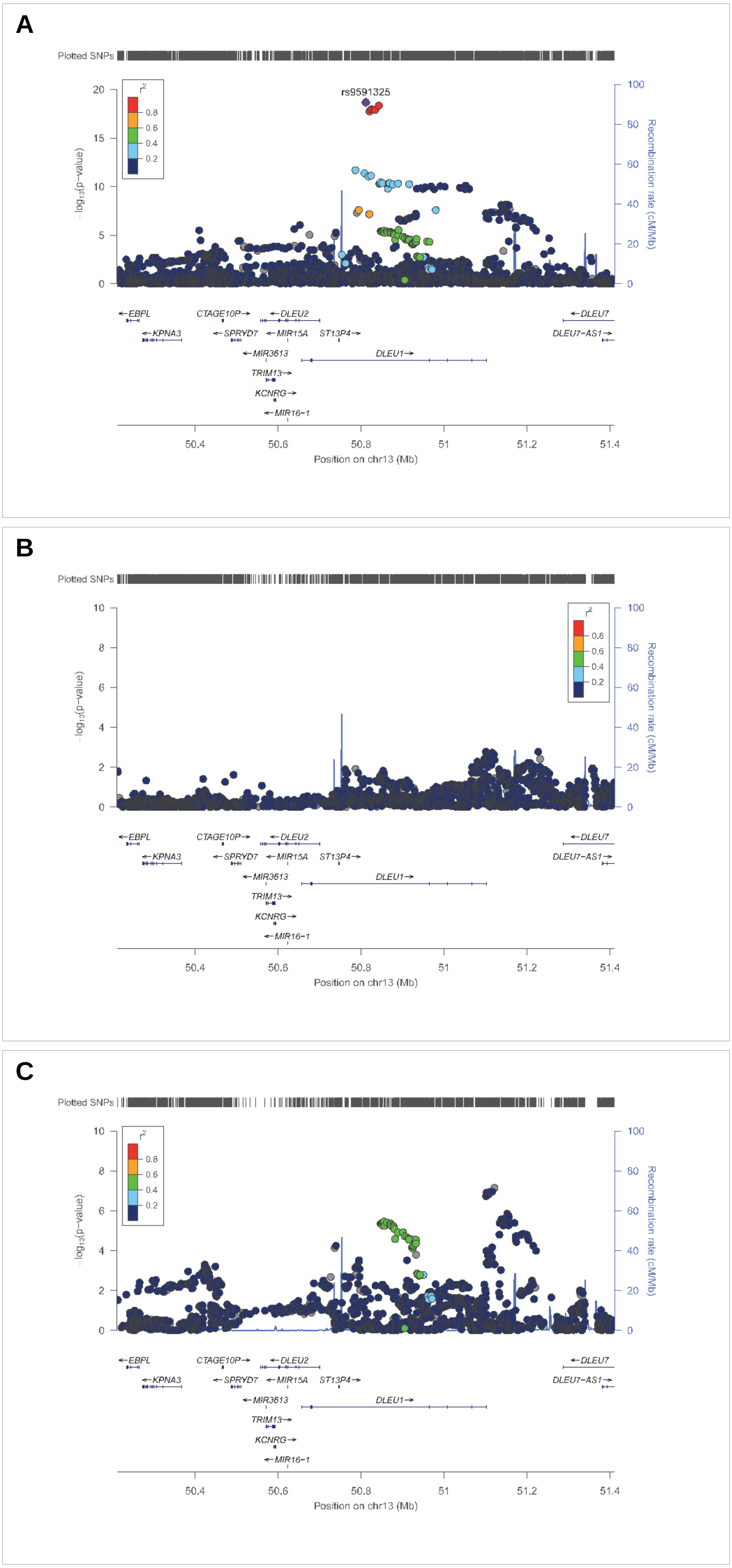


**Fig. S2.42: LocusZoom plots of the known PBC risk locus 13q14.2.** (A) European panels, (B) Asian panels, and (C) all panels combined. Association at this locus reached genome-wide significance in the European panels (rs9591325 at 13:50,811,220; $P = 2.14\times10^{-19}$); nominal significance in the Asian panels (rs279075 at 13:51,100,714; $P = 0.0017$); and suggestive significance in all panels combined (rs199565066 at 13:51,121,377; $P = 7.09\times10^{-8}$). Permutation testing was not applied owing to futility. Fixed effect meta-analysis of the European and Asian lnORs identified significant heterogeneity in the lnORs for rs279075 ($P_{het\ meta} = 0.012$) and rs199565066 ($P_{het\ meta} = 0.037$). (Note that rs9591325 was not tested in the Asian panel.) On balance, we consider the signal at 13q14.2 to be well supported in European populations. Conversely, the study was most likely under-powered to reliably confirm or refute association in Asian populations, or trans-ethnic heterogeneity, at this locus.

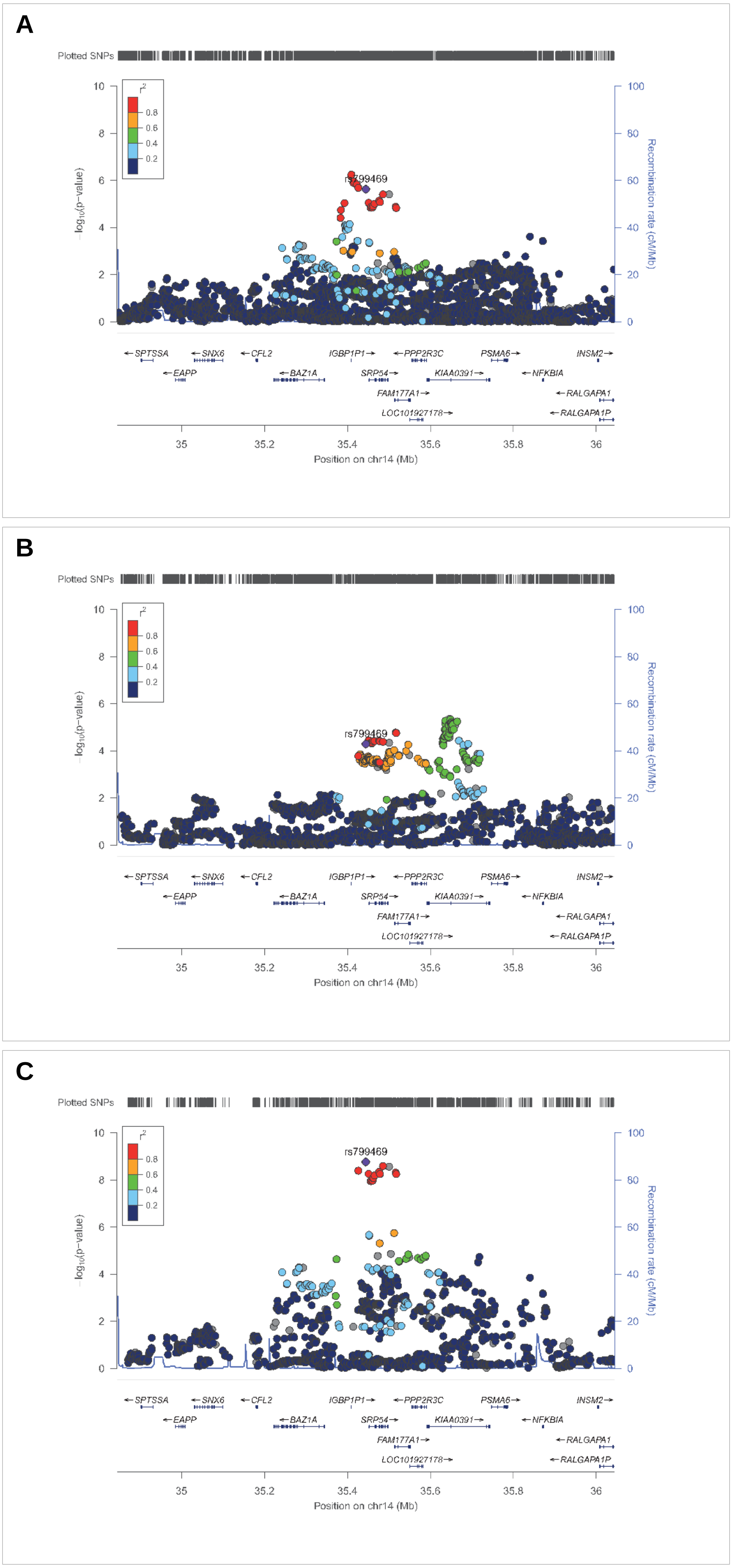


**Fig. S2.43: LocusZoom plots of the newly identified PBC risk locus 14q13.2.** (A) European panels, (B) Asian panels, and (C) all panels combined. Association at this locus reached suggestive significance in the European panels (rs712315 at 14:35,409,701; $P$ = 5.70×10$^{-7}$); suggestive significance in the Asian panels (rs199892962 at 14:35,646,404; $P$ = 4.36×10$^{-6}$); and genome-wide significance in all panels combined (rs799469 at 14:35,444,425; $P$ = 1.73×10$^{-9}$). Permutation testing suggested that the European signal at rs712315 was corroborated by an Asian signal at rs199892962 ($P_{permutation}$ = 0.002), reaching the Bonferroni-corrected threshold of $P$ <0.00217 (correcting for 23 newly identified loci) although not the Bonferroni-corrected threshold of $P$ < 0.000893 (correcting for 56 genome-wide significant loci). Fixed effect meta-analysis of the European and Asian lnORs did not identify significant heterogeneity in the lnORs for rs799469 ($P_{het\ meta}$ = 0.329). (Note that rs712315 was not tested in the Asian panels, nor rs199892962 in the European panels.) On balance, we consider the signal at 14q13.2 to be well supported across European populations, with some evidence of support across Asian populations.

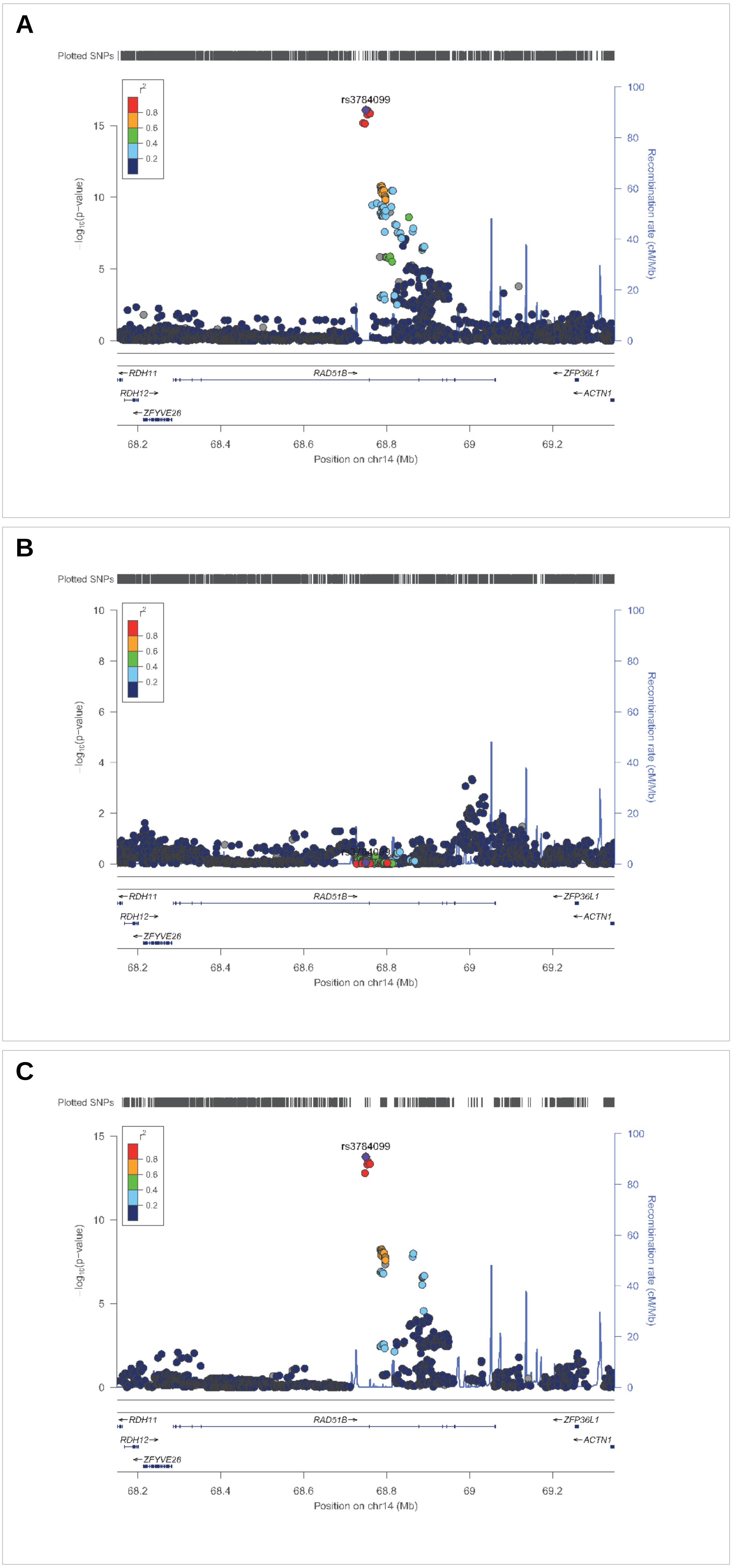


**Fig. S2.44: LocusZoom plots of the known PBC risk locus 14q24.1.** (A) European panels, (B) Asian panels, and (C) all panels combined. Association at this locus reached genome-wide significance in the European panels (rs3784099 at 14:68,749,927; $P = 8.31\times10^{-17}$); nominal significance in the Asian panels (rs12882030 at 14:69,005,283; $P = 4.42\times10^{-4}$); and genome-wide significance in the combined panels (rs3784099; $P = 1.69\times10^{-14}$). Permutation testing was not applied owing to futility. Fixed effect meta-analysis of European and Asian panels identified significant heterogeneity in the lnORs for rs3784099 ($P_{het\ meta} = 0.004$). (Note that rs12882030 was not tested in the European panels.) On balance, we consider the signal at 14q24.1 to be well supported in European populations. Conversely, the study was most likely under-powered to reliably confirm or refute association in Asian populations, or trans-ethnic heterogeneity, at this locus.

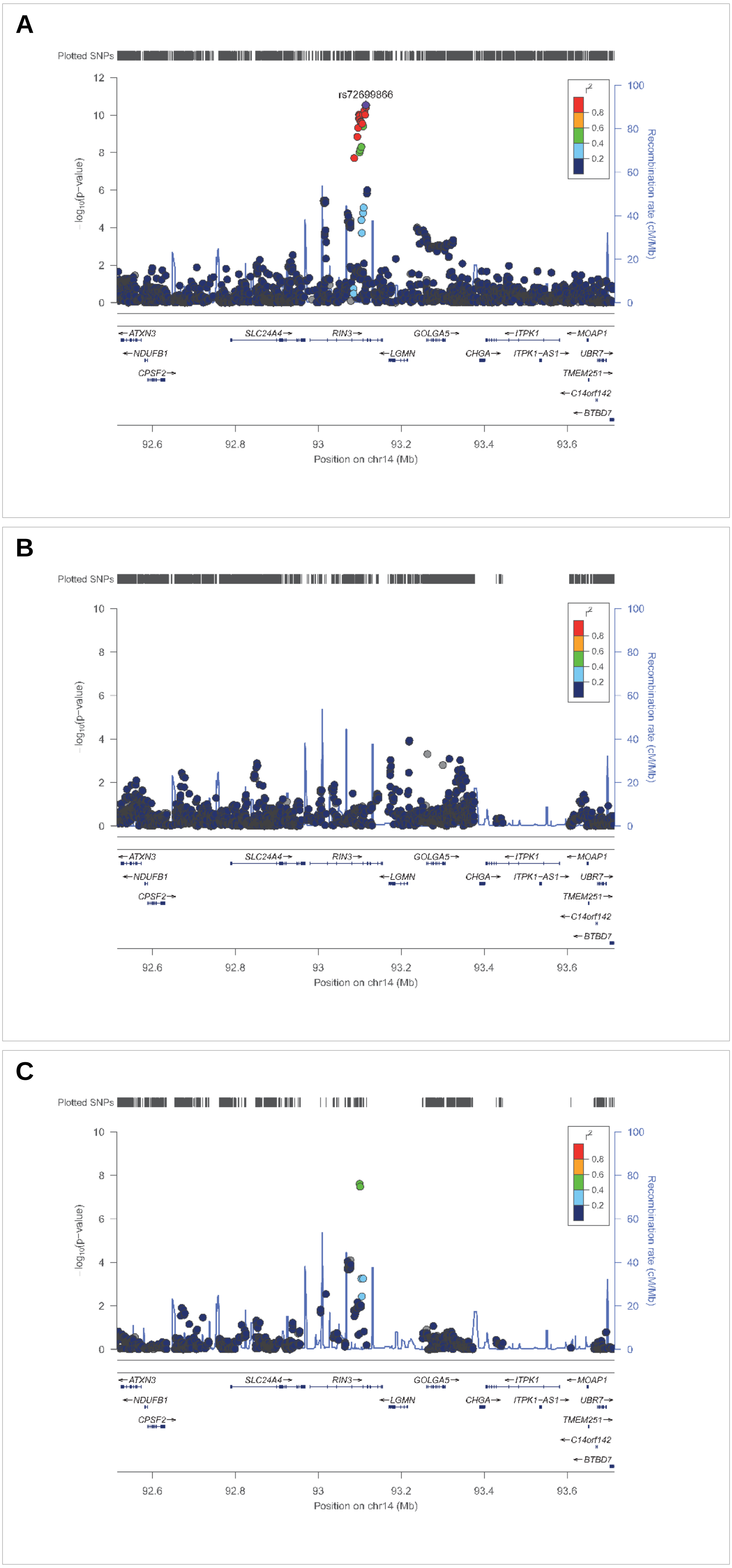


**Fig. S2.45: LocusZoom plots of the newly identified PBC risk locus 14q32.12.** (A) European panels, (B) Asian panels, and (C) all panels combined. Association at this locus reached genome-wide significance in the European panels (rs72699866 at 14:93,114,787; $P = 2.89\times10^{-11}$); suggestive significance in the Asian panels (rs76914265 at 14:93,219,854; $P = 1.16\times10^{-4}$); and genome-wide significance in all panels combined (rs4904964 at 14:93,099,867; $P = 2.45\times10^{-8}$). Permutation testing showed that the European signal at rs72699866 was weakly corroborated by an Asian signal at rs76914265 ($P_{permutation} = 0.0143$), albeit not at the Bonferroni-corrected threshold of $P < 0.00217$ (correcting for 23 newly identified loci) or $P < 0.000893$ (correcting for 56 genome-wide significant loci). Fixed effect meta-analysis of the European and Asian lnORs did not identify significant heterogeneity in the lnORs for rs4904964 ($P_{het\ meta} = 0.111$). (Note that rs72699866 was not tested in the Asian panels, nor rs76914265 in the European panels.) On balance, we consider the signal at 14q32.12 to be supported across European populations, with some weak level of support seen across Asian populations.

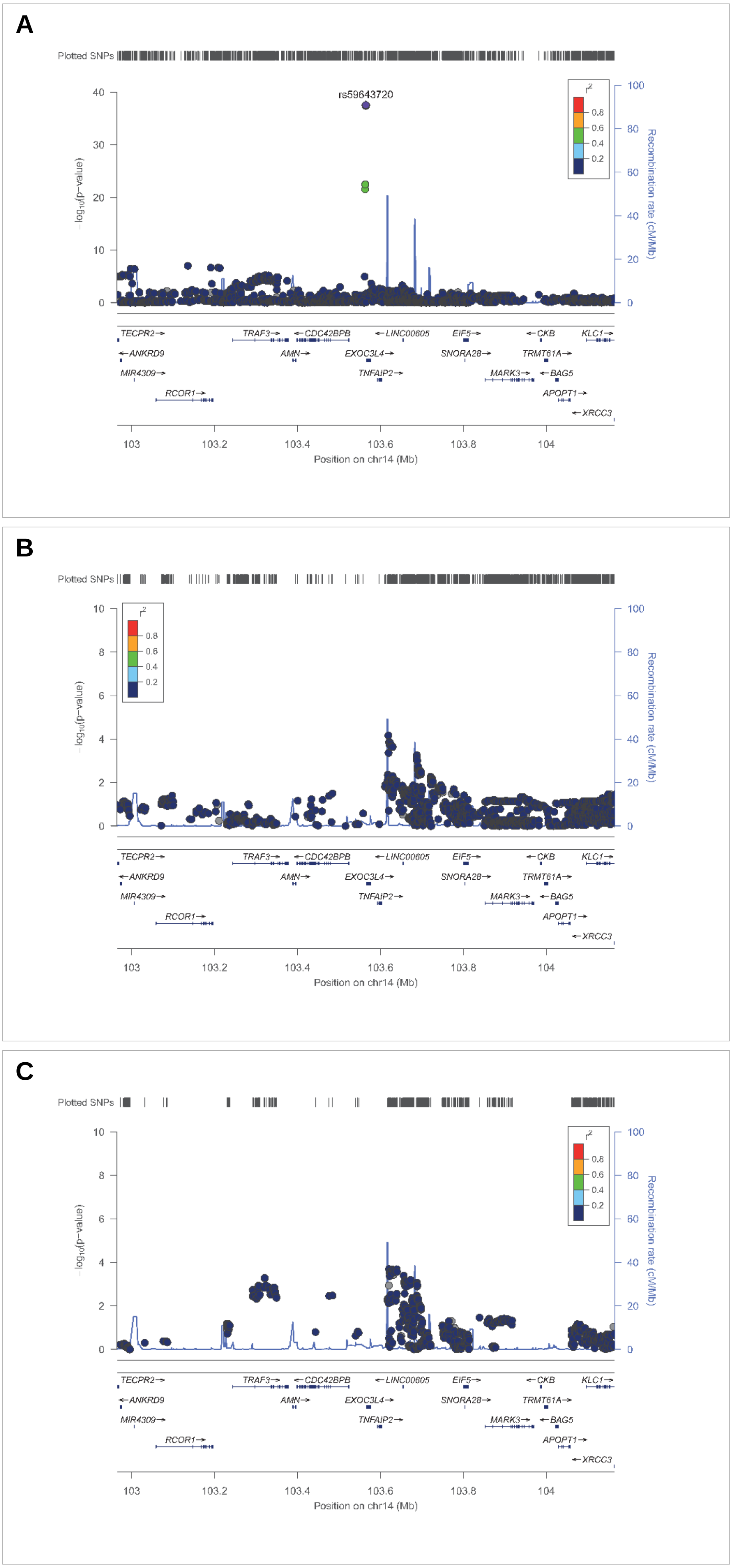


**Fig. S2.46: LocusZoom plots of the known PBC risk locus 14q32.32.** (A) European panels, (B) Asian panels, and (C) all panels combined. Association at this locus reached genome-wide significance in the European panels (rs59643720 at 14:103,564,807; P = $2.73\times10^{-38}$); suggestive significance in the Asian panels (rs2403135 at 14:103,619,239; $P = 6.75\times10^{-5}$); and nominal significance in all panels combined (rs8012262 at 14:103,620,614; $P = 2.01\times10^{-4}$). Permutation testing suggested that the European signal at rs59643720 was corroborated by an Asian signal at rs2403135 ($P_{permutation} = 0.0023$), albeit not at the Bonferroni-corrected threshold of $P < 0.000893$ (correcting for 56 genome-wide significant loci). Moreover, fixed effect meta-analysis of the European and Asian lnORs did not identify significant heterogeneity in the lnORs for rs8012262 ($P_{het\ meta} = 0.577$). (Note that rs59643720 was not tested in the Asian panel, nor rs2403135 in the European panel.) Thus, we consider this signal to be supported across European populations, with some evidence of support across Asian populations.

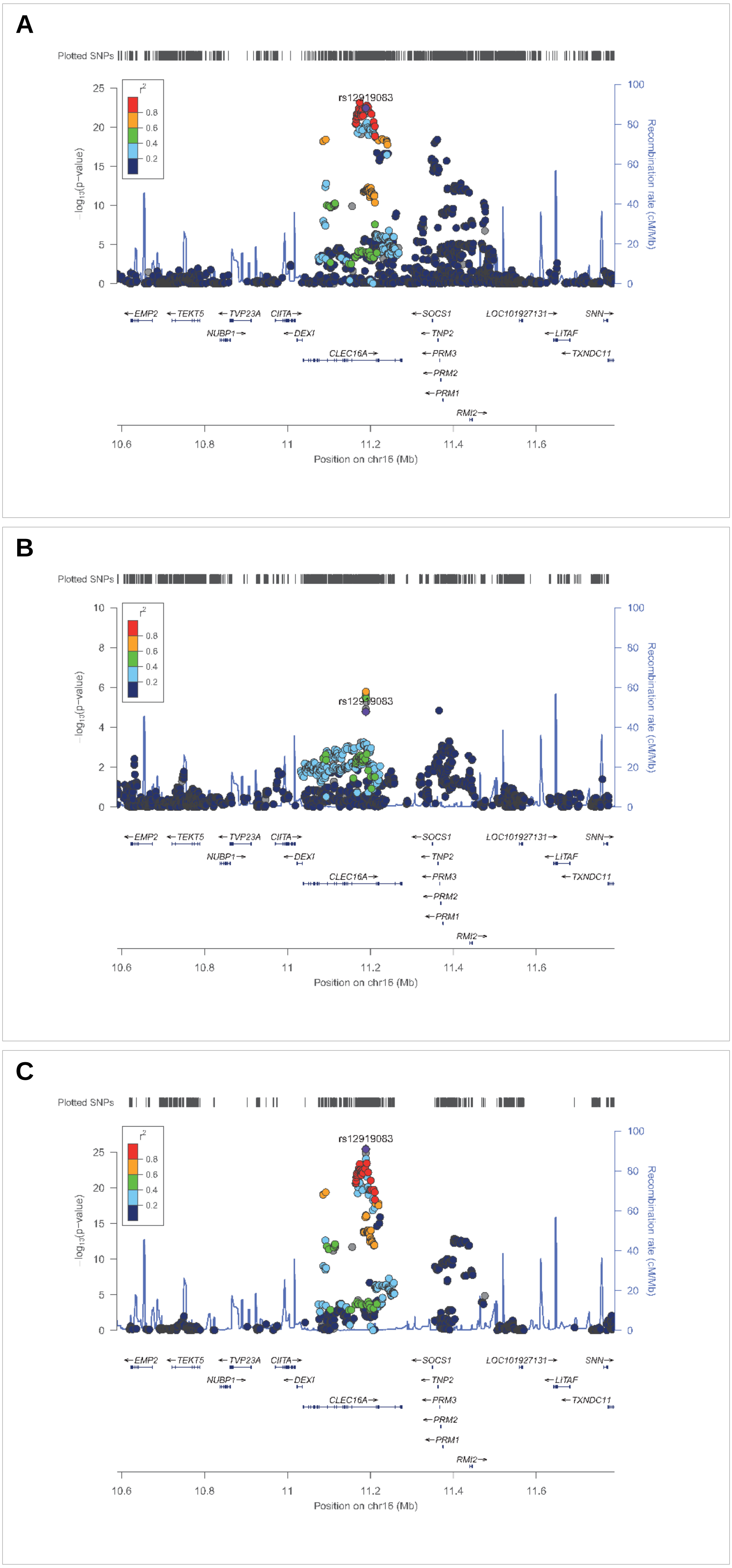


**Fig. S2.47: LocusZoom plots of the known PBC risk locus 16p13.13.** (A) European panels, (B) Asian panels, and (C) all panels combined. Association at this locus reached genome-wide significance in the European panels (rs9652601 at 16:11,174,365; $P = 6.69\times10^{-24}$); suggestive significance in the Asian panels (rs7205916 at 16:11,189,256; $P = 1.63\times10^{-6}$); and genome-wide significance in all panels combined (rs12919083 at 16:11,188,930; $P = 3.63\times10^{-26}$). Permutation testing at this locus confirmed that the European signal at rs9652601 was corroborated by an Asian signal at rs7205916 ($P_{permutation}$ <0.0001). Furthermore, fixed effect meta-analysis of the European and Asian lnORs did not identify significant heterogeneity in the lnORs for rs9652601 ($P_{het\ meta} = 0.059$), rs7205916 ($P_{het\ meta} = 0.152$), or rs12919083 ($P_{het\ meta} = 0.354$). Thus, we consider this signal to be well supported across both European and Asian populations.

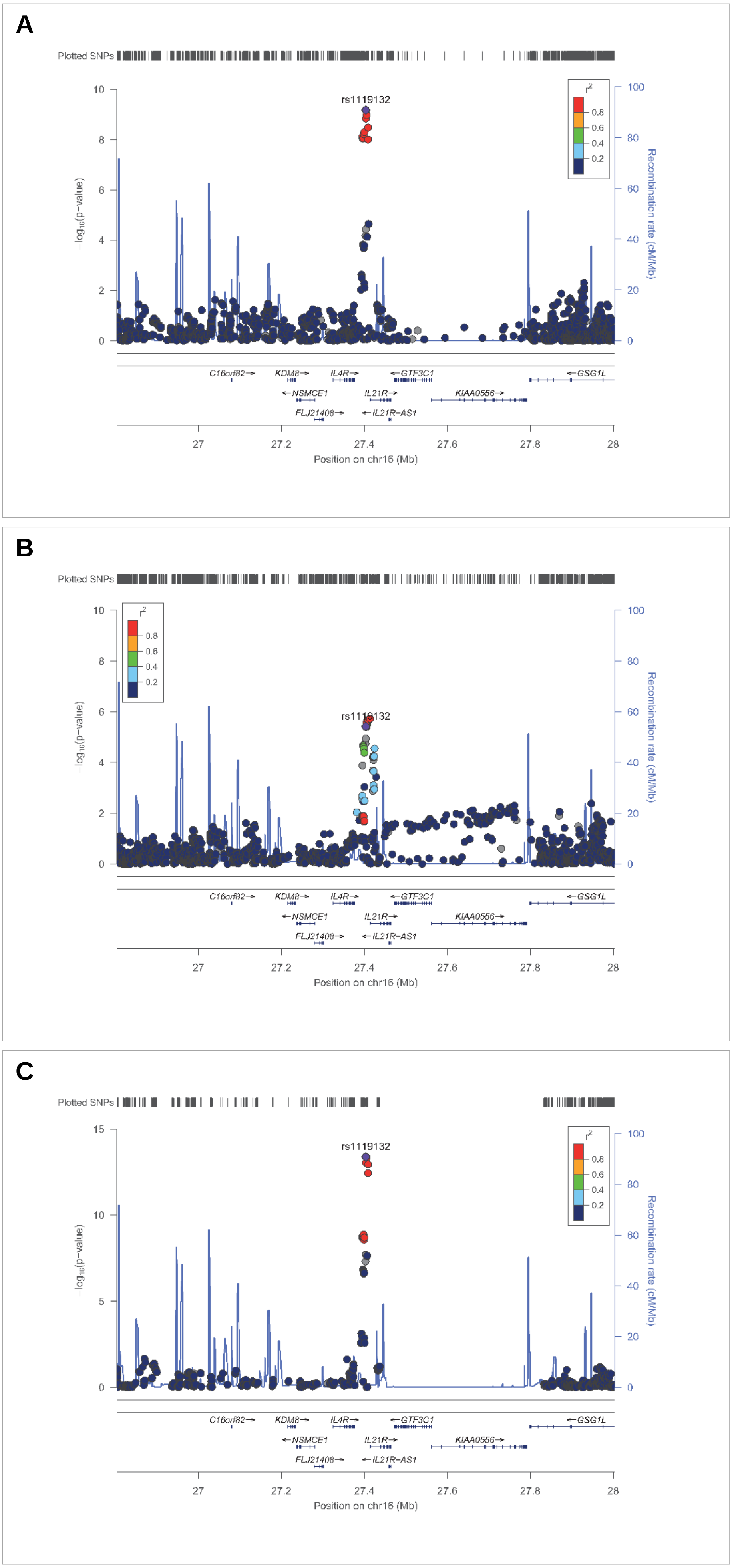


**Fig. S2.48: LocusZoom plots of the known PBC risk locus 16p12.1.** (A) European panels, (B) Asian panels, and (C) all panels combined. Association at this locus reached genome-wide significance in the European panels (rs1119132 at 16:27,403,469; $P = 6.58\times10^{-10}$); suggestive significance in the Asian panels (rs2189521 at 16:27,413,566; $P = 1.87\times10^{-6}$); and genome-wide significance in all panels combined (rs1119132; $P = 4.09\times10^{-14}$). Permutation testing confirmed that the European signal at rs1119132 was corroborated by an Asian signal at rs2189521 ($P_{permutation}$ <0.0001). Furthermore, fixed effect meta-analysis of the European and Asian lnORs did not identify significant heterogeneity in the lnORs for rs1119132 ($P_{het\ meta} = 0.754$). (Note that rs2189521 was not tested in the European panels.) Thus, we consider the signal at 16p12.1 to be well supported across both European and Asian populations.

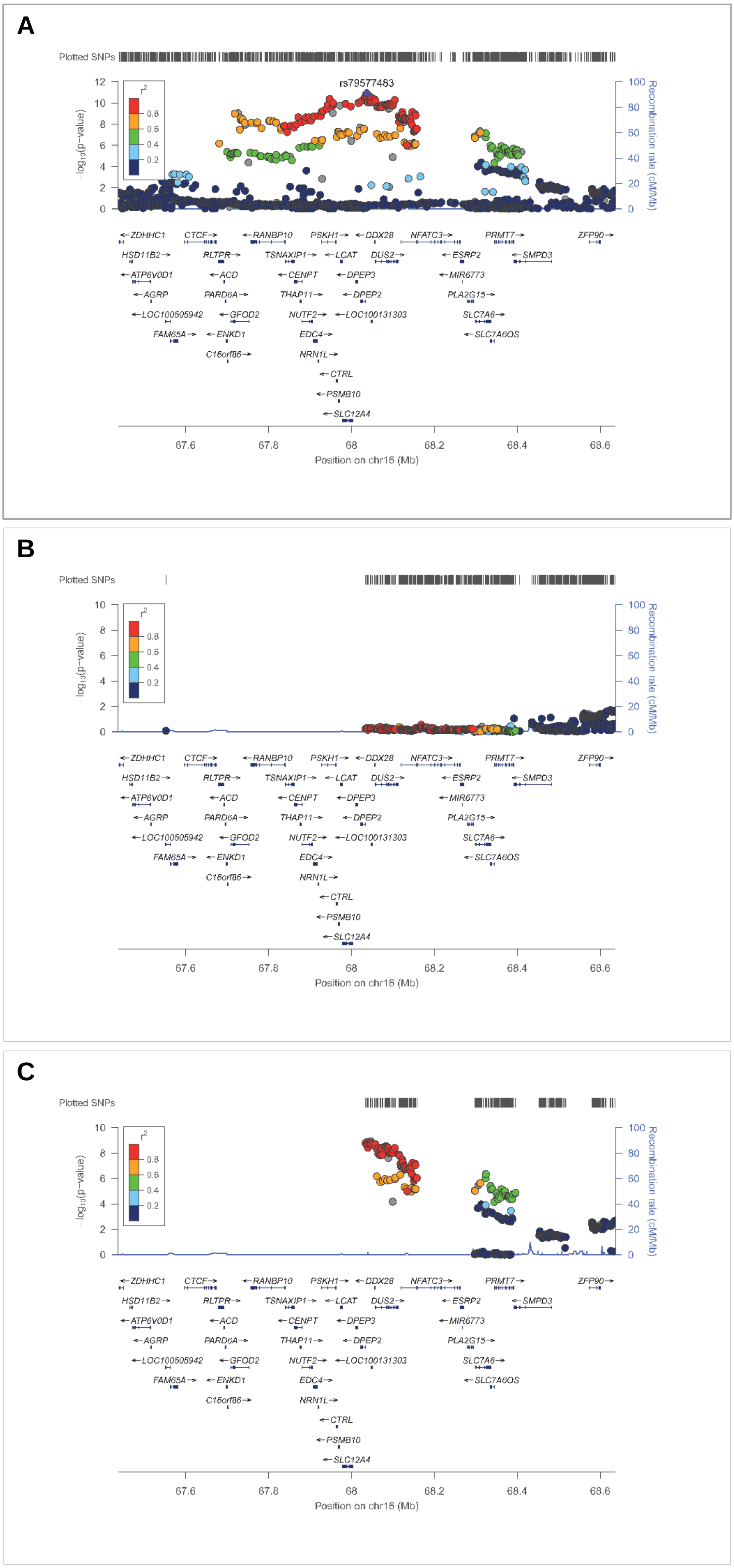


**Fig. S2.49: LocusZoom plots of the newly identified PBC risk locus 16q22.1.** (A) European panels, (B) Asian panels, and (C) all panels combined. Association at this locus reached genome-wide significance in the European panels (rs79577483 at 16:68,036,939; $P$ = $1.23\times10^{-11}$); nominal significance in the Asian panels (rs698729 at 16:68,624,205; $P$ = 0.019); and genome-wide significance in all panels combined (rs111644390 at 16:68,046,323; $P$ = $1.18\times10^{-9}$). Permutation testing was not applied owing to futility. Fixed effect meta-analysis of the European and Asian lnORs identified significant heterogeneity in the lnORs for rs111644390 ($P_{het\ meta}$ = 0.011), but not for rs698729 ($P_{het\ meta}$ = 0.261). (Note that rs79577483 was not tested in the Asian panels.) We consider the signal at 16q22.1 to be well supported in European populations. Conversely, the study was most likely under-powered to reliably confirm or refute association in Asian populations, or trans-ethnic heterogeneity, at this locus.

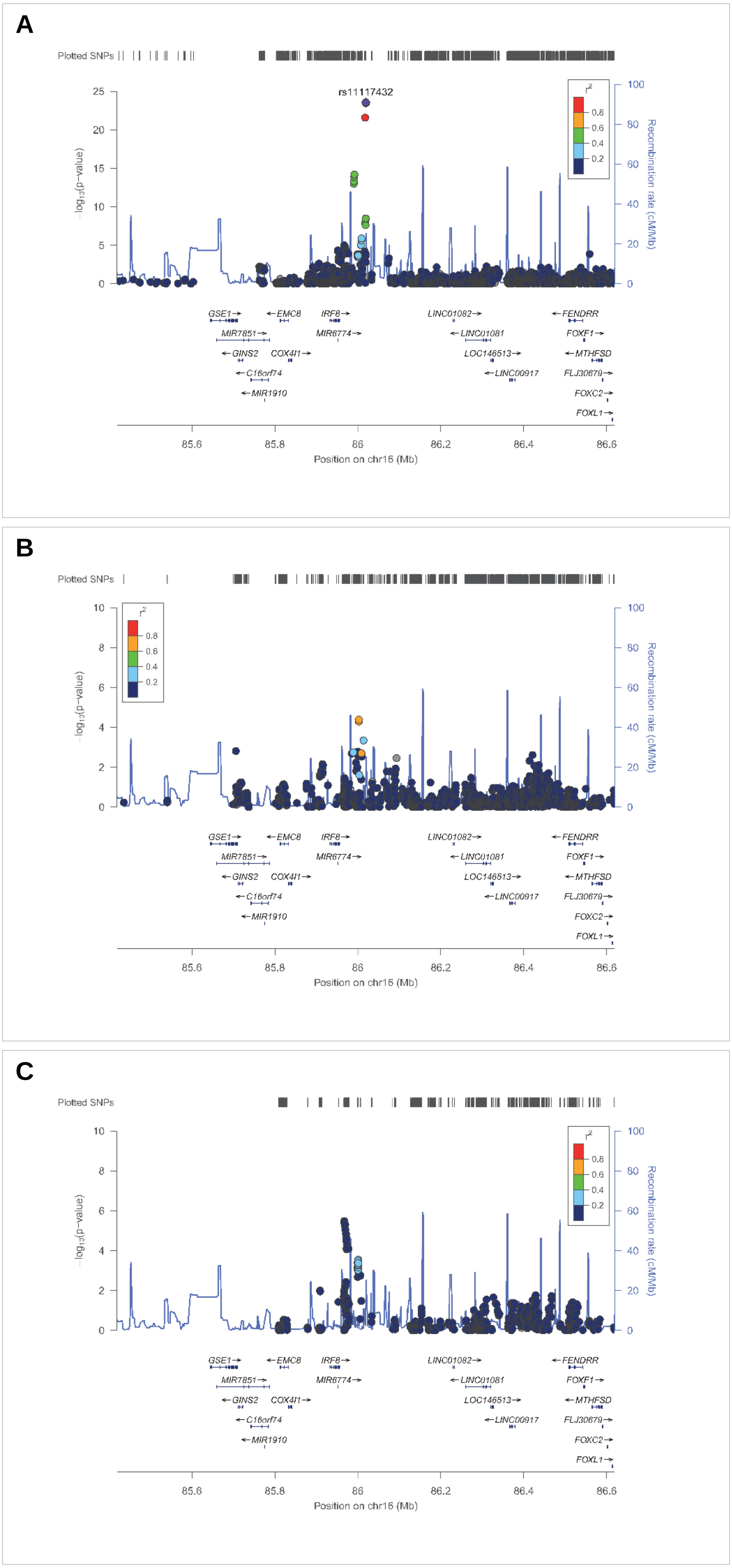


**Fig. S2.50: LocusZoom plots of the known PBC risk locus 16q24.1.** (A) European panels, (B) Asian panels, and (C) all panels combined. Association at this locus reached genome-wide significance in the European panels (rs11117432 at 16:86,019,271; $P = 2.82\times10^{-24}$); suggestive significance in the Asian panels (rs9938016 at 16:86,002,524; $P = 4.11\times10^{-5}$); and suggestive significance in all panels combined (rs12444221 at 16:85,967,285; $P = 3.26\times10^{-6}$). Permutation testing at this locus suggested that the European signal at rs11117432 was corroborated by an Asian signal at rs9938016 ($P_{permutation} = 0.0011$), albeit not at the Bonferroni-corrected threshold of $P < 0.000893$ (correcting for 56 genome-wide significant loci). Moreover, fixed effect meta-analysis of the European and Asian lnORs did not identify significant heterogeneity in the lnORs for rs12444221 ($P_{het\ meta} = 0.871$). (Note that rs11117432 was not tested in the Asian panels, nor rs9938016 in the European panels.) Thus, we consider the signal at 16q24.1 to be well supported across European populations with some evidence of support across Asian populations.

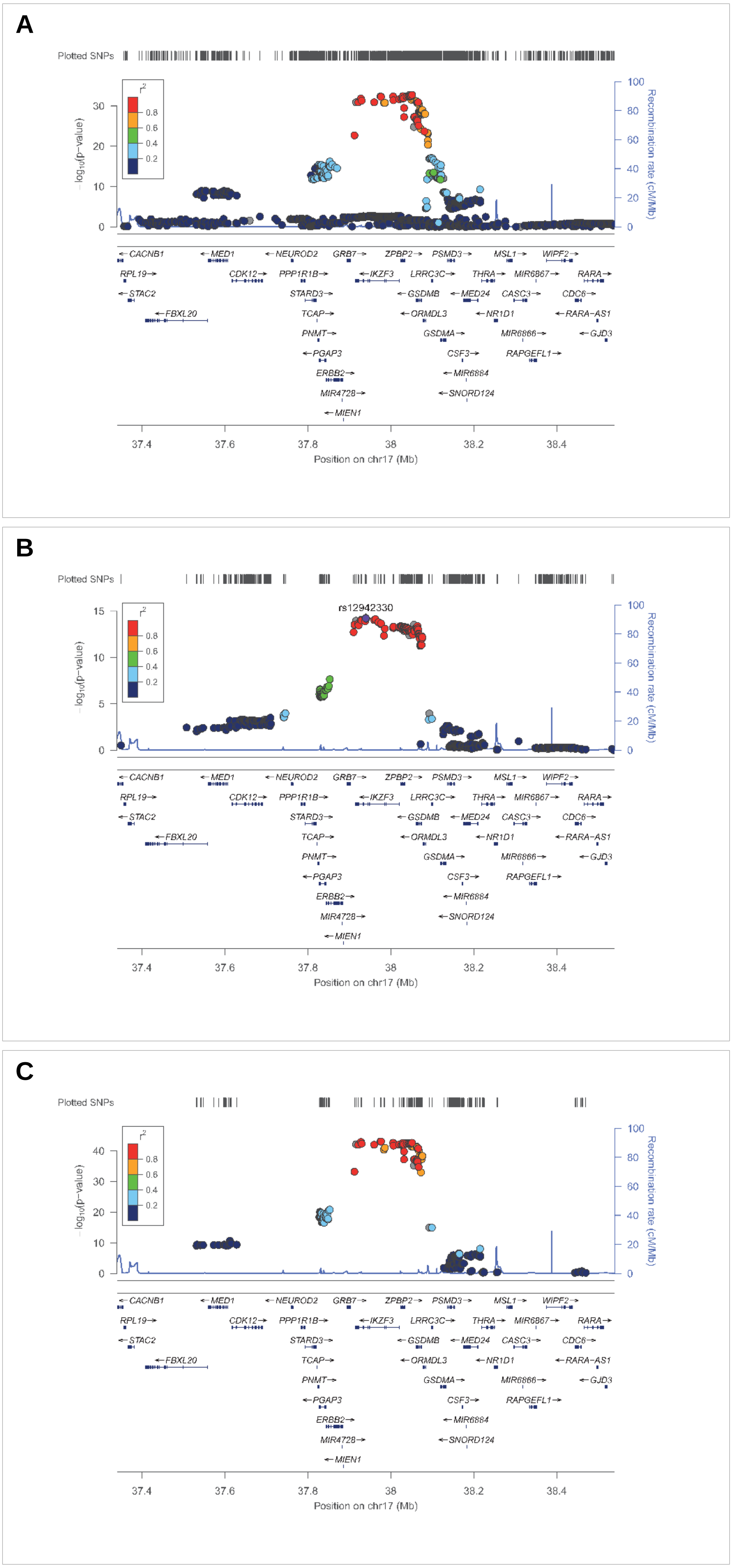


**Fig. S2.51: LocusZoom plots of the known PBC risk locus 17q12.** (A) European panels, (B) Asian panels, and (C) all panels combined. Association at this locus reached genome-wide significance in the European panels (rs33938760 at 17:38,044,893; $P$ = $1.75 \times 10^{-33}$), Asian panels (rs12942330 at 17:37,939,839; $P$ = $5.77 \times 10^{-15}$), and all panels combined (rs71152606 at 17:37,975,214; $P$ = $8.36 \times 10^{-44}$). Permutation testing confirmed that the European signal at rs33938760 was corroborated by an Asian signal at rs12942330 ($P_{permutation}$ <0.0001). Furthermore, fixed effect meta-analysis of the European and Asian lnORs did not identify significant heterogeneity in the lnORs for rs33938760 ($P_{het\ meta}$ = 0.309) or rs71152606 ($P_{het\ meta}$ = 0.254). (Note that rs12942330 was not tested in the European panels.) Thus, we consider the signal at this locus to be well supported across both European and Asian populations.

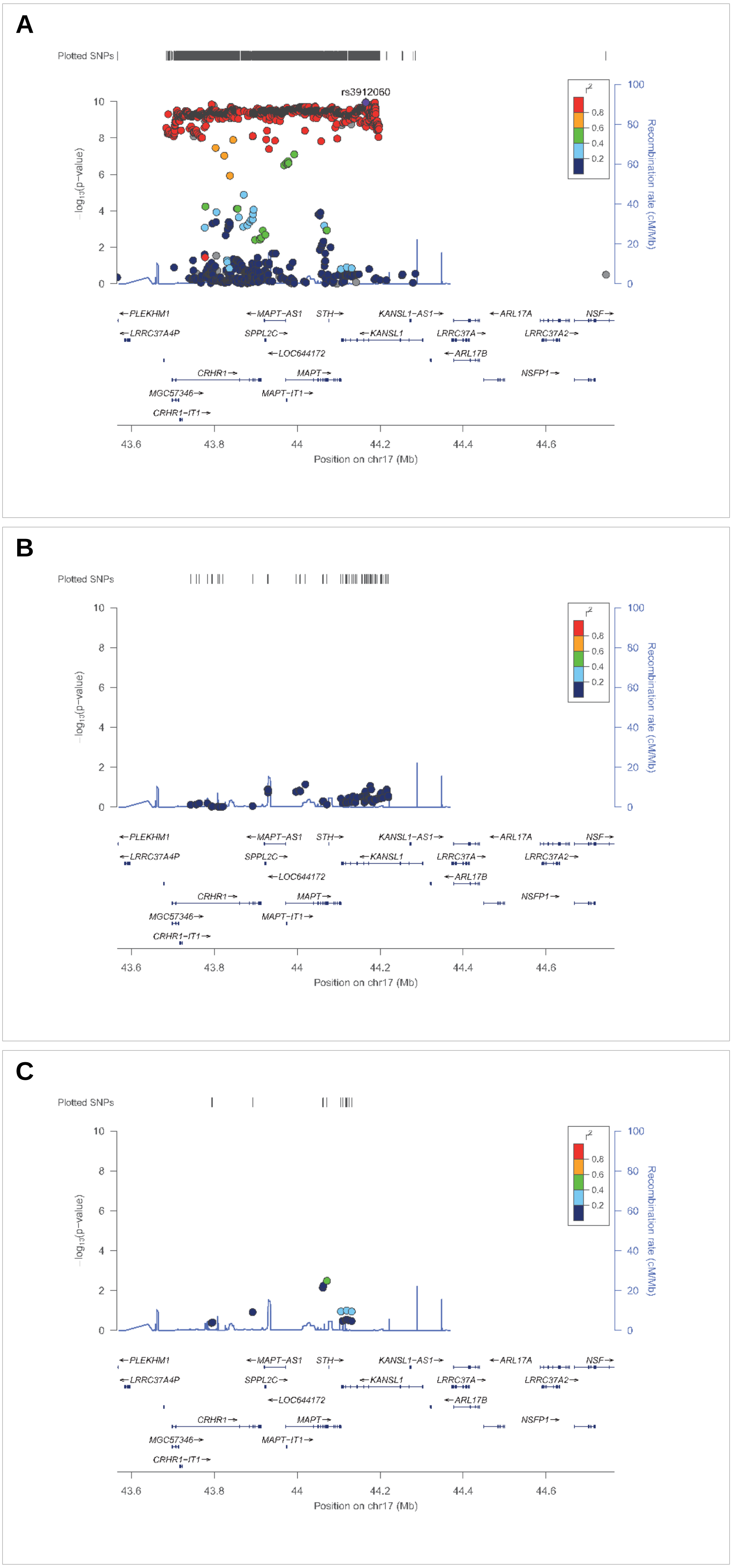


**Fig. S2.52: LocusZoom plots of the known PBC risk locus 17q21.31.** (A) European panels, (B) Asian panels, and (C) all panels combined. Association at this locus reached genome-wide significance in the European panels (17:44149348:A:C at 17:44,149,348; *P* = $8.16\times10^{-11}$); not even nominal significance in the Asian panels (rs242557 at 17:44,019,712; *P* = 0.0723); and only nominal significance in all panels combined (rs2435200 at 17:44,071,851; *P* = $3.25\times10^{-3}$). Permutation testing was not applied owing to futility. Nevertheless, fixed effect meta-analysis of the European and Asian lnORs did not identify significant heterogeneity in the lnORs for rs2435200 ($P_{het\ meta}$ = 0.192). (Note that 17:44149348:A:C was not tested Asian panels, nor rs242557 in the Asian panels.) We consider the signal at 17q21.31 to be well supported in European populations. Conversely, the study was most likely under-powered to reliably confirm or refute association in Asian populations, or trans-ethnic heterogeneity, at this locus.

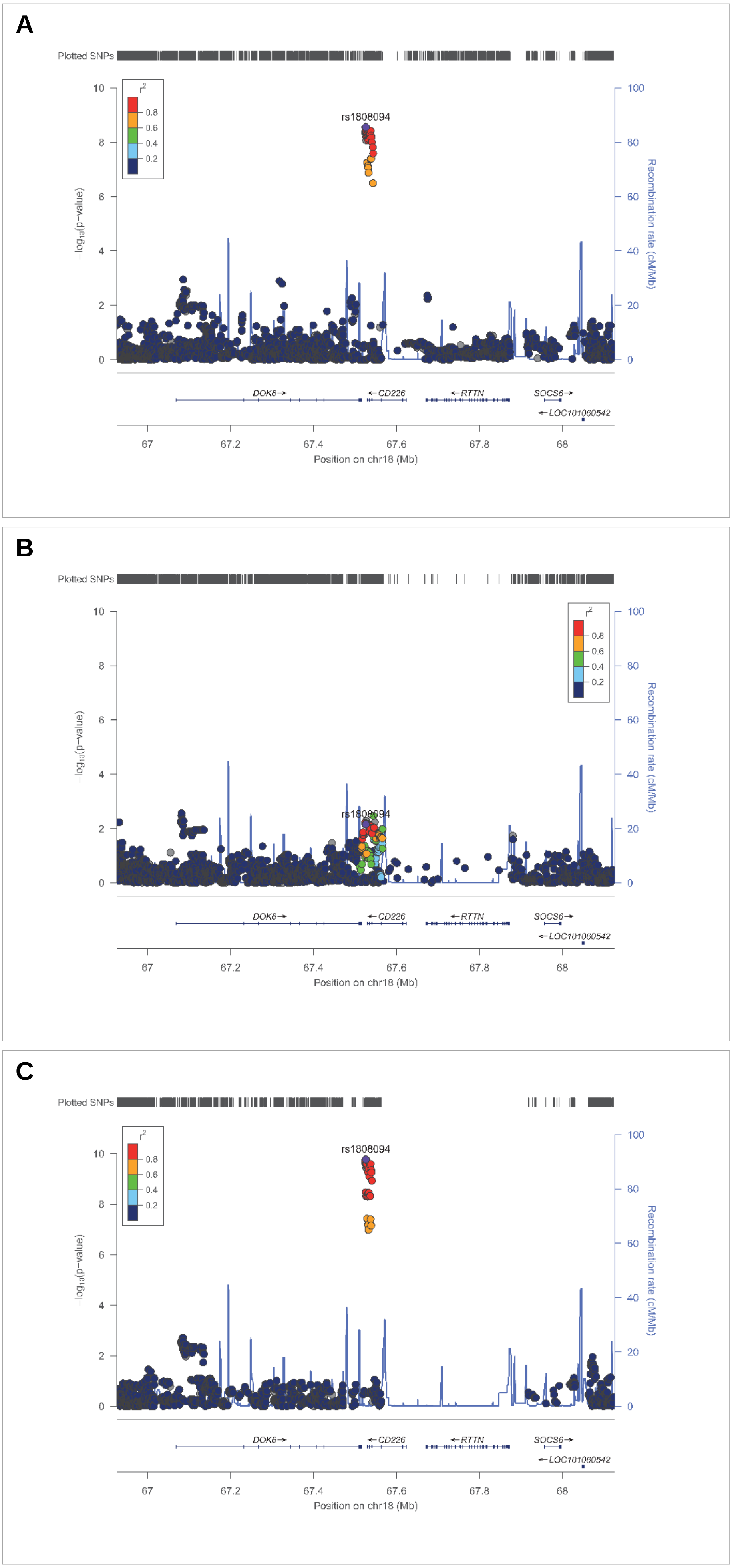


**Fig. S2.53: LocusZoom plots of the newly identified PBC risk locus 18q22.2.** (A) European panels, (B) Asian panels, and (C) all panels combined. Association at this locus reached genome-wide significance in the European panels (rs1808094 at 18:67,526,026; $P$ = 2.79×10$^{-9}$); nominal significance in the Asian panels (rs76486918 at 18:67081620; $P$ = 0.0027); and genome-wide significance in all panels combined (rs1808094; $P$ = 1.66×10$^{-10}$). Permutation testing was not applied owing to futility. Fixed effect meta-analysis of the European and Asian lnORs identified significant heterogeneity in the lnORs for rs76486918 ($P_{het\ meta}$ = 0.010), but not for rs1808094 ($P_{het\ meta}$ = 0.635). Thus, we consider the signal at 18q22.2 to be well supported in European populations. Conversely, the study was most likely under-powered to reliably confirm or refute association in Asian populations, or trans-ethnic heterogeneity, at this locus.

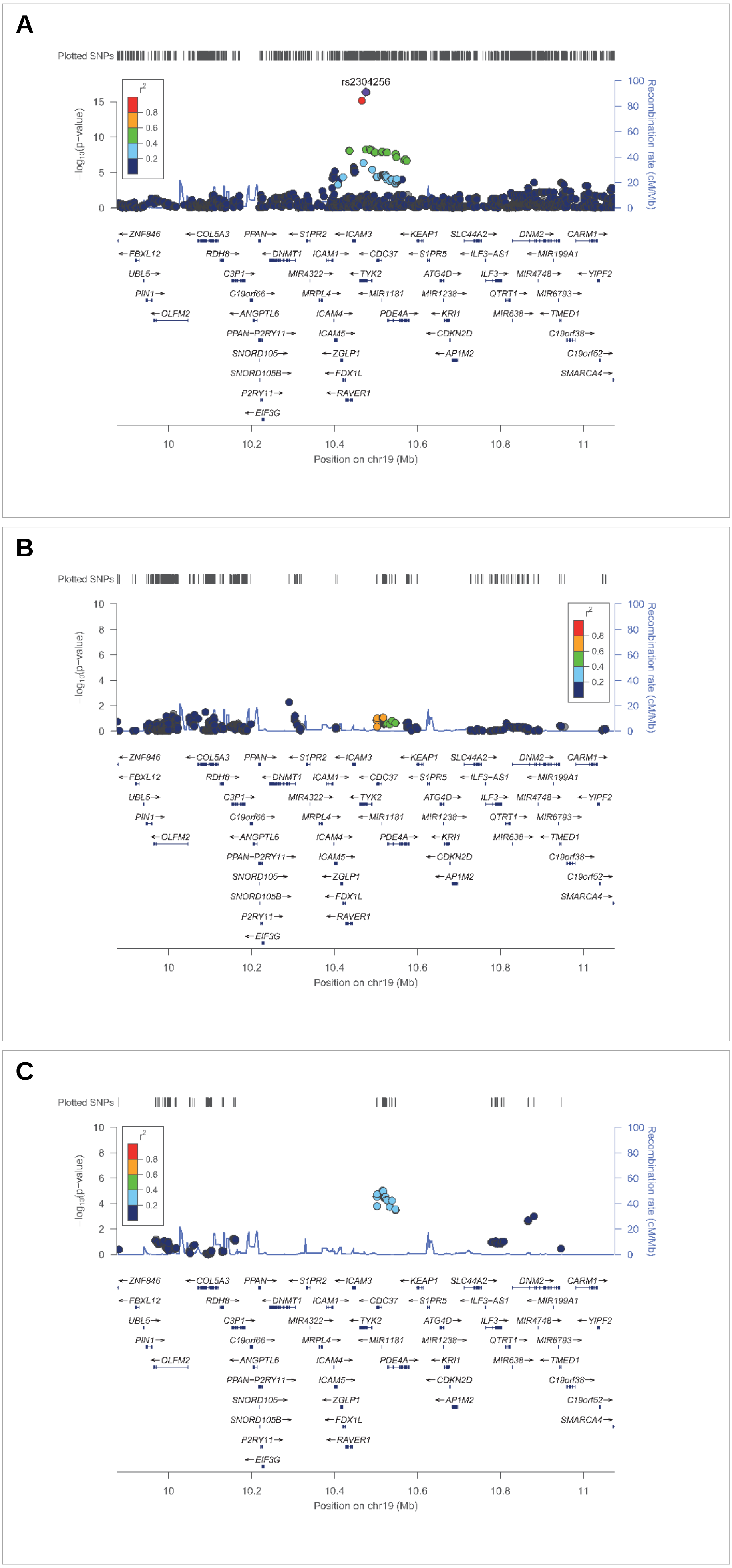


**Fig. S2.54: LocusZoom plots of the known PBC risk locus 19p13.2.** (A) European panels, (B) Asian panels, and (C) all panels combined. Association at this locus reached genome-wide significance in the European panels (rs2304256 at 19:10,475,652; $P$ = 4.43×10$^{-17}$); nominal significance in the Asian panels (rs16999593 at 19:10,291,181; $P$ = 0.0052); and suggestive significance in all panels combined (rs3760650 at 19:10,515,804; $P$ = 9.50×10$^{-6}$). Permutation testing was not applied owing to futility. Nevertheless, fixed effect meta-analysis of the European and Asian lnORs did not identify significant heterogeneity in the lnORs for rs3760650 ($P_{\text{het meta}}$ =0.475). (Note that rs2304256 was not tested in the Asian panels, nor rs16999593 in the European panels.) We consider the signal at 19p13.2 to be well supported in European populations. Conversely, the study was most likely under-powered to reliably confirm or refute association in Asian populations, or trans-ethnic heterogeneity, at this locus.

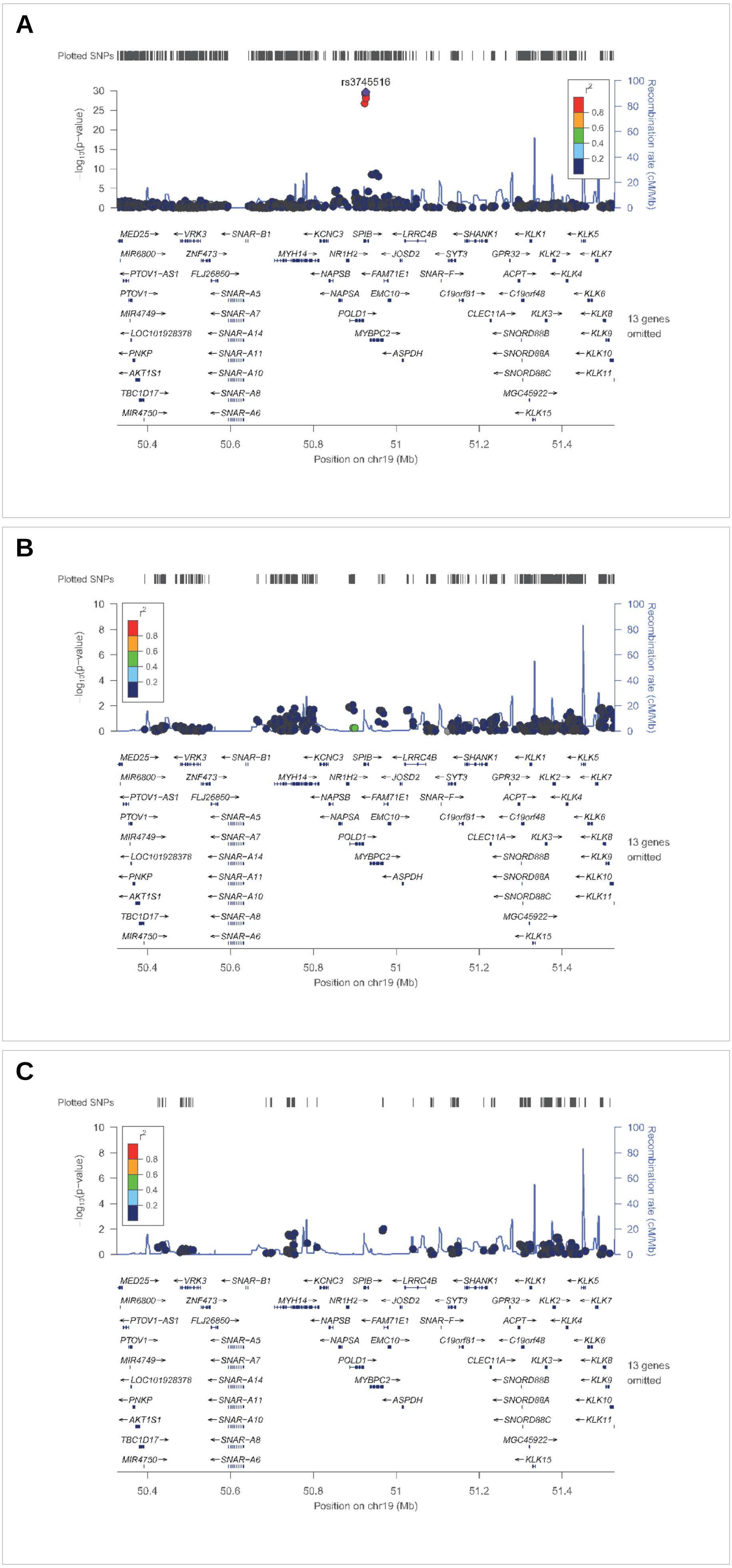


**Fig. S2.55: LocusZoom plots of the known PBC risk locus 19q13.33.** (A) European panels, (B) Asian panels, and (C) all panels combined. Association at this locus reached genome-wide significance in the European panels (rs3745516 at 19:50,926,742; $P$ = 2.65×10$^{-30}$); nominal significance in the Asian panels (rs3219325 at 19:50,895,161; $P$ = 0.0087); and nominal significance in all panels combined (rs1274604 at 19:50,969,325; $P$ = 9.41×10$^{-3}$). Permutation testing was not applied owing to futility. Fixed effect meta-analysis of the European and Asian lnORs did not identify significant heterogeneity in the lnORs for rs1274604 ($P_{\text{het meta}}$ = 0.371). (Note that rs3745516 was not tested in the Asian panels, nor rs3219325 in the European panels.) We consider the signal at 19q13.33 to be well supported in European populations. Conversely, the study was most likely under-powered to reliably confirm or refute association in Asian populations, or trans-ethnic heterogeneity, at this locus.

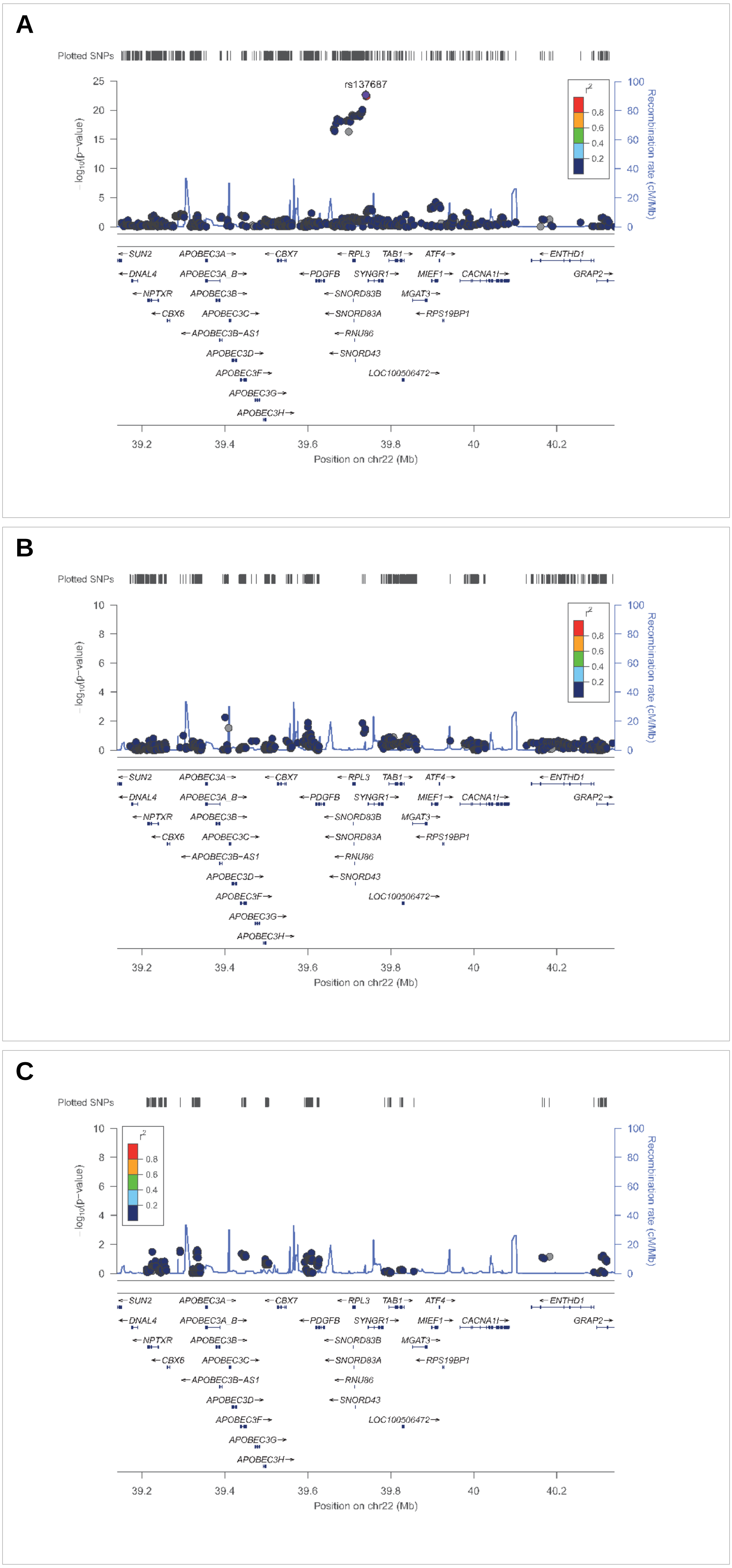


**Fig. S2.56: LocusZoom plots of the known PBC risk locus 22q13.1.** (A) European panels, (B) Asian panels, and (C) all panels combined. Association at this locus reached genome-wide significance in the European panels (rs137687 at 22:39,740,078; *P* = $2.37\times10^{-23}$); nominal significance in the Asian panels (rs118047897 at 22:39,400,858; *P* = 0.006); and nominal significance in all panels combined (rs13054016 at 22:39,333,742; *P* = 0.024). Permutation testing was not applied owing to futility. Nevertheless, fixed effect meta-analysis of the European and Asian lnORs did not identify significant heterogeneity in the lnORs for rs13054016 ($P_{\text{het meta}}$ = 0.371). (Note that rs137687 was not tested in the Asian panels, nor rs118047897 in the European panels.) On balance, we consider the signal at 19q13.33 to be well supported in European populations. Conversely, the study was most likely under-powered to reliably confirm or refute association in Asian populations, or trans-ethnic heterogeneity, at this locus.

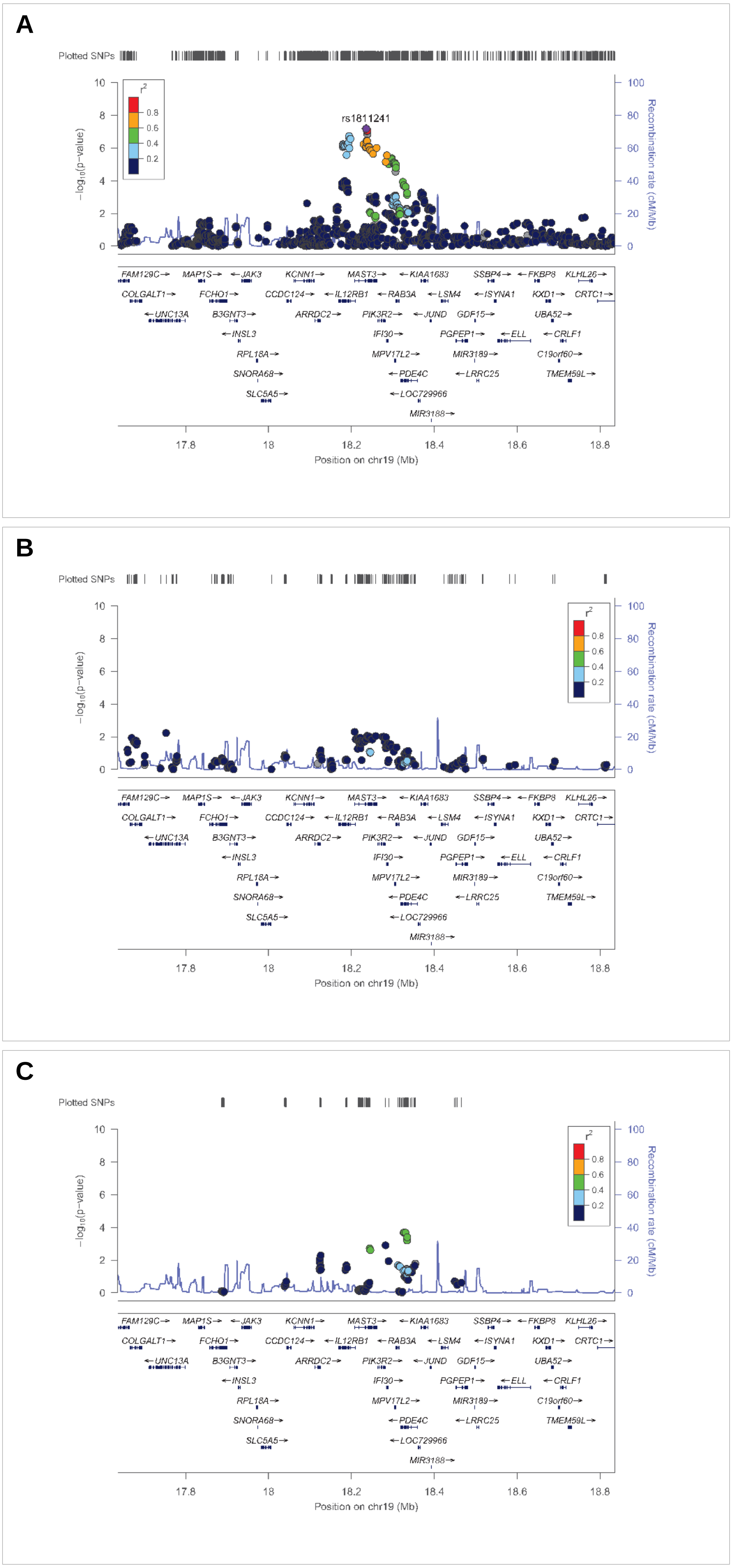


**Fig. S2.57: LocusZoom plots of the newly identified PBC risk locus 19p13.11.** (A) European panels, (B) Asian panels, and (C) all panels combined. Association at this locus reached genome-wide significance in conditional analysis of the European panels using COJO (rs1811241 at 19:18235882; P = $4.66\times10^{-8}$), having just missed genome-wide significance in the main unconditional analysis (*P* = $6.55\times10^{-8}$). There was no significant association in the Asian panels.

**Fig. S3: Previously identified PBC risk loci not reaching genome-wide significance in the current study.**

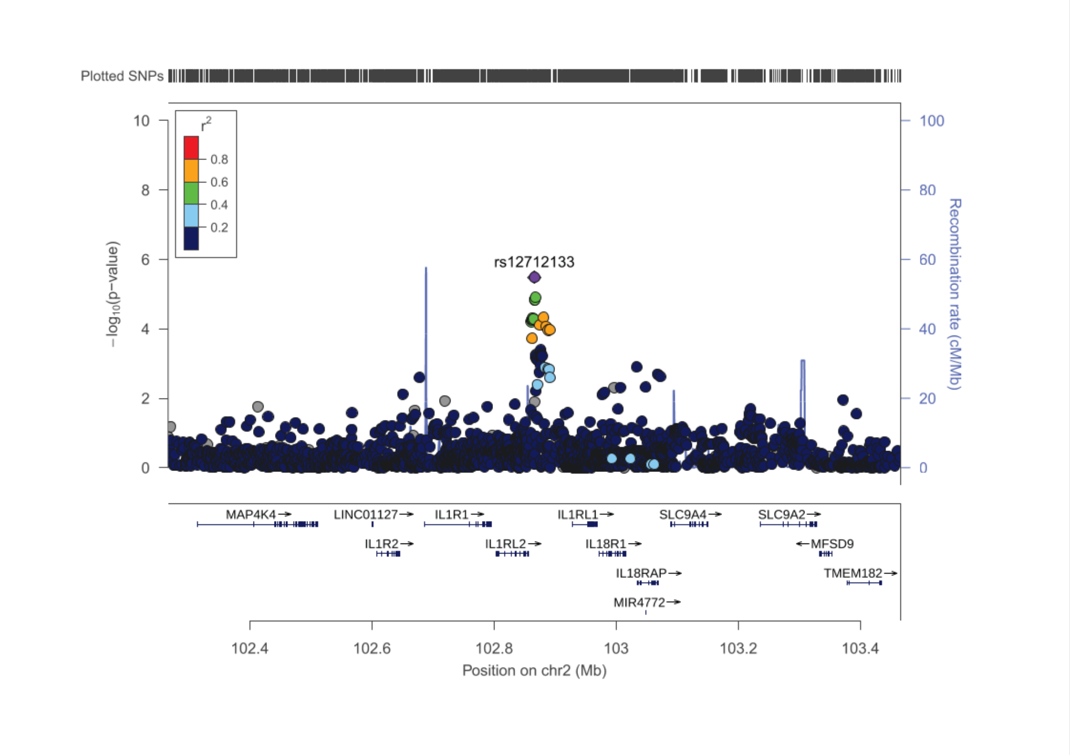


**Fig. S3.1: LocusZoom plot of the previously identified PBC risk locus 2q12.1 in the European panels**. There is an unequivocal association signal, albeit not reaching genome-wide significance (rs12712133 at 2:102,866,273; $P = 3.26\times10^{-6}$).

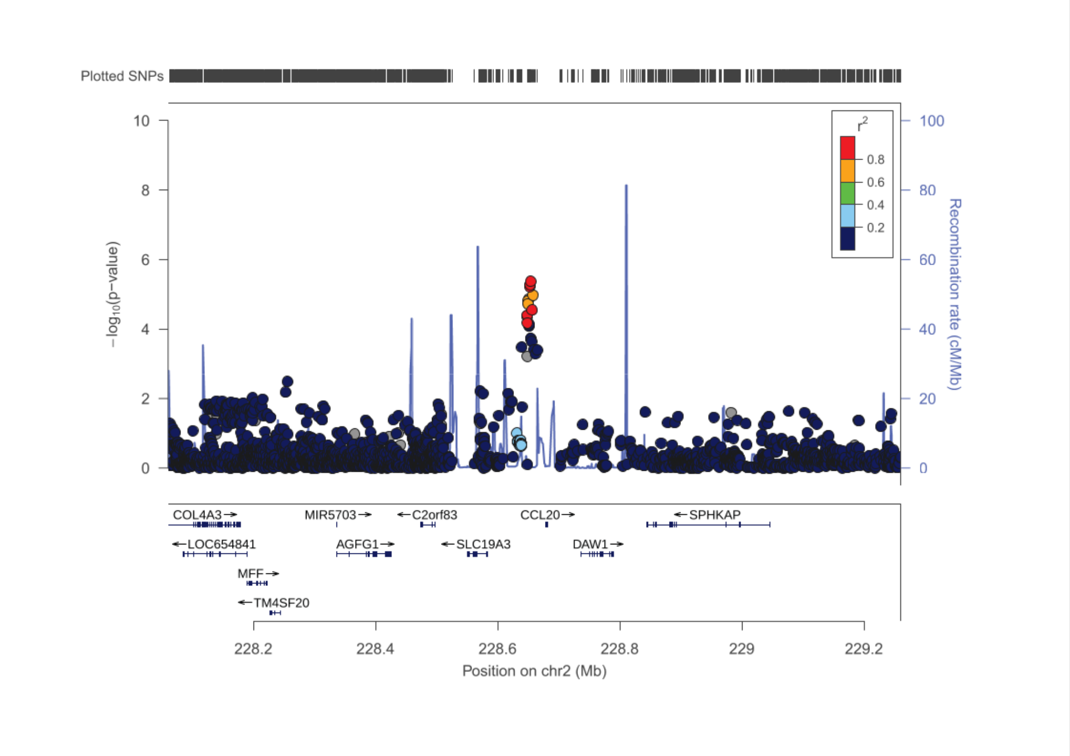


**Fig. S3.2: LocusZoom plot of the previously identified PBC risk locus 2q36.3 in the European panels.** There is an unequivocal association signal, albeit not reaching genome-wide significance (rs4973334 at 2:228,654,079; $P = 4.22\times10^{-6}$).

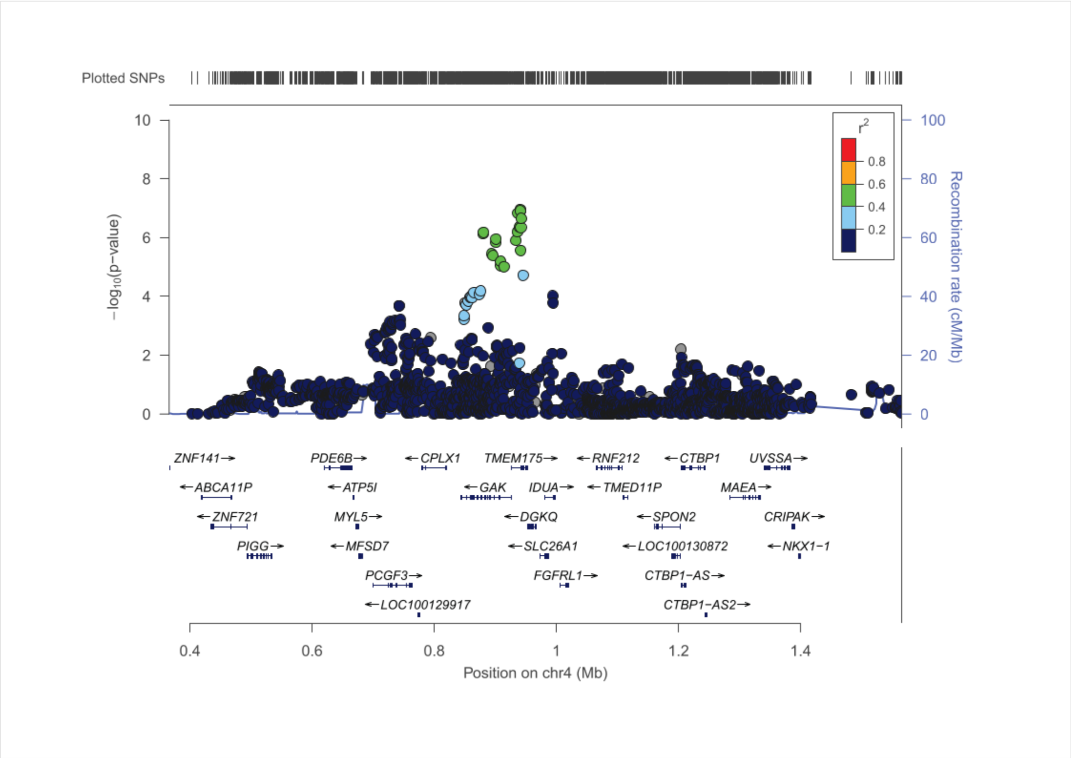


**Fig. S3.3: LocusZoom plot of the previously identified PBC risk locus 4p16.3 in the European panels.** There is an unequivocal association signal, albeit not reaching genome-wide significance (rs6848199 at 4:940,838; $P$ = 1.10×10$^{-7}$).

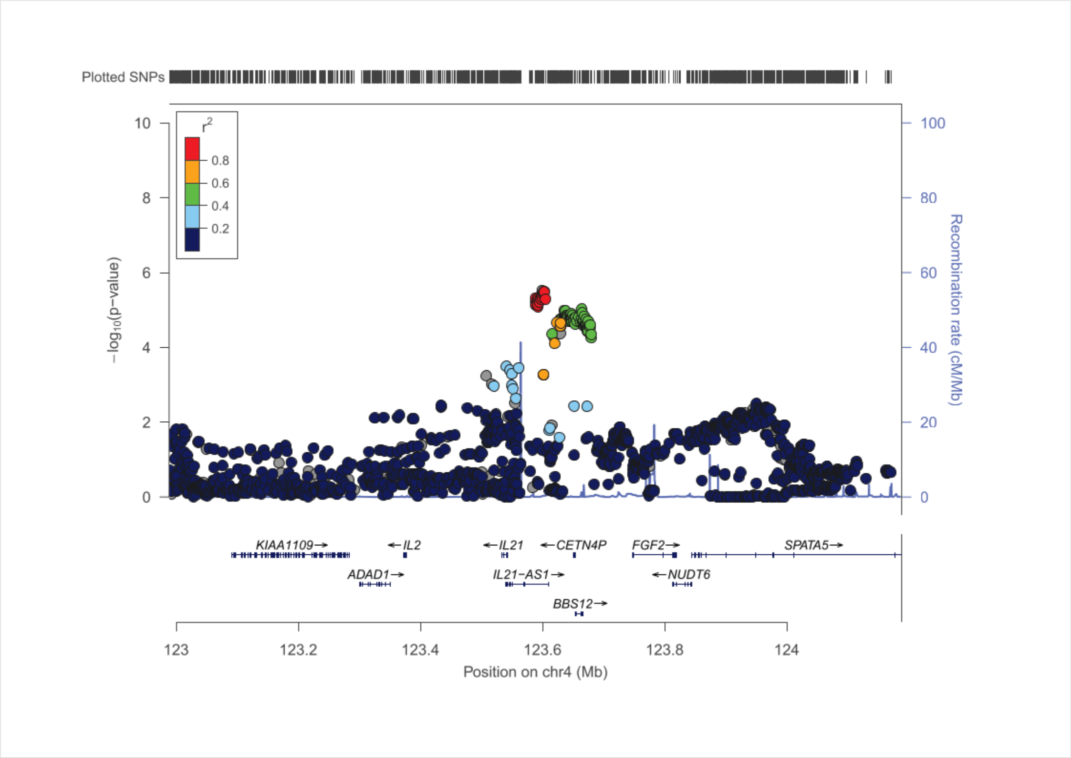


**Fig. S3.4: LocusZoom plot of the previously identified PBC risk locus 4q27 in the Asian panels.** There is an unequivocal association signal, albeit not reaching genome-wide significance (rs75802552 at 4:123,599,321; $P$ = 2.98×10$^{-6}$).

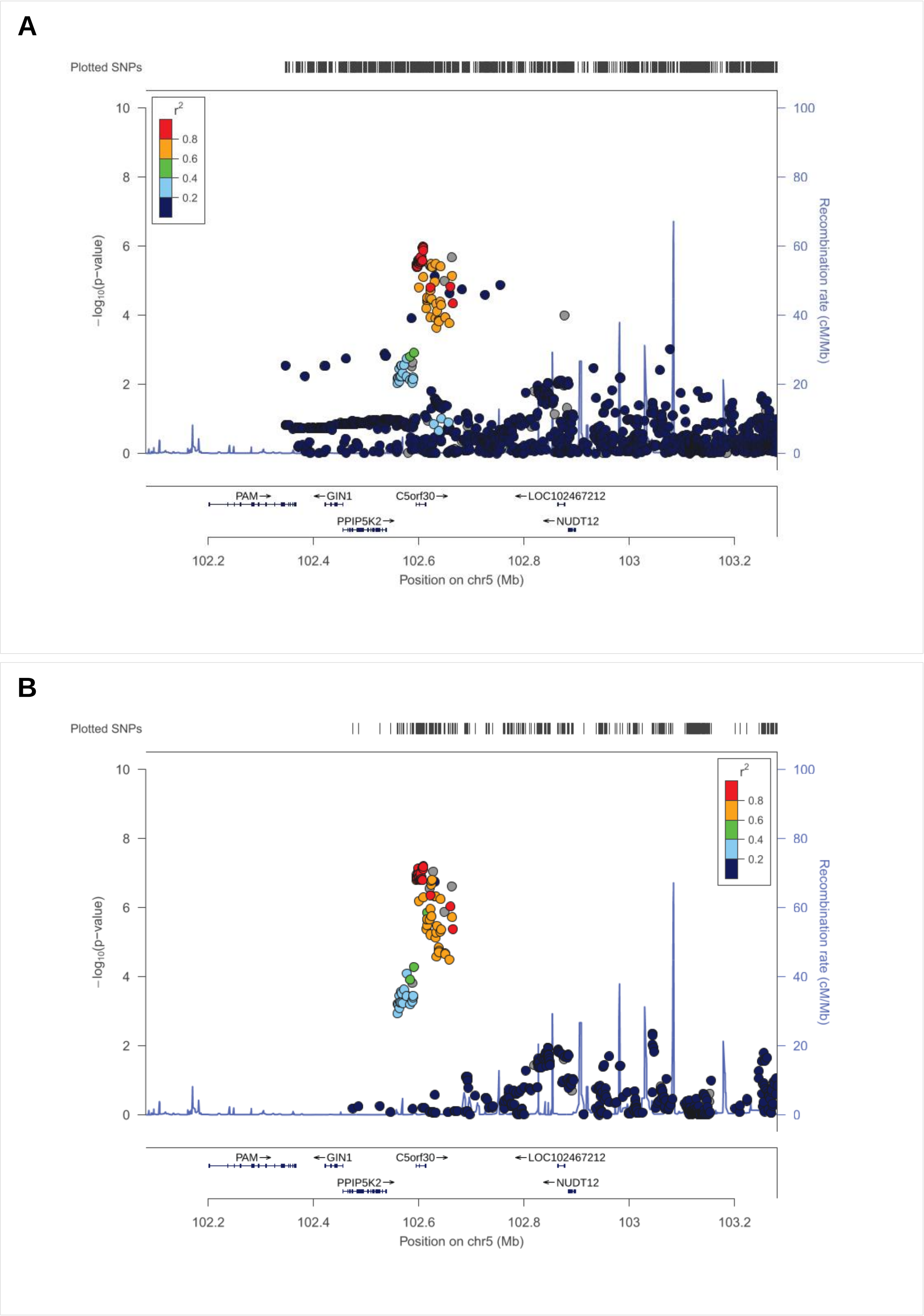


**Fig. S3.5: LocusZoom plots of the previously identified PBC risk locus 5q21.1.** (A) European panels and (B) all panels combined. There is an unequivocal association signal, albeit not reaching genome-wide level of significance.

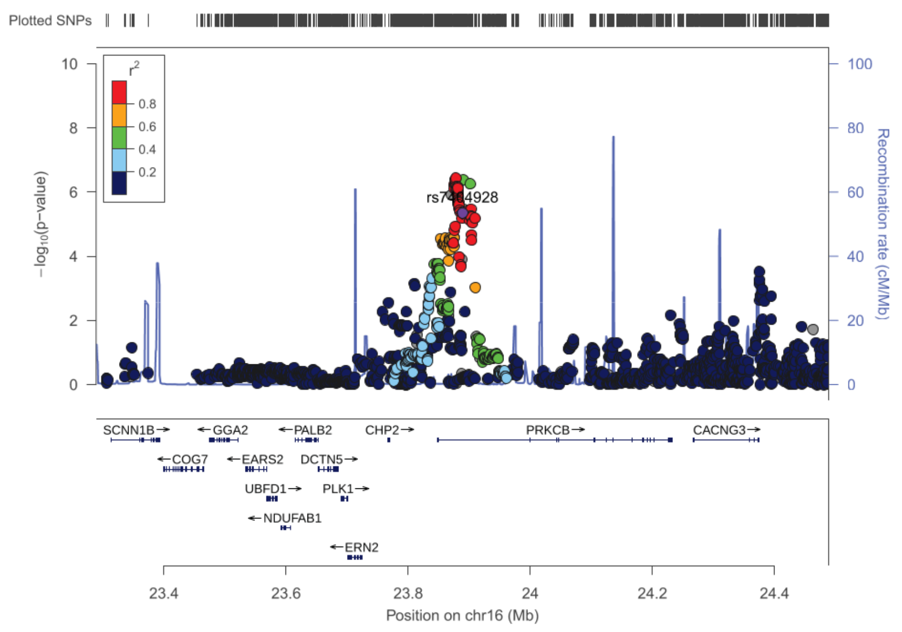


**Fig. S3.6: LocusZoom plot of the previously identified PBC risk locus 16p12.2 in the Asian panels**. There is an unequivocal association signal, albeit not reaching genome-wide significance (rs8044722 at 16:23,878,119; $P = 3.63\times10^{-7}$).

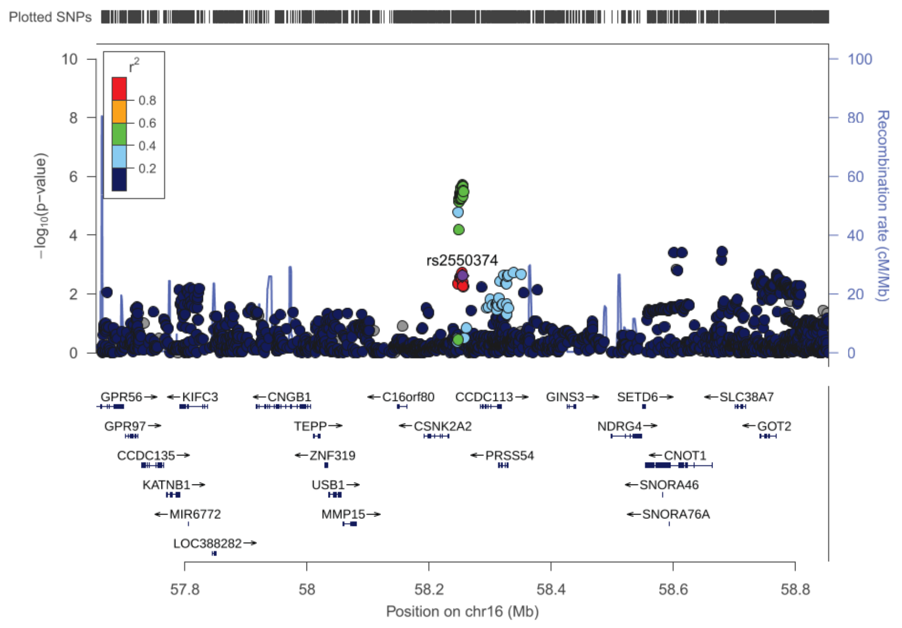


**Fig. S3.7: LocusZoom plot of the previously identified PBC risk locus 16q21 in the European panels.** There is an unequivocal association signal, albeit not reaching genome-wide significance (rs2550373 at 16:58,254,348; $P = 1.96\times10^{-6}$).

**This page is intentionally left blank.**

# Fig. S4: Comparison of original results with those with no GC correction

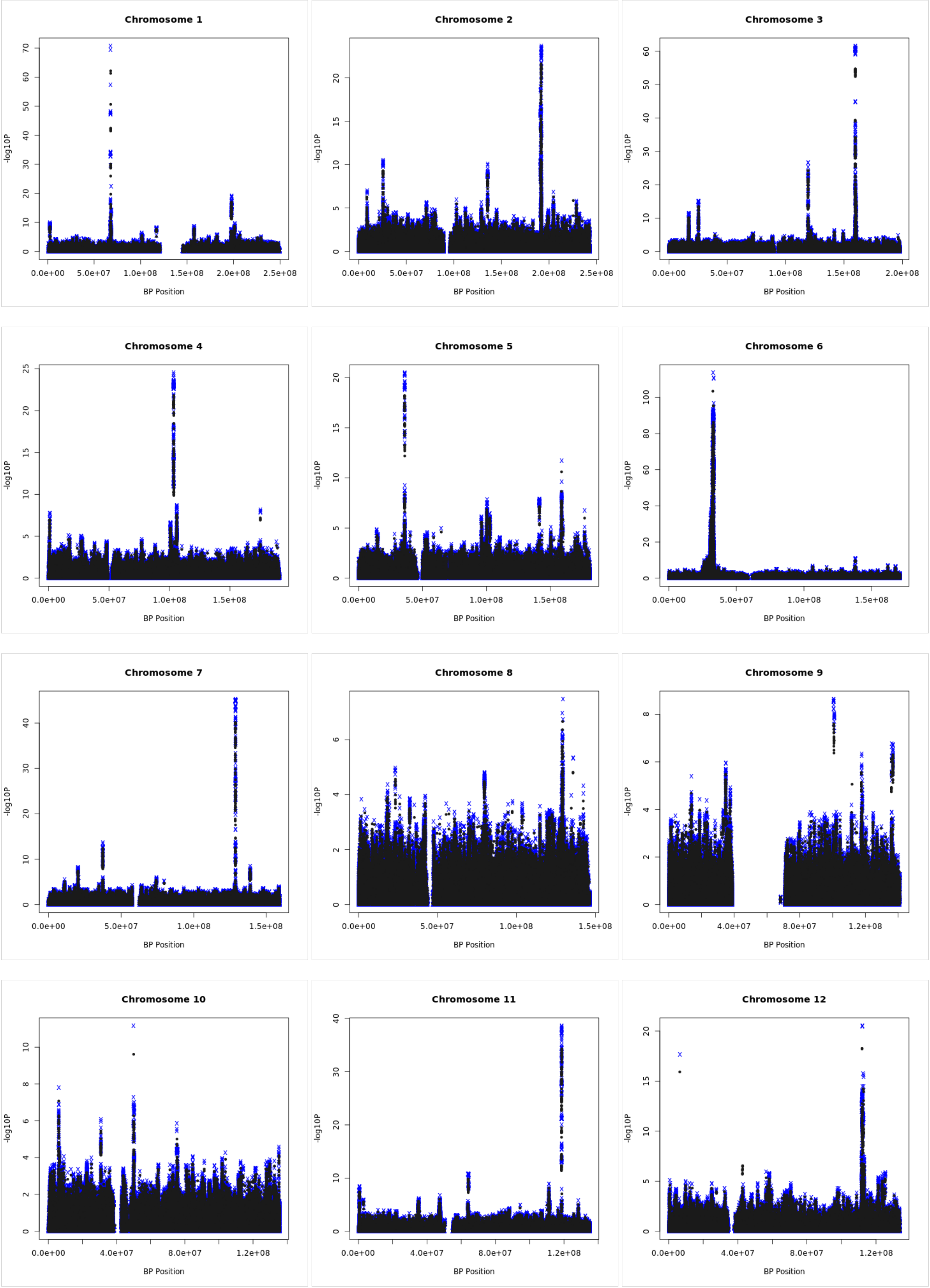

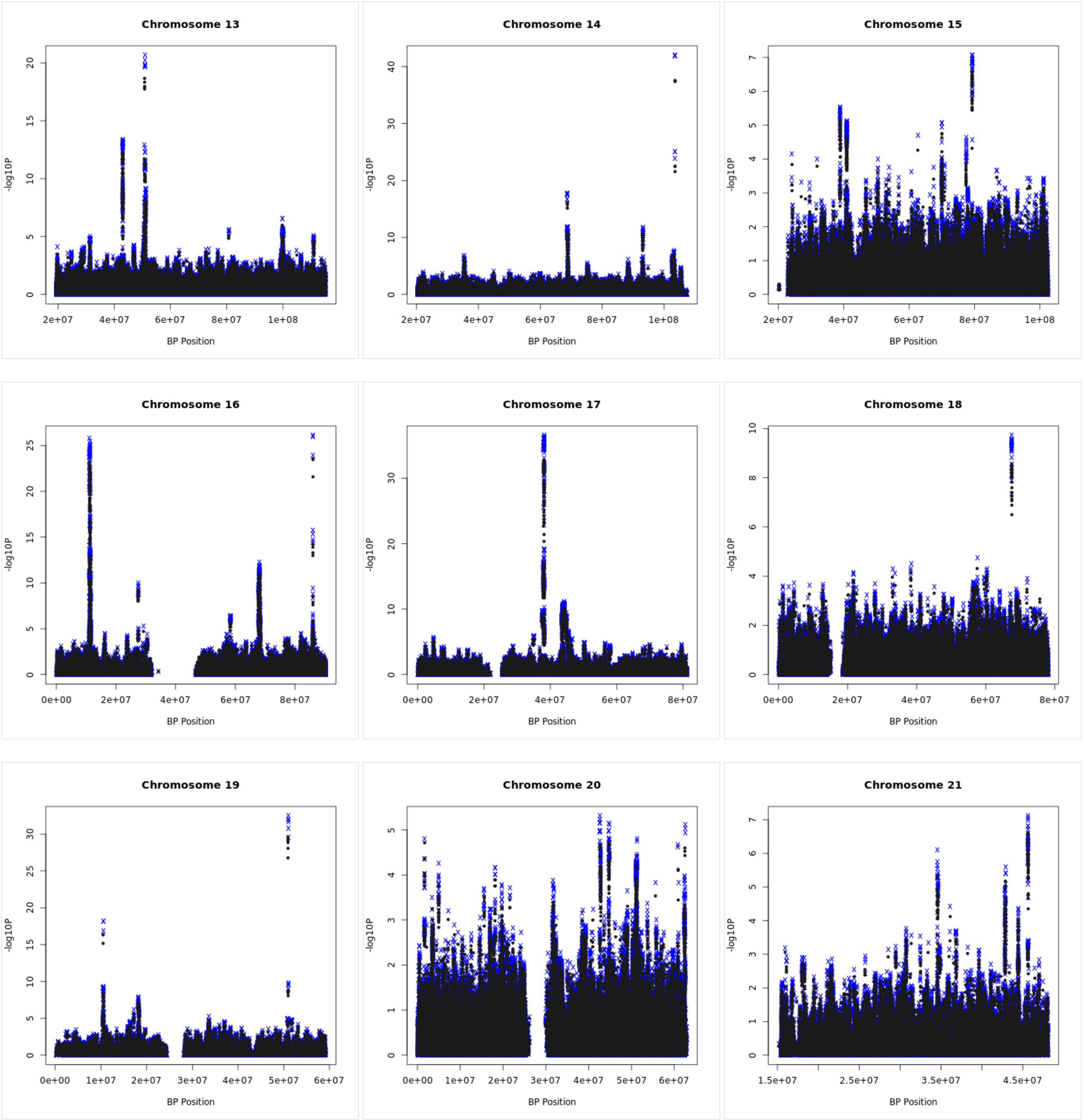


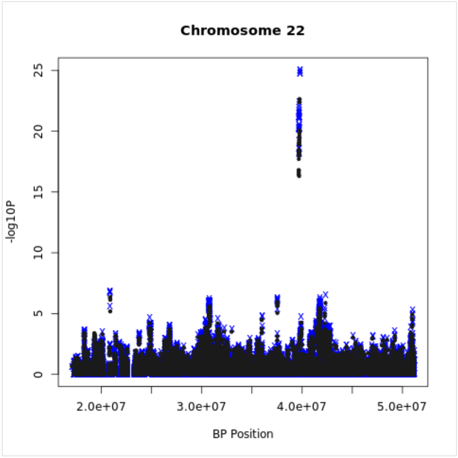


**Fig. S4.1: Comparison of original meta-analysis results for the European panels with those obtained when using no GC (or GMMAT-derived) correction**. Original results are shown as solid black circles while those obtained when using no GC (or GMMAT-derived) correction are shown as blue crosses. As expected, all loci identified as genome-wide significant with GC (or GMMAT) correction reached slightly higher levels of significance without it, while a few loci that did not reach genome-wide significance with GC (or GMMAT) correction, (just) did so without it: *i.e.*, 4p16.3 (harbouring *TMEM175*), 4q34.1, 10p15.1 (harbouring *IL2RA*), and 19p13.11 (harbouring *IL12RB1*).

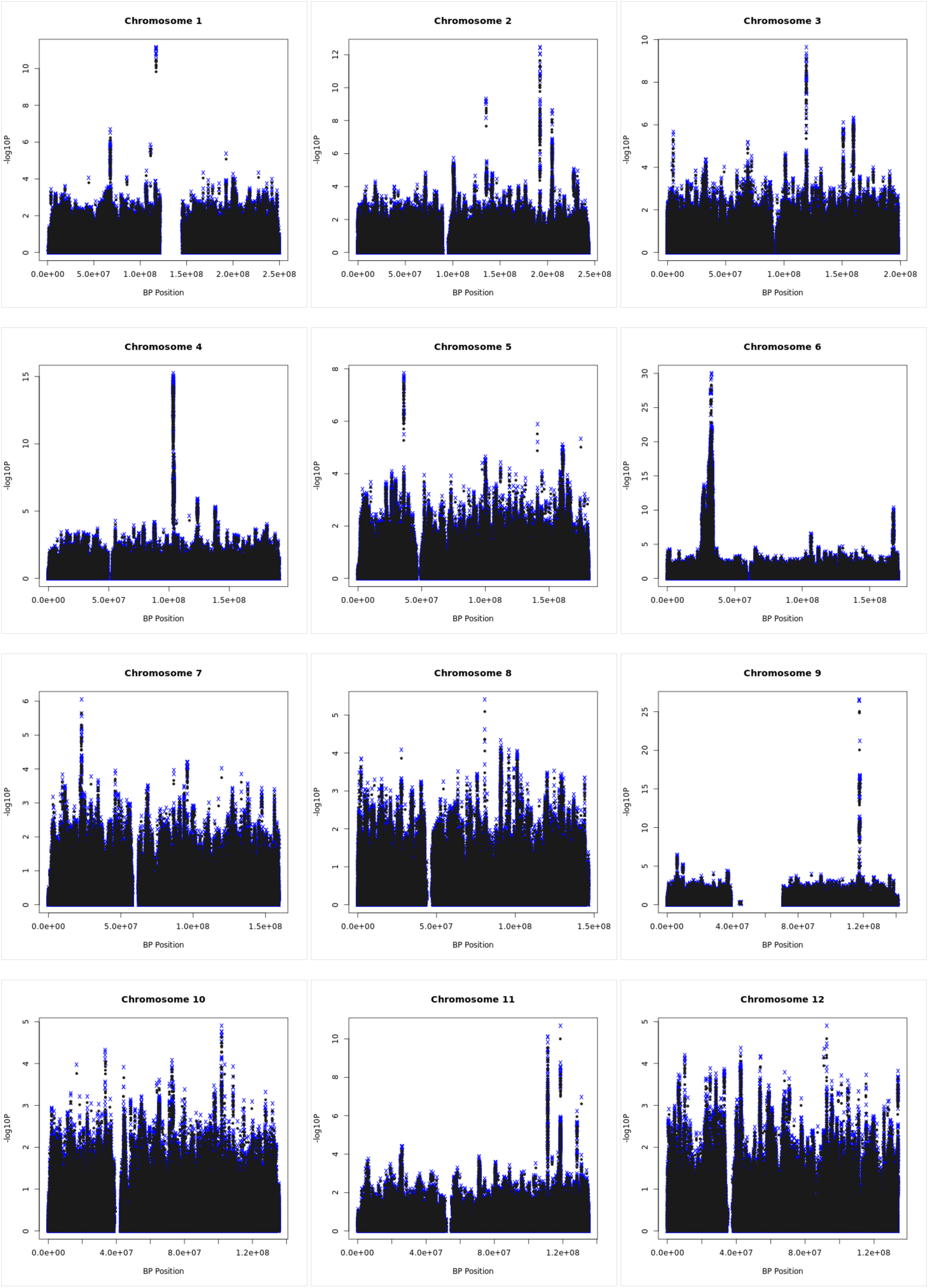
Chromosome 1
-log10P
0 2 4 6 8 10
0.0e+00 5.0e+07 1.0e+08 1.5e+08 2.0e+08 2.5e+08
BP Position
Chromosome 2
-log10P
0 2 4 6 8 10 12
0.0e+00 5.0e+07 1.0e+08 1.5e+08 2.0e+08 2.5e+08
BP Position
Chromosome 3
-log10P
0 2 4 6 8 10
0.0e+00 5.0e+07 1.0e+08 1.5e+08 2.0e+08
BP Position
Chromosome 4
-log10P
0 5 10 15
0.0e+00 5.0e+07 1.0e+08 1.5e+08
BP Position
Chromosome 5
-log10P
0 2 4 6 8
0.0e+00 5.0e+07 1.0e+08 1.5e+08
BP Position
Chromosome 6
-log10P
0 5 10 15 20 25 30
0.0e+00 5.0e+07 1.0e+08 1.5e+08
BP Position
Chromosome 7
-log10P
0 1 2 3 4 5 6
0.0e+00 5.0e+07 1.0e+08 1.5e+08
BP Position
Chromosome 8
-log10P
0 1 2 3 4 5
0.0e+00 5.0e+07 1.0e+08 1.5e+08
BP Position
Chromosome 9
-log10P
0 5 10 15 20 25
0.0e+00 4.0e+07 8.0e+07 1.2e+08
BP Position
Chromosome 10
-log10P
0 1 2 3 4 5
0.0e+00 4.0e+07 8.0e+07 1.2e+08
BP Position
Chromosome 11
-log10P
0 2 4 6 8 10
0.0e+00 4.0e+07 8.0e+07 1.2e+08
BP Position
Chromosome 12
-log10P
0 1 2 3 4 5
0.0e+00 4.0e+07 8.0e+07 1.2e+08
BP Position

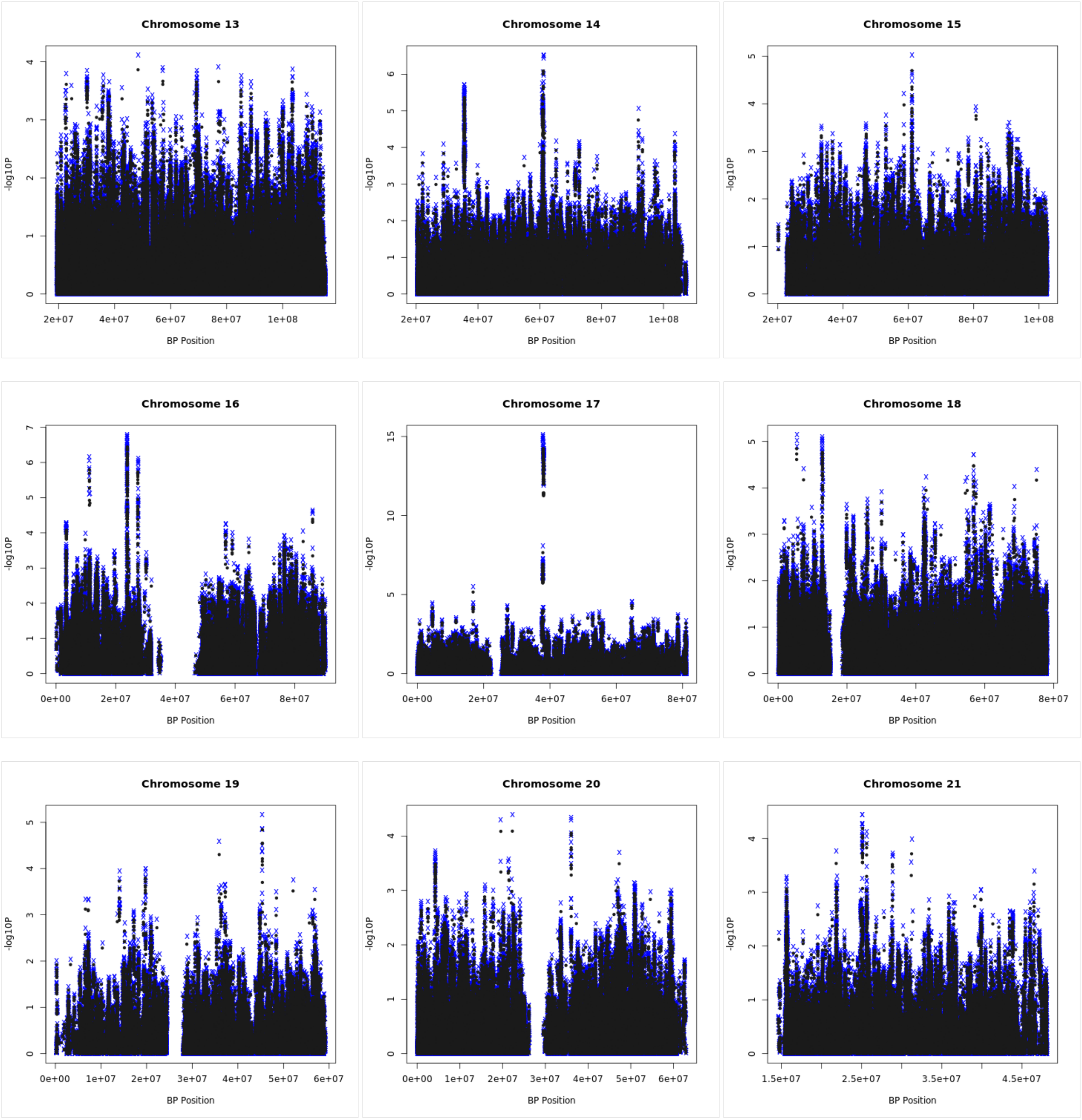


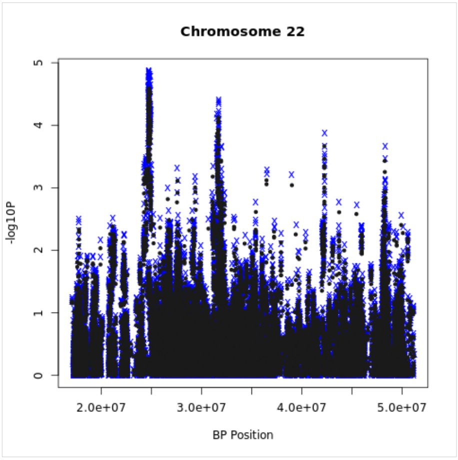


**Fig. S4.2: Comparison of original meta-analysis results for the Asian panels with those obtained when using no GC (or GMMAT-derived) correction.** Original results are shown as solid black circles while those obtained when using no GC (or GMMAT-derived) correction are shown as blue crosses. As expected, all loci identified as genome-wide significant with GC (or GMMAT) correction reached slightly higher levels of significance without it.

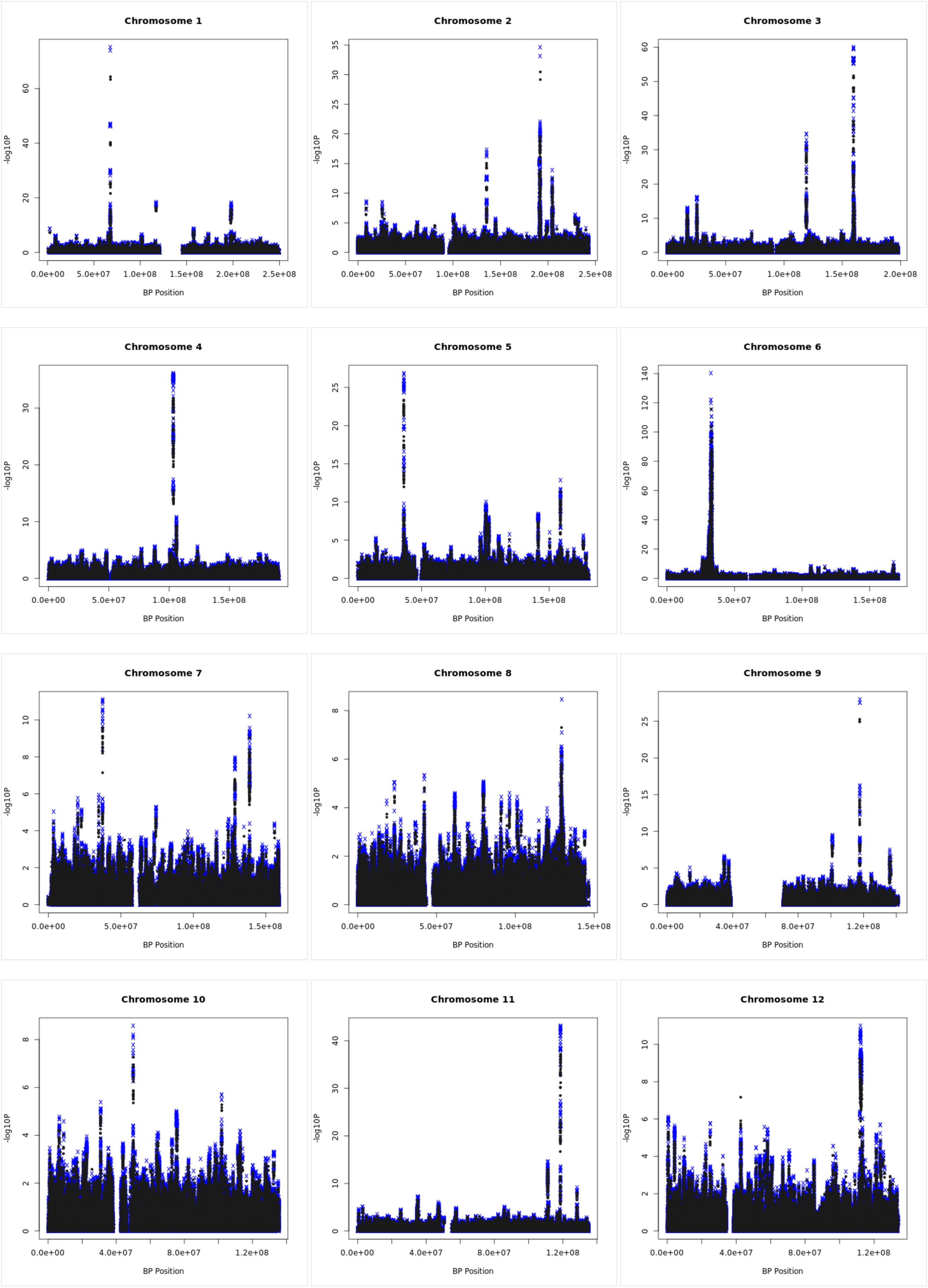
Chromosome 1
Chromosome 2
Chromosome 3
Chromosome 4
Chromosome 5
Chromosome 6
Chromosome 7
Chromosome 8
Chromosome 9
Chromosome 10
Chromosome 11
Chromosome 12
-log10P
BP Position

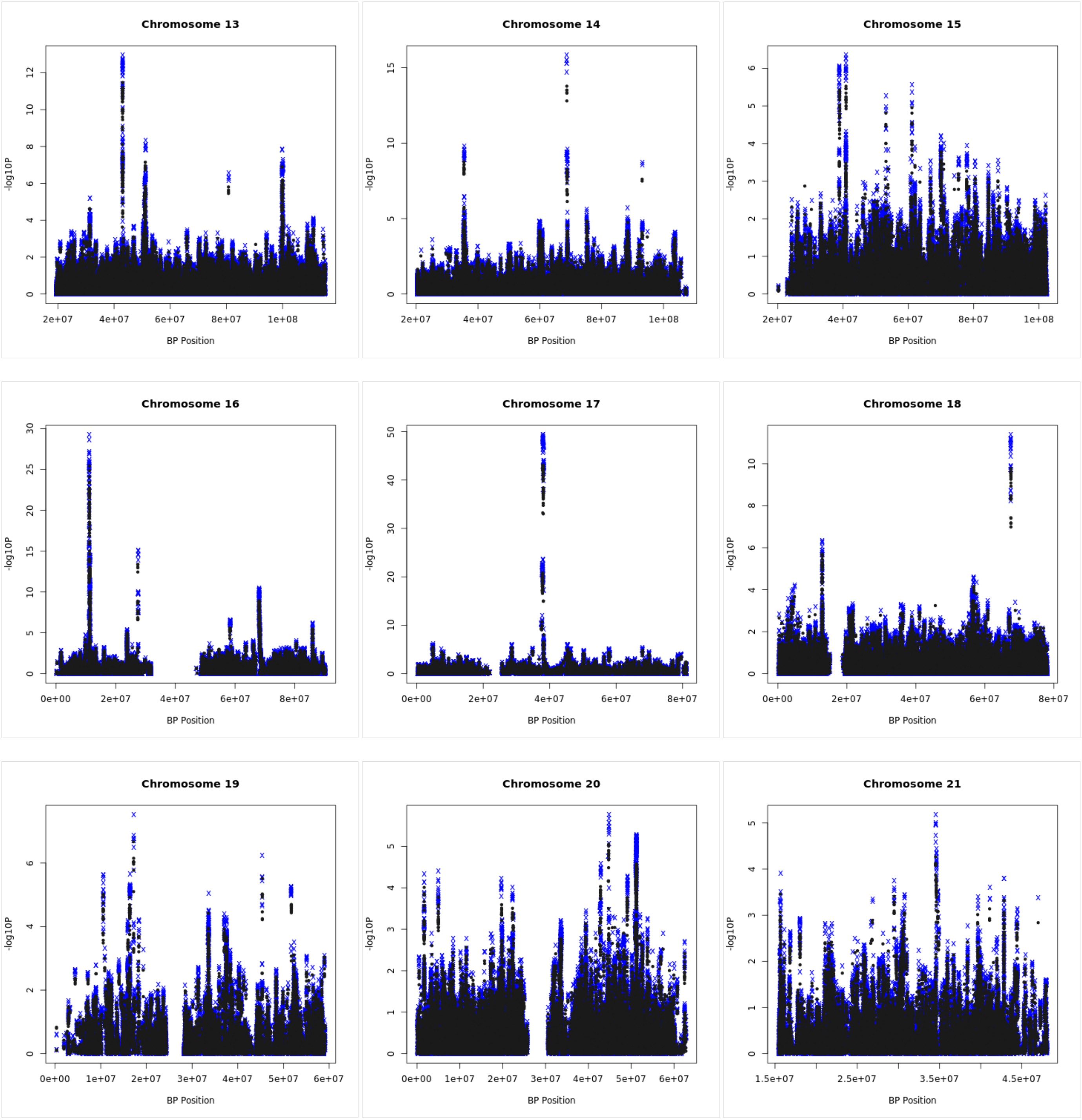


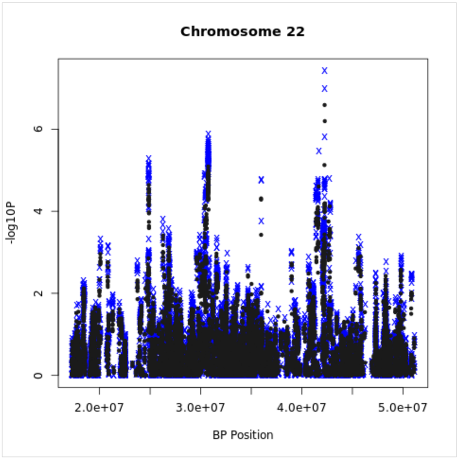


**Fig. S4.3: Comparison of original meta-analysis results for all panels combined with those obtained when using no GC (or GMMAT-derived) correction**. Original results are shown as solid black circles while those obtained when using no GC (or GMMAT-derived) correction are shown as blue crosses. As expected, all loci identified as genome-wide significant with GC (or GMMAT) correction reached slightly higher levels of significance without it, while a few loci that did not reach genome-wide significance with GC (or GMMAT) correction, (just) did so without it: *i.e.*, 5q21.1 (harbouring *MACIR*), 6q22.1, 9q34.13, 11p13, 13q32.3, 19p13.11 (harbouring *HAUS8*), and 22q13.2.

## Fig. S5: Comparison with MR-MEGA (all panels combined)

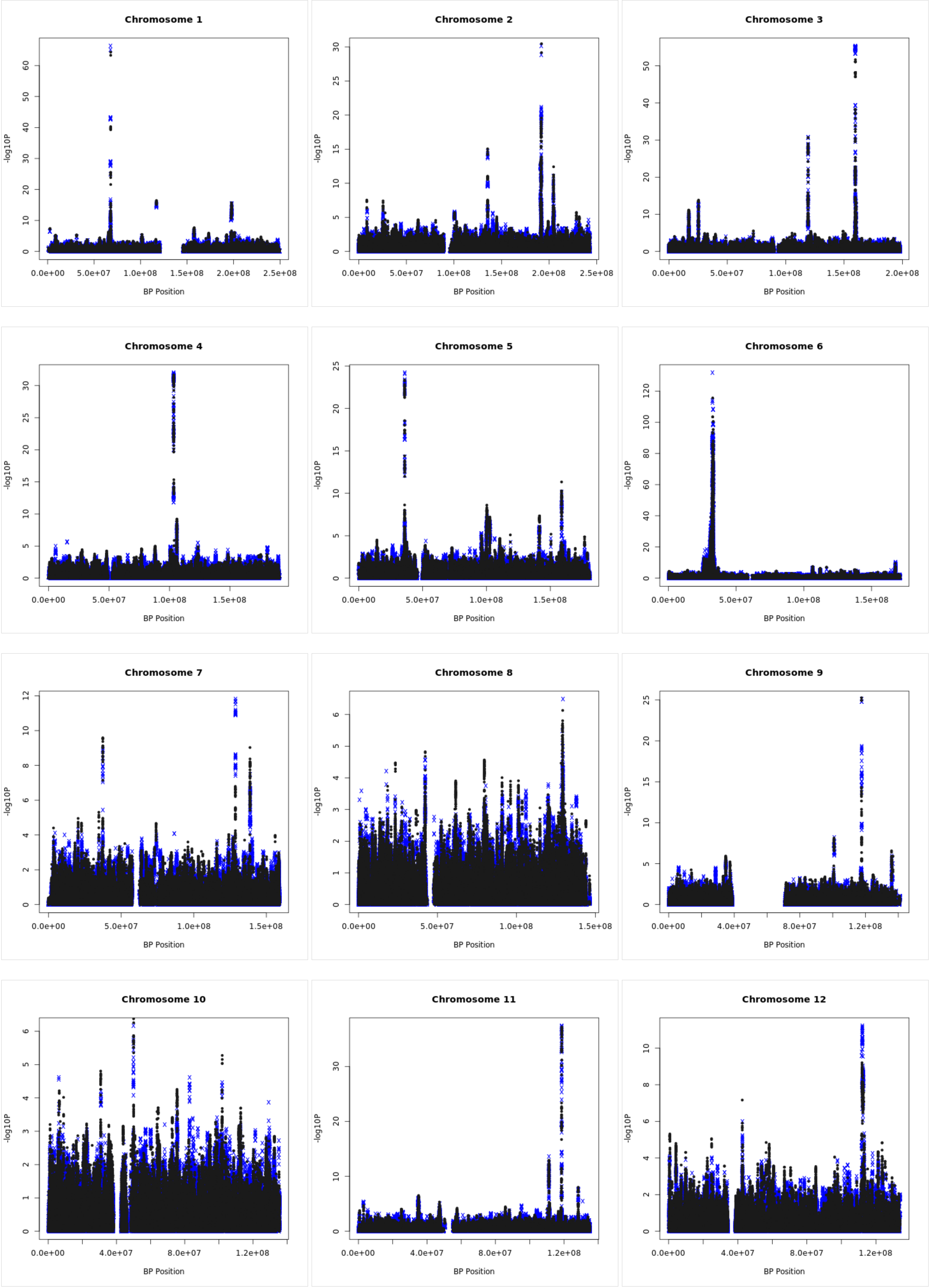

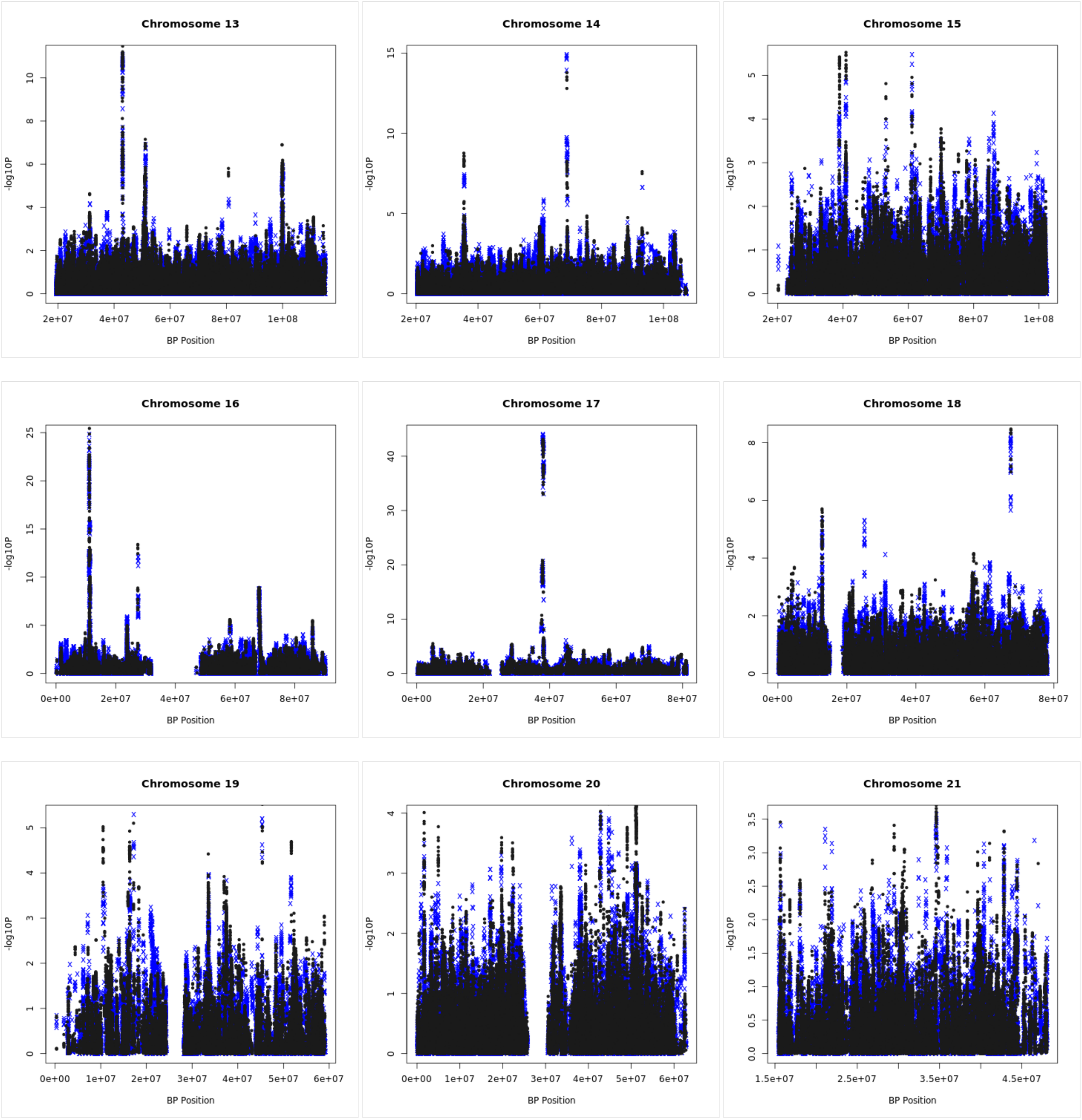


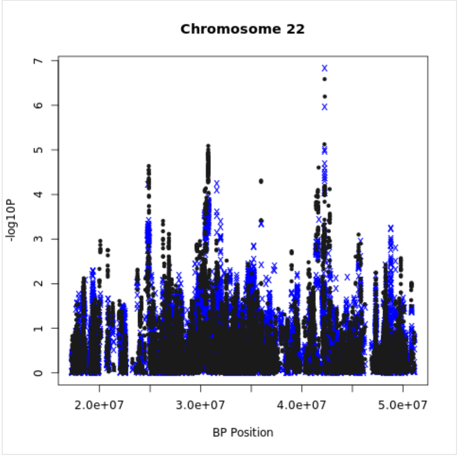


**Fig. S5: Comparison of original combined European and Asian panels meta-analysis results with those obtained when using trans-ethnic analysis implemented in MR-MEGA.** Original results are shown as solid black circles while those obtained when using MR-MEGA are shown as blue crosses.

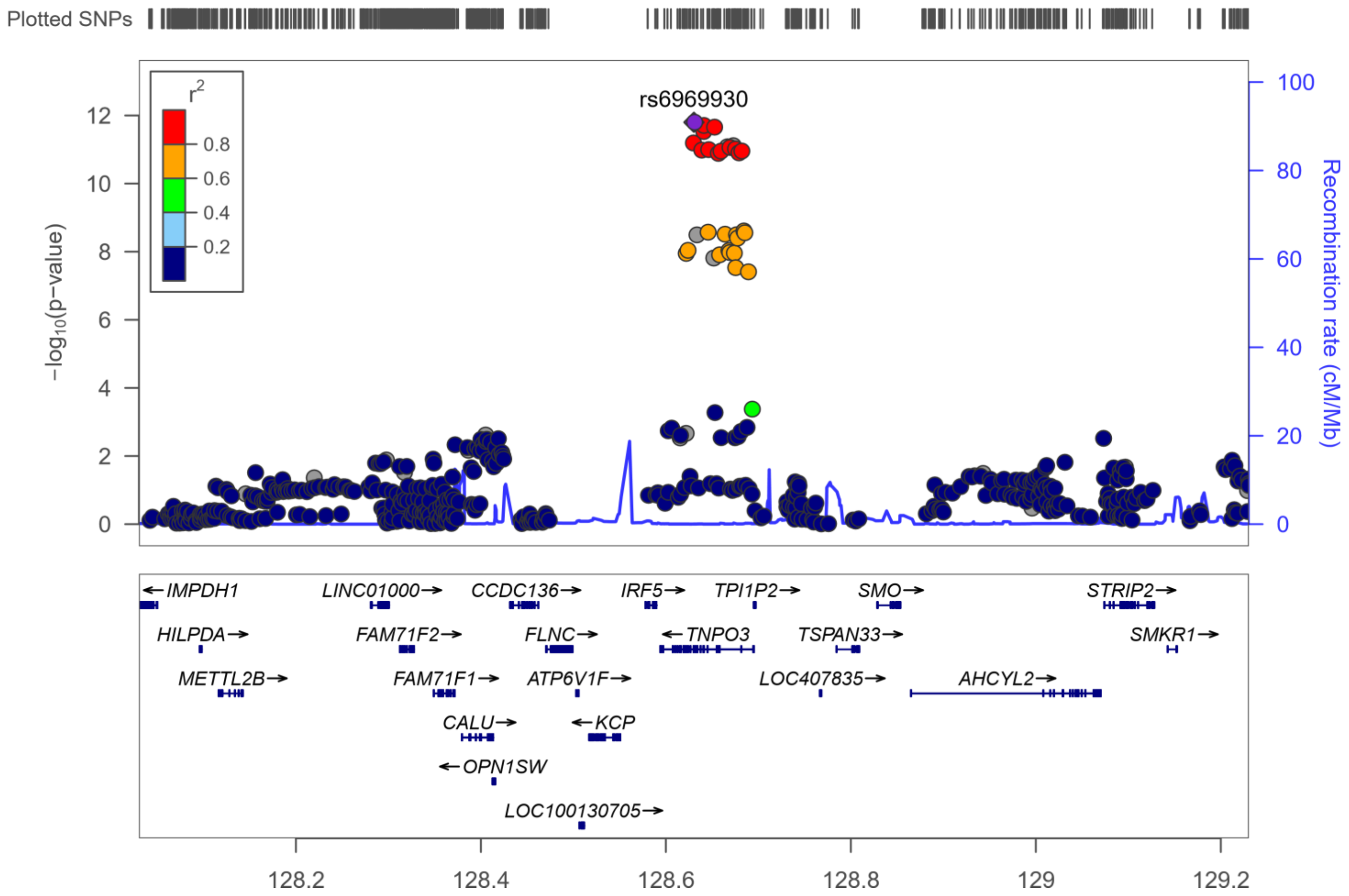


**Fig. S6: LocusZoom plot of the independent, genome-wide significant signal at 7q32.1 confirmed by trans-ethnic analysis implemented in MR-MEGA** (c.f. **Suppl. Figure 2.28**).

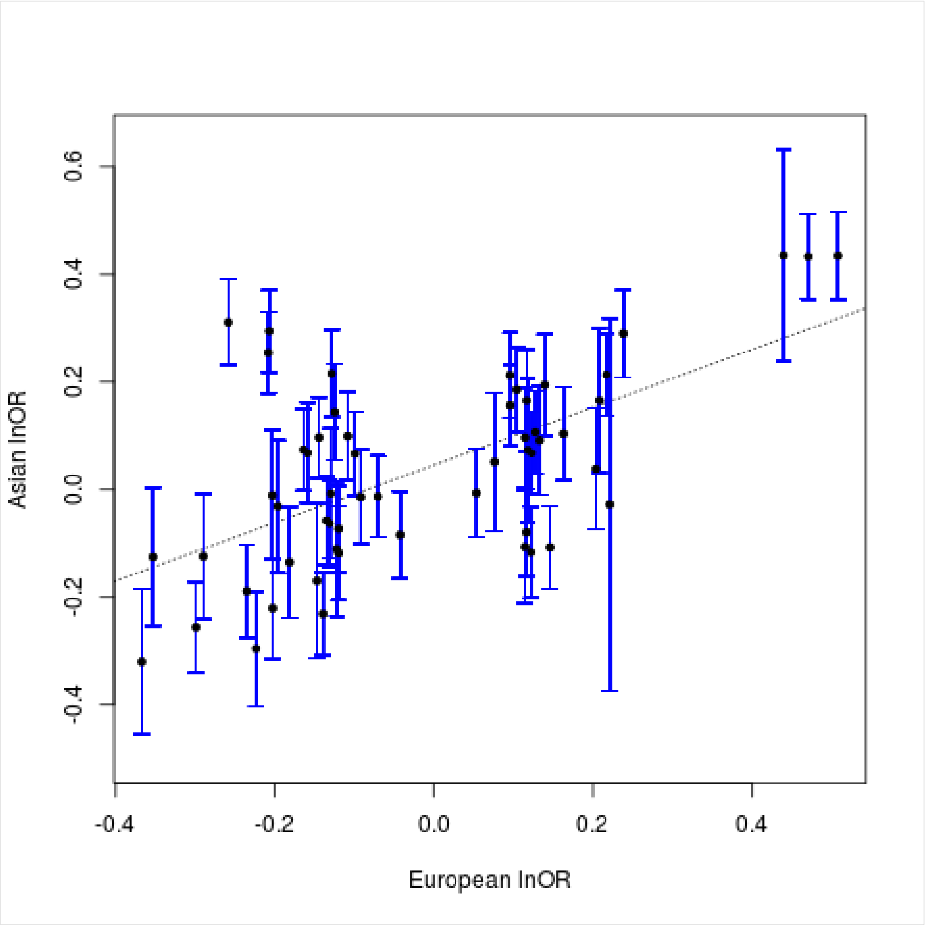


**Fig. S7: Comparison of Asian and European lnORs for the SNP scoring highest in all panels combined at each of the 56 loci listed in Suppl. Table 1.** Point estimates of lnORs are shown as solid black circles while the 95% confidence intervals for the Asian lnORs are shown as blue vertical lines. The overall correlation between the Asian and European lnORs was 0.604 while the best fit regression line (shown as a dotted black line) had estimated slope 0.537.

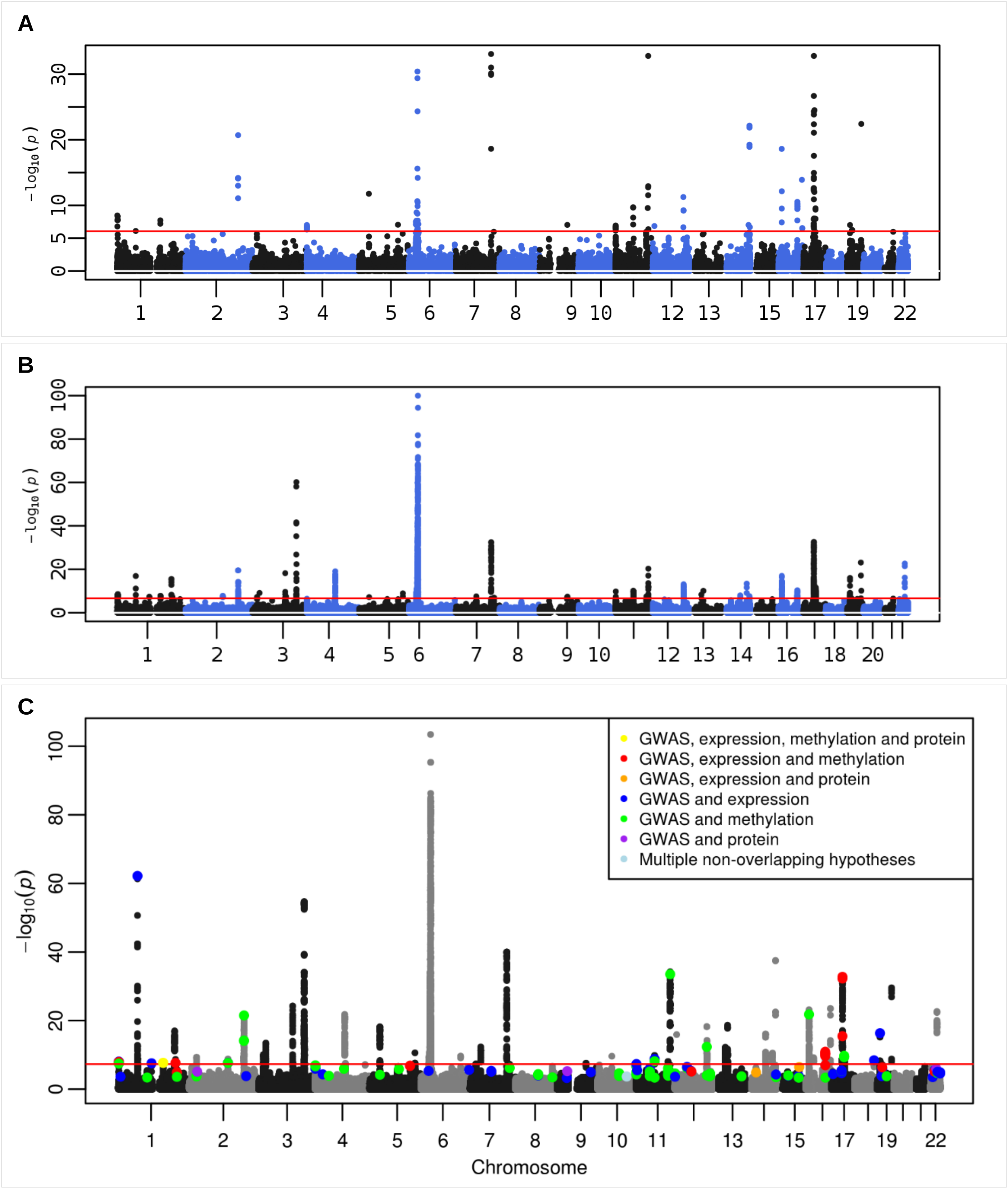


**Fig. S8. Manhattan plots from the MWAS, TWAS, and moloc co-localisation.** (A) Methylome-wide association study (MWAS), (B) transcriptome-wide association study (TWAS), and (C) moloc colocalization of association signals from the genome-wide meta-analysis (GWMA) of European panels with those from mapping of methylation (mQTL), expression (eQTL) and serum proteome quantitative trait loci (pQTL) in healthy reference panels. See **Main** and **Supplementary Text** for details.

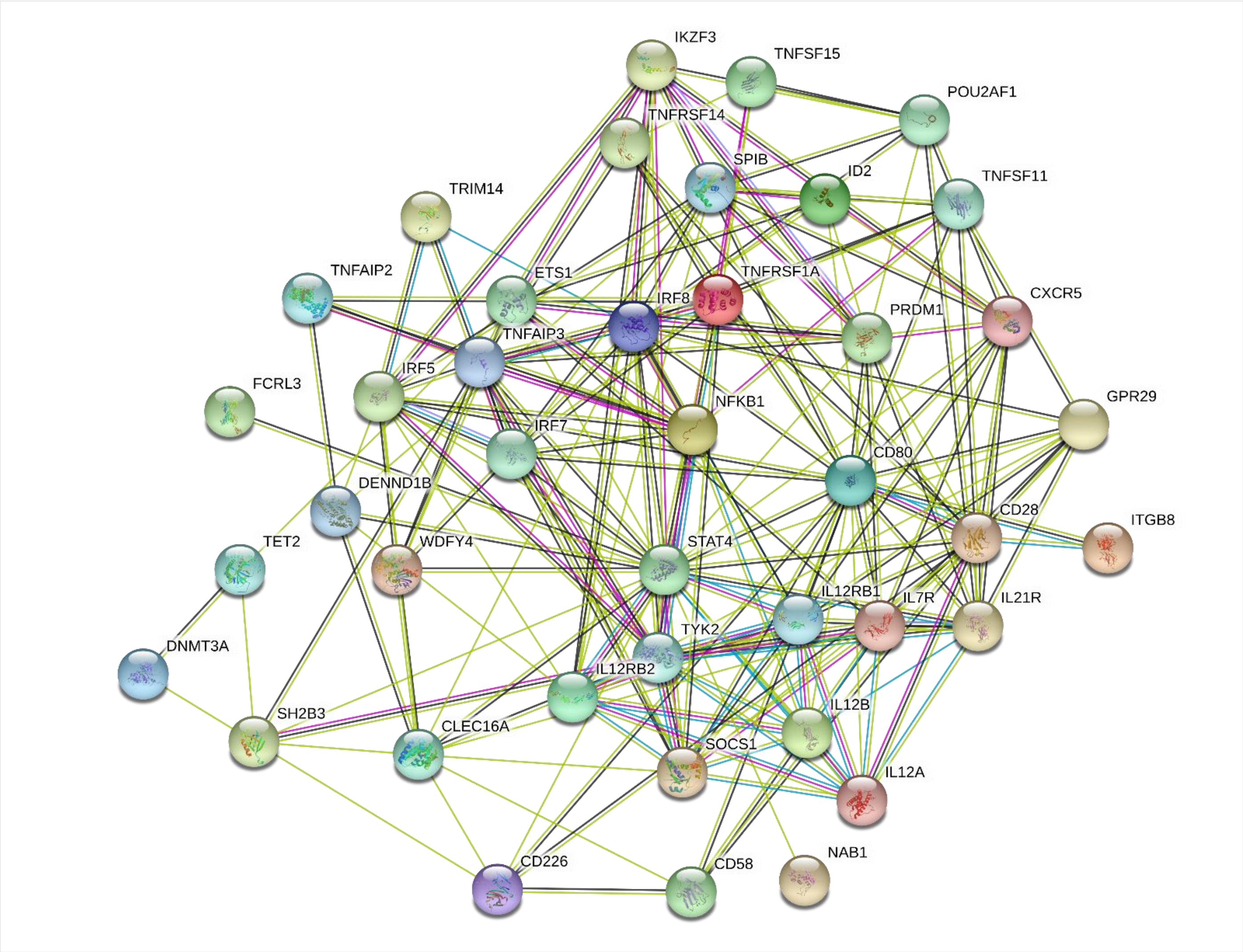


**Fig. S9: Protein-protein interaction (PPI) plot from STRING.** Note that candidate genes for PBC constitute a closely related gene network. In STRING analysis, the PBC candidate gene network had a PPI enrichment P-value $<1\times10^{-16}$; and the network was enriched for the KEGG annotations, Cytokine-cytokine receptor interaction (FDR = $4.22\times10^{-8}$); Jak-STAT signalling pathway (FDR = $6.53\times10^{-8}$); Th1 and Th2 cell differentiation (FDR = $3.15\times10^{-7}$); Toll-like receptor signalling pathway (FDR = $1.33\times10^{-5}$); RIG-I-like receptor signalling pathway (FDR = 0.00049); NF-kappa B signalling pathway (FDR = 0.0012); Th17 cell differentiation (FDR = 0.0015); NOD-like receptor signalling pathway (FDR = 0.0066); and TNF signalling pathway (FDR = 0.0144), amongst others. This analysis can be readily reproduced at the STRING website (https://string-db.org/), using the candidate genes listed in **Suppl. Table 16**.

## References

[1] European Association for the Study of the Liver. Electronic address eee, European Association for the Study of the L. EASL Clinical Practice Guidelines: The diagnosis and management of patients with primary biliary cholangitis. J Hepatol 2017;67:145-172.
[2] Liu X, Invernizzi P, Lu Y, Kosoy R, Lu Y, Bianchi I, et al. Genome-wide meta-analyses identify three loci associated with primary biliary cirrhosis. Nat Genet 2010;42:658-660.
[3] Mells GF, Floyd JA, Morley KI, Cordell HJ, Franklin CS, Shin SY, et al. Genome-wide association study identifies 12 new susceptibility loci for primary biliary cirrhosis. Nat Genet 2011;43:329-332.
[4] Kawashima M, Hitomi Y, Aiba Y, Nishida N, Kojima K, Kawai Y, et al. Genome-wide association studies identify PRKCB as a novel genetic susceptibility locus for primary biliary cholangitis in the Japanese population. Hum Mol Genet 2017;26:650-659.
[5] Qiu F, Tang R, Zuo X, Shi X, Wei Y, Zheng X, et al. A genome-wide association study identifies six novel risk loci for primary biliary cholangitis. Nat Commun 2017;8:14828.
[6] Prins BP, Kuchenbaecker KB, Bao Y, Smart M, Zabaneh D, Fatemifar G, et al. Genome-wide analysis of health-related biomarkers in the UK Household Longitudinal Study reveals novel associations. Sci Rep 2017;7:11008.
[7] Watanabe K, Taskesen E, van Bochoven A, Posthuma D. Functional mapping and annotation of genetic associations with FUMA. Nat Commun 2017;8:1826.
[8] Bulik-Sullivan BK, Loh PR, Finucane HK, Ripke S, Yang J, Schizophrenia Working Group of the Psychiatric Genomics C, et al. LD Score regression distinguishes confounding from polygenicity in genome-wide association studies. Nat Genet 2015;47:291-295.
[9] Magi R, Horikoshi M, Sofer T, Mahajan A, Kitajima H, Franceschini N, et al. Trans-ethnic meta-regression of genome-wide association studies accounting for ancestry increases power for discovery and improves fine-mapping resolution. Hum Mol Genet 2017;26:3639-3650.
[10] Benner C, Spencer CC, Havulinna AS, Salomaa V, Ripatti S, Pirinen M. FINEMAP: efficient variable selection using summary data from genome-wide association studies. Bioinformatics 2016;32:1493-1501.
[11] Yang J, Ferreira T, Morris AP, Medland SE, Genetic Investigation of ATC, Replication DIG, et al. Conditional and joint multiple-SNP analysis of GWAS summary statistics identifies additional variants influencing complex traits. Nature genetics 2012;44:369-375, S361-363.
[12] Yang J, Lee SH, Goddard ME, Visscher PM. GCTA: a tool for genome-wide complex trait analysis. American journal of human genetics 2011;88:76-82.
[13] Purcell S, Neale B, Todd-Brown K, Thomas L, Ferreira MA, Bender D, et al. PLINK: a tool set for whole-genome association and population-based linkage analyses. Am J Hum Genet 2007;81:559-575.
[14] Liu JZ, Almarri MA, Gaffney DJ, Mells GF, Jostins L, Cordell HJ, et al. Dense fine-mapping study identifies new susceptibility loci for primary biliary cirrhosis. Nat Genet 2012;44:1137-1141.
[15] Barbeira AN, Dickinson SP, Bonazzola R, Zheng J, Wheeler HE, Torres JM, et al. Exploring the phenotypic consequences of tissue specific gene expression variation inferred from GWAS summary statistics. Nat Commun 2018;9:1825.
[16] Consortium GT. The Genotype-Tissue Expression (GTEx) project. Nature genetics 2013;45:580-585.

[17] Boyd A, Golding J, Macleod J, Lawlor DA, Fraser A, Henderson J, et al. Cohort Profile: the 'children of the 90s'--the index offspring of the Avon Longitudinal Study of Parents and Children. Int J Epidemiol 2013;42:111-127.
[18] Fraser A, Macdonald-Wallis C, Tilling K, Boyd A, Golding J, Davey Smith G, et al. Cohort Profile: the Avon Longitudinal Study of Parents and Children: ALSPAC mothers cohort. Int J Epidemiol 2013;42:97-110.
[19] Sun BB, Maranville JC, Peters JE, Stacey D, Staley JR, Blackshaw J, et al. Genomic atlas of the human plasma proteome. Nature 2018;558:73-79.
[20] Harris PA, Taylor R, Thielke R, Payne J, Gonzalez N, Conde JG. Research electronic data capture (REDCap)--a metadata-driven methodology and workflow process for providing translational research informatics support. J Biomed Inform 2009;42:377-381.
[21] Harris PA, Taylor R, Minor BL, Elliott V, Fernandez M, O'Neal L, et al. The REDCap consortium: Building an international community of software platform partners. J Biomed Inform 2019;95:103208.
[22] Giambartolomei C, Zhenli Liu J, Zhang W, Hauberg M, Shi H, Boocock J, et al. A Bayesian framework for multiple trait colocalization from summary association statistics. Bioinformatics 2018;34:2538-2545.
[23] Huang da W, Sherman BT, Lempicki RA. Bioinformatics enrichment tools: paths toward the comprehensive functional analysis of large gene lists. Nucleic Acids Res 2009;37:1-13.
[24] Guney E, Menche J, Vidal M, Barabasi AL. Network-based in silico drug efficacy screening. Nat Commun 2016;7:10331.
[25] Menche J, Sharma A, Kitsak M, Ghiassian SD, Vidal M, Loscalzo J, et al. Disease networks. Uncovering disease-disease relationships through the incomplete interactome. Science 2015;347:1257601.

**Appendix**

## STEP-BY-STEP GUIDE TO NETWORK-BASED IN SILICO DRUG EFFICACY SCREENING

Here is a step-by-step guide to carrying out the Network-based in silico drug efficacy screening approach used to derive the results in Table 3 and Supplementary Table 20. Note that the naming of directories, paths etc. described here (and contained within any scripts provided) is specific to the file system of the high performance computing (HPC) environment (https://services.ncl.ac.uk/itservice/research/hpc/) that we used to carry out the analysis. Therefore, these path names will need to be changed appropriately to carry out any analysis on your own system.

**Step 1:** Download the required packages from the emreg00 GitHub repository. Specifically, the required packages are the drugbox package (https://github.com/emreg00/drugbox), the proximity package (https://github.com/emreg00/proximity) and the toolbox package (https://github.com/emreg00/toolbox).

**Step 2:** Download the XML file “full_database.xml” listing information on drugs and their drug targets from DrugBank (https://go.drugbank.com/releases/latest). This requires you to register for a free account to obtain a username/password to download the file. We initially downloaded this file in June 2019; subsequently in February 2021 (while the manuscript was in revision) we re-downloaded the 2021-01-03 release

version of the file, and this 2021-01-03 release version is the version used to derive the final set of results presented.

**Step 3:** Use the Python 3 utilities provided in the drugbox package to parse the DrugBank XML file. On our file system, this involved typing the following commands:

```
#Start an interactive terminal session
srun --pty /bin/bash

#Change to the appropriate directory
cd ~/SOFTWARE/TOOLBOX/drugbox-master/drugbox-master

#Load Python 3
module load Anaconda3/5.0.1

#Start Python
python

#Point to directory containing the xml file
from drugbox import drugbank
file_name = "~/DRUGBANK-FEB2021/full_database.xml"

#choose an output filename
out_file = "targetsFeb2021.tsv"
drugbank.output_target_info(file_name, out_file)

#Quit Python
quit()
```

**Step 4:** Use the following R commands (within the R package obtainable from https://cran.r-project.org/) to read in the resulting parsed DrugBank file and write out the list of gene targets to the file "genesFeb2021.txt":

```
targ=read.delim("targetsFeb2021.tsv", sep="\t",header=T)
genes=levels(targ$Target)
write.table(data.frame(genes),file="genesFeb2021.txt",col.names=F,row.names=F,q
uote=F)
```

**Step 5:** Use Ensembl BioMart (https://grch37.ensembl.org/biomart/martview/96b5727e07c4736c651d1fcb273128a4) to convert the list of genes in "genesFeb2021.txt" to Entrez (NCBI) gene IDs. This involves the following steps:

Click new (top left)
Choose Ensembl Genes 97
Choose Human genes

Click filters (left)
Expand GENE
Check Input external references
Pick Gene Name(s) (or whatever the input IDs are)
Paste in list or Choose file

Click Attributes
Expand GENE
Choose data to retrieve
  Gene stable ID
  Gene stable ID version
  Gene name
Expand EXTERNAL to get Entrez (NCBI) gene ID

Click Results (top left)
Click Go (top right) to export to tsv file

**Step 6:** Use the following R commands to read the resulting exported file "Feb2021_mart_export.txt" into R, retain columns 3 and 4 (containing the Gene.name and EntrezGene.ID), and save only unique entries as an R object "genes3". Then remove any lines from "genes3" that have EntrezGene.ID listed as "NA" to create an R object "genes4":

```
genes=read.delim("Feb2021_mart_export.txt", sep="\t",header=T)
genes2=genes[,3:4]
genes3=unique(genes2)
genes4=genes3[!is.na(genes3$EntrezGene.ID),]
```

**Step 7:** Still within R, merge the resulting genes with the "targetsFeb2021.tsv" file, keeping them in the same order as in "targetsFeb2021.tsv, using the following R commands:

```
targ=read.delim("targetsFeb2021.tsv", sep="\t",header=T)
targ$order=1:length(targ$Name)
mg=merge(targ,genes4,by.x="Target",by.y="Gene.name",sort=F)
ord=mg[order(mg$order),]
```

**Step 8:** Still within R, use the following commands to output the list of Entrez (NCBI) IDs of the drug targets to a file "Feb2021_drug_nodes_from.txt", and the list of DrugBank IDs, drug names, gene names of drug targets and additional useful information about the drug effect on each target to a file "Feb2021_drug_nodes_full.txt". Note that the order of the drugs in these two files is in the same order as in "targetsFeb2021.tsv", except that no lines of output are produced for any drugs that have no drug targets listed.

```
levs=levels(ord$Drugbank.id)

for (i in 1:length(levs)) {
drugtargets=ord[ord$Drugbank.id==levs[i],]
if ((dim(drugtargets))[1]>0) {

write.table(t(as.character(drugtargets$EntrezGene.ID)),
file="Feb2021_drug_nodes_from.txt",col.names=F,row.names=F,sep=",",append=T)

drugtargets$Gene_Type_Action=paste(as.character(drugtargets$Target),as.characte
r(drugtargets$Type),
as.character(drugtargets$Action),sep="_")
full=data.frame(drugtargets$Drugbank.id[1],drugtargets$Name[1],t(as.character(drug
targets$Target)),
t(as.character(drugtargets$Gene_Type_Action)))
write.table(full,
file="Feb2021_drug_nodes_full.txt",col.names=F,row.names=F,sep=",",append=T)

}
}
```

**Step 9:** Use the provided Perl program “make6runfilesFeb2021.pl” to process the file “Feb2021_drug_nodes_from.txt” to create 6 helper files (botprot1.txt, botprot2.txt, … botprot6.txt) that will be useful for carrying out the analysis with the drugs divided into 6 separate batches (to speed up the analysis by running the batches in parallel). Specifically, on a Linux system, type:

```
./make6runfilesFeb2021.pl < Feb2021_drug_nodes_from.txt
```

**Step 10:** For each of the files “botprox*.txt”, use a text editor to manually remove the indent from each of the first four lines, so that the lines read:

```
d=99999
z=99999
mean=99999
sd=99999
```

Then add the required additional lines (described below) immediately above these four lines and save the file with a new filename - we chose to use “runfiftysix*Feb2021.txt”. This should result in 6 new helper files (runfiftysix1Feb2021.txt, runfiftysix2Feb2021.txt, … runfiftysix6Feb2021.txt). We chose to move these helper files into the subdirectory ~/SOFTWARE/TOOLBOX/PBC_DRUG_REPURPOSING/.

The required additional lines at the top of “runfiftysix*Feb2021.txt” need to specify the list of the Entrez (NCBI) IDs of the genes to be compared to the drug targets for each drug, a random number seed, and an output file name. We used the Ensembl BioMart resource (see Step 5 above) to derive the Entrez (NCBI) IDs of the 56 prioritized candidate genes for PBC as listed in Supplementary Table 18. We chose to use random number seeds (462461, 4624612, … 462466) for drug batches (1, 2, … 6)

respectively, with output file names chosen as (outfiftysix1Feb2021.txt, outfiftysix2Feb2021.txt … outfiftysix6Feb2021.txt), respectively. We specified that these output files were to be saved within the subdirectory ~/SOFTWARE/TOOLBOX/PBC_DRUG_REPURPOSING/. Thus, the top 7 lines of runfiftysix1Feb2021.txt should read:

```
node_to=["8600","92092","54790","7127","10666","23274","8651","7903","3575","178
8","283635","4790","3594","1235","5915","3665","8764","4664","643","8764","3665","
6775","2113","81615","79890","5450","80762","4137","9830","5890","115352","9966"
,"50615","283234","7297","3592","639","3398","22806","3696","64180","163486","94
0","6689","55765","10019","9145","941","3595","4137","23228","9844","57705","3663
","4137","7128","3394","3593","965","7132","23228"]

seednum=452461

f=open("~/SOFTWARE/TOOLBOX/PBC_DRUG_REPURPOSING/outfiftysix1Feb202
1.txt",'w')

d=99999
z=99999
mean=99999
sd=99999
```

with the top 7 lines of runfiftysix2Feb2021.txt, runfiftysix3Feb2021.txt, etc., taking an identical form, except for the required changes to the random number seeds and to the output file names.

**Step 11:** Carry out the network proximity analysis from within the directory that contains the required toolbox package functions (which on our system was ~/SOFTWARE/TOOLBOX/toolbox-master/toolbox-master), while making use of the Menche et al. (2015) network provided within the proximity package (which on our system was saved in ~/SOFTWARE/TOOLBOX/proximity-master/proximity-

master/data/network/network.sif). **Note that the functions in this step require use of Python 2 rather than Python 3**.

Specifically, we used the six provided job scripts (runfiftysix1Feb2021.sh, runfiftysix2Feb2021.sh, … runfiftysix6Feb2021.sh) that could be used within our HPC SLURM system to set off the six jobs (corresponding to the six batches of dugs) simultaneously, by typing:

```
sbatch runfiftysix1Feb2021.sh
sbatch runfiftysix2Feb2021.sh
sbatch runfiftysix3Feb2021.sh
sbatch runfiftysix4Feb2021.sh
sbatch runfiftysix5Feb2021.sh
sbatch runfiftysix6Feb2021.sh
```

Each job script needed to be saved in ~/SOFTWARE/TOOLBOX/toolbox-master/toolbox-master, along with the python file that the job script calls. We named these python files runfiftysix1Feb2021.py, runfiftysix2Feb2021.py, … runfiftysix6Feb2021.py. If carrying out the analysis without using a job script, one could simply type:

```
module load Anaconda2/5.0.1
python runfiftysix1Feb2021.py
python runfiftysix2Feb2021.py
python runfiftysix3Feb2021.py
python runfiftysix4Feb2021.py
python runfiftysix5Feb2021.py
python runfiftysix6Feb2021.py
```

The six python files provided (runfiftysix1Feb2021.py, runfiftysix2Feb2021.py, … runfiftysix6Feb2021.py) import various required functions from the toolbox package,

read in the Menche et al. (2015) network file and then execute the commands contained in the helper files (runfiftysix1Feb2021.txt, runfiftysix2Feb2021.txt, … runfiftysix6Feb2021.txt) that were created in Step 10.

**Step 12:** Once all six jobs have finished, concatenate the results into a single file using the command:

```
cat outfiftysix1Feb2021.txt outfiftysix2Feb2021.txt outfiftysix3Feb2021.txt
outfiftysix4Feb2021.txt outfiftysix5Feb2021.txt outfiftysix6Feb2021.txt >
outfiftysixFullFeb2021.txt
```

from within the directory where the files had been saved (which on our system was ~/SOFTWARE/TOOLBOX/PBC_DRUG_REPURPOSING/).

**Step 13:** The output file "outfiftysixFullFeb2021.txt" contains four columns of results (d, z, mean, sd), corresponding to the distance $d_c$, z-score $z = (d_c-\mu)/\sigma$, empirical mean $\mu$ and empirical standard deviation $\sigma$ (see Supplementary Text) between the set of prioritized PBC genes and the relevant drug. These outputs should be in exactly the same order as the drugs (and additional information) listed in the previously created (from Step 8) file "Feb2021_drug_nodes_full.txt". However, there is the slight complication that "outfiftysixFullFeb2021.txt" contains a variety of extraneous commas and left and right brackets, in addition to the space delimiter separating the columns. In principle, one could use a variety of command line tools (including R or Perl scripts) to process the file "outfiftysixFullFeb2021.txt" to get rid of these and then combine it appropriately column-wise with "Feb2021_drug_nodes_full.txt". We used a simpler approach of opening both files in Excel, copying and pasting the four columns from

“outfiftysixFullFeb2021.txt” into four (newly inserted) columns in "Feb2021_drug_nodes_full.txt", and then using the “Find and Replace” function within Excel to remove extraneous commas and left and right brackets from the relevant four columns.

**Step 14:** To produce an ordered list of drugs (with the drugs that have the most negative drug-disease proximity measure listed at the top), one could simply sort the Excel file produced at the end of Step 13 on the relevant z-score column. However, the very large number of gene targets for some drugs (resulting in a very large number of columns that would need to be highlighted while sorting) makes this procedure somewhat error-prone within Excel. We recommend instead saving the Excel file produced at the end of Step 13 (with appropriate column headers) as a tab delimited text file (e.g., named “PBC_Feb2021_drug_resultsTAB.txt”), and then using the following commands in R to do the ordering:

```
new=read.delim("PBC_Feb2021_drug_resultsTAB.txt", sep="\t",header=T)
newsorted=new[order(new$z),]
write.table(newsorted,file="sortedPSC_Feb2021_drug_resultsTAB.txt",
sep="\t",col.names=T,row.names=F,quote=F)
```
