# Supplementary material for: An international genome-wide meta-analysis of primary biliary cholangitis: Novel risk loci and candidate drugs
Source: J Hepatol. 2021 Sep;75(3):572–81. doi: 10.1016/j.jhep.2021.04.055 (PMC8811537; doi:10.1016/j.jhep.2021.04.055)
Supplement: Multimedia component 2 [file mmc2.zip › STEP_BY_STEP_SCRIPTS/StepByStepGuide.pdf]

STEP-BY-STEP GUIDE TO NETWORK-BASED IN SILICO DRUG EFFICACY SCREENING

Here is a step-by-step guide to carrying out the *Network-based in silico drug efficacy screening* approach used to derive the results in Table 3 and Supplementary Table 20. Note that the naming of directories, paths etc. described here (and contained within any scripts provided) is specific to the file system of the high performance computing (HPC) environment (https://services.ncl.ac.uk/itservice/research/hpc/) that we used to carry out the analysis. Therefore, these path names will need to be changed appropriately in order to carry out any analysis on your own system

Step 1: Download the required packages from the emreg00 GitHub repository. Specifically, the required packages are the drugbox package (https://github.com/emreg00/drugbox), the proximity package (https://github.com/emreg00/proximity) and the toolbox package (https://github.com/emreg00/toolbox).

Step 2: Download the XML file "full_database.xml" listing information on drugs and their drug targets from DrugBank (https://go.drugbank.com/releases/latest). This requires you to register for a free account to obtain a username/password in order to download the file. We initially downloaded this file in June 2019; subsequently in February 2021 (while the manuscript was in revision) we re-downloaded the 2021-01-03 release version of the file, and this 2021-01-03 release version is the version used to derive the final results presented.

Step 3: Use the Python 3 utilities provided in the drugbox package to parse the DrugBank XML file. On our file system, this involved typing the following commands:

```
srun --pty /bin/bash                                     #Start an interactive terminal session
cd ~/SOFTWARE/TOOLBOX/drugbox-master/drugbox-master      #Change to the appropriate directory
module load Anaconda3/5.0.1                              #Load Python 3
python                                                   #Start Python
from drugbox import drugbank
file_name = "~/DRUGBANK-FEB2021/full_database.xml"       #Point to directory containing the xml file
out_file = "targetsFeb2021.tsv"                          #choose an output filename
drugbank.output_target_info(file_name, out_file)
quit()                                                   #Quit Python
```

Step 4: Use the following R commands (within the R package obtainable from https://cran.r-project.org/) to read in the resulting parsed DrugBank file and write out the list of gene targets to the file "genesFeb2021.txt":

```
targ=read.delim("targetsFeb2021.tsv", sep="\t",header=T)
genes=levels(targ$Target)
write.table(data.frame(genes),file="genesFeb2021.txt",col.names=F,row.names=F,quote=F)
```

Step 5: Use Ensembl BioMart (https://grch37.ensembl.org/biomart/martview/96b5727e07c4736c651d1fcb273128a4) to convert the list of genes in "genesFeb2021.txt" to Entrez (NCBI) gene IDs. This involves the following steps:

```
Click new (top left)
Choose Ensembl Genes 97
Choose Human genes

Click filters (left)
Expand GENE
Check Input external references
Pick Gene Name(s) (or whatever the input IDs are)
Paste in list or Choose file

Click Attributes
Expand GENE
Choose data to retrieve
        Gene stable ID
        Gene stable ID version
        Gene name
Expand EXTERNAL to get Entrez (NCBI) gene ID

Click Results (top left)
Click Go (top right) to export to tsv file
```

Step 6: Use the following R commands to read the resulting exported file “Feb2021_mart_export.txt” into R, retain columns 3 and 4 (containing the Gene.name and EntrezGene.ID), and save only unique entries as an R object “genes3”. Then remove any lines from “genes3” that have EntrezGene.ID listed as “NA” to create an R object “genes4”:

```
genes=read.delim("Feb2021_mart_export.txt", sep="\t",header=T)
genes2=genes[,3:4]
genes3=unique(genes2)
genes4=genes3[!is.na(genes3$EntrezGene.ID),]
```

Step 7: Still within R, merge the resulting genes with the "targetsFeb2021.tsv" file, keeping them in the same order as in "targetsFeb2021.tsv, using the following R commands:

```
targ=read.delim("targetsFeb2021.tsv", sep="\t",header=T)
targ$order=1:length(targ$Name)
mg=merge(targ,genes4,by.x="Target",by.y="Gene.name",sort=F)
ord=mg[order(mg$order),]
```

Step 8: Still within R, use the following commands to output the list of Entrez (NCBI) IDs of the drug targets to a file "Feb2021_drug_nodes_from.txt", and the list of DrugBank IDs, drug names, gene names of drug targets and additional useful information about the drug effect on each target to a file "Feb2021_drug_nodes_full.txt". Note that the order of the drugs in these two files is in the same order as in "targetsFeb2021.tsv", except that no lines of output are produced for any drugs that have no drug targets listed.

```
levs=levels(ord$Drugbank.id)

for (i in 1:length(levs)) {
drugtargets=ord[ord$Drugbank.id==levs[i],]
if ((dim(drugtargets))[1]>0) {

write.table(t(as.character(drugtargets$EntrezGene.ID)),
file="Feb2021_drug_nodes_from.txt",col.names=F,row.names=F,sep=",",append=T)

drugtargets$Gene_Type_Action=paste(as.character(drugtargets$Target),as.character(drugtargets$Type),
as.character(drugtargets$Action),sep="_")
full=data.frame(drugtargets$Drugbank.id[1],drugtargets$Name[1],t(as.character(drugtargets$Target)),
t(as.character(drugtargets$Gene_Type_Action)))
write.table(full, file="Feb2021_drug_nodes_full.txt",col.names=F,row.names=F,sep=",",append=T)

}
}
```

Step 9: Use the provided Perl program “make6runfilesFeb2021.pl” to process the file “Feb2021_drug_nodes_from.txt” to create 6 helper files (botprot1.txt, botprot2.txt, … botprot6.txt) that will be useful for carrying out the analysis with the drugs divided into 6 separate batches (in order to speed up the analysis by running the batches in parallel). Specifically, on a Linux system, type:

```
./make6runfilesFeb2021.pl < Feb2021_drug_nodes_from.txt
```

Step 10: For each of the files “botprox*.txt”, use a text editor to manually remove the indent from each of the first four lines, so that the lines read:

```
d=99999
z=99999
mean=99999
sd=99999
```

Then add the required additional lines (described below) immediately above these four lines, and save the file with a new filename - we chose to use “runfiftysix*Feb2021.txt”. This should result in 6 new helper files (runfiftysix1Feb2021.txt, runfiftysix2Feb2021.txt, … runfiftysix6Feb2021.txt). We chose to move these helper files into the subdirectory ~/SOFTWARE/TOOLBOX/PBC_DRUG_REPURPOSING/.

The required additional lines at the top of “runfiftysix*Feb2021.txt“ need to specify the list of the Entrez (NCBI) IDs of the genes to be compared to the drug targets for each drug, a random number seed, and an output file name. We used the Ensembl BioMart resource (see Step 5 above) to derive the Entrez (NCBI) IDs of the 56 prioritized candidate genes for PBC as listed in Supplementary Table 18. We chose to use random number seeds (462461, 4624612, … 462466) for drug batches (1, 2, … 6) respectively, with output file names chosen as (outfiftysix1Feb2021.txt, outfiftysix2Feb2021.txt … outfiftysix6Feb2021.txt), respectively. We specified that these output files were to be saved within the subdirectory ~/SOFTWARE/TOOLBOX/PBC_DRUG_REPURPOSING/. Thus, the top 7 lines of runfiftysix1Feb2021.txt should read:

```
node_to=["8600","92092","54790","7127","10666","23274","8651","7903","3575","1788","283635","4790","3594","1235","5915","
3665","8764","4664","643","8764","3665","6775","2113","81615","79890","5450","80762","4137","9830","5890","115352","9966"
,"50615","283234","7297","3592","639","3398","22806","3696","64180","163486","940","6689","55765","10019","9145","941","3
595","4137","23228","9844","57705","3663","4137","7128","3394","3593","965","7132","23228"]

seednum=452461

f=open("~/SOFTWARE/TOOLBOX/PBC_DRUG_REPURPOSING/outfiftysix1Feb2021.txt",'w')

d=99999
z=99999
mean=99999
sd=99999
```

with the top 7 lines of runfiftysix2Feb2021.txt, runfiftysix3Feb2021.txt etc. taking an identical form, except for the required changes to the random number seeds and to the output file names.

Step 11: Carry out the network proximity analysis from within the directory that contains the required toolbox package functions (which on our system was ~/SOFTWARE/TOOLBOX/toolbox-master/toolbox-master), while making use of the Menche *et al*. (2015) network provided within the proximity package (which on our system was saved in ~/SOFTWARE/TOOLBOX/proximity-master/proximity-master/data/network/network.sif).
Note that the functions in this step require use of Python 2 rather than Python 3.

Specifically, we used the six provided job scripts (runfiftysix1Feb2021.sh, runfiftysix2Feb2021.sh, … runfiftysix6Feb2021.sh) that could be used within our HPC SLURM system to set off the six jobs (corresponding to the six batches of dugs) simultaneously, by typing:

```
sbatch runfiftysix1Feb2021.sh
sbatch runfiftysix2Feb2021.sh
sbatch runfiftysix3Feb2021.sh
sbatch runfiftysix4Feb2021.sh
sbatch runfiftysix5Feb2021.sh
sbatch runfiftysix6Feb2021.sh
```

Each job script needed to be saved in ~/SOFTWARE/TOOLBOX/toolbox-master/toolbox-master, along with the python file that the job script calls. We named these python files runfiftysix1Feb2021.py, runfiftysix2Feb2021.py, … runfiftysix6Feb2021.py. If carrying out the analysis without using a job script, one could simply type:

```
module load Anaconda2/5.0.1

python runfiftysix1Feb2021.py
python runfiftysix2Feb2021.py
python runfiftysix3Feb2021.py
python runfiftysix4Feb2021.py
python runfiftysix5Feb2021.py
python runfiftysix6Feb2021.py
```

The six python files provided (runfiftysix1Feb2021.py, runfiftysix2Feb2021.py, … runfiftysix6Feb2021.py) import various required functions from the toolbox package, read in the Menche *et al*. (2015) network file and then execute the commands contained in the helper files (runfiftysix1Feb2021.txt, runfiftysix2Feb2021.txt, … runfiftysix6Feb2021.txt) that were created in Step 10.

Step 12: Once all six jobs have finished, concatenate the results into a single file using the command:

```
cat outfiftysix1Feb2021.txt outfiftysix2Feb2021.txt outfiftysix3Feb2021.txt outfiftysix4Feb2021.txt
outfiftysix5Feb2021.txt outfiftysix6Feb2021.txt > outfiftysixFullFeb2021.txt
```

from within the directory where the files had been saved (which on our system was ~/SOFTWARE/TOOLBOX/PBC_DRUG_REPURPOSING/).

Step 13: The output file "outfiftysixFullFeb2021.txt" contains four columns of results (d, z, mean, sd), corresponding to the distance d_c, z-score $z = (d_c-\mu)/\sigma$, empirical mean μ and empirical standard deviation σ (see Supplementary Text) between the set of prioritized PBC genes and the relevant drug. These outputs should be in exactly the same in the same order as the drugs (and additional information) listed in the previously-created (from Step 8) file "Feb2021_drug_nodes_full.txt". However, there is the slight complication that "outfiftysixFullFeb2021.txt" contains a variety of extraneous commas and left and right brackets, in addition to the space delimiter separating the columns. In principle, one could use a variety of command line tools (including R or Perl scripts) to process the file "outfiftysixFullFeb2021.txt" to get rid of these and then combine it appropriately column-wise with "Feb2021_drug_nodes_full.txt". We used a simpler approach of opening both files in Excel, copying and pasting the four columns from "outfiftysixFullFeb2021.txt" into four (newly inserted) columns in "Feb2021_drug_nodes_full.txt", and then using the "Find and Replace" function within Excel to remove extraneous commas and left and right brackets from the relevant four columns.

Step 14: To produce an ordered list of drugs (with the drugs that have the most negative drug-disease proximity measure listed at the top), one could simply sort the Excel file produced at the end of Step 13 on the relevant z-score column. However, the very large number of gene targets for some drugs (resulting in a very large number of columns that would need to be highlighted while sorting) makes this procedure somewhat error-prone within Excel. We recommend instead saving the Excel file produced at the end of Step 13 (with appropriate column headers) as a tab delimited text file (e.g. named "PBC_Feb2021_drug_resultsTAB.txt"), and then using the following commands in R to do the ordering:

```
new=read.delim("PBC_Feb2021_drug_resultsTAB.txt", sep="\t",header=T)
newsorted=new[order(new$z),]
write.table(newsorted,file="sortedPSC_Feb2021_drug_resultsTAB.txt", sep="\t",col.names=T,row.names=F,quote=F)
```
