# Supplementary material for: An international genome-wide meta-analysis of primary biliary cholangitis: Novel risk loci and candidate drugs
Source: J Hepatol. 2021 Sep;75(3):572–81. doi: 10.1016/j.jhep.2021.04.055 (PMC8811537; doi:10.1016/j.jhep.2021.04.055)
Supplement: Multimedia component 3 [file mmc3.pdf]

JOURNAL
OF HEPATOLOGY

Journal of Hepatology

CTAT methods

Tables for a "Complete, Transparent, Accurate and Timely account" (CTAT) are now mandatory for all revised submissions. The aim is to enhance the reproducibility of methods.

- Only include the parts relevant to your study
- Refer to the CTAT in the main text as 'Supplementary CTAT Table'
- Do not add subheadings
- Add as many rows as needed to include all information
- Only include one item per row

If the CTAT form is not relevant to your study, please outline the reasons why:

1.1 Antibodies

Name	Citation	Supplier	Cat no.	Clone no.

1.2 Cell lines

Name	Citation	Supplier	Cat no.	Passage no.	Authentication test method

1.3 Organisms

Name	Citation	Supplier	Strain	Sex	Age	Overall n number

1.4 Sequence based reagents

Name	Sequence	Supplier

1.5 Biological samples

Description	Source	Identifier

1.6 Deposited data

Name of repository	Identifier	Link
1000 Genomes Phase 3 panel		ftp://ftp.1000genomes.ebi.ac.uk/vol1/ftp/release/20130502/;

1000 Genomes Phase 3 panel		http://csg.sph.umich.edu/abecasis/MACH/download/1000G.Phase3.v5.html
ALSPAC		http://www.bristol.ac.uk/alspac/
INTERVAL		http://www.donorhealth-btru.nihr.ac.uk/studies/interval-study/
GTEx		https://www.gtexportal.org/home
GWMA data		Summary statistics will be deposited with the European Genome phenome Archive (EGA, https://ega-archive.org/) after publication

1.7 Software

Software name	Manufacturer	Version
PLINK	http://zzz.bwh.harvard.edu/plink/	v1.07
Michigan Imputation Server	https://imputationserver.sph.umich.edu/index.html#!	v1.2.4
SHAPEIT	https://mathgen.stats.ox.ac.uk/genetics_software/shapeit/shapeit.html	
IMPUTE2	https://mathgen.stats.ox.ac.uk/impute/impute_v2.html	v2
GMMAT	R package authored by Han Chen (Han.Chen.2@uth.tmc.edu)	v1.3.1
META	https://mathgen.stats.ox.ac.uk/genetics_software/meta/meta.html	v1.7
FINEMAP	http://www.christianbenner.com/	v1.4
COJO	http://cnsgenomics.com/software/gcta/	v1.91.7
FUMA GWAS	https://fuma.ctglab.nl/	
MetaXcan	https://github.com/hakyimlab/MetaXcan	
STRING	https://string-db.org/	v11.0
DAVID	https://david.ncifcrf.gov/conversion.jsp?VFROM=NA	v6.8

1.8 Other (e.g. drugs, proteins, vectors etc.)

1.9 Please provide the details of the corresponding methods author for the manuscript:

Heather J. Cordell
Professor of Statistical Genetics
Newcastle University
International Centre for Life
Central Parkway
Newcastle upon Tyne
NE1 3BZ, UK
heather.cordell@newcastle.ac.uk

2.0 Please confirm for randomised controlled trials all versions of the clinical protocol are included in the submission. These will be published online as supplementary information.

